# Visual acuity improvement in children with albinism beyond the first decade of life

**DOI:** 10.1371/journal.pone.0296744

**Published:** 2024-01-17

**Authors:** Claudia Yahalom, Ana Navarrete, Atara Juster, Ayan Galbinur, Anat Blumenfeld, Karen Hendler

**Affiliations:** 1 Faculty of Medicine, Hebrew University of Jerusalem, Jerusalem, Israel; 2 Department of Ophthalmology, Hadassah Medical Center, Jerusalem, Israel; LV Prasad Eye Institute, INDIA

## Abstract

**Purpose:**

To determine if visual maturation continues beyond the first decade of life in children with albinism and whether this is related to albinism type, presence of nystagmus, eye muscle surgery or refractive errors.

**Design:**

Case series based on retrospective study of children with confirmed genetic diagnosis of albinism.

**Methods:**

Clinical data were obtained from medical files of children examined during school years, including albinism type, visual acuity, eye muscle surgery, nystagmus, and others on different visits (Visit 1: ages 7–9; Visit 2: ages: 10–12; Visit 3: ages 13–16; Visit 4: ages >16).

**Results:**

Seventy-five children with albinism were included in the study. Patients were divided into different groups according to the albinism type including OCA1A: 17; OCA1B: 28; OCA2: 26; HPS: 3; OCA4: 1. Follow-up ranged from 3–13 years. Progressive visual acuity improvement was seen in all three main groups. T-test paired samples showed a statistically significant improvement when comparing vision from Visit 1 and Visit 3 in both OCA1A and OCA2 groups, with a mean vision improvement of 2 lines. There was no correlation between visual improvement and refractive error, eye muscle surgery or nystagmus.

**Conclusion:**

An improved visual performance was seen in a large percentage of children with albinism during the second decade of life. The reason for this late improvement in vision is not clear but may be related to late foveal maturation or improvement in nystagmus with time. This information is useful for clinicians of these patients and when counseling parents.

## Introduction

Oculocutaneous albinism (OCA) is an autosomal recessive disorder, characterized by a partial or complete absence of the melanin pigment in the skin, hair, and eyes [1].

It has a prevalence of approximately 1:14,000 worldwide [1] while in Israel–at least 1:10,000 [2]. To date, 8 genes have been identified which cause OCA 1–8 [1, 3, 4]. Prevalence of the different types of albinism varies according to the ethnicity; OCA1 is the most common subtype in Caucasians (around 50% of cases worldwide). OCA1 is caused by a mutation in the tyrosinase gene on chromosome 11q. Affected patients without ability to produce any melanin (OCA1A) typically have visual acuity ranging from 20/100 to 20/400, whereas those who can make some pigment due to residual enzyme function (OCA1B) often have better vision [5].

OCA2 is the most common subtype in Africa (around 30% of cases worldwide) with a visual acuity that usually ranges from 20/40 to 20/60. It is produced by mutations in the OCA2 gene on chromosome 15q. Individuals with OCA2 have abnormal function of a membrane protein essential for melanogenesis and typically have better vision than individuals with OCA1 [5].

OCA4 (around 17% of albinos worldwide) is the most common type in Japan [6].

The degree of hypopigmentation varies in albinism, ranging from no pigment to nearly normal pigment. Visual impairment varies as well and is generally related to the type of albinism and the severity of hypopigmentation [7].

The most disabling feature of albinism is the visual impairment, usually present from birth. The visual potential in albinism is usually limited, probably due to the combination of foveal hypoplasia, nystagmus and photophobia [7].

Some cases of improved vision in persons with albinism have been reported in the past, but details about the amount of improvement and related factors are not available [8, 9].

The aims of our study were to evaluate whether visual development of children with albinism continues to occur beyond the first decade of life and at what age full visual potential is reached.

## Methods

A retrospective cohort study design was used to evaluate visual performance of children with genetically confirmed diagnosis of albinism, who were seen at the Michaelson Institute for Low Vision, Department of Ophthalmology, Hadassah Medical Center in Jerusalem, between January 2008 and December 2021. Approval for this study, according to the Helsinki tenets, was obtained from the Institutional Review Board at Hadassah-Hebrew University Medical Center, Jerusalem.

About 400 children with albinism from all over the country are examined annually in our low vision institute, which is a national center for visually impaired children in Israel. Evaluation is carried out by a multidisciplinary team including optometrists, social workers, genetic counselors, and ophthalmologists. Data were collected from medical records including genotype, visual acuity (VA), refraction, presence of strabismus or nystagmus, and history of eye muscle surgery. Visual acuities were recorded at 4 different visits at specified ages: visit 1 (V1) at ages 7–9; visit 2 (V2) at ages 10–12; Visit 3 (V3) at ages 13–16 and visit 4 (V4) at age >16 years. A time zone of at least two years was kept between recorded visits. Inclusion criteria included children with genetically confirmed albinism aged 7 years or older, with at least 3 years of follow up and 2 or more visits. Snellen linear numbers were used for visual acuity testing. Vision was recorded as binocular best corrected visual acuity (B-BCVA). Preferred head posture was allowed for binocular acuity testing.

### Statistical analysis

The characteristics of the study cohorts are expressed as means +/- SD for age and VA (on a logMAR scale).

To explore the data behavior, the Shapiro-Wilk test of normality was run.

To examine the relationship between two categorical variables, Chi-square (χ2) and Fisher’s exact test were used. Comparison of quantitative variables was performed using a T-test paired test for normal data. The asymmetric Mann-Whitney test and Friedman’s Two-Way Analysis of Variance were used in case of non-normal distribution.

Statistical analyses were performed using IBM SPSS software V.26. Significance levels were set as p < 0.05.

## Results

Seventy-five children with both clinical and genetic diagnosis (two pathogenic/likely pathogenic variants identified in specific genes) of albinism were included in the study (OCA1A: 17; OCA1B: 28; OCA2: 26; Hermansky Pudlak syndrome (HPS): 3; OCA4: 1). Follow up ranged from 3–13 years (mean 8.8). HPS (3 children) and OCA4 (one child) groups were too small to reach conclusions and were not included in the statistical analysis.

B-BCVA improved in the interval between V1 and V2 in all three main groups (OCA1A, OCA1B and OCA2), with further improvement in vision in the OCA1A and OCA2 groups in V3. (Table 1).

**Table 1:** Clinical characteristics in 3 main groups (OCA1A, OCA1B and OCA2) over time.

Albinism type	V1 Vision 7–9 years	V2 Vision 10–12 years	V3 Vision 13–16 years	V4 Vision >16 years	Follow-up (years)	Age last exam
**OCA1A**						
N. patients	16	16	8	5	17	17
Mean*	0.95	0.86	0.71	0.69(0.21)	10 (2.09)	14 (3.3)
(SD)	(0.32)	(0.20)	(0.14)	0.35–0.90	5–13	10–22
Min-Max	0.6–2	0.5–1.3	0.4–0.9	6/30		
Mean	6/48	6/36	6/30			
Snellen						
**OCA1B**						
N. patients	26	28	13	5	28	28
Mean*	0.60	0.51	0.50	0.60(0.12)	8.74(2.1)	13.2(2.9)
(SD)	(0.16)	(0.19)	(0.22)	0.4–0.7	3–12	10–19
Min-Max	0.3–1	0.2–1	0.1–0.8	6/24		
Mean	6/24	6/18	6/18			
Snellen						
**OCA2**						
N. patients	23	24	12	9	26	26
Mean*	0.70	0.62	0.53	0.52(0.23)	7.92(2.3)	13(3.1)
(SD)	(0.18)	(0.19)	(0.22)	0.1–0.8	3–13	10–19
Min-Max	0.3–1	0.3–1.1	0.1–0.8	6/18		
Mean	6/30	6/24	6/18			
Snellen						

V1: Visit 1, V2: visit 2, V3: visit 3, V4: visit 4

*: Visual acuity in LogMar

SD: standard deviation

Patients with albinism types OCA1B and OCA2 had the best final visual outcomes (mean vision 6/18; LogMar 0.5; SD 0.2) reaching maximum VA by age 10–12 in the OCA1B group and by 13–16 years in the OCA2 group (Table 1).

Paired samples t-test (corrected for multiple comparisons) showed statistically significant improvement when comparing vision in OCA2 group from V1 to V3 and from V2 to V4 (0.04 and 0.03 respectively) (Table 2).

**Table 2:** Paired samples T-test.

Albinism type			Paired differences		Significance 2-tailed))	Adjusted p-value Bonferroni))
			Mean	SD		
**OCA1A**	Pair 1	V1-V2	0.81	0.19	0.109	0.436
	Pair 2	V2-V3	0.42	0.09	0.289	1.156
	Pair 3	V1-V3	0.10	0.08	**0.018** *	0.072
	Pair 4	V1-V4	-0.09	0.12	0.235	0.94
	Pair 5	V2-V4	0.06	0.08	0.194	0.776
	Pair 6	V3-V4	0.01	0.07	0.778	3.112
**OCA2**	Pair 1	V1-V2	0.08	0.11	**0.03** *	0.12
	Pair 2	V2-V3	0.06	0.06	**0.02** *	0.08
	Pair 3	V1-V3	0.14	0.08	**0.01** *	**0.04** *
	Pair 4	V1-V4	-0.14	0.10	**0.019** *	0.076
	Pair 5	V2-V4	0.11	0.07	**0.008** *	**0.032** *
	Pair 6	V3-V4	0.03	0.04	0.095	0.38

Statistically significant values*

Parametric tests could not be used in OCA1B group because test of normality (Kolmogorov-Smirnov) showed that the variable V1-V2_difference was not normal. A non- parametric test (Friedman’s Two-Way analysis) was used for global significance in OCA1B group and showed that visual acuity improved with time but did not reach statistical significance (0.127).

Strabismus incidence was high in all 3 main groups but extraocular muscle surgery was performed more and at a relatively younger age in the OCA1A group (Table 3).

**Table 3:** Related clinical factors in 3 main groups (OCA1A, OCA1B and OCA2).

	Eye muscle surgery			Strabismus		Refractive error		
	No	Yes/%	Mean age at sx (y)	None	Strabismus (%)	Hypermetropia	Myopia	Cylinder
**OCA1A**	5	12/70.5	4.00	6	11 (64.)	13	4	16
**OCA1B**	17	11/39.2	5.73	8	19 (67.8)	23	5	21
**OCA2**	14	12/46.1	6.42	8	18 (69.2)	19	7	20
**Total**	36	35		22	48 (67.6)	55	16	57

The interval between V1 and V2 was studied with non-parametric tests (Mann-Whitney U test; Kruskal-Walis test) in order to see the significance of factors that could have been related to further maturation of visual acuity with time. There was no significant effect of extraocular muscle surgery, nystagmus, or refractive errors on change in vision.

Mean visual acuity in children without nystagmus was significantly better in both OCA1B group and OCA2 groups but visual improvement with time, was similar to children with nystagmus (Table 4).

**Table 4:** Visual acuities in 3 main groups in relation with nystagmus.

Albinism type	Nystagmus (number)	V1 LogMar	V2 LogMar	V3 LogMar	V4 LogMar
**OCA1A**	No (0)				
	Yes (17)	0.95	0.87	0.71	0.69
**OCA1B**	No (3)	0.53	0.43	0.30	-
	Yes (25)	0.61	0.53	0.52	0.60
**OCA2**	No (2)	0.30	0.30	0.10	0.10
	Yes (24)	0.72	0.64	0.57	0.58

Of the 75 children included in the study, visual acuity improved from first to last visit in 50 (66.6%), stayed stable in 20 (26.6%) and decreased in 5 patients (6.6%). No explanation for decreased vision was detected in these patients. Further analysis of visual acuities revealed that significant improvement (2 to 3 lines) was recorded in 28 (37.3%) patients, mainly in OCA1B and OCA2 groups. Table 5.

**Table 5:** Change in visual acuity in the studied groups.

Improvement in vision in lines (LogMar)	OCA1A	OCA1B	OCA2	OCA4	HPS	Total
**None**	4	5	8		3	20
**1 line**	8	8	6			22
**2 lines**	1	10	7	1		19
**3 lines**	2	4	3			9
**Lost 1–2 lines**	2	1	2			5
**Total**	17	28	26	1	3	75

**Fig 1:**
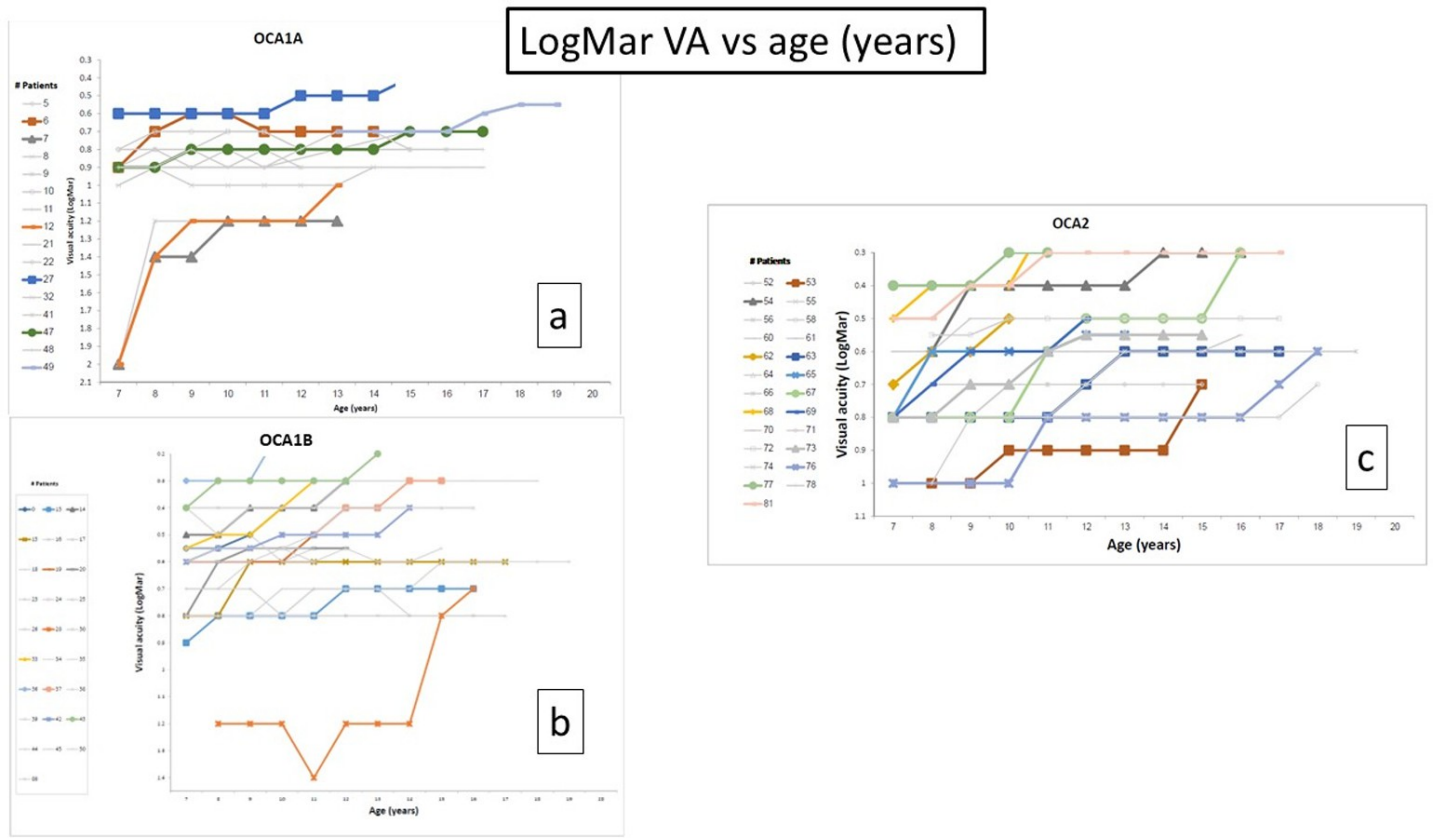
Individual LogMar VA plotted against age. Individual representation of the log MAR VA plotted against age, upon different time intervals between exams, for each patient. Gray lines represent patients with no significant VA improvement, colored lines represent patients with VA improvement of 2 lines or more during follow-up time. Two patients (one from OCA1A and OCA2 groups respectively), were removed from the graph due to lost VA lines, because of interference with graphic representation of rest of VA.

A graphic representation of the log MAR VA plotted against age, upon different time intervals for each child, is shown in Fig 1.

## Discussion

Defining when visual function in normal seeing children is completely "adult-like" has been addressed in the literature and, even though it is difficult to establish, it is believed that visual maturation is achieved around age 6 years. Ellemberg published that VA is similar to an adult value of 6/6 by the age of 5 years [10]. Lewis and Maurer suggested that grating acuity is adult-like by 4–6 years and letter acuity is adult-like by 6 years of age [11]. The age at which children with albinism reach mature vision (adult-like vision) seems to occur later in life, beyond the first decade, when compared with children with no eye pathology [9]. The reason for this late improvement in vision is not clear but may be related to late maturation of the macular area, or improvement in nystagmus with time, or gain of macular pigment, or a combination of the above.

Our study includes a relatively large cohort of 75 children with genetically confirmed albinism with a long follow up (mean 8.8+- 2.1 years). It is of importance to highlight that long term ophthalmic follow up is standard practice for all children with albinism in order to monitor changing significant refractive errors, as well as development of strabismus and/or head posture that might require surgical treatment.

In our study, visual acuity improved in 66.6% of studied children over the school years, although clinically significant improvement (2–3 Snellen lines gain in vision) for an individual child was reached only in 37.3% of studied children and it was noted mainly in OCA1B and OCA2 groups. The inability to assess each patient longitudinally but rather group patients together for specific periods of time was a restriction. To try to obtain a clearer idea of VA change through the studied period, we included an individual analysis of VA change at each exam point, for each studied child against time in years.

Possible related factors such as refractive errors, nystagmus, and eye muscle surgery did not show any significant correlation with visual improvement.

Previous studies have mentioned improvement in visual acuities in children with albinism beyond the age of 6 years. Summers and King studied a cohort of 9 individuals with OCA1B and noted a significant improvement in vision with time in one patient [12]. Blohmé and Tornqvist conducted another study with almost 2 decades of follow up, on a cohort of 9 patients with albinism. Among them 5/9 patients had a significant visual improvement that qualified for exclusion from low vision range [13].

Dijkstal et al published in 2012 further data suggesting late visual acuity improvement in children with albinism. They described an improved visual acuity during the early school years in 80% of the 65 studied children. They also concluded that ocular melanin pigment, particularly in the macula, is related to a relatively better level of measured visual acuity for OCA1B, but such could not be shown to be related to the improvement in B-BCVA [9].

Our study restrictions include its retrospective nature, lack of data such as optical coherence tomography (OCT), pigmentation grade, and lack of a significant number of patients for the last visit (V4) analysis.

We believe that our study supports the clinical feeling of ophthalmologists who follow many patients with albinism for many years and embellishes previous studies which mainly focused on younger patients.

In conclusion, our results show that VA in children with albinism continues to improve into their teen years in around 2/3 of patients, reaching final "adult-like" vision later during the second decade of life.

Our work provides useful information about the natural history of VA in a relatively large sample of individuals with varying forms of albinism. The differences found for the different genotypes provide useful information for clinicians in counseling these patients and their parents.

## Supporting information

S1 Table Visual acuity over time and clinical characteristics in studied patients. (XLSX)

S1 Data Inferential statistics_part 1: Statistical analysis, T-test, paired samples correlations. (HTML)

S2 Data Inferential statistics_part 2: Case processing summary. (HTML)

S3 Data Descriptive statistics: Clinical characteristics in studied patients. (HTML)
