# Supplementary material for: Visual acuity improvement in children with albinism beyond the first decade of life
Source: PLoS One. 2024 Jan 17;19(1):e0296744. doi: 10.1371/journal.pone.0296744 (PMC10793880; doi:10.1371/journal.pone.0296744)
Supplement: S1 Data — (HTML) [file pone.0296744.s002.html]

Inferential Statistics\_new1


|  |  |  |
| --- | --- | --- |
| IBM SPSS Web Report - Inferential Statistics\_new1.spv     ---   Contents  Previous  Next  Help |  | Not connected to the server     --- |

- Log

  - Log
- Nonparametric Tests

  - Hypothesis Test Summary
  - Independent-Samples Mann-Whitney U Test

    - V1\_V2\_Diff across nystagmus

      - Independent-Samples Mann-Whitney U Test Summary
      - Independent-Samples Mann-Whitney U Test
  - Continuous Field Information V1\_V2\_Diff
  - Categorical Field Information nystagmus
- Log

  - Log
- Nonparametric Tests

  - Hypothesis Test Summary
  - Independent-Samples Mann-Whitney U Test

    - V1\_V2\_Diff across Eye muscle surgery

      - Independent-Samples Mann-Whitney U Test Summary
      - Independent-Samples Mann-Whitney U Test
  - Continuous Field Information V1\_V2\_Diff
  - Categorical Field Information Eye muscle surgery
- Log

  - Log
- Nonparametric Tests

  - Hypothesis Test Summary
  - Independent-Samples Kruskal-Wallis Test

    - V1\_V2\_Diff across Srabismus

      - Independent-Samples Kruskal-Wallis Test Summary
      - Independent-Samples Kruskal-Wallis Test
  - Continuous Field Information V1\_V2\_Diff
  - Categorical Field Information Srabismus
- Log

  - Log
- Nonparametric Tests

  - Hypothesis Test Summary
  - Independent-Samples Mann-Whitney U Test

    - V1\_V2\_Diff across RE\_Myopia

      - Independent-Samples Mann-Whitney U Test Summary
      - Independent-Samples Mann-Whitney U Test
  - Continuous Field Information V1\_V2\_Diff
  - Categorical Field Information RE\_Myopia
- Log

  - Log
- Nonparametric Tests

  - Hypothesis Test Summary
  - Independent-Samples Mann-Whitney U Test

    - V1\_V2\_Diff across RE\_ref error

      - Independent-Samples Mann-Whitney U Test Summary
      - Independent-Samples Mann-Whitney U Test
  - Continuous Field Information V1\_V2\_Diff
  - Categorical Field Information RE\_ref error
- Log

  - Log
- Nonparametric Tests

  - Hypothesis Test Summary
  - Independent-Samples Mann-Whitney U Test

    - V1\_V2\_Diff across RE\_cyl

      - Independent-Samples Mann-Whitney U Test Summary
      - Independent-Samples Mann-Whitney U Test
  - Continuous Field Information V1\_V2\_Diff
  - Categorical Field Information RE\_cyl
- Log

  - Log
- Nonparametric Tests

  - Hypothesis Test Summary
  - Independent-Samples Mann-Whitney U Test

    - V1\_V2\_Diff across LE\_Myopia

      - Independent-Samples Mann-Whitney U Test Summary
      - Independent-Samples Mann-Whitney U Test
  - Continuous Field Information V1\_V2\_Diff
  - Categorical Field Information LE\_Myopia
- Log

  - Log
- Nonparametric Tests

  - Hypothesis Test Summary
  - Independent-Samples Kruskal-Wallis Test

    - V1\_V2\_Diff across LE\_ref error

      - Independent-Samples Kruskal-Wallis Test Summary
      - Independent-Samples Kruskal-Wallis Test
  - Continuous Field Information V1\_V2\_Diff
  - Categorical Field Information LE\_ref error
- Log

  - Log
- Nonparametric Tests

  - Hypothesis Test Summary
  - Independent-Samples Mann-Whitney U Test

    - V1\_V2\_Diff across LE\_cyl

      - Independent-Samples Mann-Whitney U Test Summary
      - Independent-Samples Mann-Whitney U Test
  - Continuous Field Information V1\_V2\_Diff
  - Categorical Field Information LE\_cyl
- Log

  - Log
- Frequencies

  - Statistics
- Log

  - Log
- Explore

  - Case Processing Summary
  - Tests of Normality
  - V3 LogMar

    - Normal Q-Q Plot
    - Detrended Normal Q-Q Plot
  - V4 LogMar

    - Normal Q-Q Plot
    - Detrended Normal Q-Q Plot
- Log

  - Log
- T-Test

  - Paired Samples Statistics
  - Paired Samples Correlations
  - Paired Samples Test

- Delete

Log  
Log - Log - May 11, 2022

\*Nonparametric Tests: Independent Samples.  
NPTESTS  
  /INDEPENDENT TEST (V1\_V2\_Diff) GROUP (nystagmus)  
  /MISSING SCOPE=ANALYSIS USERMISSING=EXCLUDE  
  /CRITERIA ALPHA=0.05  CILEVEL=95.

Nonparametric Tests  
Nonparametric Tests - Hypothesis Test Summary - May 11, 2022

Hypothesis Test SummaryHypothesis Test Summary, table, 1 levels of column headers and 1 levels of row headers, table with 5 columns and 5 rows

|  |  |  |  |  |
| --- | --- | --- | --- | --- |
|  | Null Hypothesis | Test | Sig. | Decision |
| 1 | The distribution of V1\_V2\_Diff is the same across categories of nystagmus. | Independent-Samples Mann-Whitney U Test | .906a | Retain the null hypothesis. |
|  |  |  |  |  |
| --- | --- | --- | --- | --- |
| Asymptotic significances are displayed. The significance level is .050. | | | | |
| a. Exact significance is displayed for this test. | | | | |
|  |  |  |  |  |

V1\_V2\_Diff across nystagmus  
V1\_V2\_Diff across nystagmus - Independent-Samples Mann-Whitney U Test Summary - May 11, 2022

Independent-Samples Mann-Whitney U Test SummaryIndependent-Samples Mann-Whitney U Test Summary, table, 0 levels of column headers and 1 levels of row headers, table with 2 columns and 9 rows

|  |  |
| --- | --- |
| Total N | 65 |
| Mann-Whitney U | 126.500 |
| Wilcoxon W | 2017.500 |
| Test Statistic | 126.500 |
| Standard Error | 35.199 |
| Standardized Test Statistic | .128 |
| Asymptotic Sig.(2-sided test) | .898 |
| Exact Sig.(2-sided test) | .906 |
|  |  |

V1\_V2\_Diff across nystagmus  
V1\_V2\_Diff across nystagmus - Independent-Samples Mann-Whitney U Test - May 11, 2022

Nonparametric Tests  
Nonparametric Tests - Continuous Field Information V1\_V2\_Diff - May 11, 2022

Nonparametric Tests  
Nonparametric Tests - Categorical Field Information nystagmus - May 11, 2022

Log  
Log - Log - May 11, 2022

\*Nonparametric Tests: Independent Samples.  
NPTESTS  
  /INDEPENDENT TEST (V1\_V2\_Diff) GROUP (Eyemusclesurgery)  
  /MISSING SCOPE=ANALYSIS USERMISSING=EXCLUDE  
  /CRITERIA ALPHA=0.05  CILEVEL=95.

Nonparametric Tests  
Nonparametric Tests - Hypothesis Test Summary - May 11, 2022

Hypothesis Test SummaryHypothesis Test Summary, table, 1 levels of column headers and 1 levels of row headers, table with 5 columns and 4 rows

|  |  |  |  |  |
| --- | --- | --- | --- | --- |
|  | Null Hypothesis | Test | Sig. | Decision |
| 1 | The distribution of V1\_V2\_Diff is the same across categories of Eye muscle surgery. | Independent-Samples Mann-Whitney U Test | .717 | Retain the null hypothesis. |
|  |  |  |  |  |
| --- | --- | --- | --- | --- |
| Asymptotic significances are displayed. The significance level is .050. | | | | |
|  |  |  |  |  |

V1\_V2\_Diff across Eye muscle surgery  
V1\_V2\_Diff across Eye muscle surgery - Independent-Samples Mann-Whitney U Test Summary - May 11, 202(more)2(less)

Independent-Samples Mann-Whitney U Test SummaryIndependent-Samples Mann-Whitney U Test Summary, table, 0 levels of column headers and 1 levels of row headers, table with 2 columns and 8 rows

|  |  |
| --- | --- |
| Total N | 65 |
| Mann-Whitney U | 553.500 |
| Wilcoxon W | 1148.500 |
| Test Statistic | 553.500 |
| Standard Error | 73.156 |
| Standardized Test Statistic | .362 |
| Asymptotic Sig.(2-sided test) | .717 |
|  |  |

V1\_V2\_Diff across Eye muscle surgery  
V1\_V2\_Diff across Eye muscle surgery - Independent-Samples Mann-Whitney U Test - May 11, 2022

Nonparametric Tests  
Nonparametric Tests - Continuous Field Information V1\_V2\_Diff - May 11, 2022

Nonparametric Tests  
Nonparametric Tests - Categorical Field Information Eye muscle surgery - May 11, 2022

Log  
Log - Log - May 11, 2022

\*Nonparametric Tests: Independent Samples.  
NPTESTS  
  /INDEPENDENT TEST (V1\_V2\_Diff) GROUP (Srabismus)  
  /MISSING SCOPE=ANALYSIS USERMISSING=EXCLUDE  
  /CRITERIA ALPHA=0.05  CILEVEL=95.

Nonparametric Tests  
Nonparametric Tests - Hypothesis Test Summary - May 11, 2022

Hypothesis Test SummaryHypothesis Test Summary, table, 1 levels of column headers and 1 levels of row headers, table with 5 columns and 4 rows

|  |  |  |  |  |
| --- | --- | --- | --- | --- |
|  | Null Hypothesis | Test | Sig. | Decision |
| 1 | The distribution of V1\_V2\_Diff is the same across categories of Srabismus. | Independent-Samples Kruskal-Wallis Test | .441 | Retain the null hypothesis. |
|  |  |  |  |  |
| --- | --- | --- | --- | --- |
| Asymptotic significances are displayed. The significance level is .050. | | | | |
|  |  |  |  |  |

V1\_V2\_Diff across Srabismus  
V1\_V2\_Diff across Srabismus - Independent-Samples Kruskal-Wallis Test Summary - May 11, 2022

Independent-Samples Kruskal-Wallis Test SummaryIndependent-Samples Kruskal-Wallis Test Summary, table, 0 levels of column headers and 1 levels of row headers, table with 2 columns and 7 rows

|  |  |
| --- | --- |
| Total N | 65 |
| Test Statistic | 1.636a,b |
| Degree Of Freedom | 2 |
| Asymptotic Sig.(2-sided test) | .441 |
|  |  |
| --- | --- |
| a. The test statistic is adjusted for ties. | |
| b. Multiple comparisons are not performed because the overall test does not show significant differences across samples. | |
|  |  |

V1\_V2\_Diff across Srabismus  
V1\_V2\_Diff across Srabismus - Independent-Samples Kruskal-Wallis Test - May 11, 2022

Nonparametric Tests  
Nonparametric Tests - Continuous Field Information V1\_V2\_Diff - May 11, 2022

Nonparametric Tests  
Nonparametric Tests - Categorical Field Information Srabismus - May 11, 2022

Log  
Log - Log - May 11, 2022

\*Nonparametric Tests: Independent Samples.  
NPTESTS  
  /INDEPENDENT TEST (V1\_V2\_Diff) GROUP (RE\_Myopia)  
  /MISSING SCOPE=ANALYSIS USERMISSING=EXCLUDE  
  /CRITERIA ALPHA=0.05  CILEVEL=95.

Nonparametric Tests  
Nonparametric Tests - Hypothesis Test Summary - May 11, 2022

Hypothesis Test SummaryHypothesis Test Summary, table, 1 levels of column headers and 1 levels of row headers, table with 5 columns and 4 rows

|  |  |  |  |  |
| --- | --- | --- | --- | --- |
|  | Null Hypothesis | Test | Sig. | Decision |
| 1 | The distribution of V1\_V2\_Diff is the same across categories of RE\_Myopia. | Independent-Samples Mann-Whitney U Test | .672 | Retain the null hypothesis. |
|  |  |  |  |  |
| --- | --- | --- | --- | --- |
| Asymptotic significances are displayed. The significance level is .050. | | | | |
|  |  |  |  |  |

V1\_V2\_Diff across RE\_Myopia  
V1\_V2\_Diff across RE\_Myopia - Independent-Samples Mann-Whitney U Test Summary - May 11, 2022

Independent-Samples Mann-Whitney U Test SummaryIndependent-Samples Mann-Whitney U Test Summary, table, 0 levels of column headers and 1 levels of row headers, table with 2 columns and 8 rows

|  |  |
| --- | --- |
| Total N | 65 |
| Mann-Whitney U | 331.500 |
| Wilcoxon W | 436.500 |
| Test Statistic | 331.500 |
| Standard Error | 60.212 |
| Standardized Test Statistic | -.424 |
| Asymptotic Sig.(2-sided test) | .672 |
|  |  |

V1\_V2\_Diff across RE\_Myopia  
V1\_V2\_Diff across RE\_Myopia - Independent-Samples Mann-Whitney U Test - May 11, 2022

Nonparametric Tests  
Nonparametric Tests - Continuous Field Information V1\_V2\_Diff - May 11, 2022

Nonparametric Tests  
Nonparametric Tests - Categorical Field Information RE\_Myopia - May 11, 2022

Log  
Log - Log - May 11, 2022

\*Nonparametric Tests: Independent Samples.  
NPTESTS  
  /INDEPENDENT TEST (V1\_V2\_Diff) GROUP (RE\_referror)  
  /MISSING SCOPE=ANALYSIS USERMISSING=EXCLUDE  
  /CRITERIA ALPHA=0.05  CILEVEL=95.

Nonparametric Tests  
Nonparametric Tests - Hypothesis Test Summary - May 11, 2022

Hypothesis Test SummaryHypothesis Test Summary, table, 1 levels of column headers and 1 levels of row headers, table with 5 columns and 4 rows

|  |  |  |  |  |
| --- | --- | --- | --- | --- |
|  | Null Hypothesis | Test | Sig. | Decision |
| 1 | The distribution of V1\_V2\_Diff is the same across categories of RE\_ref error. | Independent-Samples Mann-Whitney U Test | .609 | Retain the null hypothesis. |
|  |  |  |  |  |
| --- | --- | --- | --- | --- |
| Asymptotic significances are displayed. The significance level is .050. | | | | |
|  |  |  |  |  |

V1\_V2\_Diff across RE\_ref error  
V1\_V2\_Diff across RE\_ref error - Independent-Samples Mann-Whitney U Test Summary - May 11, 2022

Independent-Samples Mann-Whitney U Test SummaryIndependent-Samples Mann-Whitney U Test Summary, table, 0 levels of column headers and 1 levels of row headers, table with 2 columns and 8 rows

|  |  |
| --- | --- |
| Total N | 65 |
| Mann-Whitney U | 456.500 |
| Wilcoxon W | 1584.500 |
| Test Statistic | 456.500 |
| Standard Error | 65.541 |
| Standardized Test Statistic | .511 |
| Asymptotic Sig.(2-sided test) | .609 |
|  |  |

V1\_V2\_Diff across RE\_ref error  
V1\_V2\_Diff across RE\_ref error - Independent-Samples Mann-Whitney U Test - May 11, 2022

Nonparametric Tests  
Nonparametric Tests - Continuous Field Information V1\_V2\_Diff - May 11, 2022

Nonparametric Tests  
Nonparametric Tests - Categorical Field Information RE\_ref error - May 11, 2022

Log  
Log - Log - May 11, 2022

\*Nonparametric Tests: Independent Samples.  
NPTESTS  
  /INDEPENDENT TEST (V1\_V2\_Diff) GROUP (RE\_cyl)  
  /MISSING SCOPE=ANALYSIS USERMISSING=EXCLUDE  
  /CRITERIA ALPHA=0.05  CILEVEL=95.

Nonparametric Tests  
Nonparametric Tests - Hypothesis Test Summary - May 11, 2022

Hypothesis Test SummaryHypothesis Test Summary, table, 1 levels of column headers and 1 levels of row headers, table with 5 columns and 4 rows

|  |  |  |  |  |
| --- | --- | --- | --- | --- |
|  | Null Hypothesis | Test | Sig. | Decision |
| 1 | The distribution of V1\_V2\_Diff is the same across categories of RE\_cyl. | Independent-Samples Mann-Whitney U Test | .322 | Retain the null hypothesis. |
|  |  |  |  |  |
| --- | --- | --- | --- | --- |
| Asymptotic significances are displayed. The significance level is .050. | | | | |
|  |  |  |  |  |

V1\_V2\_Diff across RE\_cyl  
V1\_V2\_Diff across RE\_cyl - Independent-Samples Mann-Whitney U Test Summary - May 11, 2022

Independent-Samples Mann-Whitney U Test SummaryIndependent-Samples Mann-Whitney U Test Summary, table, 0 levels of column headers and 1 levels of row headers, table with 2 columns and 8 rows

|  |  |
| --- | --- |
| Total N | 65 |
| Mann-Whitney U | 396.000 |
| Wilcoxon W | 1774.000 |
| Test Statistic | 396.000 |
| Standard Error | 58.587 |
| Standardized Test Statistic | .990 |
| Asymptotic Sig.(2-sided test) | .322 |
|  |  |

V1\_V2\_Diff across RE\_cyl  
V1\_V2\_Diff across RE\_cyl - Independent-Samples Mann-Whitney U Test - May 11, 2022

Nonparametric Tests  
Nonparametric Tests - Continuous Field Information V1\_V2\_Diff - May 11, 2022

Nonparametric Tests  
Nonparametric Tests - Categorical Field Information RE\_cyl - May 11, 2022

Log  
Log - Log - May 11, 2022

\*Nonparametric Tests: Independent Samples.  
NPTESTS  
  /INDEPENDENT TEST (V1\_V2\_Diff) GROUP (LE\_Myopia)  
  /MISSING SCOPE=ANALYSIS USERMISSING=EXCLUDE  
  /CRITERIA ALPHA=0.05  CILEVEL=95.

Nonparametric Tests  
Nonparametric Tests - Hypothesis Test Summary - May 11, 2022

Hypothesis Test SummaryHypothesis Test Summary, table, 1 levels of column headers and 1 levels of row headers, table with 5 columns and 4 rows

|  |  |  |  |  |
| --- | --- | --- | --- | --- |
|  | Null Hypothesis | Test | Sig. | Decision |
| 1 | The distribution of V1\_V2\_Diff is the same across categories of LE\_Myopia. | Independent-Samples Mann-Whitney U Test | .600 | Retain the null hypothesis. |
|  |  |  |  |  |
| --- | --- | --- | --- | --- |
| Asymptotic significances are displayed. The significance level is .050. | | | | |
|  |  |  |  |  |

V1\_V2\_Diff across LE\_Myopia  
V1\_V2\_Diff across LE\_Myopia - Independent-Samples Mann-Whitney U Test Summary - May 11, 2022

Independent-Samples Mann-Whitney U Test SummaryIndependent-Samples Mann-Whitney U Test Summary, table, 0 levels of column headers and 1 levels of row headers, table with 2 columns and 8 rows

|  |  |
| --- | --- |
| Total N | 64 |
| Mann-Whitney U | 319.000 |
| Wilcoxon W | 424.000 |
| Test Statistic | 319.000 |
| Standard Error | 59.055 |
| Standardized Test Statistic | -.525 |
| Asymptotic Sig.(2-sided test) | .600 |
|  |  |

V1\_V2\_Diff across LE\_Myopia  
V1\_V2\_Diff across LE\_Myopia - Independent-Samples Mann-Whitney U Test - May 11, 2022

Nonparametric Tests  
Nonparametric Tests - Continuous Field Information V1\_V2\_Diff - May 11, 2022

Nonparametric Tests  
Nonparametric Tests - Categorical Field Information LE\_Myopia - May 11, 2022

Log  
Log - Log - May 11, 2022

\*Nonparametric Tests: Independent Samples.  
NPTESTS  
  /INDEPENDENT TEST (V1\_V2\_Diff) GROUP (LE\_referror)  
  /MISSING SCOPE=ANALYSIS USERMISSING=EXCLUDE  
  /CRITERIA ALPHA=0.05  CILEVEL=95.

Nonparametric Tests  
Nonparametric Tests - Hypothesis Test Summary - May 11, 2022

Hypothesis Test SummaryHypothesis Test Summary, table, 1 levels of column headers and 1 levels of row headers, table with 5 columns and 4 rows

|  |  |  |  |  |
| --- | --- | --- | --- | --- |
|  | Null Hypothesis | Test | Sig. | Decision |
| 1 | The distribution of V1\_V2\_Diff is the same across categories of LE\_ref error. | Independent-Samples Kruskal-Wallis Test | .629 | Retain the null hypothesis. |
|  |  |  |  |  |
| --- | --- | --- | --- | --- |
| Asymptotic significances are displayed. The significance level is .050. | | | | |
|  |  |  |  |  |

V1\_V2\_Diff across LE\_ref error  
V1\_V2\_Diff across LE\_ref error - Independent-Samples Kruskal-Wallis Test Summary - May 11, 2022

Independent-Samples Kruskal-Wallis Test SummaryIndependent-Samples Kruskal-Wallis Test Summary, table, 0 levels of column headers and 1 levels of row headers, table with 2 columns and 7 rows

|  |  |
| --- | --- |
| Total N | 64 |
| Test Statistic | .233a,b |
| Degree Of Freedom | 1 |
| Asymptotic Sig.(2-sided test) | .629 |
|  |  |
| --- | --- |
| a. The test statistic is adjusted for ties. | |
| b. Multiple comparisons are not performed because the overall test does not show significant differences across samples. | |
|  |  |

V1\_V2\_Diff across LE\_ref error  
V1\_V2\_Diff across LE\_ref error - Independent-Samples Kruskal-Wallis Test - May 11, 2022

Nonparametric Tests  
Nonparametric Tests - Continuous Field Information V1\_V2\_Diff - May 11, 2022

Nonparametric Tests  
Nonparametric Tests - Categorical Field Information LE\_ref error - May 11, 2022

Log  
Log - Log - May 11, 2022

\*Nonparametric Tests: Independent Samples.  
NPTESTS  
  /INDEPENDENT TEST (V1\_V2\_Diff) GROUP (LE\_cyl)  
  /MISSING SCOPE=ANALYSIS USERMISSING=EXCLUDE  
  /CRITERIA ALPHA=0.05  CILEVEL=95.

Nonparametric Tests  
Nonparametric Tests - Hypothesis Test Summary - May 11, 2022

Hypothesis Test SummaryHypothesis Test Summary, table, 1 levels of column headers and 1 levels of row headers, table with 5 columns and 4 rows

|  |  |  |  |  |
| --- | --- | --- | --- | --- |
|  | Null Hypothesis | Test | Sig. | Decision |
| 1 | The distribution of V1\_V2\_Diff is the same across categories of LE\_cyl. | Independent-Samples Mann-Whitney U Test | .488 | Retain the null hypothesis. |
|  |  |  |  |  |
| --- | --- | --- | --- | --- |
| Asymptotic significances are displayed. The significance level is .050. | | | | |
|  |  |  |  |  |

V1\_V2\_Diff across LE\_cyl  
V1\_V2\_Diff across LE\_cyl - Independent-Samples Mann-Whitney U Test Summary - May 11, 2022

Independent-Samples Mann-Whitney U Test SummaryIndependent-Samples Mann-Whitney U Test Summary, table, 0 levels of column headers and 1 levels of row headers, table with 2 columns and 8 rows

|  |  |
| --- | --- |
| Total N | 64 |
| Mann-Whitney U | 391.000 |
| Wilcoxon W | 1666.000 |
| Test Statistic | 391.000 |
| Standard Error | 59.055 |
| Standardized Test Statistic | .694 |
| Asymptotic Sig.(2-sided test) | .488 |
|  |  |

V1\_V2\_Diff across LE\_cyl  
V1\_V2\_Diff across LE\_cyl - Independent-Samples Mann-Whitney U Test - May 11, 2022

Nonparametric Tests  
Nonparametric Tests - Continuous Field Information V1\_V2\_Diff - May 11, 2022

Nonparametric Tests  
Nonparametric Tests - Categorical Field Information LE\_cyl - May 11, 2022

Log  
Log - Log - May 11, 2022

DATASET ACTIVATE DataSet1.  
  
SAVE OUTFILE='D:\liavofra\עבודה\לקוחות\Hadassah\Claudia\Albinism\WF2\_new.sav'  
  /COMPRESSED.  
USE ALL.  
COMPUTE filter\_$=(AlbinismType=2  & MISSING(V4LogMar) =0).  
VARIABLE LABELS filter\_$ 'AlbinismType=2  & MISSING(V4LogMar) =0 (FILTER)'.  
VALUE LABELS filter\_$ 0 'Not Selected' 1 'Selected'.  
FORMATS filter\_$ (f1.0).  
FILTER BY filter\_$.  
EXECUTE.  
FREQUENCIES VARIABLES=V3LogMar V4LogMar  
  /STATISTICS=MEAN  
  /ORDER=ANALYSIS.

Frequencies  
Frequencies - Statistics - May 11, 2022

StatisticsStatistics, table, 1 levels of column headers and 2 levels of row headers, table with 4 columns and 5 rows

|  |  |  |  |
| --- | --- | --- | --- |
|  | | V3 LogMar | V4 LogMar |
| N | Valid | 5 | 5 |
|  | Missing | 0 | 0 |
| Mean | | .6400 | .6000 |
|  |  |  |  |

Log  
Log - Log - May 11, 2022

EXAMINE VARIABLES=V3LogMar V4LogMar  
  /PLOT NPPLOT  
  /STATISTICS NONE  
  /CINTERVAL 95  
  /MISSING LISTWISE  
  /NOTOTAL.

Explore  
Explore - Case Processing Summary - May 11, 2022

Case Processing SummaryCase Processing Summary, table, 3 levels of column headers and 1 levels of row headers, table with 7 columns and 6 rows

|  |  |  |  |  |  |  |
| --- | --- | --- | --- | --- | --- | --- |
|  | Cases | | | | | |
|  | Valid | | Missing | | Total | |
|  | N | Percent | N | Percent | N | Percent |
| V3 LogMar | 5 | 100.0% | 0 | 0.0% | 5 | 100.0% |
| V4 LogMar | 5 | 100.0% | 0 | 0.0% | 5 | 100.0% |
|  |  |  |  |  |  |  |

Explore  
Explore - Tests of Normality - May 11, 2022

Tests of NormalityTests of Normality, table, 2 levels of column headers and 1 levels of row headers, table with 7 columns and 7 rows

|  |  |  |  |  |  |  |
| --- | --- | --- | --- | --- | --- | --- |
|  | Kolmogorov-Smirnova | | | Shapiro-Wilk | | |
|  | Statistic | df | Sig. | Statistic | df | Sig. |
| V3 LogMar | .231 | 5 | .200\* | .881 | 5 | .314 |
| V4 LogMar | .300 | 5 | .161 | .833 | 5 | .146 |
|  |  |  |  |  |  |  |
| --- | --- | --- | --- | --- | --- | --- |
| \*. This is a lower bound of the true significance. | | | | | | |
| a. Lilliefors Significance Correction | | | | | | |
|  |  |  |  |  |  |  |

V3 LogMar  
V3 LogMar - Normal Q-Q Plot - May 11, 2022

V3 LogMar  
V3 LogMar - Detrended Normal Q-Q Plot - May 11, 2022

{"copyright":"(C) Copyright IBM Corp. 2011","grammar":[{"elements":[{"data":{"$ref":"dSource"},"style":{"symbol":"circle","outline":{"r":0,"b":157,"g":100},"size":6.6666665,"fill":{"r":119,"b":119,"g":118}},"position":[{"field":{"$ref":"fVariable1"}},{"field":{"$ref":"fVariable"}}],"type":"point"},{"data":{"$ref":"dSource"},"style":{"fill":{"r":0,"b":157,"g":100},"stroke":{"width":3.3333333}},"position":[{"value":"0"},{"field":{"$ref":"fVariable"}}],"type":"line"}],"coordinates":{"style":{"outline":{"r":0,"b":157,"g":100},"fill":{"r":255,"b":255,"g":255}},"dimensions":[{"scale":{"padding":{"left":"5%","right":"5%"}},"axis":[{"tickStyle":{"padding":5.0,"fill":{"r":0,"b":157,"g":100},"font":{"size":"8pt","weight":"normal","family":"sans-serif"}},"gridStyle":{"fill":{"r":0,"b":157,"g":100}},"lineStyle":{"fill":{"r":0,"b":157,"g":100},"stroke":{"width":0.6666667}},"titleStyle":{"padding":6.0,"fill":{"r":0,"b":157,"g":100},"font":{"size":"12pt","weight":"bold","family":"sans-serif"}},"title":["Dev from Normal"],"markStyle":{"fill":{"a":0,"r":0,"b":157,"g":100},"stroke":{"width":1.3333334}}}]},{"scale":{"padding":{"left":"5%","right":"5%"}},"axis":[{"tickStyle":{"padding":5.0,"fill":{"r":0,"b":157,"g":100},"font":{"size":"8pt","weight":"normal","family":"sans-serif"}},"gridStyle":{"fill":{"r":0,"b":157,"g":100}},"lineStyle":{"fill":{"r":0,"b":157,"g":100},"stroke":{"width":0.6666667}},"titleStyle":{"padding":6.0,"fill":{"r":0,"b":157,"g":100},"font":{"size":"12pt","weight":"bold","family":"sans-serif"}},"title":["Observed Value"],"markStyle":{"fill":{"a":0,"r":0,"b":157,"g":100},"stroke":{"width":1.3333334}}}]}]}}],"data":[{"id":"dSource","fields":[{"min":0.4,"max":0.8,"id":"fVariable","label":"X Variable\_1"},{"min":-0.4668527650995706,"max":0.2816931372714333,"id":"fVariable1","label":"Y Axis"},{"min":51.0,"max":75.0,"id":"fVariable2","label":"Case Number"}],"rows":[[0.4,-0.4668527650995706,72],[0.6,-0.02861732761895419,75],[0.6,-0.02861732761895419,51],[0.8,0.2816931372714333,61],[0.8,0.2816931372714333,58]]}],"size":{"width":850.0,"height":500.0},"style":{"outline":{"a":0.0,"r":0,"b":0,"g":0},"fill":{"r":255,"b":255,"g":255}},"titles":[{"backgroundStyle":{"outline":{"a":0.0,"r":0,"b":0,"g":0},"fill":{"a":0.0,"r":0,"b":0,"g":0}},"style":{"padding":3.0,"fill":{"r":0,"b":157,"g":100},"font":{"size":"12pt","weight":"bold","family":"sans-serif"}},"type":"title","content":["Detrended Normal Q-Q Plot of V3 LogMar"]}],"version":"6.0"}

V4 LogMar  
V4 LogMar - Normal Q-Q Plot - May 11, 2022

V4 LogMar  
V4 LogMar - Detrended Normal Q-Q Plot - May 11, 2022

{"copyright":"(C) Copyright IBM Corp. 2011","grammar":[{"elements":[{"data":{"$ref":"dSource"},"style":{"symbol":"circle","outline":{"r":0,"b":157,"g":100},"size":6.6666665,"fill":{"r":119,"b":119,"g":118}},"position":[{"field":{"$ref":"fVariable1"}},{"field":{"$ref":"fVariable"}}],"type":"point"},{"data":{"$ref":"dSource"},"style":{"fill":{"r":0,"b":157,"g":100},"stroke":{"width":3.3333333}},"position":[{"value":"0"},{"field":{"$ref":"fVariable"}}],"type":"line"}],"coordinates":{"style":{"outline":{"r":0,"b":157,"g":100},"fill":{"r":255,"b":255,"g":255}},"dimensions":[{"scale":{"padding":{"left":"5%","right":"5%"}},"axis":[{"tickStyle":{"padding":5.0,"fill":{"r":0,"b":157,"g":100},"font":{"size":"8pt","weight":"normal","family":"sans-serif"}},"gridStyle":{"fill":{"r":0,"b":157,"g":100}},"lineStyle":{"fill":{"r":0,"b":157,"g":100},"stroke":{"width":0.6666667}},"titleStyle":{"padding":6.0,"fill":{"r":0,"b":157,"g":100},"font":{"size":"12pt","weight":"bold","family":"sans-serif"}},"title":["Dev from Normal"],"markStyle":{"fill":{"a":0,"r":0,"b":157,"g":100},"stroke":{"width":1.3333334}}}]},{"scale":{"padding":{"left":"5%","right":"5%"}},"axis":[{"tickStyle":{"padding":5.0,"fill":{"r":0,"b":157,"g":100},"font":{"size":"8pt","weight":"normal","family":"sans-serif"}},"gridStyle":{"fill":{"r":0,"b":157,"g":100}},"lineStyle":{"fill":{"r":0,"b":157,"g":100},"stroke":{"width":0.6666667}},"titleStyle":{"padding":6.0,"fill":{"r":0,"b":157,"g":100},"font":{"size":"12pt","weight":"bold","family":"sans-serif"}},"title":["Observed Value"],"markStyle":{"fill":{"a":0,"r":0,"b":157,"g":100},"stroke":{"width":1.3333334}}}]}]}}],"data":[{"id":"dSource","fields":[{"min":0.4,"max":0.7,"id":"fVariable","label":"X Variable\_1"},{"min":-0.6655715957537507,"max":0.2104283942479247,"id":"fVariable1","label":"Y Axis"},{"min":51.0,"max":75.0,"id":"fVariable2","label":"Case Number"}],"rows":[[0.4,-0.6655715957537507,72],[0.6,0.2104283942479247,75],[0.6,0.2104283942479247,51],[0.7,0.1420068307316443,61],[0.7,0.1420068307316443,58]]}],"size":{"width":850.0,"height":500.0},"style":{"outline":{"a":0.0,"r":0,"b":0,"g":0},"fill":{"r":255,"b":255,"g":255}},"titles":[{"backgroundStyle":{"outline":{"a":0.0,"r":0,"b":0,"g":0},"fill":{"a":0.0,"r":0,"b":0,"g":0}},"style":{"padding":3.0,"fill":{"r":0,"b":157,"g":100},"font":{"size":"12pt","weight":"bold","family":"sans-serif"}},"type":"title","content":["Detrended Normal Q-Q Plot of V4 LogMar"]}],"version":"6.0"}

Log  
Log - Log - May 11, 2022

T-TEST PAIRS=V3LogMar WITH V4LogMar (PAIRED)  
  /CRITERIA=CI(.9500)  
  /MISSING=ANALYSIS.

T-Test  
T-Test - Paired Samples Statistics - May 11, 2022

Paired Samples StatisticsPaired Samples Statistics, table, 1 levels of column headers and 2 levels of row headers, table with 6 columns and 4 rows

|  |  |  |  |  |  |
| --- | --- | --- | --- | --- | --- |
|  | | Mean | N | Std. Deviation | Std. Error Mean |
| Pair 1 | V3 LogMar | .6400 | 5 | .16733 | .07483 |
|  | V4 LogMar | .6000 | 5 | .12247 | .05477 |
|  |  |  |  |  |  |

T-Test  
T-Test - Paired Samples Correlations - May 11, 2022

Paired Samples CorrelationsPaired Samples Correlations, table, 1 levels of column headers and 2 levels of row headers, table with 5 columns and 3 rows

|  |  |  |  |  |
| --- | --- | --- | --- | --- |
|  | | N | Correlation | Sig. |
| Pair 1 | V3 LogMar & V4 LogMar | 5 | .976 | .004 |
|  |  |  |  |  |

T-Test  
T-Test - Paired Samples Test - May 11, 2022

Paired Samples TestPaired Samples Test, table, 3 levels of column headers and 2 levels of row headers, table with 10 columns and 5 rows

|  |  |  |  |  |  |  |  |  |  |
| --- | --- | --- | --- | --- | --- | --- | --- | --- | --- |
|  | | Paired Differences | | | | | t | df | Sig. (2-tailed) |
|  |  | Mean | Std. Deviation | Std. Error Mean | 95% Confidence Interval of the Difference | |  |  |  |
|  |  |  |  |  | Lower | Upper |  |  |  |
| Pair 1 | V3 LogMar - V4 LogMar | .04000 | .05477 | .02449 | -.02801 | .10801 | 1.633 | 4 | .178 |
|  |  |  |  |  |  |  |  |  |  |

IBM SPSS Web Report

X

ABOUT

|  |
| --- |
| Created Using: IBM SPSS Statistics 26 |
| Creation Date: May 11, 2022 |
| Document Version: OriginalSaved Copy |
| Saved Date:  May 11, 2022 |

Navigation Controls

|  |
| --- |
| Contents - Opens and closes the list of charts and tables in the Web Report |
| Next & Previous - Display the next or previous table or chart in the Web Report |
| Help - Opens Help |

Toolbar Buttons

|  |  |
| --- | --- |
|  | Undo - Undoes the last change in the document. |
|  | Edit - Open the Editor tool for tables and charts. Certain editing options are only available when you are connected to an Internet server. |
|  | Save - Creates a new copy of the Web Report with the saved changes. |
|  | Print - Prints the current object when in Object View and all objects in Page View. |
|  | Page View - Switches the Web Report to display all the tables and charts on a single page. |
|  | Object View - Switches the Web Report so that each table or chart is displayed one at a time. |

Connecting to a Server

:   The status of the Web Report's connection to an Internet server appears in the top right corner of the Web Report.
:   An Internet connection is not required to open a Web Report. With a saved copy of the Web Report you can view all of the charts and tables, and have some limited editing ability, when not connected to the Internet.
:   Connecting a Web Report to an Internet server will enable far greater editing capabilities for tables and for charts.

- If the author specified an Internet server when they created the Web Report, the Web Report will attempt to connect to the server automatically when it is opened.
- If the Web Report does not connect to a server, click on the server Status Message to open tools to retry the connection, try a different server, or enter a new server address.
- For information about adding the enhanced controls to your Internet Server, go to https://developer.ibm.com/predictiveanalytics.
- If you specify a new server connection, the preferred format is http://xxx.xxx.xxx.xxx:xxxx.

Editing Tables

|  |  |
| --- | --- |
| Some of this functionality is only available when connected to an Internet server. | |
|  | Create a chart - Create a chart from the selected cells in the table. |
|  | Pivot and Sort - Transpose, sort, and pivot the table. |
|  | Background color - The background color of the selected cells. |
|  | Text Color and Style - Font color, style, and size. |
|  | Number Format - Font color, style, and size. |

Editing Charts

|  |  |
| --- | --- |
| All of this functionality is only available when connected to an Internet server. | |
|  | Chart Size - Change the height and width of the chart |
|  | Background color - The background color of the selected object. |
|  | Border and Line Style - The color and thickness of the line or border. |
|  | Text Color and Style - Font color, style, and size. |
|  | Number Format - Font color, style, and size. |
|  | Axis Properties - Change the scale and display axis titles and ticks. |

Save

X
New Name  
   
  
What to Save   

Save the entire document  
Only save the current object

Server Connection

X
  
Saved Server Connections  
    http://127.0.0.1:9080/webreport/   
  
  
  
Status  
Cannot connect to the specified server.

Add a chart

Pivot and Sort

Chart Size   
  

|  |  |  |
| --- | --- | --- |
|  |  |  |
|  |  |  |
| Lock aspect ratio | | |

Background   

|  |  |  |  |  |  |
| --- | --- | --- | --- | --- | --- |
|  | |  | |  | |
|  |  |  |  |  |  |
|  |  |  |  |  |  |
|  |  |  |  |  |  |

Line and Borders   

|  |  |  |  |  |  |
| --- | --- | --- | --- | --- | --- |
|  | |  | |  | |
|  |  |  |  |  |  |
|  |  |  |  |  |  |
|  |  |  |  |  |  |

  

|  |  |  |
| --- | --- | --- |
|  |  |  |

Text Format   

|  |  |  |  |  |  |
| --- | --- | --- | --- | --- | --- |
|  | |  | |  | |
|  |  |  |  |  |  |
|  |  |  |  |  |  |
|  |  |  |  |  |  |

  

|  |  |  |
| --- | --- | --- |
|  |  |  |

  

|  |  |  |  |
| --- | --- | --- | --- |
|  |  |  | Font Family  Agency FB Aharoni Algerian Arial Arial Black Arial Narrow Arial Rounded MT Bold Bahnschrift Baskerville Old Face Bauhaus 93 Bell MT Berlin Sans FB Berlin Sans FB Demi Bernard MT Condensed Blackadder ITC Bodoni MT Bodoni MT Black Bodoni MT Condensed Bodoni MT Poster Compressed Book Antiqua Bookman Old Style Bookshelf Symbol 7 Bradley Hand ITC Britannic Bold Broadway Brush Script MT Calibri Calibri Light Californian FB Calisto MT Cambria Cambria Math Candara Candara Light Castellar Centaur Century Century Gothic Century Schoolbook Chiller Colonna MT Comic Sans MS Consolas Constantia Cooper Black Copperplate Gothic Bold Copperplate Gothic Light Corbel Corbel Light Courier New Curlz MT David Dialog DialogInput Dubai Dubai Light Dubai Medium Ebrima Edwardian Script ITC Elephant Engravers MT Eras Bold ITC Eras Demi ITC Eras Light ITC Eras Medium ITC Felix Titling Footlight MT Light Forte Franklin Gothic Book Franklin Gothic Demi Franklin Gothic Demi Cond Franklin Gothic Heavy Franklin Gothic Medium Franklin Gothic Medium Cond FrankRuehl Freestyle Script French Script MT Gabriola Gadugi Garamond Georgia Gigi Gill Sans MT Gill Sans MT Condensed Gill Sans MT Ext Condensed Bold Gill Sans Ultra Bold Gill Sans Ultra Bold Condensed Gisha Gloucester MT Extra Condensed Goudy Old Style Goudy Stout Guttman Aharoni Guttman Drogolin Guttman Frank Guttman Frnew Guttman Haim Guttman Haim-Condensed Guttman Hatzvi Guttman Kav Guttman Kav-Light Guttman Logo1 Guttman Mantova Guttman Mantova-Decor Guttman Miryam Guttman Myamfix Guttman Rashi Guttman Stam Guttman Stam1 Guttman Vilna Guttman Yad Guttman Yad-Brush Guttman Yad-Light Guttman-Aharoni Guttman-Aram Guttman-CourMir Hadassah Friedlaender Haettenschweiler Harlow Solid Italic Harrington HelvNeue Roman for IBM High Tower Text HoloLens MDL2 Assets Impact Imprint MT Shadow Informal Roman Ink Free Javanese Text Jokerman Juice ITC Kristen ITC Kunstler Script Leelawadee Leelawadee UI Leelawadee UI Semilight Levenim MT Lucida Bright Lucida Calligraphy Lucida Console Lucida Fax Lucida Handwriting Lucida Sans Lucida Sans Typewriter Lucida Sans Unicode Magneto Maiandra GD Malgun Gothic Malgun Gothic Semilight Marlett Matura MT Script Capitals Microsoft Himalaya Microsoft JhengHei Microsoft JhengHei Light Microsoft JhengHei UI Microsoft JhengHei UI Light Microsoft New Tai Lue Microsoft PhagsPa Microsoft Sans Serif Microsoft Tai Le Microsoft Uighur Microsoft YaHei Microsoft YaHei Light Microsoft YaHei UI Microsoft YaHei UI Light Microsoft Yi Baiti MingLiU-ExtB MingLiU\_HKSCS-ExtB Miriam Miriam Fixed Mistral Modern No. 20 Mongolian Baiti Monospaced Monotype Corsiva MS Gothic MS Outlook MS PGothic MS Reference Sans Serif MS Reference Specialty MS UI Gothic MT Extra MV Boli Myanmar Text Narkisim Niagara Engraved Niagara Solid Nirmala UI Nirmala UI Semilight NSimSun OCR A Extended Old English Text MT Onyx Palace Script MT Palatino Linotype Papyrus Parchment Perpetua Perpetua Titling MT Playbill PMingLiU-ExtB Poor Richard Pristina Rage Italic Ravie Rockwell Rockwell Condensed Rockwell Extra Bold Rod SansSerif Script MT Bold Segoe MDL2 Assets Segoe Print Segoe Script Segoe UI Segoe UI Black Segoe UI Emoji Segoe UI Historic Segoe UI Light Segoe UI Semibold Segoe UI Semilight Segoe UI Symbol Serif Showcard Gothic SimSun SimSun-ExtB Sitka Banner Sitka Display Sitka Heading Sitka Small Sitka Subheading Sitka Text Snap ITC Stencil Sylfaen Symbol Tahoma TeamViewer15 Tempus Sans ITC Times New Roman Trebuchet MS Tw Cen MT Tw Cen MT Condensed Tw Cen MT Condensed Extra Bold Verdana Viner Hand ITC Vivaldi Vladimir Script Webdings Wide Latin Wingdings Wingdings 2 Wingdings 3 Yu Gothic Yu Gothic Light Yu Gothic Medium Yu Gothic UI Yu Gothic UI Light Yu Gothic UI Semibold Yu Gothic UI Semilight |

Number Format   
  

|  |  |  |
| --- | --- | --- |
| 0.00 |  |  |

Axis Options   
  

|  |  |  |
| --- | --- | --- |
|  |  |  |
|  |  |  |
| Display Axis Title | | | |
| Display Ticks | | | |
