# Supplementary material for: Visual acuity improvement in children with albinism beyond the first decade of life
Source: PLoS One. 2024 Jan 17;19(1):e0296744. doi: 10.1371/journal.pone.0296744 (PMC10793880; doi:10.1371/journal.pone.0296744)
Supplement: S2 Data — (HTML) [file pone.0296744.s003.html]

Inferential Statistics\_new


|  |  |  |
| --- | --- | --- |
| IBM SPSS Web Report - Inferential Statistics\_new.spv     ---   Contents  Previous  Next  Help |  | Not connected to the server     --- |

- Log

  - Log
- Explore

  - Case Processing Summary
  - Tests of Normality
  - V1 LogMar

    - Normal Q-Q Plots

      - For AlbinismType = OCA1A
      - For AlbinismType = OCA1B
      - For AlbinismType = OCA2
    - Detrended Normal Q-Q Plots

      - For AlbinismType = OCA1A
      - For AlbinismType = OCA1B
      - For AlbinismType = OCA2
  - V2 LogMar

    - Normal Q-Q Plots

      - For AlbinismType = OCA1A
      - For AlbinismType = OCA1B
      - For AlbinismType = OCA2
    - Detrended Normal Q-Q Plots

      - For AlbinismType = OCA1A
      - For AlbinismType = OCA1B
      - For AlbinismType = OCA2
  - V3 LogMar

    - Normal Q-Q Plots

      - For AlbinismType = OCA1A
      - For AlbinismType = OCA1B
      - For AlbinismType = OCA2
    - Detrended Normal Q-Q Plots

      - For AlbinismType = OCA1A
      - For AlbinismType = OCA1B
      - For AlbinismType = OCA2
  - V4 LogMar

    - Normal Q-Q Plots

      - For AlbinismType = OCA1A
      - For AlbinismType = OCA1B
      - For AlbinismType = OCA2
    - Detrended Normal Q-Q Plots

      - For AlbinismType = OCA1A
      - For AlbinismType = OCA1B
      - For AlbinismType = OCA2
- Log

  - Log
- T-Test

  - Paired Samples Statistics
  - Paired Samples Correlations
  - Paired Samples Test
- Log

  - Log
- Nonparametric Tests

  - Hypothesis Test Summary
  - Related-Samples Friedman's Two-Way Analysis of Var...

    - V1 LogMar, V2 LogMar, V3 LogMar, V4 LogMar

      - Related-Samples Friedman's Two-Way Analysis of Var...
      - Related-Samples Friedman's Two-Way Analysis of Var...
  - Continuous Field Information V1 LogMar; Albinism T...
  - Continuous Field Information V2 LogMar; Albinism T...
  - Continuous Field Information V3 LogMar; Albinism T...
  - Continuous Field Information V4 LogMar; Albinism T...
- Log

  - Log
- Crosstabs

  - Warnings
- Log

  - Log
- Crosstabs

  - Warnings
- Log

  - Log
- Crosstabs

  - Case Processing Summary
  - Albinism Type \* nystagmus

    - Crosstab
    - Chi-Square Tests
    - Bar Chart
  - Albinism Type \* Albinism

    - Crosstab
    - Chi-Square Tests
    - Bar Chart
  - Albinism Type \* Eye muscle surgery

    - Crosstab
    - Chi-Square Tests
    - Bar Chart
  - Albinism Type \* Srabismus

    - Crosstab
    - Chi-Square Tests
    - Bar Chart
  - Albinism Type \* RE\_Myopia

    - Crosstab
    - Chi-Square Tests
    - Bar Chart
  - Albinism Type \* RE\_ref error

    - Crosstab
    - Chi-Square Tests
    - Bar Chart
  - Albinism Type \* RE\_cyl

    - Crosstab
    - Chi-Square Tests
    - Bar Chart
  - Albinism Type \* LE\_Myopia

    - Crosstab
    - Chi-Square Tests
    - Bar Chart
  - Albinism Type \* LE\_ref error

    - Crosstab
    - Chi-Square Tests
    - Bar Chart
  - Albinism Type \* LE\_cyl

    - Crosstab
    - Chi-Square Tests
    - Bar Chart
- Log

  - Log
- Create

  - Created Series
- Log

  - Log
- Oneway

  - Descriptives
  - ANOVA
  - Post Hoc Tests

    - Multiple Comparisons
    - Homogeneous Subsets

      - V1 LogMar
      - V2 LogMar
      - V3 LogMar
      - V4 LogMar
  - Means Plots

    - V1 LogMar
    - V2 LogMar
    - V3 LogMar
    - V4 LogMar
- Log

  - Log
- General Linear Model

  - Within-Subjects Factors
  - Between-Subjects Factors
  - Multivariate Tests
  - Mauchly's Test of Sphericity
  - Tests of Within-Subjects Effects
  - Tests of Within-Subjects Contrasts
  - Tests of Between-Subjects Effects
- Log

  - Log
- General Linear Model

  - Within-Subjects Factors
  - Between-Subjects Factors
  - Multivariate Tests
  - Mauchly's Test of Sphericity
  - Tests of Within-Subjects Effects
  - Tests of Within-Subjects Contrasts
  - Tests of Between-Subjects Effects
- Log

  - Log
- General Linear Model

  - Within-Subjects Factors
  - Between-Subjects Factors
  - Multivariate Tests
  - Mauchly's Test of Sphericity
  - Tests of Within-Subjects Effects
  - Tests of Within-Subjects Contrasts
  - Tests of Between-Subjects Effects
- Log

  - Log
- Explore

  - Case Processing Summary
  - Tests of Normality
  - V1 LogMar

    - Normal Q-Q Plot
    - Detrended Normal Q-Q Plot
  - V2 LogMar

    - Normal Q-Q Plot
    - Detrended Normal Q-Q Plot
  - V3 LogMar

    - Normal Q-Q Plot
    - Detrended Normal Q-Q Plot
  - V4 LogMar

    - Normal Q-Q Plot
    - Detrended Normal Q-Q Plot

- Delete

Log  
Log - Log - May 11, 2022

EXAMINE VARIABLES=V1LogMar V2LogMar V3LogMar V4LogMar  
  /PLOT NPPLOT  
  /STATISTICS NONE  
  /CINTERVAL 95  
  /MISSING LISTWISE  
  /NOTOTAL.

Explore  
Explore - Case Processing Summary - May 11, 2022

Case Processing SummaryCase Processing Summary, table, 3 levels of column headers and 2 levels of row headers, table with 8 columns and 16 rows

|  |  |  |  |  |  |  |  |
| --- | --- | --- | --- | --- | --- | --- | --- |
| Albinism Type | | Cases | | | | | |
|  |  | Valid | | Missing | | Total | |
|  |  | N | Percent | N | Percent | N | Percent |
| OCA1A | V1 LogMar | 4 | 23.5% | 13 | 76.5% | 17 | 100.0% |
|  | V2 LogMar | 4 | 23.5% | 13 | 76.5% | 17 | 100.0% |
|  | V3 LogMar | 4 | 23.5% | 13 | 76.5% | 17 | 100.0% |
|  | V4 LogMar | 4 | 23.5% | 13 | 76.5% | 17 | 100.0% |
| OCA1B | V1 LogMar | 4 | 14.3% | 24 | 85.7% | 28 | 100.0% |
|  | V2 LogMar | 4 | 14.3% | 24 | 85.7% | 28 | 100.0% |
|  | V3 LogMar | 4 | 14.3% | 24 | 85.7% | 28 | 100.0% |
|  | V4 LogMar | 4 | 14.3% | 24 | 85.7% | 28 | 100.0% |
| OCA2 | V1 LogMar | 6 | 23.1% | 20 | 76.9% | 26 | 100.0% |
|  | V2 LogMar | 6 | 23.1% | 20 | 76.9% | 26 | 100.0% |
|  | V3 LogMar | 6 | 23.1% | 20 | 76.9% | 26 | 100.0% |
|  | V4 LogMar | 6 | 23.1% | 20 | 76.9% | 26 | 100.0% |
|  |  |  |  |  |  |  |  |

Explore  
Explore - Tests of Normality - May 11, 2022

Tests of NormalityTests of Normality, table, 2 levels of column headers and 2 levels of row headers, table with 8 columns and 17 rows

|  |  |  |  |  |  |  |  |
| --- | --- | --- | --- | --- | --- | --- | --- |
| Albinism Type | | Kolmogorov-Smirnova | | | Shapiro-Wilk | | |
|  |  | Statistic | df | Sig. | Statistic | df | Sig. |
| OCA1A | V1 LogMar | .260 | 4 | . | .827 | 4 | .161 |
|  | V2 LogMar | .303 | 4 | . | .791 | 4 | .086 |
|  | V3 LogMar | .250 | 4 | . | .927 | 4 | .577 |
|  | V4 LogMar | .389 | 4 | . | .786 | 4 | .079 |
| OCA1B | V1 LogMar | .441 | 4 | . | .630 | 4 | .001 |
|  | V2 LogMar | .192 | 4 | . | .971 | 4 | .850 |
|  | V3 LogMar | .307 | 4 | . | .729 | 4 | .024 |
|  | V4 LogMar | .307 | 4 | . | .729 | 4 | .024 |
| OCA2 | V1 LogMar | .187 | 6 | .200\* | .914 | 6 | .466 |
|  | V2 LogMar | .204 | 6 | .200\* | .902 | 6 | .389 |
|  | V3 LogMar | .199 | 6 | .200\* | .903 | 6 | .393 |
|  | V4 LogMar | .149 | 6 | .200\* | .977 | 6 | .938 |
|  |  |  |  |  |  |  |  |
| --- | --- | --- | --- | --- | --- | --- | --- |
| \*. This is a lower bound of the true significance. | | | | | | | |
| a. Lilliefors Significance Correction | | | | | | | |
|  |  |  |  |  |  |  |  |

Normal Q-Q Plots  
Normal Q-Q Plots - For AlbinismType = OCA1A - May 11, 2022

Normal Q-Q Plots  
Normal Q-Q Plots - For AlbinismType = OCA1B - May 11, 2022

Normal Q-Q Plots  
Normal Q-Q Plots - For AlbinismType = OCA2 - May 11, 2022

Detrended Normal Q-Q Plots  
Detrended Normal Q-Q Plots - For AlbinismType = OCA1A - May 11, 2022

{"copyright":"(C) Copyright IBM Corp. 2011","grammar":[{"elements":[{"data":{"$ref":"dSource"},"style":{"symbol":"circle","outline":{"r":0,"b":157,"g":100},"size":6.6666665,"fill":{"r":119,"b":119,"g":118}},"position":[{"field":{"$ref":"fVariable1"}},{"field":{"$ref":"fVariable"}}],"type":"point"},{"data":{"$ref":"dSource"},"style":{"fill":{"r":0,"b":157,"g":100},"stroke":{"width":3.3333333}},"position":[{"value":"0"},{"field":{"$ref":"fVariable"}}],"type":"line"}],"coordinates":{"style":{"outline":{"r":0,"b":157,"g":100},"fill":{"r":255,"b":255,"g":255}},"dimensions":[{"scale":{"padding":{"left":"5%","right":"5%"}},"axis":[{"tickStyle":{"padding":5.0,"fill":{"r":0,"b":157,"g":100},"font":{"size":"8pt","weight":"normal","family":"sans-serif"}},"gridStyle":{"fill":{"r":0,"b":157,"g":100}},"lineStyle":{"fill":{"r":0,"b":157,"g":100},"stroke":{"width":0.6666667}},"titleStyle":{"padding":6.0,"fill":{"r":0,"b":157,"g":100},"font":{"size":"12pt","weight":"bold","family":"sans-serif"}},"title":["Dev from Normal"],"markStyle":{"fill":{"a":0,"r":0,"b":157,"g":100},"stroke":{"width":1.3333334}}}]},{"scale":{"padding":{"left":"5%","right":"5%"}},"axis":[{"tickStyle":{"padding":5.0,"fill":{"r":0,"b":157,"g":100},"font":{"size":"8pt","weight":"normal","family":"sans-serif"}},"gridStyle":{"fill":{"r":0,"b":157,"g":100}},"lineStyle":{"fill":{"r":0,"b":157,"g":100},"stroke":{"width":0.6666667}},"titleStyle":{"padding":6.0,"fill":{"r":0,"b":157,"g":100},"font":{"size":"12pt","weight":"bold","family":"sans-serif"}},"title":["Observed Value"],"markStyle":{"fill":{"a":0,"r":0,"b":157,"g":100},"stroke":{"width":1.3333334}}}]}]}}],"data":[{"id":"dSource","fields":[{"min":0.6,"max":0.9,"id":"fVariable","label":"X Variable\_1"},{"min":-0.5725923288001812,"max":0.2533471031357998,"id":"fVariable1","label":"Y Axis"},{"min":39.0,"max":46.0,"id":"fVariable2","label":"Case Number"}],"rows":[[0.6,-0.5725923288001812,41],[0.8,0.2533471031357998,46],[0.9,0.1827062684785066,43],[0.9,0.1827062684785066,39]]}],"size":{"width":850.0,"height":500.0},"style":{"outline":{"a":0.0,"r":0,"b":0,"g":0},"fill":{"r":255,"b":255,"g":255}},"titles":[{"backgroundStyle":{"outline":{"a":0.0,"r":0,"b":0,"g":0},"fill":{"a":0.0,"r":0,"b":0,"g":0}},"style":{"padding":3.0,"fill":{"r":0,"b":157,"g":100},"font":{"size":"12pt","weight":"bold","family":"sans-serif"}},"type":"title","content":["Detrended Normal Q-Q Plot of V1 LogMar"]},{"backgroundStyle":{"outline":{"a":0.0,"r":0,"b":0,"g":0},"fill":{"a":0.0,"r":0,"b":0,"g":0}},"style":{"padding":3.0,"fill":{"r":0,"b":157,"g":100},"font":{"size":"10pt","weight":"bold","family":"sans-serif"}},"type":"title","content":["Albinism Type= OCA1A"]}],"version":"6.0"}

Detrended Normal Q-Q Plots  
Detrended Normal Q-Q Plots - For AlbinismType = OCA1B - May 11, 2022

{"copyright":"(C) Copyright IBM Corp. 2011","grammar":[{"elements":[{"data":{"$ref":"dSource"},"style":{"symbol":"circle","outline":{"r":0,"b":157,"g":100},"size":6.6666665,"fill":{"r":119,"b":119,"g":118}},"position":[{"field":{"$ref":"fVariable1"}},{"field":{"$ref":"fVariable"}}],"type":"point"},{"data":{"$ref":"dSource"},"style":{"fill":{"r":0,"b":157,"g":100},"stroke":{"width":3.3333333}},"position":[{"value":"0"},{"field":{"$ref":"fVariable"}}],"type":"line"}],"coordinates":{"style":{"outline":{"r":0,"b":157,"g":100},"fill":{"r":255,"b":255,"g":255}},"dimensions":[{"scale":{"padding":{"left":"5%","right":"5%"}},"axis":[{"tickStyle":{"padding":5.0,"fill":{"r":0,"b":157,"g":100},"font":{"size":"8pt","weight":"normal","family":"sans-serif"}},"gridStyle":{"fill":{"r":0,"b":157,"g":100}},"lineStyle":{"fill":{"r":0,"b":157,"g":100},"stroke":{"width":0.6666667}},"titleStyle":{"padding":6.0,"fill":{"r":0,"b":157,"g":100},"font":{"size":"12pt","weight":"bold","family":"sans-serif"}},"title":["Dev from Normal"],"markStyle":{"fill":{"a":0,"r":0,"b":157,"g":100},"stroke":{"width":1.3333334}}}]},{"scale":{"padding":{"left":"5%","right":"5%"}},"axis":[{"tickStyle":{"padding":5.0,"fill":{"r":0,"b":157,"g":100},"font":{"size":"8pt","weight":"normal","family":"sans-serif"}},"gridStyle":{"fill":{"r":0,"b":157,"g":100}},"lineStyle":{"fill":{"r":0,"b":157,"g":100},"stroke":{"width":0.6666667}},"titleStyle":{"padding":6.0,"fill":{"r":0,"b":157,"g":100},"font":{"size":"12pt","weight":"bold","family":"sans-serif"}},"title":["Observed Value"],"markStyle":{"fill":{"a":0,"r":0,"b":157,"g":100},"stroke":{"width":1.3333334}}}]}]}}],"data":[{"id":"dSource","fields":[{"min":0.7,"max":1.0,"id":"fVariable","label":"X Variable\_1"},{"min":-0.2466528968641998,"max":0.658378766427086,"id":"fVariable1","label":"Y Axis"},{"min":51.0,"max":75.0,"id":"fVariable2","label":"Case Number"}],"rows":[[0.7,-0.2466528968641998,75],[0.7,-0.2466528968641998,58],[0.7,-0.2466528968641998,51],[1,0.658378766427086,61]]}],"size":{"width":850.0,"height":500.0},"style":{"outline":{"a":0.0,"r":0,"b":0,"g":0},"fill":{"r":255,"b":255,"g":255}},"titles":[{"backgroundStyle":{"outline":{"a":0.0,"r":0,"b":0,"g":0},"fill":{"a":0.0,"r":0,"b":0,"g":0}},"style":{"padding":3.0,"fill":{"r":0,"b":157,"g":100},"font":{"size":"12pt","weight":"bold","family":"sans-serif"}},"type":"title","content":["Detrended Normal Q-Q Plot of V1 LogMar"]},{"backgroundStyle":{"outline":{"a":0.0,"r":0,"b":0,"g":0},"fill":{"a":0.0,"r":0,"b":0,"g":0}},"style":{"padding":3.0,"fill":{"r":0,"b":157,"g":100},"font":{"size":"10pt","weight":"bold","family":"sans-serif"}},"type":"title","content":["Albinism Type= OCA1B"]}],"version":"6.0"}

Detrended Normal Q-Q Plots  
Detrended Normal Q-Q Plots - For AlbinismType = OCA2 - May 11, 2022

{"copyright":"(C) Copyright IBM Corp. 2011","grammar":[{"elements":[{"data":{"$ref":"dSource"},"style":{"symbol":"circle","outline":{"r":0,"b":157,"g":100},"size":6.6666665,"fill":{"r":119,"b":119,"g":118}},"position":[{"field":{"$ref":"fVariable1"}},{"field":{"$ref":"fVariable"}}],"type":"point"},{"data":{"$ref":"dSource"},"style":{"fill":{"r":0,"b":157,"g":100},"stroke":{"width":3.3333333}},"position":[{"value":"0"},{"field":{"$ref":"fVariable"}}],"type":"line"}],"coordinates":{"style":{"outline":{"r":0,"b":157,"g":100},"fill":{"r":255,"b":255,"g":255}},"dimensions":[{"scale":{"padding":{"left":"5%","right":"5%"}},"axis":[{"tickStyle":{"padding":5.0,"fill":{"r":0,"b":157,"g":100},"font":{"size":"8pt","weight":"normal","family":"sans-serif"}},"gridStyle":{"fill":{"r":0,"b":157,"g":100}},"lineStyle":{"fill":{"r":0,"b":157,"g":100},"stroke":{"width":0.6666667}},"titleStyle":{"padding":6.0,"fill":{"r":0,"b":157,"g":100},"font":{"size":"12pt","weight":"bold","family":"sans-serif"}},"title":["Dev from Normal"],"markStyle":{"fill":{"a":0,"r":0,"b":157,"g":100},"stroke":{"width":1.3333334}}}]},{"scale":{"padding":{"left":"5%","right":"5%"}},"axis":[{"tickStyle":{"padding":5.0,"fill":{"r":0,"b":157,"g":100},"font":{"size":"8pt","weight":"normal","family":"sans-serif"}},"gridStyle":{"fill":{"r":0,"b":157,"g":100}},"lineStyle":{"fill":{"r":0,"b":157,"g":100},"stroke":{"width":0.6666667}},"titleStyle":{"padding":6.0,"fill":{"r":0,"b":157,"g":100},"font":{"size":"12pt","weight":"bold","family":"sans-serif"}},"title":["Observed Value"],"markStyle":{"fill":{"a":0,"r":0,"b":157,"g":100},"stroke":{"width":1.3333334}}}]}]}}],"data":[{"id":"dSource","fields":[{"min":0.5,"max":1.0,"id":"fVariable","label":"X Variable\_1"},{"min":-0.2624539456460857,"max":0.5135683062060479,"id":"fVariable1","label":"Y Axis"},{"min":89.0,"max":101.0,"id":"fVariable2","label":"Case Number"}],"rows":[[0.5,-0.2624539456460857,101],[0.5,-0.2624539456460857,95],[0.7,-5.851389114294503E-16,96],[0.7,-5.851389114294503E-16,89],[0.8,-0.0389025452381333,100],[1,0.5135683062060479,98]]}],"size":{"width":850.0,"height":500.0},"style":{"outline":{"a":0.0,"r":0,"b":0,"g":0},"fill":{"r":255,"b":255,"g":255}},"titles":[{"backgroundStyle":{"outline":{"a":0.0,"r":0,"b":0,"g":0},"fill":{"a":0.0,"r":0,"b":0,"g":0}},"style":{"padding":3.0,"fill":{"r":0,"b":157,"g":100},"font":{"size":"12pt","weight":"bold","family":"sans-serif"}},"type":"title","content":["Detrended Normal Q-Q Plot of V1 LogMar"]},{"backgroundStyle":{"outline":{"a":0.0,"r":0,"b":0,"g":0},"fill":{"a":0.0,"r":0,"b":0,"g":0}},"style":{"padding":3.0,"fill":{"r":0,"b":157,"g":100},"font":{"size":"10pt","weight":"bold","family":"sans-serif"}},"type":"title","content":["Albinism Type= OCA2"]}],"version":"6.0"}

Normal Q-Q Plots  
Normal Q-Q Plots - For AlbinismType = OCA1A - May 11, 2022

Normal Q-Q Plots  
Normal Q-Q Plots - For AlbinismType = OCA1B - May 11, 2022

Normal Q-Q Plots  
Normal Q-Q Plots - For AlbinismType = OCA2 - May 11, 2022

Detrended Normal Q-Q Plots  
Detrended Normal Q-Q Plots - For AlbinismType = OCA1A - May 11, 2022

{"copyright":"(C) Copyright IBM Corp. 2011","grammar":[{"elements":[{"data":{"$ref":"dSource"},"style":{"symbol":"circle","outline":{"r":0,"b":157,"g":100},"size":6.6666665,"fill":{"r":119,"b":119,"g":118}},"position":[{"field":{"$ref":"fVariable1"}},{"field":{"$ref":"fVariable"}}],"type":"point"},{"data":{"$ref":"dSource"},"style":{"fill":{"r":0,"b":157,"g":100},"stroke":{"width":3.3333333}},"position":[{"value":"0"},{"field":{"$ref":"fVariable"}}],"type":"line"}],"coordinates":{"style":{"outline":{"r":0,"b":157,"g":100},"fill":{"r":255,"b":255,"g":255}},"dimensions":[{"scale":{"padding":{"left":"5%","right":"5%"}},"axis":[{"tickStyle":{"padding":5.0,"fill":{"r":0,"b":157,"g":100},"font":{"size":"8pt","weight":"normal","family":"sans-serif"}},"gridStyle":{"fill":{"r":0,"b":157,"g":100}},"lineStyle":{"fill":{"r":0,"b":157,"g":100},"stroke":{"width":0.6666667}},"titleStyle":{"padding":6.0,"fill":{"r":0,"b":157,"g":100},"font":{"size":"12pt","weight":"bold","family":"sans-serif"}},"title":["Dev from Normal"],"markStyle":{"fill":{"a":0,"r":0,"b":157,"g":100},"stroke":{"width":1.3333334}}}]},{"scale":{"padding":{"left":"5%","right":"5%"}},"axis":[{"tickStyle":{"padding":5.0,"fill":{"r":0,"b":157,"g":100},"font":{"size":"8pt","weight":"normal","family":"sans-serif"}},"gridStyle":{"fill":{"r":0,"b":157,"g":100}},"lineStyle":{"fill":{"r":0,"b":157,"g":100},"stroke":{"width":0.6666667}},"titleStyle":{"padding":6.0,"fill":{"r":0,"b":157,"g":100},"font":{"size":"12pt","weight":"bold","family":"sans-serif"}},"title":["Observed Value"],"markStyle":{"fill":{"a":0,"r":0,"b":157,"g":100},"stroke":{"width":1.3333334}}}]}]}}],"data":[{"id":"dSource","fields":[{"min":0.5,"max":0.9,"id":"fVariable","label":"X Variable\_1"},{"min":-0.6111227618643652,"max":0.3854147390846435,"id":"fVariable1","label":"Y Axis"},{"min":39.0,"max":46.0,"id":"fVariable2","label":"Case Number"}],"rows":[[0.5,-0.6111227618643652,41],[0.8,0.3854147390846435,43],[0.9,0.1359376670361773,46],[0.9,0.1359376670361773,39]]}],"size":{"width":850.0,"height":500.0},"style":{"outline":{"a":0.0,"r":0,"b":0,"g":0},"fill":{"r":255,"b":255,"g":255}},"titles":[{"backgroundStyle":{"outline":{"a":0.0,"r":0,"b":0,"g":0},"fill":{"a":0.0,"r":0,"b":0,"g":0}},"style":{"padding":3.0,"fill":{"r":0,"b":157,"g":100},"font":{"size":"12pt","weight":"bold","family":"sans-serif"}},"type":"title","content":["Detrended Normal Q-Q Plot of V2 LogMar"]},{"backgroundStyle":{"outline":{"a":0.0,"r":0,"b":0,"g":0},"fill":{"a":0.0,"r":0,"b":0,"g":0}},"style":{"padding":3.0,"fill":{"r":0,"b":157,"g":100},"font":{"size":"10pt","weight":"bold","family":"sans-serif"}},"type":"title","content":["Albinism Type= OCA1A"]}],"version":"6.0"}

Detrended Normal Q-Q Plots  
Detrended Normal Q-Q Plots - For AlbinismType = OCA1B - May 11, 2022

{"copyright":"(C) Copyright IBM Corp. 2011","grammar":[{"elements":[{"data":{"$ref":"dSource"},"style":{"symbol":"circle","outline":{"r":0,"b":157,"g":100},"size":6.6666665,"fill":{"r":119,"b":119,"g":118}},"position":[{"field":{"$ref":"fVariable1"}},{"field":{"$ref":"fVariable"}}],"type":"point"},{"data":{"$ref":"dSource"},"style":{"fill":{"r":0,"b":157,"g":100},"stroke":{"width":3.3333333}},"position":[{"value":"0"},{"field":{"$ref":"fVariable"}}],"type":"line"}],"coordinates":{"style":{"outline":{"r":0,"b":157,"g":100},"fill":{"r":255,"b":255,"g":255}},"dimensions":[{"scale":{"padding":{"left":"5%","right":"5%"}},"axis":[{"tickStyle":{"padding":5.0,"fill":{"r":0,"b":157,"g":100},"font":{"size":"8pt","weight":"normal","family":"sans-serif"}},"gridStyle":{"fill":{"r":0,"b":157,"g":100}},"lineStyle":{"fill":{"r":0,"b":157,"g":100},"stroke":{"width":0.6666667}},"titleStyle":{"padding":6.0,"fill":{"r":0,"b":157,"g":100},"font":{"size":"12pt","weight":"bold","family":"sans-serif"}},"title":["Dev from Normal"],"markStyle":{"fill":{"a":0,"r":0,"b":157,"g":100},"stroke":{"width":1.3333334}}}]},{"scale":{"padding":{"left":"5%","right":"5%"}},"axis":[{"tickStyle":{"padding":5.0,"fill":{"r":0,"b":157,"g":100},"font":{"size":"8pt","weight":"normal","family":"sans-serif"}},"gridStyle":{"fill":{"r":0,"b":157,"g":100}},"lineStyle":{"fill":{"r":0,"b":157,"g":100},"stroke":{"width":0.6666667}},"titleStyle":{"padding":6.0,"fill":{"r":0,"b":157,"g":100},"font":{"size":"12pt","weight":"bold","family":"sans-serif"}},"title":["Observed Value"],"markStyle":{"fill":{"a":0,"r":0,"b":157,"g":100},"stroke":{"width":1.3333334}}}]}]}}],"data":[{"id":"dSource","fields":[{"min":0.6,"max":1.0,"id":"fVariable","label":"X Variable\_1"},{"min":-0.1858079296910399,"max":0.4758438649076059,"id":"fVariable1","label":"Y Axis"},{"min":51.0,"max":75.0,"id":"fVariable2","label":"Case Number"}],"rows":[[0.6,-0.1830738430230451,51],[0.7,-0.1858079296910399,75],[0.8,-0.106962092193519,58],[1,0.4758438649076059,61]]}],"size":{"width":850.0,"height":500.0},"style":{"outline":{"a":0.0,"r":0,"b":0,"g":0},"fill":{"r":255,"b":255,"g":255}},"titles":[{"backgroundStyle":{"outline":{"a":0.0,"r":0,"b":0,"g":0},"fill":{"a":0.0,"r":0,"b":0,"g":0}},"style":{"padding":3.0,"fill":{"r":0,"b":157,"g":100},"font":{"size":"12pt","weight":"bold","family":"sans-serif"}},"type":"title","content":["Detrended Normal Q-Q Plot of V2 LogMar"]},{"backgroundStyle":{"outline":{"a":0.0,"r":0,"b":0,"g":0},"fill":{"a":0.0,"r":0,"b":0,"g":0}},"style":{"padding":3.0,"fill":{"r":0,"b":157,"g":100},"font":{"size":"10pt","weight":"bold","family":"sans-serif"}},"type":"title","content":["Albinism Type= OCA1B"]}],"version":"6.0"}

Detrended Normal Q-Q Plots  
Detrended Normal Q-Q Plots - For AlbinismType = OCA2 - May 11, 2022

{"copyright":"(C) Copyright IBM Corp. 2011","grammar":[{"elements":[{"data":{"$ref":"dSource"},"style":{"symbol":"circle","outline":{"r":0,"b":157,"g":100},"size":6.6666665,"fill":{"r":119,"b":119,"g":118}},"position":[{"field":{"$ref":"fVariable1"}},{"field":{"$ref":"fVariable"}}],"type":"point"},{"data":{"$ref":"dSource"},"style":{"fill":{"r":0,"b":157,"g":100},"stroke":{"width":3.3333333}},"position":[{"value":"0"},{"field":{"$ref":"fVariable"}}],"type":"line"}],"coordinates":{"style":{"outline":{"r":0,"b":157,"g":100},"fill":{"r":255,"b":255,"g":255}},"dimensions":[{"scale":{"padding":{"left":"5%","right":"5%"}},"axis":[{"tickStyle":{"padding":5.0,"fill":{"r":0,"b":157,"g":100},"font":{"size":"8pt","weight":"normal","family":"sans-serif"}},"gridStyle":{"fill":{"r":0,"b":157,"g":100}},"lineStyle":{"fill":{"r":0,"b":157,"g":100},"stroke":{"width":0.6666667}},"titleStyle":{"padding":6.0,"fill":{"r":0,"b":157,"g":100},"font":{"size":"12pt","weight":"bold","family":"sans-serif"}},"title":["Dev from Normal"],"markStyle":{"fill":{"a":0,"r":0,"b":157,"g":100},"stroke":{"width":1.3333334}}}]},{"scale":{"padding":{"left":"5%","right":"5%"}},"axis":[{"tickStyle":{"padding":5.0,"fill":{"r":0,"b":157,"g":100},"font":{"size":"8pt","weight":"normal","family":"sans-serif"}},"gridStyle":{"fill":{"r":0,"b":157,"g":100}},"lineStyle":{"fill":{"r":0,"b":157,"g":100},"stroke":{"width":0.6666667}},"titleStyle":{"padding":6.0,"fill":{"r":0,"b":157,"g":100},"font":{"size":"12pt","weight":"bold","family":"sans-serif"}},"title":["Observed Value"],"markStyle":{"fill":{"a":0,"r":0,"b":157,"g":100},"stroke":{"width":1.3333334}}}]}]}}],"data":[{"id":"dSource","fields":[{"min":0.4,"max":0.8,"id":"fVariable","label":"X Variable\_1"},{"min":-0.5808806596113256,"max":0.197432102350306,"id":"fVariable1","label":"Y Axis"},{"min":89.0,"max":101.0,"id":"fVariable2","label":"Case Number"}],"rows":[[0.4,-0.5808806596113256,101],[0.6,0.03641612010267592,96],[0.6,0.03641612010267592,95],[0.7,0.1496778669051881,89],[0.8,0.197432102350306,100],[0.8,0.197432102350306,98]]}],"size":{"width":850.0,"height":500.0},"style":{"outline":{"a":0.0,"r":0,"b":0,"g":0},"fill":{"r":255,"b":255,"g":255}},"titles":[{"backgroundStyle":{"outline":{"a":0.0,"r":0,"b":0,"g":0},"fill":{"a":0.0,"r":0,"b":0,"g":0}},"style":{"padding":3.0,"fill":{"r":0,"b":157,"g":100},"font":{"size":"12pt","weight":"bold","family":"sans-serif"}},"type":"title","content":["Detrended Normal Q-Q Plot of V2 LogMar"]},{"backgroundStyle":{"outline":{"a":0.0,"r":0,"b":0,"g":0},"fill":{"a":0.0,"r":0,"b":0,"g":0}},"style":{"padding":3.0,"fill":{"r":0,"b":157,"g":100},"font":{"size":"10pt","weight":"bold","family":"sans-serif"}},"type":"title","content":["Albinism Type= OCA2"]}],"version":"6.0"}

Normal Q-Q Plots  
Normal Q-Q Plots - For AlbinismType = OCA1A - May 11, 2022

Normal Q-Q Plots  
Normal Q-Q Plots - For AlbinismType = OCA1B - May 11, 2022

Normal Q-Q Plots  
Normal Q-Q Plots - For AlbinismType = OCA2 - May 11, 2022

Detrended Normal Q-Q Plots  
Detrended Normal Q-Q Plots - For AlbinismType = OCA1A - May 11, 2022

{"copyright":"(C) Copyright IBM Corp. 2011","grammar":[{"elements":[{"data":{"$ref":"dSource"},"style":{"symbol":"circle","outline":{"r":0,"b":157,"g":100},"size":6.6666665,"fill":{"r":119,"b":119,"g":118}},"position":[{"field":{"$ref":"fVariable1"}},{"field":{"$ref":"fVariable"}}],"type":"point"},{"data":{"$ref":"dSource"},"style":{"fill":{"r":0,"b":157,"g":100},"stroke":{"width":3.3333333}},"position":[{"value":"0"},{"field":{"$ref":"fVariable"}}],"type":"line"}],"coordinates":{"style":{"outline":{"r":0,"b":157,"g":100},"fill":{"r":255,"b":255,"g":255}},"dimensions":[{"scale":{"padding":{"left":"5%","right":"5%"}},"axis":[{"tickStyle":{"padding":5.0,"fill":{"r":0,"b":157,"g":100},"font":{"size":"8pt","weight":"normal","family":"sans-serif"}},"gridStyle":{"fill":{"r":0,"b":157,"g":100}},"lineStyle":{"fill":{"r":0,"b":157,"g":100},"stroke":{"width":0.6666667}},"titleStyle":{"padding":6.0,"fill":{"r":0,"b":157,"g":100},"font":{"size":"12pt","weight":"bold","family":"sans-serif"}},"title":["Dev from Normal"],"markStyle":{"fill":{"a":0,"r":0,"b":157,"g":100},"stroke":{"width":1.3333334}}}]},{"scale":{"padding":{"left":"5%","right":"5%"}},"axis":[{"tickStyle":{"padding":5.0,"fill":{"r":0,"b":157,"g":100},"font":{"size":"8pt","weight":"normal","family":"sans-serif"}},"gridStyle":{"fill":{"r":0,"b":157,"g":100}},"lineStyle":{"fill":{"r":0,"b":157,"g":100},"stroke":{"width":0.6666667}},"titleStyle":{"padding":6.0,"fill":{"r":0,"b":157,"g":100},"font":{"size":"12pt","weight":"bold","family":"sans-serif"}},"title":["Observed Value"],"markStyle":{"fill":{"a":0,"r":0,"b":157,"g":100},"stroke":{"width":1.3333334}}}]}]}}],"data":[{"id":"dSource","fields":[{"min":0.4,"max":0.9,"id":"fVariable","label":"X Variable\_1"},{"min":-0.5471089160859132,"max":0.2533471031357993,"id":"fVariable1","label":"Y Axis"},{"min":39.0,"max":46.0,"id":"fVariable2","label":"Case Number"}],"rows":[[0.4,-0.5471089160859132,41],[0.7,0.2533471031357993,46],[0.8,0.2095629467504759,43],[0.9,0.0841988661996369,39]]}],"size":{"width":850.0,"height":500.0},"style":{"outline":{"a":0.0,"r":0,"b":0,"g":0},"fill":{"r":255,"b":255,"g":255}},"titles":[{"backgroundStyle":{"outline":{"a":0.0,"r":0,"b":0,"g":0},"fill":{"a":0.0,"r":0,"b":0,"g":0}},"style":{"padding":3.0,"fill":{"r":0,"b":157,"g":100},"font":{"size":"12pt","weight":"bold","family":"sans-serif"}},"type":"title","content":["Detrended Normal Q-Q Plot of V3 LogMar"]},{"backgroundStyle":{"outline":{"a":0.0,"r":0,"b":0,"g":0},"fill":{"a":0.0,"r":0,"b":0,"g":0}},"style":{"padding":3.0,"fill":{"r":0,"b":157,"g":100},"font":{"size":"10pt","weight":"bold","family":"sans-serif"}},"type":"title","content":["Albinism Type= OCA1A"]}],"version":"6.0"}

Detrended Normal Q-Q Plots  
Detrended Normal Q-Q Plots - For AlbinismType = OCA1B - May 11, 2022

{"copyright":"(C) Copyright IBM Corp. 2011","grammar":[{"elements":[{"data":{"$ref":"dSource"},"style":{"symbol":"circle","outline":{"r":0,"b":157,"g":100},"size":6.6666665,"fill":{"r":119,"b":119,"g":118}},"position":[{"field":{"$ref":"fVariable1"}},{"field":{"$ref":"fVariable"}}],"type":"point"},{"data":{"$ref":"dSource"},"style":{"fill":{"r":0,"b":157,"g":100},"stroke":{"width":3.3333333}},"position":[{"value":"0"},{"field":{"$ref":"fVariable"}}],"type":"line"}],"coordinates":{"style":{"outline":{"r":0,"b":157,"g":100},"fill":{"r":255,"b":255,"g":255}},"dimensions":[{"scale":{"padding":{"left":"5%","right":"5%"}},"axis":[{"tickStyle":{"padding":5.0,"fill":{"r":0,"b":157,"g":100},"font":{"size":"8pt","weight":"normal","family":"sans-serif"}},"gridStyle":{"fill":{"r":0,"b":157,"g":100}},"lineStyle":{"fill":{"r":0,"b":157,"g":100},"stroke":{"width":0.6666667}},"titleStyle":{"padding":6.0,"fill":{"r":0,"b":157,"g":100},"font":{"size":"12pt","weight":"bold","family":"sans-serif"}},"title":["Dev from Normal"],"markStyle":{"fill":{"a":0,"r":0,"b":157,"g":100},"stroke":{"width":1.3333334}}}]},{"scale":{"padding":{"left":"5%","right":"5%"}},"axis":[{"tickStyle":{"padding":5.0,"fill":{"r":0,"b":157,"g":100},"font":{"size":"8pt","weight":"normal","family":"sans-serif"}},"gridStyle":{"fill":{"r":0,"b":157,"g":100}},"lineStyle":{"fill":{"r":0,"b":157,"g":100},"stroke":{"width":0.6666667}},"titleStyle":{"padding":6.0,"fill":{"r":0,"b":157,"g":100},"font":{"size":"12pt","weight":"bold","family":"sans-serif"}},"title":["Observed Value"],"markStyle":{"fill":{"a":0,"r":0,"b":157,"g":100},"stroke":{"width":1.3333334}}}]}]}}],"data":[{"id":"dSource","fields":[{"min":0.6,"max":0.8,"id":"fVariable","label":"X Variable\_1"},{"min":-0.3416248910763975,"max":0.3416248910763985,"id":"fVariable1","label":"Y Axis"},{"min":51.0,"max":75.0,"id":"fVariable2","label":"Case Number"}],"rows":[[0.6,-0.3416248910763975,75],[0.6,-0.3416248910763975,51],[0.8,0.3416248910763985,61],[0.8,0.3416248910763985,58]]}],"size":{"width":850.0,"height":500.0},"style":{"outline":{"a":0.0,"r":0,"b":0,"g":0},"fill":{"r":255,"b":255,"g":255}},"titles":[{"backgroundStyle":{"outline":{"a":0.0,"r":0,"b":0,"g":0},"fill":{"a":0.0,"r":0,"b":0,"g":0}},"style":{"padding":3.0,"fill":{"r":0,"b":157,"g":100},"font":{"size":"12pt","weight":"bold","family":"sans-serif"}},"type":"title","content":["Detrended Normal Q-Q Plot of V3 LogMar"]},{"backgroundStyle":{"outline":{"a":0.0,"r":0,"b":0,"g":0},"fill":{"a":0.0,"r":0,"b":0,"g":0}},"style":{"padding":3.0,"fill":{"r":0,"b":157,"g":100},"font":{"size":"10pt","weight":"bold","family":"sans-serif"}},"type":"title","content":["Albinism Type= OCA1B"]}],"version":"6.0"}

Detrended Normal Q-Q Plots  
Detrended Normal Q-Q Plots - For AlbinismType = OCA2 - May 11, 2022

{"copyright":"(C) Copyright IBM Corp. 2011","grammar":[{"elements":[{"data":{"$ref":"dSource"},"style":{"symbol":"circle","outline":{"r":0,"b":157,"g":100},"size":6.6666665,"fill":{"r":119,"b":119,"g":118}},"position":[{"field":{"$ref":"fVariable1"}},{"field":{"$ref":"fVariable"}}],"type":"point"},{"data":{"$ref":"dSource"},"style":{"fill":{"r":0,"b":157,"g":100},"stroke":{"width":3.3333333}},"position":[{"value":"0"},{"field":{"$ref":"fVariable"}}],"type":"line"}],"coordinates":{"style":{"outline":{"r":0,"b":157,"g":100},"fill":{"r":255,"b":255,"g":255}},"dimensions":[{"scale":{"padding":{"left":"5%","right":"5%"}},"axis":[{"tickStyle":{"padding":5.0,"fill":{"r":0,"b":157,"g":100},"font":{"size":"8pt","weight":"normal","family":"sans-serif"}},"gridStyle":{"fill":{"r":0,"b":157,"g":100}},"lineStyle":{"fill":{"r":0,"b":157,"g":100},"stroke":{"width":0.6666667}},"titleStyle":{"padding":6.0,"fill":{"r":0,"b":157,"g":100},"font":{"size":"12pt","weight":"bold","family":"sans-serif"}},"title":["Dev from Normal"],"markStyle":{"fill":{"a":0,"r":0,"b":157,"g":100},"stroke":{"width":1.3333334}}}]},{"scale":{"padding":{"left":"5%","right":"5%"}},"axis":[{"tickStyle":{"padding":5.0,"fill":{"r":0,"b":157,"g":100},"font":{"size":"8pt","weight":"normal","family":"sans-serif"}},"gridStyle":{"fill":{"r":0,"b":157,"g":100}},"lineStyle":{"fill":{"r":0,"b":157,"g":100},"stroke":{"width":0.6666667}},"titleStyle":{"padding":6.0,"fill":{"r":0,"b":157,"g":100},"font":{"size":"12pt","weight":"bold","family":"sans-serif"}},"title":["Observed Value"],"markStyle":{"fill":{"a":0,"r":0,"b":157,"g":100},"stroke":{"width":1.3333334}}}]}]}}],"data":[{"id":"dSource","fields":[{"min":0.3,"max":0.8,"id":"fVariable","label":"X Variable\_1"},{"min":-0.3321974170876356,"max":0.2698069474626692,"id":"fVariable1","label":"Y Axis"},{"min":89.0,"max":101.0,"id":"fVariable2","label":"Case Number"}],"rows":[[0.3,-0.3204736636989929,101],[0.4,-0.3321974170876356,95],[0.6,0.08164965809277232,96],[0.6,0.08164965809277232,89],[0.8,0.2698069474626692,100],[0.8,0.2698069474626692,98]]}],"size":{"width":850.0,"height":500.0},"style":{"outline":{"a":0.0,"r":0,"b":0,"g":0},"fill":{"r":255,"b":255,"g":255}},"titles":[{"backgroundStyle":{"outline":{"a":0.0,"r":0,"b":0,"g":0},"fill":{"a":0.0,"r":0,"b":0,"g":0}},"style":{"padding":3.0,"fill":{"r":0,"b":157,"g":100},"font":{"size":"12pt","weight":"bold","family":"sans-serif"}},"type":"title","content":["Detrended Normal Q-Q Plot of V3 LogMar"]},{"backgroundStyle":{"outline":{"a":0.0,"r":0,"b":0,"g":0},"fill":{"a":0.0,"r":0,"b":0,"g":0}},"style":{"padding":3.0,"fill":{"r":0,"b":157,"g":100},"font":{"size":"10pt","weight":"bold","family":"sans-serif"}},"type":"title","content":["Albinism Type= OCA2"]}],"version":"6.0"}

Normal Q-Q Plots  
Normal Q-Q Plots - For AlbinismType = OCA1A - May 11, 2022

Normal Q-Q Plots  
Normal Q-Q Plots - For AlbinismType = OCA1B - May 11, 2022

Normal Q-Q Plots  
Normal Q-Q Plots - For AlbinismType = OCA2 - May 11, 2022

Detrended Normal Q-Q Plots  
Detrended Normal Q-Q Plots - For AlbinismType = OCA1A - May 11, 2022

{"copyright":"(C) Copyright IBM Corp. 2011","grammar":[{"elements":[{"data":{"$ref":"dSource"},"style":{"symbol":"circle","outline":{"r":0,"b":157,"g":100},"size":6.6666665,"fill":{"r":119,"b":119,"g":118}},"position":[{"field":{"$ref":"fVariable1"}},{"field":{"$ref":"fVariable"}}],"type":"point"},{"data":{"$ref":"dSource"},"style":{"fill":{"r":0,"b":157,"g":100},"stroke":{"width":3.3333333}},"position":[{"value":"0"},{"field":{"$ref":"fVariable"}}],"type":"line"}],"coordinates":{"style":{"outline":{"r":0,"b":157,"g":100},"fill":{"r":255,"b":255,"g":255}},"dimensions":[{"scale":{"padding":{"left":"5%","right":"5%"}},"axis":[{"tickStyle":{"padding":5.0,"fill":{"r":0,"b":157,"g":100},"font":{"size":"8pt","weight":"normal","family":"sans-serif"}},"gridStyle":{"fill":{"r":0,"b":157,"g":100}},"lineStyle":{"fill":{"r":0,"b":157,"g":100},"stroke":{"width":0.6666667}},"titleStyle":{"padding":6.0,"fill":{"r":0,"b":157,"g":100},"font":{"size":"12pt","weight":"bold","family":"sans-serif"}},"title":["Dev from Normal"],"markStyle":{"fill":{"a":0,"r":0,"b":157,"g":100},"stroke":{"width":1.3333334}}}]},{"scale":{"padding":{"left":"5%","right":"5%"}},"axis":[{"tickStyle":{"padding":5.0,"fill":{"r":0,"b":157,"g":100},"font":{"size":"8pt","weight":"normal","family":"sans-serif"}},"gridStyle":{"fill":{"r":0,"b":157,"g":100}},"lineStyle":{"fill":{"r":0,"b":157,"g":100},"stroke":{"width":0.6666667}},"titleStyle":{"padding":6.0,"fill":{"r":0,"b":157,"g":100},"font":{"size":"12pt","weight":"bold","family":"sans-serif"}},"title":["Observed Value"],"markStyle":{"fill":{"a":0,"r":0,"b":157,"g":100},"stroke":{"width":1.3333334}}}]}]}}],"data":[{"id":"dSource","fields":[{"min":0.35,"max":0.9,"id":"fVariable","label":"X Variable\_1"},{"min":-0.6306307058708335,"max":0.3553711577967667,"id":"fVariable1","label":"Y Axis"},{"min":39.0,"max":46.0,"id":"fVariable2","label":"Case Number"}],"rows":[[0.35,-0.6306307058708335,41],[0.8,0.3553711577967667,46],[0.8,0.3553711577967667,43],[0.9,-0.08011160972270026,39]]}],"size":{"width":850.0,"height":500.0},"style":{"outline":{"a":0.0,"r":0,"b":0,"g":0},"fill":{"r":255,"b":255,"g":255}},"titles":[{"backgroundStyle":{"outline":{"a":0.0,"r":0,"b":0,"g":0},"fill":{"a":0.0,"r":0,"b":0,"g":0}},"style":{"padding":3.0,"fill":{"r":0,"b":157,"g":100},"font":{"size":"12pt","weight":"bold","family":"sans-serif"}},"type":"title","content":["Detrended Normal Q-Q Plot of V4 LogMar"]},{"backgroundStyle":{"outline":{"a":0.0,"r":0,"b":0,"g":0},"fill":{"a":0.0,"r":0,"b":0,"g":0}},"style":{"padding":3.0,"fill":{"r":0,"b":157,"g":100},"font":{"size":"10pt","weight":"bold","family":"sans-serif"}},"type":"title","content":["Albinism Type= OCA1A"]}],"version":"6.0"}

Detrended Normal Q-Q Plots  
Detrended Normal Q-Q Plots - For AlbinismType = OCA1B - May 11, 2022

{"copyright":"(C) Copyright IBM Corp. 2011","grammar":[{"elements":[{"data":{"$ref":"dSource"},"style":{"symbol":"circle","outline":{"r":0,"b":157,"g":100},"size":6.6666665,"fill":{"r":119,"b":119,"g":118}},"position":[{"field":{"$ref":"fVariable1"}},{"field":{"$ref":"fVariable"}}],"type":"point"},{"data":{"$ref":"dSource"},"style":{"fill":{"r":0,"b":157,"g":100},"stroke":{"width":3.3333333}},"position":[{"value":"0"},{"field":{"$ref":"fVariable"}}],"type":"line"}],"coordinates":{"style":{"outline":{"r":0,"b":157,"g":100},"fill":{"r":255,"b":255,"g":255}},"dimensions":[{"scale":{"padding":{"left":"5%","right":"5%"}},"axis":[{"tickStyle":{"padding":5.0,"fill":{"r":0,"b":157,"g":100},"font":{"size":"8pt","weight":"normal","family":"sans-serif"}},"gridStyle":{"fill":{"r":0,"b":157,"g":100}},"lineStyle":{"fill":{"r":0,"b":157,"g":100},"stroke":{"width":0.6666667}},"titleStyle":{"padding":6.0,"fill":{"r":0,"b":157,"g":100},"font":{"size":"12pt","weight":"bold","family":"sans-serif"}},"title":["Dev from Normal"],"markStyle":{"fill":{"a":0,"r":0,"b":157,"g":100},"stroke":{"width":1.3333334}}}]},{"scale":{"padding":{"left":"5%","right":"5%"}},"axis":[{"tickStyle":{"padding":5.0,"fill":{"r":0,"b":157,"g":100},"font":{"size":"8pt","weight":"normal","family":"sans-serif"}},"gridStyle":{"fill":{"r":0,"b":157,"g":100}},"lineStyle":{"fill":{"r":0,"b":157,"g":100},"stroke":{"width":0.6666667}},"titleStyle":{"padding":6.0,"fill":{"r":0,"b":157,"g":100},"font":{"size":"12pt","weight":"bold","family":"sans-serif"}},"title":["Observed Value"],"markStyle":{"fill":{"a":0,"r":0,"b":157,"g":100},"stroke":{"width":1.3333334}}}]}]}}],"data":[{"id":"dSource","fields":[{"min":0.6,"max":0.7,"id":"fVariable","label":"X Variable\_1"},{"min":-0.341624891076397,"max":0.341624891076399,"id":"fVariable1","label":"Y Axis"},{"min":51.0,"max":75.0,"id":"fVariable2","label":"Case Number"}],"rows":[[0.6,-0.341624891076397,75],[0.6,-0.341624891076397,51],[0.7,0.341624891076399,61],[0.7,0.341624891076399,58]]}],"size":{"width":850.0,"height":500.0},"style":{"outline":{"a":0.0,"r":0,"b":0,"g":0},"fill":{"r":255,"b":255,"g":255}},"titles":[{"backgroundStyle":{"outline":{"a":0.0,"r":0,"b":0,"g":0},"fill":{"a":0.0,"r":0,"b":0,"g":0}},"style":{"padding":3.0,"fill":{"r":0,"b":157,"g":100},"font":{"size":"12pt","weight":"bold","family":"sans-serif"}},"type":"title","content":["Detrended Normal Q-Q Plot of V4 LogMar"]},{"backgroundStyle":{"outline":{"a":0.0,"r":0,"b":0,"g":0},"fill":{"a":0.0,"r":0,"b":0,"g":0}},"style":{"padding":3.0,"fill":{"r":0,"b":157,"g":100},"font":{"size":"10pt","weight":"bold","family":"sans-serif"}},"type":"title","content":["Albinism Type= OCA1B"]}],"version":"6.0"}

Detrended Normal Q-Q Plots  
Detrended Normal Q-Q Plots - For AlbinismType = OCA2 - May 11, 2022

{"copyright":"(C) Copyright IBM Corp. 2011","grammar":[{"elements":[{"data":{"$ref":"dSource"},"style":{"symbol":"circle","outline":{"r":0,"b":157,"g":100},"size":6.6666665,"fill":{"r":119,"b":119,"g":118}},"position":[{"field":{"$ref":"fVariable1"}},{"field":{"$ref":"fVariable"}}],"type":"point"},{"data":{"$ref":"dSource"},"style":{"fill":{"r":0,"b":157,"g":100},"stroke":{"width":3.3333333}},"position":[{"value":"0"},{"field":{"$ref":"fVariable"}}],"type":"line"}],"coordinates":{"style":{"outline":{"r":0,"b":157,"g":100},"fill":{"r":255,"b":255,"g":255}},"dimensions":[{"scale":{"padding":{"left":"5%","right":"5%"}},"axis":[{"tickStyle":{"padding":5.0,"fill":{"r":0,"b":157,"g":100},"font":{"size":"8pt","weight":"normal","family":"sans-serif"}},"gridStyle":{"fill":{"r":0,"b":157,"g":100}},"lineStyle":{"fill":{"r":0,"b":157,"g":100},"stroke":{"width":0.6666667}},"titleStyle":{"padding":6.0,"fill":{"r":0,"b":157,"g":100},"font":{"size":"12pt","weight":"bold","family":"sans-serif"}},"title":["Dev from Normal"],"markStyle":{"fill":{"a":0,"r":0,"b":157,"g":100},"stroke":{"width":1.3333334}}}]},{"scale":{"padding":{"left":"5%","right":"5%"}},"axis":[{"tickStyle":{"padding":5.0,"fill":{"r":0,"b":157,"g":100},"font":{"size":"8pt","weight":"normal","family":"sans-serif"}},"gridStyle":{"fill":{"r":0,"b":157,"g":100}},"lineStyle":{"fill":{"r":0,"b":157,"g":100},"stroke":{"width":0.6666667}},"titleStyle":{"padding":6.0,"fill":{"r":0,"b":157,"g":100},"font":{"size":"12pt","weight":"bold","family":"sans-serif"}},"title":["Observed Value"],"markStyle":{"fill":{"a":0,"r":0,"b":157,"g":100},"stroke":{"width":1.3333334}}}]}]}}],"data":[{"id":"dSource","fields":[{"min":0.3,"max":0.8,"id":"fVariable","label":"X Variable\_1"},{"min":-0.3249321731022477,"max":0.2350932894260949,"id":"fVariable1","label":"Y Axis"},{"min":89.0,"max":101.0,"id":"fVariable2","label":"Case Number"}],"rows":[[0.3,-0.3249321731022477,101],[0.4,-0.287520572990601,95],[0.55,0.1350929279546288,96],[0.6,0.04458483939768093,89],[0.7,0.1976816893144477,98],[0.8,0.2350932894260949,100]]}],"size":{"width":850.0,"height":500.0},"style":{"outline":{"a":0.0,"r":0,"b":0,"g":0},"fill":{"r":255,"b":255,"g":255}},"titles":[{"backgroundStyle":{"outline":{"a":0.0,"r":0,"b":0,"g":0},"fill":{"a":0.0,"r":0,"b":0,"g":0}},"style":{"padding":3.0,"fill":{"r":0,"b":157,"g":100},"font":{"size":"12pt","weight":"bold","family":"sans-serif"}},"type":"title","content":["Detrended Normal Q-Q Plot of V4 LogMar"]},{"backgroundStyle":{"outline":{"a":0.0,"r":0,"b":0,"g":0},"fill":{"a":0.0,"r":0,"b":0,"g":0}},"style":{"padding":3.0,"fill":{"r":0,"b":157,"g":100},"font":{"size":"10pt","weight":"bold","family":"sans-serif"}},"type":"title","content":["Albinism Type= OCA2"]}],"version":"6.0"}

Log  
Log - Log - May 11, 2022

USE ALL.  
COMPUTE filter\_$=(AlbinismType=1  | AlbinismType=3).  
VARIABLE LABELS filter\_$ 'AlbinismType=1  | AlbinismType=3 (FILTER)'.  
VALUE LABELS filter\_$ 0 'Not Selected' 1 'Selected'.  
FORMATS filter\_$ (f1.0).  
FILTER BY filter\_$.  
EXECUTE.  
  
T-TEST PAIRS=V1LogMar V2LogMar V3LogMar V4LogMar V1LogMar V2LogMar WITH V2LogMar V3LogMar V4LogMar  
    V1LogMar V3LogMar V4LogMar (PAIRED)  
  /CRITERIA=CI(.9500)  
  /MISSING=ANALYSIS.

T-Test  
T-Test - Paired Samples Statistics - May 11, 2022

Paired Samples StatisticsPaired Samples Statistics, table, 1 levels of column headers and 3 levels of row headers, table with 7 columns and 26 rows

|  |  |  |  |  |  |  |
| --- | --- | --- | --- | --- | --- | --- |
| Albinism Type | | | Mean | N | Std. Deviation | Std. Error Mean |
| OCA1A | Pair 1 | V1 LogMar | .9500 | 16 | .32863 | .08216 |
|  |  | V2 LogMar | .8688 | 16 | .20238 | .05060 |
|  | Pair 2 | V2 LogMar | .7571 | 7 | .15119 | .05714 |
|  |  | V3 LogMar | .7143 | 7 | .15736 | .05948 |
|  | Pair 3 | V3 LogMar | .7000 | 5 | .18708 | .08367 |
|  |  | V4 LogMar | .6900 | 5 | .21909 | .09798 |
|  | Pair 4 | V4 LogMar | .7125 | 4 | .24622 | .12311 |
|  |  | V1 LogMar | .8000 | 4 | .14142 | .07071 |
|  | Pair 5 | V1 LogMar | .8143 | 7 | .12150 | .04592 |
|  |  | V3 LogMar | .7143 | 7 | .15736 | .05948 |
|  | Pair 6 | V2 LogMar | .7750 | 4 | .18930 | .09465 |
|  |  | V4 LogMar | .7125 | 4 | .24622 | .12311 |
| OCA2 | Pair 1 | V1 LogMar | .7043 | 23 | .18210 | .03797 |
|  |  | V2 LogMar | .6217 | 23 | .19761 | .04121 |
|  | Pair 2 | V2 LogMar | .6100 | 10 | .16633 | .05260 |
|  |  | V3 LogMar | .5500 | 10 | .17795 | .05627 |
|  | Pair 3 | V3 LogMar | .5556 | 9 | .24552 | .08184 |
|  |  | V4 LogMar | .5278 | 9 | .23333 | .07778 |
|  | Pair 4 | V4 LogMar | .5583 | 6 | .18552 | .07574 |
|  |  | V1 LogMar | .7000 | 6 | .18974 | .07746 |
|  | Pair 5 | V1 LogMar | .6889 | 9 | .19003 | .06334 |
|  |  | V3 LogMar | .5444 | 9 | .18782 | .06261 |
|  | Pair 6 | V2 LogMar | .6571 | 7 | .13973 | .05281 |
|  |  | V4 LogMar | .5500 | 7 | .17078 | .06455 |
|  |  |  |  |  |  |  |

T-Test  
T-Test - Paired Samples Correlations - May 11, 2022

Paired Samples CorrelationsPaired Samples Correlations, table, 1 levels of column headers and 3 levels of row headers, table with 6 columns and 14 rows

|  |  |  |  |  |  |
| --- | --- | --- | --- | --- | --- |
| Albinism Type | | | N | Correlation | Sig. |
| OCA1A | Pair 1 | V1 LogMar & V2 LogMar | 16 | .847 | .000 |
|  | Pair 2 | V2 LogMar & V3 LogMar | 7 | .801 | .031 |
|  | Pair 3 | V3 LogMar & V4 LogMar | 5 | .945 | .015 |
|  | Pair 4 | V4 LogMar & V1 LogMar | 4 | .957 | .043 |
|  | Pair 5 | V1 LogMar & V3 LogMar | 7 | .859 | .013 |
|  | Pair 6 | V2 LogMar & V4 LogMar | 4 | .974 | .026 |
| OCA2 | Pair 1 | V1 LogMar & V2 LogMar | 23 | .806 | .000 |
|  | Pair 2 | V2 LogMar & V3 LogMar | 10 | .920 | .000 |
|  | Pair 3 | V3 LogMar & V4 LogMar | 9 | .984 | .000 |
|  | Pair 4 | V4 LogMar & V1 LogMar | 6 | .852 | .031 |
|  | Pair 5 | V1 LogMar & V3 LogMar | 9 | .891 | .001 |
|  | Pair 6 | V2 LogMar & V4 LogMar | 7 | .908 | .005 |
|  |  |  |  |  |  |

T-Test  
T-Test - Paired Samples Test - May 11, 2022

Paired Samples TestPaired Samples Test, table, 3 levels of column headers and 3 levels of row headers, table with 11 columns and 16 rows

|  |  |  |  |  |  |  |  |  |  |  |
| --- | --- | --- | --- | --- | --- | --- | --- | --- | --- | --- |
| Albinism Type | | | Paired Differences | | | | | t | df | Sig. (2-tailed) |
|  |  |  | Mean | Std. Deviation | Std. Error Mean | 95% Confidence Interval of the Difference | |  |  |  |
|  |  |  |  |  |  | Lower | Upper |  |  |  |
| OCA1A | Pair 1 | V1 LogMar - V2 LogMar | .08125 | .19050 | .04763 | -.02026 | .18276 | 1.706 | 15 | .109 |
|  | Pair 2 | V2 LogMar - V3 LogMar | .04286 | .09759 | .03689 | -.04740 | .13311 | 1.162 | 6 | .289 |
|  | Pair 3 | V3 LogMar - V4 LogMar | .01000 | .07416 | .03317 | -.08208 | .10208 | .302 | 4 | .778 |
|  | Pair 4 | V4 LogMar - V1 LogMar | -.08750 | .11815 | .05907 | -.27550 | .10050 | -1.481 | 3 | .235 |
|  | Pair 5 | V1 LogMar - V3 LogMar | .10000 | .08165 | .03086 | .02449 | .17551 | 3.240 | 6 | .018 |
|  | Pair 6 | V2 LogMar - V4 LogMar | .06250 | .07500 | .03750 | -.05684 | .18184 | 1.667 | 3 | .194 |
| OCA2 | Pair 1 | V1 LogMar - V2 LogMar | .08261 | .11929 | .02487 | .03103 | .13419 | 3.321 | 22 | .003 |
|  | Pair 2 | V2 LogMar - V3 LogMar | .06000 | .06992 | .02211 | .00998 | .11002 | 2.714 | 9 | .024 |
|  | Pair 3 | V3 LogMar - V4 LogMar | .02778 | .04410 | .01470 | -.00612 | .06167 | 1.890 | 8 | .095 |
|  | Pair 4 | V4 LogMar - V1 LogMar | -.14167 | .10206 | .04167 | -.24877 | -.03456 | -3.400 | 5 | .019 |
|  | Pair 5 | V1 LogMar - V3 LogMar | .14444 | .08819 | .02940 | .07665 | .21223 | 4.914 | 8 | .001 |
|  | Pair 6 | V2 LogMar - V4 LogMar | .10714 | .07319 | .02766 | .03945 | .17483 | 3.873 | 6 | .008 |
|  |  |  |  |  |  |  |  |  |  |  |

Log  
Log - Log - May 11, 2022

USE ALL.  
COMPUTE filter\_$=(AlbinismType=2).  
VARIABLE LABELS filter\_$ 'AlbinismType=2 (FILTER)'.  
VALUE LABELS filter\_$ 0 'Not Selected' 1 'Selected'.  
FORMATS filter\_$ (f1.0).  
FILTER BY filter\_$.  
EXECUTE.  
  
  
\*Nonparametric Tests: Related Samples.  
NPTESTS  
  /RELATED TEST(V1LogMar V2LogMar V3LogMar V4LogMar)  
  /MISSING SCOPE=ANALYSIS USERMISSING=EXCLUDE  
  /CRITERIA ALPHA=0.05  CILEVEL=95.

Nonparametric Tests  
Nonparametric Tests - Hypothesis Test Summary - May 11, 2022

Hypothesis Test SummaryHypothesis Test Summary, table, 1 levels of column headers and 2 levels of row headers, table with 6 columns and 4 rows

|  |  |  |  |  |  |
| --- | --- | --- | --- | --- | --- |
| Albinism Type | | Null Hypothesis | Test | Sig. | Decision |
| OCA1B | 1 | The distributions of V1 LogMar, V2 LogMar, V3 LogMar and V4 LogMar are the same. | Related-Samples Friedman's Two-Way Analysis of Variance by Ranks | .127 | Retain the null hypothesis. |
|  |  |  |  |  |  |
| --- | --- | --- | --- | --- | --- |
| Asymptotic significances are displayed. The significance level is .050. | | | | | |
|  |  |  |  |  |  |

V1 LogMar, V2 LogMar, V3 LogMar, V4 LogMar  
V1 LogMar, V2 LogMar, V3 LogMar, V4 LogMar - Related-Samples Friedman's Two-Way Analysis of Variance(more) by Ranks Summary - May 11, 2022(less)

Related-Samples Friedman's Two-Way Analysis of Variance by Ranks SummaryRelated-Samples Friedman's Two-Way Analysis of Variance by Ranks Summary, table, 0 levels of column headers and 2 levels of row headers, table with 3 columns and 6 rows

|  |  |  |
| --- | --- | --- |
| OCA1B | Total N | 4 |
|  | Test Statistic | 5.710a |
|  | Degree Of Freedom | 3 |
|  | Asymptotic Sig.(2-sided test) | .127 |
|  |  |  |
| --- | --- | --- |
| a. Multiple comparisons are not performed because the overall test retained the null hypothesis of no differences. | | |
|  |  |  |

V1 LogMar, V2 LogMar, V3 LogMar, V4 LogMar  
V1 LogMar, V2 LogMar, V3 LogMar, V4 LogMar - Related-Samples Friedman's Two-Way Analysis of Variance(more) by Ranks; Albinism Type: OCA1B - May 11, 2022(less)

Nonparametric Tests  
Nonparametric Tests - Continuous Field Information V1 LogMar; Albinism Type: OCA1B - May 11, 2022

Nonparametric Tests  
Nonparametric Tests - Continuous Field Information V2 LogMar; Albinism Type: OCA1B - May 11, 2022

Nonparametric Tests  
Nonparametric Tests - Continuous Field Information V3 LogMar; Albinism Type: OCA1B - May 11, 2022

Nonparametric Tests  
Nonparametric Tests - Continuous Field Information V4 LogMar; Albinism Type: OCA1B - May 11, 2022

Log  
Log - Log - May 11, 2022

DATASET ACTIVATE DataSet1.  
  
SAVE OUTFILE='D:\liavofra\עבודה\לקוחות\Hadassah\Claudia\Albinism\WF2\_new.sav'  
  /COMPRESSED.  
USE ALL.  
COMPUTE filter\_$=(AlbinismType=1  | AlbinismType=2  | AlbinismType=3).  
VARIABLE LABELS filter\_$ 'AlbinismType=1  | AlbinismType=2  | AlbinismType=3 (FILTER)'.  
VALUE LABELS filter\_$ 0 'Not Selected' 1 'Selected'.  
FORMATS filter\_$ (f1.0).  
FILTER BY filter\_$.  
EXECUTE.  
CROSSTABS  
  /TABLES=AlbinismType BY nystagmus Albinism Eyemusclesurgery Srabismus RE\_Myopia RE\_referror  
    RE\_cyl LE\_Myopia LE\_referror LE\_cyl  
  /FORMAT=AVALUE TABLES  
  /STATISTICS=CHISQ  
  /CELLS=COUNT ROW COLUMN TOTAL  
  /COUNT ROUND CELL  
  /BARCHART.

Crosstabs  
Crosstabs - Warnings - May 11, 2022

WarningsWarnings, table, 0 levels of column headers and 0 levels of row headers, table with 1 columns and 3 rows

|  |
| --- |
| The variable Albinism Type is in use for SPLIT FILES, so it may not be used in a CROSSTABS command. |
| Execution of this command stops. |
|  |

Log  
Log - Log - May 11, 2022

FILTER OFF.  
USE ALL.  
EXECUTE.  
DATASET ACTIVATE DataSet1.  
  
SAVE OUTFILE='D:\liavofra\עבודה\לקוחות\Hadassah\Claudia\Albinism\WF2\_new.sav'  
  /COMPRESSED.  
CROSSTABS  
  /TABLES=AlbinismType BY nystagmus Albinism Eyemusclesurgery Srabismus RE\_Myopia RE\_referror  
    RE\_cyl LE\_Myopia LE\_referror LE\_cyl  
  /FORMAT=AVALUE TABLES  
  /STATISTICS=CHISQ  
  /CELLS=COUNT ROW COLUMN TOTAL  
  /COUNT ROUND CELL  
  /BARCHART.

Crosstabs  
Crosstabs - Warnings - May 11, 2022

WarningsWarnings, table, 0 levels of column headers and 0 levels of row headers, table with 1 columns and 3 rows

|  |
| --- |
| The variable Albinism Type is in use for SPLIT FILES, so it may not be used in a CROSSTABS command. |
| Execution of this command stops. |
|  |

Log  
Log - Log - May 11, 2022

SPLIT FILE OFF.  
CROSSTABS  
  /TABLES=AlbinismType BY nystagmus Albinism Eyemusclesurgery Srabismus RE\_Myopia RE\_referror  
    RE\_cyl LE\_Myopia LE\_referror LE\_cyl  
  /FORMAT=AVALUE TABLES  
  /STATISTICS=CHISQ  
  /CELLS=COUNT ROW COLUMN TOTAL  
  /COUNT ROUND CELL  
  /BARCHART.

Crosstabs  
Crosstabs - Case Processing Summary - May 11, 2022

Case Processing SummaryCase Processing Summary, table, 3 levels of column headers and 1 levels of row headers, table with 7 columns and 14 rows

|  |  |  |  |  |  |  |
| --- | --- | --- | --- | --- | --- | --- |
|  | Cases | | | | | |
|  | Valid | | Missing | | Total | |
|  | N | Percent | N | Percent | N | Percent |
| Albinism Type \* nystagmus | 71 | 67.6% | 34 | 32.4% | 105 | 100.0% |
| Albinism Type \* Albinism | 71 | 67.6% | 34 | 32.4% | 105 | 100.0% |
| Albinism Type \* Eye muscle surgery | 71 | 67.6% | 34 | 32.4% | 105 | 100.0% |
| Albinism Type \* Srabismus | 70 | 66.7% | 35 | 33.3% | 105 | 100.0% |
| Albinism Type \* RE\_Myopia | 71 | 67.6% | 34 | 32.4% | 105 | 100.0% |
| Albinism Type \* RE\_ref error | 71 | 67.6% | 34 | 32.4% | 105 | 100.0% |
| Albinism Type \* RE\_cyl | 71 | 67.6% | 34 | 32.4% | 105 | 100.0% |
| Albinism Type \* LE\_Myopia | 70 | 66.7% | 35 | 33.3% | 105 | 100.0% |
| Albinism Type \* LE\_ref error | 70 | 66.7% | 35 | 33.3% | 105 | 100.0% |
| Albinism Type \* LE\_cyl | 70 | 66.7% | 35 | 33.3% | 105 | 100.0% |
|  |  |  |  |  |  |  |

Albinism Type \* nystagmus  
Albinism Type \* nystagmus - Crosstab - May 11, 2022

CrosstabCrosstab, table, 2 levels of column headers and 3 levels of row headers, table with 6 columns and 19 rows

|  |  |  |  |  |  |
| --- | --- | --- | --- | --- | --- |
|  | | | nystagmus | | Total |
|  |  |  | No | Yes |  |
| Albinism Type | OCA1A | Count | 0 | 17 | 17 |
|  |  | % within Albinism Type | 0.0% | 100.0% | 100.0% |
|  |  | % within nystagmus | 0.0% | 25.8% | 23.9% |
|  |  | % of Total | 0.0% | 23.9% | 23.9% |
|  | OCA1B | Count | 3 | 25 | 28 |
|  |  | % within Albinism Type | 10.7% | 89.3% | 100.0% |
|  |  | % within nystagmus | 60.0% | 37.9% | 39.4% |
|  |  | % of Total | 4.2% | 35.2% | 39.4% |
|  | OCA2 | Count | 2 | 24 | 26 |
|  |  | % within Albinism Type | 7.7% | 92.3% | 100.0% |
|  |  | % within nystagmus | 40.0% | 36.4% | 36.6% |
|  |  | % of Total | 2.8% | 33.8% | 36.6% |
| Total | | Count | 5 | 66 | 71 |
|  |  | % within Albinism Type | 7.0% | 93.0% | 100.0% |
|  |  | % within nystagmus | 100.0% | 100.0% | 100.0% |
|  |  | % of Total | 7.0% | 93.0% | 100.0% |
|  |  |  |  |  |  |

Albinism Type \* nystagmus  
Albinism Type \* nystagmus - Chi-Square Tests - May 11, 2022

Chi-Square TestsChi-Square Tests, table, 1 levels of column headers and 1 levels of row headers, table with 4 columns and 7 rows

|  |  |  |  |
| --- | --- | --- | --- |
|  | Value | df | Asymptotic Significance (2-sided) |
| Pearson Chi-Square | 1.881a | 2 | .390 |
| Likelihood Ratio | 3.002 | 2 | .223 |
| Linear-by-Linear Association | .672 | 1 | .413 |
| N of Valid Cases | 71 |  |  |
|  |  |  |  |
| --- | --- | --- | --- |
| a. 3 cells (50.0%) have expected count less than 5. The minimum expected count is 1.20. | | | |
|  |  |  |  |

Albinism Type \* nystagmus  
Albinism Type \* nystagmus - Bar Chart - May 11, 2022

{"copyright":"(C) Copyright IBM Corp. 2011","grammar":[{"elements":[{"color":[{"field":{"$ref":"fVariable2"},"palette":["#00B2EF","#D9182D","#FFCF10","#7F1C7D","#007670","#DD731C","#17AF4B","#EE3D96","#838329","#82D1F5","#F04E37","#FFE14F","#AB1A86","#00A6A0","#F19027","#8CC63F","#F389AF","#A5A215","#00649D","#A91024","#FDB813","#3B2056","#006058","#B8471B","#00BA52","#BA006E","#594F13"],"id":"colorAestheticID"}],"data":{"$ref":"dSource"},"style":{"outline":{"r":0,"b":0,"g":0},"size":"100.0%","width":"90.0%","stroke":{"width":0.0}},"position":[{"field":{"$ref":"fVariable1"}},{"field":{"$ref":"fVariable"}},{"field":{"$ref":"fVariable2"}}],"type":"interval"}],"coordinates":{"transforms":[{"type":"cluster"}],"style":{"outline":{"r":0,"b":157,"g":100},"fill":{"r":255,"b":255,"g":255}},"dimensions":[{"scale":{"padding":{"right":"5%"},"spans":[{"outRange":{"min":0.0,"max":1.0}}]},"axis":[{"tickStyle":{"padding":5.0,"fill":{"r":0,"b":157,"g":100},"font":{"size":"8pt","weight":"normal","family":"sans-serif"}},"gridStyle":{"fill":{"r":0,"b":157,"g":100}},"lineStyle":{"fill":{"r":0,"b":157,"g":100},"stroke":{"width":0.6666667}},"titleStyle":{"padding":6.0,"fill":{"r":0,"b":157,"g":100},"font":{"size":"12pt","weight":"bold","family":"sans-serif"}},"title":["Count"],"markStyle":{"fill":{"a":0,"r":0,"b":157,"g":100},"stroke":{"width":1.3333334}}}]},{"scale":{"categoryStructure":{"gap":"15%","nest":true},"local":false},"axis":[{"tickStyle":{"padding":5.0,"fill":{"r":0,"b":157,"g":100},"font":{"size":"12pt","weight":"normal","family":"sans-serif"}},"lineStyle":{"fill":{"r":0,"b":157,"g":100},"stroke":{"width":0.6666667}},"titleStyle":{"padding":6.0,"fill":{"r":0,"b":157,"g":100},"font":{"size":"12pt","weight":"bold","family":"sans-serif"}},"title":["Albinism Type"],"markStyle":{"fill":{"a":0,"r":0,"b":157,"g":100},"stroke":{"width":1.3333334}}}]},{}]}}],"legends":[{"labelStyle":{"fill":{"r":0,"b":157,"g":100},"font":{"size":"10pt","weight":"normal","family":"sans-serif"}},"itemStyle":{"padding":3,"size":11},"titleStyle":{"padding":3.0,"fill":{"r":0,"b":157,"g":100},"align":"start","font":{"size":"12pt","weight":"bold","family":"sans-serif"}},"location":{"width":"24%"},"title":["nystagmus"],"aesthetics":[{"aesthetic":{"$ref":"colorAestheticID"}}],"boundsStyle":{"padding":5,"outline":{"a":0.0,"r":0,"b":0,"g":0},"fill":{"a":0.0,"r":0,"b":0,"g":0}}}],"data":[{"id":"dSource","fields":[{"format":{"numericPattern":"###"},"id":"fVariable","label":"Albinism Type","categories":["OCA1B","OCA2","OCA1A"]},{"min":2.0,"max":25.0,"id":"fVariable1","label":"Count"},{"format":{"numericPattern":"###"},"id":"fVariable2","label":"nystagmus","categories":["No","Yes"]}],"rows":[[0,3,0],[1,2,0],[2,17,1],[0,25,1],[1,24,1]]}],"size":{"width":850.0,"height":500.0},"style":{"outline":{"a":0.0,"r":0,"b":0,"g":0},"fill":{"r":255,"b":255,"g":255}},"titles":[{"backgroundStyle":{"outline":{"a":0.0,"r":0,"b":0,"g":0},"fill":{"a":0.0,"r":0,"b":0,"g":0}},"style":{"padding":3.0,"fill":{"r":0,"b":157,"g":100},"font":{"size":"12pt","weight":"bold","family":"sans-serif"}},"type":"title","content":["Bar Chart"]}],"version":"6.0"}

Albinism Type \* Albinism  
Albinism Type \* Albinism - Crosstab - May 11, 2022

CrosstabCrosstab, table, 2 levels of column headers and 3 levels of row headers, table with 5 columns and 19 rows

|  |  |  |  |  |
| --- | --- | --- | --- | --- |
|  | | | Albinism | Total |
|  |  |  | Yes |  |
| Albinism Type | OCA1A | Count | 17 | 17 |
|  |  | % within Albinism Type | 100.0% | 100.0% |
|  |  | % within Albinism | 23.9% | 23.9% |
|  |  | % of Total | 23.9% | 23.9% |
|  | OCA1B | Count | 28 | 28 |
|  |  | % within Albinism Type | 100.0% | 100.0% |
|  |  | % within Albinism | 39.4% | 39.4% |
|  |  | % of Total | 39.4% | 39.4% |
|  | OCA2 | Count | 26 | 26 |
|  |  | % within Albinism Type | 100.0% | 100.0% |
|  |  | % within Albinism | 36.6% | 36.6% |
|  |  | % of Total | 36.6% | 36.6% |
| Total | | Count | 71 | 71 |
|  |  | % within Albinism Type | 100.0% | 100.0% |
|  |  | % within Albinism | 100.0% | 100.0% |
|  |  | % of Total | 100.0% | 100.0% |
|  |  |  |  |  |

Albinism Type \* Albinism  
Albinism Type \* Albinism - Chi-Square Tests - May 11, 2022

Chi-Square TestsChi-Square Tests, table, 1 levels of column headers and 1 levels of row headers, table with 2 columns and 5 rows

|  |  |
| --- | --- |
|  | Value |
| Pearson Chi-Square | .a |
| N of Valid Cases | 71 |
|  |  |
| --- | --- |
| a. No statistics are computed because Albinism is a constant. | |
|  |  |

Albinism Type \* Albinism  
Albinism Type \* Albinism - Bar Chart - May 11, 2022

{"copyright":"(C) Copyright IBM Corp. 2011","grammar":[{"elements":[{"data":{"$ref":"dSource"},"style":{"outline":{"r":0,"b":157,"g":100},"size":"75.0%","width":"90.0%","fill":{"r":0,"b":239,"g":178},"stroke":{"width":0.0}},"position":[{"field":{"$ref":"fVariable1"}},{"field":{"$ref":"fVariable"}}],"type":"interval"}],"coordinates":{"style":{"outline":{"r":0,"b":157,"g":100},"fill":{"r":255,"b":255,"g":255}},"dimensions":[{"scale":{"padding":{"right":"5%"},"spans":[{"outRange":{"min":0.0,"max":1.0}}]},"axis":[{"tickStyle":{"padding":5.0,"fill":{"r":0,"b":157,"g":100},"font":{"size":"8pt","weight":"normal","family":"sans-serif"}},"gridStyle":{"fill":{"r":0,"b":157,"g":100}},"lineStyle":{"fill":{"r":0,"b":157,"g":100},"stroke":{"width":0.6666667}},"titleStyle":{"padding":6.0,"fill":{"r":0,"b":157,"g":100},"font":{"size":"12pt","weight":"bold","family":"sans-serif"}},"title":["Count"],"markStyle":{"fill":{"a":0,"r":0,"b":157,"g":100},"stroke":{"width":1.3333334}}}]},{"scale":{"padding":{"left":"5%","right":"5%"}},"axis":[{"tickStyle":{"padding":5.0,"fill":{"r":0,"b":157,"g":100},"font":{"size":"12pt","weight":"normal","family":"sans-serif"}},"lineStyle":{"fill":{"r":0,"b":157,"g":100},"stroke":{"width":0.6666667}},"titleStyle":{"padding":6.0,"fill":{"r":0,"b":157,"g":100},"font":{"size":"12pt","weight":"bold","family":"sans-serif"}},"title":["Albinism Type"],"markStyle":{"fill":{"a":0,"r":0,"b":157,"g":100},"stroke":{"width":1.3333334}}}]}]}}],"data":[{"id":"dSource","fields":[{"format":{"numericPattern":"###"},"id":"fVariable","label":"Albinism Type","categories":["OCA1A","OCA1B","OCA2"]},{"min":17.0,"max":28.0,"id":"fVariable1","label":"Count"},{"format":{"numericPattern":"###"},"id":"fVariable2","label":"Albinism","categories":["Yes"]}],"rows":[[0,17,0],[1,28,0],[2,26,0]]}],"size":{"width":850.0,"height":500.0},"style":{"outline":{"a":0.0,"r":0,"b":0,"g":0},"fill":{"r":255,"b":255,"g":255}},"titles":[{"backgroundStyle":{"outline":{"a":0.0,"r":0,"b":0,"g":0},"fill":{"a":0.0,"r":0,"b":0,"g":0}},"style":{"padding":3.0,"fill":{"r":0,"b":157,"g":100},"font":{"size":"12pt","weight":"bold","family":"sans-serif"}},"type":"title","content":["Bar Chart"]}],"version":"6.0"}

Albinism Type \* Eye muscle surgery  
Albinism Type \* Eye muscle surgery - Crosstab - May 11, 2022

CrosstabCrosstab, table, 2 levels of column headers and 3 levels of row headers, table with 6 columns and 19 rows

|  |  |  |  |  |  |
| --- | --- | --- | --- | --- | --- |
|  | | | Eye muscle surgery | | Total |
|  |  |  | No | Yes |  |
| Albinism Type | OCA1A | Count | 5 | 12 | 17 |
|  |  | % within Albinism Type | 29.4% | 70.6% | 100.0% |
|  |  | % within Eye muscle surgery | 13.9% | 34.3% | 23.9% |
|  |  | % of Total | 7.0% | 16.9% | 23.9% |
|  | OCA1B | Count | 17 | 11 | 28 |
|  |  | % within Albinism Type | 60.7% | 39.3% | 100.0% |
|  |  | % within Eye muscle surgery | 47.2% | 31.4% | 39.4% |
|  |  | % of Total | 23.9% | 15.5% | 39.4% |
|  | OCA2 | Count | 14 | 12 | 26 |
|  |  | % within Albinism Type | 53.8% | 46.2% | 100.0% |
|  |  | % within Eye muscle surgery | 38.9% | 34.3% | 36.6% |
|  |  | % of Total | 19.7% | 16.9% | 36.6% |
| Total | | Count | 36 | 35 | 71 |
|  |  | % within Albinism Type | 50.7% | 49.3% | 100.0% |
|  |  | % within Eye muscle surgery | 100.0% | 100.0% | 100.0% |
|  |  | % of Total | 50.7% | 49.3% | 100.0% |
|  |  |  |  |  |  |

Albinism Type \* Eye muscle surgery  
Albinism Type \* Eye muscle surgery - Chi-Square Tests - May 11, 2022

Chi-Square TestsChi-Square Tests, table, 1 levels of column headers and 1 levels of row headers, table with 4 columns and 7 rows

|  |  |  |  |
| --- | --- | --- | --- |
|  | Value | df | Asymptotic Significance (2-sided) |
| Pearson Chi-Square | 4.309a | 2 | .116 |
| Likelihood Ratio | 4.406 | 2 | .110 |
| Linear-by-Linear Association | 1.855 | 1 | .173 |
| N of Valid Cases | 71 |  |  |
|  |  |  |  |
| --- | --- | --- | --- |
| a. 0 cells (.0%) have expected count less than 5. The minimum expected count is 8.38. | | | |
|  |  |  |  |

Albinism Type \* Eye muscle surgery  
Albinism Type \* Eye muscle surgery - Bar Chart - May 11, 2022

{"copyright":"(C) Copyright IBM Corp. 2011","grammar":[{"elements":[{"color":[{"field":{"$ref":"fVariable2"},"palette":["#00B2EF","#D9182D","#FFCF10","#7F1C7D","#007670","#DD731C","#17AF4B","#EE3D96","#838329","#82D1F5","#F04E37","#FFE14F","#AB1A86","#00A6A0","#F19027","#8CC63F","#F389AF","#A5A215","#00649D","#A91024","#FDB813","#3B2056","#006058","#B8471B","#00BA52","#BA006E","#594F13"],"id":"colorAestheticID"}],"data":{"$ref":"dSource"},"style":{"outline":{"r":0,"b":0,"g":0},"size":"100.0%","width":"90.0%","stroke":{"width":0.0}},"position":[{"field":{"$ref":"fVariable1"}},{"field":{"$ref":"fVariable"}},{"field":{"$ref":"fVariable2"}}],"type":"interval"}],"coordinates":{"transforms":[{"type":"cluster"}],"style":{"outline":{"r":0,"b":157,"g":100},"fill":{"r":255,"b":255,"g":255}},"dimensions":[{"scale":{"padding":{"right":"5%"},"spans":[{"outRange":{"min":0.0,"max":1.0}}]},"axis":[{"tickStyle":{"padding":5.0,"fill":{"r":0,"b":157,"g":100},"font":{"size":"8pt","weight":"normal","family":"sans-serif"}},"gridStyle":{"fill":{"r":0,"b":157,"g":100}},"lineStyle":{"fill":{"r":0,"b":157,"g":100},"stroke":{"width":0.6666667}},"titleStyle":{"padding":6.0,"fill":{"r":0,"b":157,"g":100},"font":{"size":"12pt","weight":"bold","family":"sans-serif"}},"title":["Count"],"markStyle":{"fill":{"a":0,"r":0,"b":157,"g":100},"stroke":{"width":1.3333334}}}]},{"scale":{"categoryStructure":{"gap":"15%","nest":true},"local":false},"axis":[{"tickStyle":{"padding":5.0,"fill":{"r":0,"b":157,"g":100},"font":{"size":"12pt","weight":"normal","family":"sans-serif"}},"lineStyle":{"fill":{"r":0,"b":157,"g":100},"stroke":{"width":0.6666667}},"titleStyle":{"padding":6.0,"fill":{"r":0,"b":157,"g":100},"font":{"size":"12pt","weight":"bold","family":"sans-serif"}},"title":["Albinism Type"],"markStyle":{"fill":{"a":0,"r":0,"b":157,"g":100},"stroke":{"width":1.3333334}}}]},{}]}}],"legends":[{"labelStyle":{"fill":{"r":0,"b":157,"g":100},"font":{"size":"10pt","weight":"normal","family":"sans-serif"}},"itemStyle":{"padding":3,"size":11},"titleStyle":{"padding":3.0,"fill":{"r":0,"b":157,"g":100},"align":"start","font":{"size":"12pt","weight":"bold","family":"sans-serif"}},"location":{"width":"24%"},"title":["Eye muscle surgery"],"aesthetics":[{"aesthetic":{"$ref":"colorAestheticID"}}],"boundsStyle":{"padding":5,"outline":{"a":0.0,"r":0,"b":0,"g":0},"fill":{"a":0.0,"r":0,"b":0,"g":0}}}],"data":[{"id":"dSource","fields":[{"format":{"numericPattern":"###"},"id":"fVariable","label":"Albinism Type","categories":["OCA1A","OCA1B","OCA2"]},{"min":5.0,"max":17.0,"id":"fVariable1","label":"Count"},{"format":{"numericPattern":"###"},"id":"fVariable2","label":"Eye muscle surgery","categories":["No","Yes"]}],"rows":[[0,5,0],[1,17,0],[2,14,0],[0,12,1],[1,11,1],[2,12,1]]}],"size":{"width":850.0,"height":500.0},"style":{"outline":{"a":0.0,"r":0,"b":0,"g":0},"fill":{"r":255,"b":255,"g":255}},"titles":[{"backgroundStyle":{"outline":{"a":0.0,"r":0,"b":0,"g":0},"fill":{"a":0.0,"r":0,"b":0,"g":0}},"style":{"padding":3.0,"fill":{"r":0,"b":157,"g":100},"font":{"size":"12pt","weight":"bold","family":"sans-serif"}},"type":"title","content":["Bar Chart"]}],"version":"6.0"}

Albinism Type \* Srabismus  
Albinism Type \* Srabismus - Crosstab - May 11, 2022

CrosstabCrosstab, table, 2 levels of column headers and 3 levels of row headers, table with 7 columns and 19 rows

|  |  |  |  |  |  |  |
| --- | --- | --- | --- | --- | --- | --- |
|  | | | Srabismus | | | Total |
|  |  |  | None | Iso | Exo |  |
| Albinism Type | OCA1A | Count | 6 | 8 | 3 | 17 |
|  |  | % within Albinism Type | 35.3% | 47.1% | 17.6% | 100.0% |
|  |  | % within Srabismus | 27.3% | 33.3% | 12.5% | 24.3% |
|  |  | % of Total | 8.6% | 11.4% | 4.3% | 24.3% |
|  | OCA1B | Count | 8 | 9 | 10 | 27 |
|  |  | % within Albinism Type | 29.6% | 33.3% | 37.0% | 100.0% |
|  |  | % within Srabismus | 36.4% | 37.5% | 41.7% | 38.6% |
|  |  | % of Total | 11.4% | 12.9% | 14.3% | 38.6% |
|  | OCA2 | Count | 8 | 7 | 11 | 26 |
|  |  | % within Albinism Type | 30.8% | 26.9% | 42.3% | 100.0% |
|  |  | % within Srabismus | 36.4% | 29.2% | 45.8% | 37.1% |
|  |  | % of Total | 11.4% | 10.0% | 15.7% | 37.1% |
| Total | | Count | 22 | 24 | 24 | 70 |
|  |  | % within Albinism Type | 31.4% | 34.3% | 34.3% | 100.0% |
|  |  | % within Srabismus | 100.0% | 100.0% | 100.0% | 100.0% |
|  |  | % of Total | 31.4% | 34.3% | 34.3% | 100.0% |
|  |  |  |  |  |  |  |

Albinism Type \* Srabismus  
Albinism Type \* Srabismus - Chi-Square Tests - May 11, 2022

Chi-Square TestsChi-Square Tests, table, 1 levels of column headers and 1 levels of row headers, table with 4 columns and 7 rows

|  |  |  |  |
| --- | --- | --- | --- |
|  | Value | df | Asymptotic Significance (2-sided) |
| Pearson Chi-Square | 3.260a | 4 | .515 |
| Likelihood Ratio | 3.469 | 4 | .483 |
| Linear-by-Linear Association | 1.184 | 1 | .277 |
| N of Valid Cases | 70 |  |  |
|  |  |  |  |
| --- | --- | --- | --- |
| a. 0 cells (.0%) have expected count less than 5. The minimum expected count is 5.34. | | | |
|  |  |  |  |

Albinism Type \* Srabismus  
Albinism Type \* Srabismus - Bar Chart - May 11, 2022

{"copyright":"(C) Copyright IBM Corp. 2011","grammar":[{"elements":[{"color":[{"field":{"$ref":"fVariable2"},"palette":["#00B2EF","#D9182D","#FFCF10","#7F1C7D","#007670","#DD731C","#17AF4B","#EE3D96","#838329","#82D1F5","#F04E37","#FFE14F","#AB1A86","#00A6A0","#F19027","#8CC63F","#F389AF","#A5A215","#00649D","#A91024","#FDB813","#3B2056","#006058","#B8471B","#00BA52","#BA006E","#594F13"],"id":"colorAestheticID"}],"data":{"$ref":"dSource"},"style":{"outline":{"r":0,"b":0,"g":0},"size":"100.0%","width":"90.0%","stroke":{"width":0.0}},"position":[{"field":{"$ref":"fVariable1"}},{"field":{"$ref":"fVariable"}},{"field":{"$ref":"fVariable2"}}],"type":"interval"}],"coordinates":{"transforms":[{"type":"cluster"}],"style":{"outline":{"r":0,"b":157,"g":100},"fill":{"r":255,"b":255,"g":255}},"dimensions":[{"scale":{"padding":{"right":"5%"},"spans":[{"outRange":{"min":0.0,"max":1.0}}]},"axis":[{"tickStyle":{"padding":5.0,"fill":{"r":0,"b":157,"g":100},"font":{"size":"8pt","weight":"normal","family":"sans-serif"}},"gridStyle":{"fill":{"r":0,"b":157,"g":100}},"lineStyle":{"fill":{"r":0,"b":157,"g":100},"stroke":{"width":0.6666667}},"titleStyle":{"padding":6.0,"fill":{"r":0,"b":157,"g":100},"font":{"size":"12pt","weight":"bold","family":"sans-serif"}},"title":["Count"],"markStyle":{"fill":{"a":0,"r":0,"b":157,"g":100},"stroke":{"width":1.3333334}}}]},{"scale":{"categoryStructure":{"gap":"15%","nest":true},"local":false},"axis":[{"tickStyle":{"padding":5.0,"fill":{"r":0,"b":157,"g":100},"font":{"size":"12pt","weight":"normal","family":"sans-serif"}},"lineStyle":{"fill":{"r":0,"b":157,"g":100},"stroke":{"width":0.6666667}},"titleStyle":{"padding":6.0,"fill":{"r":0,"b":157,"g":100},"font":{"size":"12pt","weight":"bold","family":"sans-serif"}},"title":["Albinism Type"],"markStyle":{"fill":{"a":0,"r":0,"b":157,"g":100},"stroke":{"width":1.3333334}}}]},{}]}}],"legends":[{"labelStyle":{"fill":{"r":0,"b":157,"g":100},"font":{"size":"10pt","weight":"normal","family":"sans-serif"}},"itemStyle":{"padding":3,"size":11},"titleStyle":{"padding":3.0,"fill":{"r":0,"b":157,"g":100},"align":"start","font":{"size":"12pt","weight":"bold","family":"sans-serif"}},"location":{"width":"24%"},"title":["Srabismus"],"aesthetics":[{"aesthetic":{"$ref":"colorAestheticID"}}],"boundsStyle":{"padding":5,"outline":{"a":0.0,"r":0,"b":0,"g":0},"fill":{"a":0.0,"r":0,"b":0,"g":0}}}],"data":[{"id":"dSource","fields":[{"format":{"numericPattern":"###"},"id":"fVariable","label":"Albinism Type","categories":["OCA1A","OCA1B","OCA2"]},{"min":3.0,"max":11.0,"id":"fVariable1","label":"Count"},{"format":{"numericPattern":"###"},"id":"fVariable2","label":"Srabismus","categories":["None","Iso","Exo"]}],"rows":[[0,6,0],[1,8,0],[2,8,0],[0,8,1],[1,9,1],[2,7,1],[0,3,2],[1,10,2],[2,11,2]]}],"size":{"width":850.0,"height":500.0},"style":{"outline":{"a":0.0,"r":0,"b":0,"g":0},"fill":{"r":255,"b":255,"g":255}},"titles":[{"backgroundStyle":{"outline":{"a":0.0,"r":0,"b":0,"g":0},"fill":{"a":0.0,"r":0,"b":0,"g":0}},"style":{"padding":3.0,"fill":{"r":0,"b":157,"g":100},"font":{"size":"12pt","weight":"bold","family":"sans-serif"}},"type":"title","content":["Bar Chart"]}],"version":"6.0"}

Albinism Type \* RE\_Myopia  
Albinism Type \* RE\_Myopia - Crosstab - May 11, 2022

CrosstabCrosstab, table, 2 levels of column headers and 3 levels of row headers, table with 6 columns and 19 rows

|  |  |  |  |  |  |
| --- | --- | --- | --- | --- | --- |
|  | | | RE\_Myopia | | Total |
|  |  |  | Hyperm | Myopia |  |
| Albinism Type | OCA1A | Count | 13 | 4 | 17 |
|  |  | % within Albinism Type | 76.5% | 23.5% | 100.0% |
|  |  | % within RE\_Myopia | 23.6% | 25.0% | 23.9% |
|  |  | % of Total | 18.3% | 5.6% | 23.9% |
|  | OCA1B | Count | 23 | 5 | 28 |
|  |  | % within Albinism Type | 82.1% | 17.9% | 100.0% |
|  |  | % within RE\_Myopia | 41.8% | 31.3% | 39.4% |
|  |  | % of Total | 32.4% | 7.0% | 39.4% |
|  | OCA2 | Count | 19 | 7 | 26 |
|  |  | % within Albinism Type | 73.1% | 26.9% | 100.0% |
|  |  | % within RE\_Myopia | 34.5% | 43.8% | 36.6% |
|  |  | % of Total | 26.8% | 9.9% | 36.6% |
| Total | | Count | 55 | 16 | 71 |
|  |  | % within Albinism Type | 77.5% | 22.5% | 100.0% |
|  |  | % within RE\_Myopia | 100.0% | 100.0% | 100.0% |
|  |  | % of Total | 77.5% | 22.5% | 100.0% |
|  |  |  |  |  |  |

Albinism Type \* RE\_Myopia  
Albinism Type \* RE\_Myopia - Chi-Square Tests - May 11, 2022

Chi-Square TestsChi-Square Tests, table, 1 levels of column headers and 1 levels of row headers, table with 4 columns and 7 rows

|  |  |  |  |
| --- | --- | --- | --- |
|  | Value | df | Asymptotic Significance (2-sided) |
| Pearson Chi-Square | .647a | 2 | .723 |
| Likelihood Ratio | .655 | 2 | .721 |
| Linear-by-Linear Association | .127 | 1 | .721 |
| N of Valid Cases | 71 |  |  |
|  |  |  |  |
| --- | --- | --- | --- |
| a. 1 cells (16.7%) have expected count less than 5. The minimum expected count is 3.83. | | | |
|  |  |  |  |

Albinism Type \* RE\_Myopia  
Albinism Type \* RE\_Myopia - Bar Chart - May 11, 2022

{"copyright":"(C) Copyright IBM Corp. 2011","grammar":[{"elements":[{"color":[{"field":{"$ref":"fVariable2"},"palette":["#00B2EF","#D9182D","#FFCF10","#7F1C7D","#007670","#DD731C","#17AF4B","#EE3D96","#838329","#82D1F5","#F04E37","#FFE14F","#AB1A86","#00A6A0","#F19027","#8CC63F","#F389AF","#A5A215","#00649D","#A91024","#FDB813","#3B2056","#006058","#B8471B","#00BA52","#BA006E","#594F13"],"id":"colorAestheticID"}],"data":{"$ref":"dSource"},"style":{"outline":{"r":0,"b":0,"g":0},"size":"100.0%","width":"90.0%","stroke":{"width":0.0}},"position":[{"field":{"$ref":"fVariable1"}},{"field":{"$ref":"fVariable"}},{"field":{"$ref":"fVariable2"}}],"type":"interval"}],"coordinates":{"transforms":[{"type":"cluster"}],"style":{"outline":{"r":0,"b":157,"g":100},"fill":{"r":255,"b":255,"g":255}},"dimensions":[{"scale":{"padding":{"right":"5%"},"spans":[{"outRange":{"min":0.0,"max":1.0}}]},"axis":[{"tickStyle":{"padding":5.0,"fill":{"r":0,"b":157,"g":100},"font":{"size":"8pt","weight":"normal","family":"sans-serif"}},"gridStyle":{"fill":{"r":0,"b":157,"g":100}},"lineStyle":{"fill":{"r":0,"b":157,"g":100},"stroke":{"width":0.6666667}},"titleStyle":{"padding":6.0,"fill":{"r":0,"b":157,"g":100},"font":{"size":"12pt","weight":"bold","family":"sans-serif"}},"title":["Count"],"markStyle":{"fill":{"a":0,"r":0,"b":157,"g":100},"stroke":{"width":1.3333334}}}]},{"scale":{"categoryStructure":{"gap":"15%","nest":true},"local":false},"axis":[{"tickStyle":{"padding":5.0,"fill":{"r":0,"b":157,"g":100},"font":{"size":"12pt","weight":"normal","family":"sans-serif"}},"lineStyle":{"fill":{"r":0,"b":157,"g":100},"stroke":{"width":0.6666667}},"titleStyle":{"padding":6.0,"fill":{"r":0,"b":157,"g":100},"font":{"size":"12pt","weight":"bold","family":"sans-serif"}},"title":["Albinism Type"],"markStyle":{"fill":{"a":0,"r":0,"b":157,"g":100},"stroke":{"width":1.3333334}}}]},{}]}}],"legends":[{"labelStyle":{"fill":{"r":0,"b":157,"g":100},"font":{"size":"10pt","weight":"normal","family":"sans-serif"}},"itemStyle":{"padding":3,"size":11},"titleStyle":{"padding":3.0,"fill":{"r":0,"b":157,"g":100},"align":"start","font":{"size":"12pt","weight":"bold","family":"sans-serif"}},"location":{"width":"24%"},"title":["RE\_Myopia"],"aesthetics":[{"aesthetic":{"$ref":"colorAestheticID"}}],"boundsStyle":{"padding":5,"outline":{"a":0.0,"r":0,"b":0,"g":0},"fill":{"a":0.0,"r":0,"b":0,"g":0}}}],"data":[{"id":"dSource","fields":[{"format":{"numericPattern":"###"},"id":"fVariable","label":"Albinism Type","categories":["OCA1A","OCA1B","OCA2"]},{"min":4.0,"max":23.0,"id":"fVariable1","label":"Count"},{"format":{"numericPattern":"###"},"id":"fVariable2","label":"RE\_Myopia","categories":["Hyperm","Myopia"]}],"rows":[[0,13,0],[1,23,0],[2,19,0],[0,4,1],[1,5,1],[2,7,1]]}],"size":{"width":850.0,"height":500.0},"style":{"outline":{"a":0.0,"r":0,"b":0,"g":0},"fill":{"r":255,"b":255,"g":255}},"titles":[{"backgroundStyle":{"outline":{"a":0.0,"r":0,"b":0,"g":0},"fill":{"a":0.0,"r":0,"b":0,"g":0}},"style":{"padding":3.0,"fill":{"r":0,"b":157,"g":100},"font":{"size":"12pt","weight":"bold","family":"sans-serif"}},"type":"title","content":["Bar Chart"]}],"version":"6.0"}

Albinism Type \* RE\_ref error  
Albinism Type \* RE\_ref error - Crosstab - May 11, 2022

CrosstabCrosstab, table, 2 levels of column headers and 3 levels of row headers, table with 6 columns and 19 rows

|  |  |  |  |  |  |
| --- | --- | --- | --- | --- | --- |
|  | | | RE\_ref error | | Total |
|  |  |  | No | Yes |  |
| Albinism Type | OCA1A | Count | 2 | 15 | 17 |
|  |  | % within Albinism Type | 11.8% | 88.2% | 100.0% |
|  |  | % within RE\_ref error | 10.5% | 28.8% | 23.9% |
|  |  | % of Total | 2.8% | 21.1% | 23.9% |
|  | OCA1B | Count | 10 | 18 | 28 |
|  |  | % within Albinism Type | 35.7% | 64.3% | 100.0% |
|  |  | % within RE\_ref error | 52.6% | 34.6% | 39.4% |
|  |  | % of Total | 14.1% | 25.4% | 39.4% |
|  | OCA2 | Count | 7 | 19 | 26 |
|  |  | % within Albinism Type | 26.9% | 73.1% | 100.0% |
|  |  | % within RE\_ref error | 36.8% | 36.5% | 36.6% |
|  |  | % of Total | 9.9% | 26.8% | 36.6% |
| Total | | Count | 19 | 52 | 71 |
|  |  | % within Albinism Type | 26.8% | 73.2% | 100.0% |
|  |  | % within RE\_ref error | 100.0% | 100.0% | 100.0% |
|  |  | % of Total | 26.8% | 73.2% | 100.0% |
|  |  |  |  |  |  |

Albinism Type \* RE\_ref error  
Albinism Type \* RE\_ref error - Chi-Square Tests - May 11, 2022

Chi-Square TestsChi-Square Tests, table, 1 levels of column headers and 1 levels of row headers, table with 4 columns and 7 rows

|  |  |  |  |
| --- | --- | --- | --- |
|  | Value | df | Asymptotic Significance (2-sided) |
| Pearson Chi-Square | 3.096a | 2 | .213 |
| Likelihood Ratio | 3.379 | 2 | .185 |
| Linear-by-Linear Association | .807 | 1 | .369 |
| N of Valid Cases | 71 |  |  |
|  |  |  |  |
| --- | --- | --- | --- |
| a. 1 cells (16.7%) have expected count less than 5. The minimum expected count is 4.55. | | | |
|  |  |  |  |

Albinism Type \* RE\_ref error  
Albinism Type \* RE\_ref error - Bar Chart - May 11, 2022

{"copyright":"(C) Copyright IBM Corp. 2011","grammar":[{"elements":[{"color":[{"field":{"$ref":"fVariable2"},"palette":["#00B2EF","#D9182D","#FFCF10","#7F1C7D","#007670","#DD731C","#17AF4B","#EE3D96","#838329","#82D1F5","#F04E37","#FFE14F","#AB1A86","#00A6A0","#F19027","#8CC63F","#F389AF","#A5A215","#00649D","#A91024","#FDB813","#3B2056","#006058","#B8471B","#00BA52","#BA006E","#594F13"],"id":"colorAestheticID"}],"data":{"$ref":"dSource"},"style":{"outline":{"r":0,"b":0,"g":0},"size":"100.0%","width":"90.0%","stroke":{"width":0.0}},"position":[{"field":{"$ref":"fVariable1"}},{"field":{"$ref":"fVariable"}},{"field":{"$ref":"fVariable2"}}],"type":"interval"}],"coordinates":{"transforms":[{"type":"cluster"}],"style":{"outline":{"r":0,"b":157,"g":100},"fill":{"r":255,"b":255,"g":255}},"dimensions":[{"scale":{"padding":{"right":"5%"},"spans":[{"outRange":{"min":0.0,"max":1.0}}]},"axis":[{"tickStyle":{"padding":5.0,"fill":{"r":0,"b":157,"g":100},"font":{"size":"8pt","weight":"normal","family":"sans-serif"}},"gridStyle":{"fill":{"r":0,"b":157,"g":100}},"lineStyle":{"fill":{"r":0,"b":157,"g":100},"stroke":{"width":0.6666667}},"titleStyle":{"padding":6.0,"fill":{"r":0,"b":157,"g":100},"font":{"size":"12pt","weight":"bold","family":"sans-serif"}},"title":["Count"],"markStyle":{"fill":{"a":0,"r":0,"b":157,"g":100},"stroke":{"width":1.3333334}}}]},{"scale":{"categoryStructure":{"gap":"15%","nest":true},"local":false},"axis":[{"tickStyle":{"padding":5.0,"fill":{"r":0,"b":157,"g":100},"font":{"size":"12pt","weight":"normal","family":"sans-serif"}},"lineStyle":{"fill":{"r":0,"b":157,"g":100},"stroke":{"width":0.6666667}},"titleStyle":{"padding":6.0,"fill":{"r":0,"b":157,"g":100},"font":{"size":"12pt","weight":"bold","family":"sans-serif"}},"title":["Albinism Type"],"markStyle":{"fill":{"a":0,"r":0,"b":157,"g":100},"stroke":{"width":1.3333334}}}]},{}]}}],"legends":[{"labelStyle":{"fill":{"r":0,"b":157,"g":100},"font":{"size":"10pt","weight":"normal","family":"sans-serif"}},"itemStyle":{"padding":3,"size":11},"titleStyle":{"padding":3.0,"fill":{"r":0,"b":157,"g":100},"align":"start","font":{"size":"12pt","weight":"bold","family":"sans-serif"}},"location":{"width":"24%"},"title":["RE\_ref error"],"aesthetics":[{"aesthetic":{"$ref":"colorAestheticID"}}],"boundsStyle":{"padding":5,"outline":{"a":0.0,"r":0,"b":0,"g":0},"fill":{"a":0.0,"r":0,"b":0,"g":0}}}],"data":[{"id":"dSource","fields":[{"format":{"numericPattern":"###"},"id":"fVariable","label":"Albinism Type","categories":["OCA1A","OCA1B","OCA2"]},{"min":2.0,"max":19.0,"id":"fVariable1","label":"Count"},{"format":{"numericPattern":"###"},"id":"fVariable2","label":"RE\_ref error","categories":["No","Yes"]}],"rows":[[0,2,0],[1,10,0],[2,7,0],[0,15,1],[1,18,1],[2,19,1]]}],"size":{"width":850.0,"height":500.0},"style":{"outline":{"a":0.0,"r":0,"b":0,"g":0},"fill":{"r":255,"b":255,"g":255}},"titles":[{"backgroundStyle":{"outline":{"a":0.0,"r":0,"b":0,"g":0},"fill":{"a":0.0,"r":0,"b":0,"g":0}},"style":{"padding":3.0,"fill":{"r":0,"b":157,"g":100},"font":{"size":"12pt","weight":"bold","family":"sans-serif"}},"type":"title","content":["Bar Chart"]}],"version":"6.0"}

Albinism Type \* RE\_cyl  
Albinism Type \* RE\_cyl - Crosstab - May 11, 2022

CrosstabCrosstab, table, 2 levels of column headers and 3 levels of row headers, table with 6 columns and 19 rows

|  |  |  |  |  |  |
| --- | --- | --- | --- | --- | --- |
|  | | | RE\_cyl | | Total |
|  |  |  | No | Yes |  |
| Albinism Type | OCA1A | Count | 1 | 16 | 17 |
|  |  | % within Albinism Type | 5.9% | 94.1% | 100.0% |
|  |  | % within RE\_cyl | 7.1% | 28.1% | 23.9% |
|  |  | % of Total | 1.4% | 22.5% | 23.9% |
|  | OCA1B | Count | 7 | 21 | 28 |
|  |  | % within Albinism Type | 25.0% | 75.0% | 100.0% |
|  |  | % within RE\_cyl | 50.0% | 36.8% | 39.4% |
|  |  | % of Total | 9.9% | 29.6% | 39.4% |
|  | OCA2 | Count | 6 | 20 | 26 |
|  |  | % within Albinism Type | 23.1% | 76.9% | 100.0% |
|  |  | % within RE\_cyl | 42.9% | 35.1% | 36.6% |
|  |  | % of Total | 8.5% | 28.2% | 36.6% |
| Total | | Count | 14 | 57 | 71 |
|  |  | % within Albinism Type | 19.7% | 80.3% | 100.0% |
|  |  | % within RE\_cyl | 100.0% | 100.0% | 100.0% |
|  |  | % of Total | 19.7% | 80.3% | 100.0% |
|  |  |  |  |  |  |

Albinism Type \* RE\_cyl  
Albinism Type \* RE\_cyl - Chi-Square Tests - May 11, 2022

Chi-Square TestsChi-Square Tests, table, 1 levels of column headers and 1 levels of row headers, table with 4 columns and 7 rows

|  |  |  |  |
| --- | --- | --- | --- |
|  | Value | df | Asymptotic Significance (2-sided) |
| Pearson Chi-Square | 2.734a | 2 | .255 |
| Likelihood Ratio | 3.311 | 2 | .191 |
| Linear-by-Linear Association | 1.548 | 1 | .213 |
| N of Valid Cases | 71 |  |  |
|  |  |  |  |
| --- | --- | --- | --- |
| a. 1 cells (16.7%) have expected count less than 5. The minimum expected count is 3.35. | | | |
|  |  |  |  |

Albinism Type \* RE\_cyl  
Albinism Type \* RE\_cyl - Bar Chart - May 11, 2022

{"copyright":"(C) Copyright IBM Corp. 2011","grammar":[{"elements":[{"color":[{"field":{"$ref":"fVariable2"},"palette":["#00B2EF","#D9182D","#FFCF10","#7F1C7D","#007670","#DD731C","#17AF4B","#EE3D96","#838329","#82D1F5","#F04E37","#FFE14F","#AB1A86","#00A6A0","#F19027","#8CC63F","#F389AF","#A5A215","#00649D","#A91024","#FDB813","#3B2056","#006058","#B8471B","#00BA52","#BA006E","#594F13"],"id":"colorAestheticID"}],"data":{"$ref":"dSource"},"style":{"outline":{"r":0,"b":0,"g":0},"size":"100.0%","width":"90.0%","stroke":{"width":0.0}},"position":[{"field":{"$ref":"fVariable1"}},{"field":{"$ref":"fVariable"}},{"field":{"$ref":"fVariable2"}}],"type":"interval"}],"coordinates":{"transforms":[{"type":"cluster"}],"style":{"outline":{"r":0,"b":157,"g":100},"fill":{"r":255,"b":255,"g":255}},"dimensions":[{"scale":{"padding":{"right":"5%"},"spans":[{"outRange":{"min":0.0,"max":1.0}}]},"axis":[{"tickStyle":{"padding":5.0,"fill":{"r":0,"b":157,"g":100},"font":{"size":"8pt","weight":"normal","family":"sans-serif"}},"gridStyle":{"fill":{"r":0,"b":157,"g":100}},"lineStyle":{"fill":{"r":0,"b":157,"g":100},"stroke":{"width":0.6666667}},"titleStyle":{"padding":6.0,"fill":{"r":0,"b":157,"g":100},"font":{"size":"12pt","weight":"bold","family":"sans-serif"}},"title":["Count"],"markStyle":{"fill":{"a":0,"r":0,"b":157,"g":100},"stroke":{"width":1.3333334}}}]},{"scale":{"categoryStructure":{"gap":"15%","nest":true},"local":false},"axis":[{"tickStyle":{"padding":5.0,"fill":{"r":0,"b":157,"g":100},"font":{"size":"12pt","weight":"normal","family":"sans-serif"}},"lineStyle":{"fill":{"r":0,"b":157,"g":100},"stroke":{"width":0.6666667}},"titleStyle":{"padding":6.0,"fill":{"r":0,"b":157,"g":100},"font":{"size":"12pt","weight":"bold","family":"sans-serif"}},"title":["Albinism Type"],"markStyle":{"fill":{"a":0,"r":0,"b":157,"g":100},"stroke":{"width":1.3333334}}}]},{}]}}],"legends":[{"labelStyle":{"fill":{"r":0,"b":157,"g":100},"font":{"size":"10pt","weight":"normal","family":"sans-serif"}},"itemStyle":{"padding":3,"size":11},"titleStyle":{"padding":3.0,"fill":{"r":0,"b":157,"g":100},"align":"start","font":{"size":"12pt","weight":"bold","family":"sans-serif"}},"location":{"width":"24%"},"title":["RE\_cyl"],"aesthetics":[{"aesthetic":{"$ref":"colorAestheticID"}}],"boundsStyle":{"padding":5,"outline":{"a":0.0,"r":0,"b":0,"g":0},"fill":{"a":0.0,"r":0,"b":0,"g":0}}}],"data":[{"id":"dSource","fields":[{"format":{"numericPattern":"###"},"id":"fVariable","label":"Albinism Type","categories":["OCA1A","OCA1B","OCA2"]},{"min":1.0,"max":21.0,"id":"fVariable1","label":"Count"},{"format":{"numericPattern":"###"},"id":"fVariable2","label":"RE\_cyl","categories":["No","Yes"]}],"rows":[[0,1,0],[1,7,0],[2,6,0],[0,16,1],[1,21,1],[2,20,1]]}],"size":{"width":850.0,"height":500.0},"style":{"outline":{"a":0.0,"r":0,"b":0,"g":0},"fill":{"r":255,"b":255,"g":255}},"titles":[{"backgroundStyle":{"outline":{"a":0.0,"r":0,"b":0,"g":0},"fill":{"a":0.0,"r":0,"b":0,"g":0}},"style":{"padding":3.0,"fill":{"r":0,"b":157,"g":100},"font":{"size":"12pt","weight":"bold","family":"sans-serif"}},"type":"title","content":["Bar Chart"]}],"version":"6.0"}

Albinism Type \* LE\_Myopia  
Albinism Type \* LE\_Myopia - Crosstab - May 11, 2022

CrosstabCrosstab, table, 2 levels of column headers and 3 levels of row headers, table with 6 columns and 19 rows

|  |  |  |  |  |  |
| --- | --- | --- | --- | --- | --- |
|  | | | LE\_Myopia | | Total |
|  |  |  | Hyperm | Myopia |  |
| Albinism Type | OCA1A | Count | 13 | 4 | 17 |
|  |  | % within Albinism Type | 76.5% | 23.5% | 100.0% |
|  |  | % within LE\_Myopia | 23.6% | 26.7% | 24.3% |
|  |  | % of Total | 18.6% | 5.7% | 24.3% |
|  | OCA1B | Count | 22 | 5 | 27 |
|  |  | % within Albinism Type | 81.5% | 18.5% | 100.0% |
|  |  | % within LE\_Myopia | 40.0% | 33.3% | 38.6% |
|  |  | % of Total | 31.4% | 7.1% | 38.6% |
|  | OCA2 | Count | 20 | 6 | 26 |
|  |  | % within Albinism Type | 76.9% | 23.1% | 100.0% |
|  |  | % within LE\_Myopia | 36.4% | 40.0% | 37.1% |
|  |  | % of Total | 28.6% | 8.6% | 37.1% |
| Total | | Count | 55 | 15 | 70 |
|  |  | % within Albinism Type | 78.6% | 21.4% | 100.0% |
|  |  | % within LE\_Myopia | 100.0% | 100.0% | 100.0% |
|  |  | % of Total | 78.6% | 21.4% | 100.0% |
|  |  |  |  |  |  |

Albinism Type \* LE\_Myopia  
Albinism Type \* LE\_Myopia - Chi-Square Tests - May 11, 2022

Chi-Square TestsChi-Square Tests, table, 1 levels of column headers and 1 levels of row headers, table with 4 columns and 7 rows

|  |  |  |  |
| --- | --- | --- | --- |
|  | Value | df | Asymptotic Significance (2-sided) |
| Pearson Chi-Square | .222a | 2 | .895 |
| Likelihood Ratio | .225 | 2 | .893 |
| Linear-by-Linear Association | .001 | 1 | .979 |
| N of Valid Cases | 70 |  |  |
|  |  |  |  |
| --- | --- | --- | --- |
| a. 1 cells (16.7%) have expected count less than 5. The minimum expected count is 3.64. | | | |
|  |  |  |  |

Albinism Type \* LE\_Myopia  
Albinism Type \* LE\_Myopia - Bar Chart - May 11, 2022

{"copyright":"(C) Copyright IBM Corp. 2011","grammar":[{"elements":[{"color":[{"field":{"$ref":"fVariable2"},"palette":["#00B2EF","#D9182D","#FFCF10","#7F1C7D","#007670","#DD731C","#17AF4B","#EE3D96","#838329","#82D1F5","#F04E37","#FFE14F","#AB1A86","#00A6A0","#F19027","#8CC63F","#F389AF","#A5A215","#00649D","#A91024","#FDB813","#3B2056","#006058","#B8471B","#00BA52","#BA006E","#594F13"],"id":"colorAestheticID"}],"data":{"$ref":"dSource"},"style":{"outline":{"r":0,"b":0,"g":0},"size":"100.0%","width":"90.0%","stroke":{"width":0.0}},"position":[{"field":{"$ref":"fVariable1"}},{"field":{"$ref":"fVariable"}},{"field":{"$ref":"fVariable2"}}],"type":"interval"}],"coordinates":{"transforms":[{"type":"cluster"}],"style":{"outline":{"r":0,"b":157,"g":100},"fill":{"r":255,"b":255,"g":255}},"dimensions":[{"scale":{"padding":{"right":"5%"},"spans":[{"outRange":{"min":0.0,"max":1.0}}]},"axis":[{"tickStyle":{"padding":5.0,"fill":{"r":0,"b":157,"g":100},"font":{"size":"8pt","weight":"normal","family":"sans-serif"}},"gridStyle":{"fill":{"r":0,"b":157,"g":100}},"lineStyle":{"fill":{"r":0,"b":157,"g":100},"stroke":{"width":0.6666667}},"titleStyle":{"padding":6.0,"fill":{"r":0,"b":157,"g":100},"font":{"size":"12pt","weight":"bold","family":"sans-serif"}},"title":["Count"],"markStyle":{"fill":{"a":0,"r":0,"b":157,"g":100},"stroke":{"width":1.3333334}}}]},{"scale":{"categoryStructure":{"gap":"15%","nest":true},"local":false},"axis":[{"tickStyle":{"padding":5.0,"fill":{"r":0,"b":157,"g":100},"font":{"size":"12pt","weight":"normal","family":"sans-serif"}},"lineStyle":{"fill":{"r":0,"b":157,"g":100},"stroke":{"width":0.6666667}},"titleStyle":{"padding":6.0,"fill":{"r":0,"b":157,"g":100},"font":{"size":"12pt","weight":"bold","family":"sans-serif"}},"title":["Albinism Type"],"markStyle":{"fill":{"a":0,"r":0,"b":157,"g":100},"stroke":{"width":1.3333334}}}]},{}]}}],"legends":[{"labelStyle":{"fill":{"r":0,"b":157,"g":100},"font":{"size":"10pt","weight":"normal","family":"sans-serif"}},"itemStyle":{"padding":3,"size":11},"titleStyle":{"padding":3.0,"fill":{"r":0,"b":157,"g":100},"align":"start","font":{"size":"12pt","weight":"bold","family":"sans-serif"}},"location":{"width":"24%"},"title":["LE\_Myopia"],"aesthetics":[{"aesthetic":{"$ref":"colorAestheticID"}}],"boundsStyle":{"padding":5,"outline":{"a":0.0,"r":0,"b":0,"g":0},"fill":{"a":0.0,"r":0,"b":0,"g":0}}}],"data":[{"id":"dSource","fields":[{"format":{"numericPattern":"###"},"id":"fVariable","label":"Albinism Type","categories":["OCA1A","OCA1B","OCA2"]},{"min":4.0,"max":22.0,"id":"fVariable1","label":"Count"},{"format":{"numericPattern":"###"},"id":"fVariable2","label":"LE\_Myopia","categories":["Hyperm","Myopia"]}],"rows":[[0,13,0],[1,22,0],[2,20,0],[0,4,1],[1,5,1],[2,6,1]]}],"size":{"width":850.0,"height":500.0},"style":{"outline":{"a":0.0,"r":0,"b":0,"g":0},"fill":{"r":255,"b":255,"g":255}},"titles":[{"backgroundStyle":{"outline":{"a":0.0,"r":0,"b":0,"g":0},"fill":{"a":0.0,"r":0,"b":0,"g":0}},"style":{"padding":3.0,"fill":{"r":0,"b":157,"g":100},"font":{"size":"12pt","weight":"bold","family":"sans-serif"}},"type":"title","content":["Bar Chart"]}],"version":"6.0"}

Albinism Type \* LE\_ref error  
Albinism Type \* LE\_ref error - Crosstab - May 11, 2022

CrosstabCrosstab, table, 2 levels of column headers and 3 levels of row headers, table with 7 columns and 19 rows

|  |  |  |  |  |  |  |
| --- | --- | --- | --- | --- | --- | --- |
|  | | | LE\_ref error | | | Total |
|  |  |  | No | Yes | 2 |  |
| Albinism Type | OCA1A | Count | 1 | 16 | 0 | 17 |
|  |  | % within Albinism Type | 5.9% | 94.1% | 0.0% | 100.0% |
|  |  | % within LE\_ref error | 5.3% | 32.0% | 0.0% | 24.3% |
|  |  | % of Total | 1.4% | 22.9% | 0.0% | 24.3% |
|  | OCA1B | Count | 10 | 17 | 0 | 27 |
|  |  | % within Albinism Type | 37.0% | 63.0% | 0.0% | 100.0% |
|  |  | % within LE\_ref error | 52.6% | 34.0% | 0.0% | 38.6% |
|  |  | % of Total | 14.3% | 24.3% | 0.0% | 38.6% |
|  | OCA2 | Count | 8 | 17 | 1 | 26 |
|  |  | % within Albinism Type | 30.8% | 65.4% | 3.8% | 100.0% |
|  |  | % within LE\_ref error | 42.1% | 34.0% | 100.0% | 37.1% |
|  |  | % of Total | 11.4% | 24.3% | 1.4% | 37.1% |
| Total | | Count | 19 | 50 | 1 | 70 |
|  |  | % within Albinism Type | 27.1% | 71.4% | 1.4% | 100.0% |
|  |  | % within LE\_ref error | 100.0% | 100.0% | 100.0% | 100.0% |
|  |  | % of Total | 27.1% | 71.4% | 1.4% | 100.0% |
|  |  |  |  |  |  |  |

Albinism Type \* LE\_ref error  
Albinism Type \* LE\_ref error - Chi-Square Tests - May 11, 2022

Chi-Square TestsChi-Square Tests, table, 1 levels of column headers and 1 levels of row headers, table with 4 columns and 7 rows

|  |  |  |  |
| --- | --- | --- | --- |
|  | Value | df | Asymptotic Significance (2-sided) |
| Pearson Chi-Square | 7.252a | 4 | .123 |
| Likelihood Ratio | 8.677 | 4 | .070 |
| Linear-by-Linear Association | 1.457 | 1 | .227 |
| N of Valid Cases | 70 |  |  |
|  |  |  |  |
| --- | --- | --- | --- |
| a. 4 cells (44.4%) have expected count less than 5. The minimum expected count is .24. | | | |
|  |  |  |  |

Albinism Type \* LE\_ref error  
Albinism Type \* LE\_ref error - Bar Chart - May 11, 2022

{"copyright":"(C) Copyright IBM Corp. 2011","grammar":[{"elements":[{"color":[{"field":{"$ref":"fVariable2"},"palette":["#00B2EF","#D9182D","#FFCF10","#7F1C7D","#007670","#DD731C","#17AF4B","#EE3D96","#838329","#82D1F5","#F04E37","#FFE14F","#AB1A86","#00A6A0","#F19027","#8CC63F","#F389AF","#A5A215","#00649D","#A91024","#FDB813","#3B2056","#006058","#B8471B","#00BA52","#BA006E","#594F13"],"id":"colorAestheticID"}],"data":{"$ref":"dSource"},"style":{"outline":{"r":0,"b":0,"g":0},"size":"100.0%","width":"90.0%","stroke":{"width":0.0}},"position":[{"field":{"$ref":"fVariable1"}},{"field":{"$ref":"fVariable"}},{"field":{"$ref":"fVariable2"}}],"type":"interval"}],"coordinates":{"transforms":[{"type":"cluster"}],"style":{"outline":{"r":0,"b":157,"g":100},"fill":{"r":255,"b":255,"g":255}},"dimensions":[{"scale":{"padding":{"right":"5%"},"spans":[{"outRange":{"min":0.0,"max":1.0}}]},"axis":[{"tickStyle":{"padding":5.0,"fill":{"r":0,"b":157,"g":100},"font":{"size":"8pt","weight":"normal","family":"sans-serif"}},"gridStyle":{"fill":{"r":0,"b":157,"g":100}},"lineStyle":{"fill":{"r":0,"b":157,"g":100},"stroke":{"width":0.6666667}},"titleStyle":{"padding":6.0,"fill":{"r":0,"b":157,"g":100},"font":{"size":"12pt","weight":"bold","family":"sans-serif"}},"title":["Count"],"markStyle":{"fill":{"a":0,"r":0,"b":157,"g":100},"stroke":{"width":1.3333334}}}]},{"scale":{"categoryStructure":{"gap":"15%","nest":true},"local":false},"axis":[{"tickStyle":{"padding":5.0,"fill":{"r":0,"b":157,"g":100},"font":{"size":"12pt","weight":"normal","family":"sans-serif"}},"lineStyle":{"fill":{"r":0,"b":157,"g":100},"stroke":{"width":0.6666667}},"titleStyle":{"padding":6.0,"fill":{"r":0,"b":157,"g":100},"font":{"size":"12pt","weight":"bold","family":"sans-serif"}},"title":["Albinism Type"],"markStyle":{"fill":{"a":0,"r":0,"b":157,"g":100},"stroke":{"width":1.3333334}}}]},{}]}}],"legends":[{"labelStyle":{"fill":{"r":0,"b":157,"g":100},"font":{"size":"10pt","weight":"normal","family":"sans-serif"}},"itemStyle":{"padding":3,"size":11},"titleStyle":{"padding":3.0,"fill":{"r":0,"b":157,"g":100},"align":"start","font":{"size":"12pt","weight":"bold","family":"sans-serif"}},"location":{"width":"24%"},"title":["LE\_ref error"],"aesthetics":[{"aesthetic":{"$ref":"colorAestheticID"}}],"boundsStyle":{"padding":5,"outline":{"a":0.0,"r":0,"b":0,"g":0},"fill":{"a":0.0,"r":0,"b":0,"g":0}}}],"data":[{"id":"dSource","fields":[{"format":{"numericPattern":"###"},"id":"fVariable","label":"Albinism Type","categories":["OCA1A","OCA1B","OCA2"]},{"min":1.0,"max":17.0,"id":"fVariable1","label":"Count"},{"format":{"numericPattern":"###"},"id":"fVariable2","label":"LE\_ref error","categories":["No","Yes","2"]}],"rows":[[0,1,0],[1,10,0],[2,8,0],[0,16,1],[1,17,1],[2,17,1],[2,1,2]]}],"size":{"width":850.0,"height":500.0},"style":{"outline":{"a":0.0,"r":0,"b":0,"g":0},"fill":{"r":255,"b":255,"g":255}},"titles":[{"backgroundStyle":{"outline":{"a":0.0,"r":0,"b":0,"g":0},"fill":{"a":0.0,"r":0,"b":0,"g":0}},"style":{"padding":3.0,"fill":{"r":0,"b":157,"g":100},"font":{"size":"12pt","weight":"bold","family":"sans-serif"}},"type":"title","content":["Bar Chart"]}],"version":"6.0"}

Albinism Type \* LE\_cyl  
Albinism Type \* LE\_cyl - Crosstab - May 11, 2022

CrosstabCrosstab, table, 2 levels of column headers and 3 levels of row headers, table with 6 columns and 19 rows

|  |  |  |  |  |  |
| --- | --- | --- | --- | --- | --- |
|  | | | LE\_cyl | | Total |
|  |  |  | No | Yes |  |
| Albinism Type | OCA1A | Count | 2 | 15 | 17 |
|  |  | % within Albinism Type | 11.8% | 88.2% | 100.0% |
|  |  | % within LE\_cyl | 13.3% | 27.3% | 24.3% |
|  |  | % of Total | 2.9% | 21.4% | 24.3% |
|  | OCA1B | Count | 8 | 19 | 27 |
|  |  | % within Albinism Type | 29.6% | 70.4% | 100.0% |
|  |  | % within LE\_cyl | 53.3% | 34.5% | 38.6% |
|  |  | % of Total | 11.4% | 27.1% | 38.6% |
|  | OCA2 | Count | 5 | 21 | 26 |
|  |  | % within Albinism Type | 19.2% | 80.8% | 100.0% |
|  |  | % within LE\_cyl | 33.3% | 38.2% | 37.1% |
|  |  | % of Total | 7.1% | 30.0% | 37.1% |
| Total | | Count | 15 | 55 | 70 |
|  |  | % within Albinism Type | 21.4% | 78.6% | 100.0% |
|  |  | % within LE\_cyl | 100.0% | 100.0% | 100.0% |
|  |  | % of Total | 21.4% | 78.6% | 100.0% |
|  |  |  |  |  |  |

Albinism Type \* LE\_cyl  
Albinism Type \* LE\_cyl - Chi-Square Tests - May 11, 2022

Chi-Square TestsChi-Square Tests, table, 1 levels of column headers and 1 levels of row headers, table with 4 columns and 7 rows

|  |  |  |  |
| --- | --- | --- | --- |
|  | Value | df | Asymptotic Significance (2-sided) |
| Pearson Chi-Square | 2.096a | 2 | .351 |
| Likelihood Ratio | 2.154 | 2 | .341 |
| Linear-by-Linear Association | .161 | 1 | .689 |
| N of Valid Cases | 70 |  |  |
|  |  |  |  |
| --- | --- | --- | --- |
| a. 1 cells (16.7%) have expected count less than 5. The minimum expected count is 3.64. | | | |
|  |  |  |  |

Albinism Type \* LE\_cyl  
Albinism Type \* LE\_cyl - Bar Chart - May 11, 2022

{"copyright":"(C) Copyright IBM Corp. 2011","grammar":[{"elements":[{"color":[{"field":{"$ref":"fVariable2"},"palette":["#00B2EF","#D9182D","#FFCF10","#7F1C7D","#007670","#DD731C","#17AF4B","#EE3D96","#838329","#82D1F5","#F04E37","#FFE14F","#AB1A86","#00A6A0","#F19027","#8CC63F","#F389AF","#A5A215","#00649D","#A91024","#FDB813","#3B2056","#006058","#B8471B","#00BA52","#BA006E","#594F13"],"id":"colorAestheticID"}],"data":{"$ref":"dSource"},"style":{"outline":{"r":0,"b":0,"g":0},"size":"100.0%","width":"90.0%","stroke":{"width":0.0}},"position":[{"field":{"$ref":"fVariable1"}},{"field":{"$ref":"fVariable"}},{"field":{"$ref":"fVariable2"}}],"type":"interval"}],"coordinates":{"transforms":[{"type":"cluster"}],"style":{"outline":{"r":0,"b":157,"g":100},"fill":{"r":255,"b":255,"g":255}},"dimensions":[{"scale":{"padding":{"right":"5%"},"spans":[{"outRange":{"min":0.0,"max":1.0}}]},"axis":[{"tickStyle":{"padding":5.0,"fill":{"r":0,"b":157,"g":100},"font":{"size":"8pt","weight":"normal","family":"sans-serif"}},"gridStyle":{"fill":{"r":0,"b":157,"g":100}},"lineStyle":{"fill":{"r":0,"b":157,"g":100},"stroke":{"width":0.6666667}},"titleStyle":{"padding":6.0,"fill":{"r":0,"b":157,"g":100},"font":{"size":"12pt","weight":"bold","family":"sans-serif"}},"title":["Count"],"markStyle":{"fill":{"a":0,"r":0,"b":157,"g":100},"stroke":{"width":1.3333334}}}]},{"scale":{"categoryStructure":{"gap":"15%","nest":true},"local":false},"axis":[{"tickStyle":{"padding":5.0,"fill":{"r":0,"b":157,"g":100},"font":{"size":"12pt","weight":"normal","family":"sans-serif"}},"lineStyle":{"fill":{"r":0,"b":157,"g":100},"stroke":{"width":0.6666667}},"titleStyle":{"padding":6.0,"fill":{"r":0,"b":157,"g":100},"font":{"size":"12pt","weight":"bold","family":"sans-serif"}},"title":["Albinism Type"],"markStyle":{"fill":{"a":0,"r":0,"b":157,"g":100},"stroke":{"width":1.3333334}}}]},{}]}}],"legends":[{"labelStyle":{"fill":{"r":0,"b":157,"g":100},"font":{"size":"10pt","weight":"normal","family":"sans-serif"}},"itemStyle":{"padding":3,"size":11},"titleStyle":{"padding":3.0,"fill":{"r":0,"b":157,"g":100},"align":"start","font":{"size":"12pt","weight":"bold","family":"sans-serif"}},"location":{"width":"24%"},"title":["LE\_cyl"],"aesthetics":[{"aesthetic":{"$ref":"colorAestheticID"}}],"boundsStyle":{"padding":5,"outline":{"a":0.0,"r":0,"b":0,"g":0},"fill":{"a":0.0,"r":0,"b":0,"g":0}}}],"data":[{"id":"dSource","fields":[{"format":{"numericPattern":"###"},"id":"fVariable","label":"Albinism Type","categories":["OCA1A","OCA1B","OCA2"]},{"min":2.0,"max":21.0,"id":"fVariable1","label":"Count"},{"format":{"numericPattern":"###"},"id":"fVariable2","label":"LE\_cyl","categories":["No","Yes"]}],"rows":[[0,2,0],[1,8,0],[2,5,0],[0,15,1],[1,19,1],[2,21,1]]}],"size":{"width":850.0,"height":500.0},"style":{"outline":{"a":0.0,"r":0,"b":0,"g":0},"fill":{"r":255,"b":255,"g":255}},"titles":[{"backgroundStyle":{"outline":{"a":0.0,"r":0,"b":0,"g":0},"fill":{"a":0.0,"r":0,"b":0,"g":0}},"style":{"padding":3.0,"fill":{"r":0,"b":157,"g":100},"font":{"size":"12pt","weight":"bold","family":"sans-serif"}},"type":"title","content":["Bar Chart"]}],"version":"6.0"}

Log  
Log - Log - May 11, 2022

CREATE  
  /V1LogM\_1=DIFF(V1LogMar 1)/V2LogM\_1=DIFF(V2LogMar 2)/V3LogM\_1=DIFF(V3LogMar  
    3)/V4LogM\_1=DIFF(V4LogMar 4).

Create  
Create - Created Series - May 11, 2022

Created SeriesCreated Series, table, 2 levels of column headers and 1 levels of row headers, table with 6 columns and 7 rows

|  |  |  |  |  |  |
| --- | --- | --- | --- | --- | --- |
|  | Series Name | Case Number of Non-Missing Values | | N of Valid Cases | Creating Function |
|  |  | First | Last |  |  |
| 1 | V1LogM\_1 | 2 | 105 | 86 | DIFF(V1LogMar,1) |
| 2 | V2LogM\_1 | 3 | 105 | 85 | DIFF(V2LogMar,2) |
| 3 | V3LogM\_1 | 6 | 98 | 2 | DIFF(V3LogMar,3) |
| 4 | V4LogM\_1 | . | . | 0 | DIFF(V4LogMar,4) |
|  |  |  |  |  |  |

Log  
Log - Log - May 11, 2022

DATASET ACTIVATE DataSet1.  
  
SAVE OUTFILE='D:\liavofra\עבודה\לקוחות\Hadassah\Claudia\Albinism\WF2\_new.sav'  
  /COMPRESSED.  
USE ALL.  
COMPUTE filter\_$=(AlbinismType=1  | AlbinismType=2  | AlbinismType=3).  
VARIABLE LABELS filter\_$ 'AlbinismType=1  | AlbinismType=2  | AlbinismType=3 (FILTER)'.  
VALUE LABELS filter\_$ 0 'Not Selected' 1 'Selected'.  
FORMATS filter\_$ (f1.0).  
FILTER BY filter\_$.  
EXECUTE.  
ONEWAY V1LogMar V2LogMar V3LogMar V4LogMar BY AlbinismType  
  /STATISTICS DESCRIPTIVES  
  /PLOT MEANS  
  /MISSING ANALYSIS  
  /POSTHOC=SCHEFFE ALPHA(0.05).

Oneway  
Oneway - Descriptives - May 11, 2022

DescriptivesDescriptives, table, 2 levels of column headers and 2 levels of row headers, table with 10 columns and 19 rows

|  |  |  |  |  |  |  |  |  |  |
| --- | --- | --- | --- | --- | --- | --- | --- | --- | --- |
|  | | N | Mean | Std. Deviation | Std. Error | 95% Confidence Interval for Mean | | Minimum | Maximum |
|  |  |  |  |  |  | Lower Bound | Upper Bound |  |  |
| V1 LogMar | OCA1A | 16 | .9500 | .32863 | .08216 | .7749 | 1.1251 | .60 | 2.00 |
|  | OCA1B | 26 | .6000 | .16248 | .03187 | .5344 | .6656 | .30 | 1.00 |
|  | OCA2 | 23 | .7043 | .18210 | .03797 | .6256 | .7831 | .30 | 1.00 |
|  | Total | 65 | .7231 | .25725 | .03191 | .6593 | .7868 | .30 | 2.00 |
| V2 LogMar | OCA1A | 16 | .8688 | .20238 | .05060 | .7609 | .9766 | .50 | 1.30 |
|  | OCA1B | 28 | .5161 | .19581 | .03701 | .4401 | .5920 | .20 | 1.00 |
|  | OCA2 | 24 | .6250 | .19393 | .03959 | .5431 | .7069 | .30 | 1.10 |
|  | Total | 68 | .6375 | .23774 | .02883 | .5800 | .6950 | .20 | 1.30 |
| V3 LogMar | OCA1A | 8 | .7125 | .14577 | .05154 | .5906 | .8344 | .40 | .90 |
|  | OCA1B | 13 | .5000 | .22361 | .06202 | .3649 | .6351 | .10 | .80 |
|  | OCA2 | 12 | .5333 | .22293 | .06435 | .3917 | .6750 | .10 | .80 |
|  | Total | 33 | .5636 | .21911 | .03814 | .4859 | .6413 | .10 | .90 |
| V4 LogMar | OCA1A | 5 | .6900 | .21909 | .09798 | .4180 | .9620 | .35 | .90 |
|  | OCA1B | 5 | .6000 | .12247 | .05477 | .4479 | .7521 | .40 | .70 |
|  | OCA2 | 9 | .5278 | .23333 | .07778 | .3484 | .7071 | .10 | .80 |
|  | Total | 19 | .5895 | .20722 | .04754 | .4896 | .6893 | .10 | .90 |
|  |  |  |  |  |  |  |  |  |  |

Oneway  
Oneway - ANOVA - May 11, 2022

ANOVAANOVA, table, 1 levels of column headers and 2 levels of row headers, table with 7 columns and 14 rows

|  |  |  |  |  |  |  |
| --- | --- | --- | --- | --- | --- | --- |
|  | | Sum of Squares | df | Mean Square | F | Sig. |
| V1 LogMar | Between Groups | 1.226 | 2 | .613 | 12.627 | .000 |
|  | Within Groups | 3.010 | 62 | .049 |  |  |
|  | Total | 4.235 | 64 |  |  |  |
| V2 LogMar | Between Groups | 1.272 | 2 | .636 | 16.443 | .000 |
|  | Within Groups | 2.515 | 65 | .039 |  |  |
|  | Total | 3.787 | 67 |  |  |  |
| V3 LogMar | Between Groups | .241 | 2 | .120 | 2.790 | .077 |
|  | Within Groups | 1.295 | 30 | .043 |  |  |
|  | Total | 1.536 | 32 |  |  |  |
| V4 LogMar | Between Groups | .085 | 2 | .043 | .993 | .392 |
|  | Within Groups | .688 | 16 | .043 |  |  |
|  | Total | .773 | 18 |  |  |  |
|  |  |  |  |  |  |  |

Post Hoc Tests  
Post Hoc Tests - Multiple Comparisons - May 11, 2022

Multiple ComparisonsMultiple Comparisons, table, Scheffe, 1 layers, 2 levels of column headers and 3 levels of row headers, table with 8 columns and 29 rows

|  |  |  |  |  |  |  |  |  |
| --- | --- | --- | --- | --- | --- | --- | --- | --- |
| |  |  | | --- | --- | | Scheffe | Scheffe | | | | | | | | |  |
|  |  |  |  |  |  |  |  |  |
| --- | --- | --- | --- | --- | --- | --- | --- | --- |
| Dependent Variable | (I) Albinism Type | (J) Albinism Type | Mean Difference (I-J) | Std. Error | Sig. | 95% Confidence Interval | |  |
|  |  |  |  |  |  | Lower Bound | Upper Bound |  |
| V1 LogMar | OCA1A | OCA1B | .35000\* | .07001 | .000 | .1744 | .5256 |  |
|  |  | OCA2 | .24565\* | .07172 | .005 | .0658 | .4255 |  |
|  | OCA1B | OCA1A | -.35000\* | .07001 | .000 | -.5256 | -.1744 |  |
|  |  | OCA2 | -.10435 | .06307 | .262 | -.2625 | .0538 |  |
|  | OCA2 | OCA1A | -.24565\* | .07172 | .005 | -.4255 | -.0658 |  |
|  |  | OCA1B | .10435 | .06307 | .262 | -.0538 | .2625 |  |
| V2 LogMar | OCA1A | OCA1B | .35268\* | .06164 | .000 | .1983 | .5071 |  |
|  |  | OCA2 | .24375\* | .06348 | .001 | .0847 | .4028 |  |
|  | OCA1B | OCA1A | -.35268\* | .06164 | .000 | -.5071 | -.1983 |  |
|  |  | OCA2 | -.10893 | .05471 | .146 | -.2460 | .0281 |  |
|  | OCA2 | OCA1A | -.24375\* | .06348 | .001 | -.4028 | -.0847 |  |
|  |  | OCA1B | .10893 | .05471 | .146 | -.0281 | .2460 |  |
| V3 LogMar | OCA1A | OCA1B | .21250 | .09338 | .092 | -.0280 | .4530 |  |
|  |  | OCA2 | .17917 | .09485 | .185 | -.0651 | .4234 |  |
|  | OCA1B | OCA1A | -.21250 | .09338 | .092 | -.4530 | .0280 |  |
|  |  | OCA2 | -.03333 | .08319 | .923 | -.2476 | .1809 |  |
|  | OCA2 | OCA1A | -.17917 | .09485 | .185 | -.4234 | .0651 |  |
|  |  | OCA1B | .03333 | .08319 | .923 | -.1809 | .2476 |  |
| V4 LogMar | OCA1A | OCA1B | .09000 | .13111 | .793 | -.2634 | .4434 |  |
|  |  | OCA2 | .16222 | .11562 | .395 | -.1495 | .4739 |  |
|  | OCA1B | OCA1A | -.09000 | .13111 | .793 | -.4434 | .2634 |  |
|  |  | OCA2 | .07222 | .11562 | .825 | -.2395 | .3839 |  |
|  | OCA2 | OCA1A | -.16222 | .11562 | .395 | -.4739 | .1495 |  |
|  |  | OCA1B | -.07222 | .11562 | .825 | -.3839 | .2395 |  |
|  |  |  |  |  |  |  |  |  |
| --- | --- | --- | --- | --- | --- | --- | --- | --- |
| \*. The mean difference is significant at the 0.05 level. | | | | | | | |  |
|  |  |  |  |  |  |  |  |  |

Homogeneous Subsets  
Homogeneous Subsets - V1 LogMar - May 11, 2022

V1 LogMarV1 LogMar, table, Scheffe, 1 layers, 2 levels of column headers and 1 levels of row headers, table with 4 columns and 11 rows

|  |  |  |  |
| --- | --- | --- | --- |
| |  |  | | --- | --- | | Scheffea,b | Scheffea,b | | | | |
|  |  |  |  |
| --- | --- | --- | --- |
| Albinism Type | N | Subset for alpha = 0.05 | |
|  |  | 1 | 2 |
| OCA1B | 26 | .6000 |  |
| OCA2 | 23 | .7043 |  |
| OCA1A | 16 |  | .9500 |
| Sig. |  | .319 | 1.000 |
|  |  |  |  |
| --- | --- | --- | --- |
| Means for groups in homogeneous subsets are displayed. | | | |
| a. Uses Harmonic Mean Sample Size = 20.770. | | | |
| b. The group sizes are unequal. The harmonic mean of the group sizes is used. Type I error levels are not guaranteed. | | | |
|  |  |  |  |

Homogeneous Subsets  
Homogeneous Subsets - V2 LogMar - May 11, 2022

V2 LogMarV2 LogMar, table, Scheffe, 1 layers, 2 levels of column headers and 1 levels of row headers, table with 4 columns and 11 rows

|  |  |  |  |
| --- | --- | --- | --- |
| |  |  | | --- | --- | | Scheffea,b | Scheffea,b | | | | |
|  |  |  |  |
| --- | --- | --- | --- |
| Albinism Type | N | Subset for alpha = 0.05 | |
|  |  | 1 | 2 |
| OCA1B | 28 | .5161 |  |
| OCA2 | 24 | .6250 |  |
| OCA1A | 16 |  | .8688 |
| Sig. |  | .201 | 1.000 |
|  |  |  |  |
| --- | --- | --- | --- |
| Means for groups in homogeneous subsets are displayed. | | | |
| a. Uses Harmonic Mean Sample Size = 21.447. | | | |
| b. The group sizes are unequal. The harmonic mean of the group sizes is used. Type I error levels are not guaranteed. | | | |
|  |  |  |  |

Homogeneous Subsets  
Homogeneous Subsets - V3 LogMar - May 11, 2022

V3 LogMarV3 LogMar, table, Scheffe, 1 layers, 2 levels of column headers and 1 levels of row headers, table with 3 columns and 11 rows

|  |  |  |
| --- | --- | --- |
| |  |  | | --- | --- | | Scheffea,b | Scheffea,b | | | |
|  |  |  |
| --- | --- | --- |
| Albinism Type | N | Subset for alpha = 0.05 |
|  |  | 1 |
| OCA1B | 13 | .5000 |
| OCA2 | 12 | .5333 |
| OCA1A | 8 | .7125 |
| Sig. |  | .080 |
|  |  |  |
| --- | --- | --- |
| Means for groups in homogeneous subsets are displayed. | | |
| a. Uses Harmonic Mean Sample Size = 10.517. | | |
| b. The group sizes are unequal. The harmonic mean of the group sizes is used. Type I error levels are not guaranteed. | | |
|  |  |  |

Homogeneous Subsets  
Homogeneous Subsets - V4 LogMar - May 11, 2022

V4 LogMarV4 LogMar, table, Scheffe, 1 layers, 2 levels of column headers and 1 levels of row headers, table with 3 columns and 11 rows

|  |  |  |
| --- | --- | --- |
| |  |  | | --- | --- | | Scheffea,b | Scheffea,b | | | |
|  |  |  |
| --- | --- | --- |
| Albinism Type | N | Subset for alpha = 0.05 |
|  |  | 1 |
| OCA2 | 9 | .5278 |
| OCA1B | 5 | .6000 |
| OCA1A | 5 | .6900 |
| Sig. |  | .427 |
|  |  |  |
| --- | --- | --- |
| Means for groups in homogeneous subsets are displayed. | | |
| a. Uses Harmonic Mean Sample Size = 5.870. | | |
| b. The group sizes are unequal. The harmonic mean of the group sizes is used. Type I error levels are not guaranteed. | | |
|  |  |  |

Means Plots  
Means Plots - V1 LogMar - May 11, 2022

{"copyright":"(C) Copyright IBM Corp. 2011","grammar":[{"elements":[{"data":{"$ref":"dSource"},"style":{"outline":{"r":0,"b":0,"g":0},"fill":{"r":0,"b":157,"g":100},"stroke":{"width":3.3333333}},"position":[{"field":{"$ref":"fVariable1"}},{"field":{"$ref":"fVariable"}}],"type":"line"},{"data":{"$ref":"dSource"},"style":{"symbol":"circle","outline":{"r":0,"b":157,"g":100},"size":6.6666665,"fill":{"r":119,"b":119,"g":118}},"position":[{"field":{"$ref":"fVariable1"}},{"field":{"$ref":"fVariable"}}],"type":"point"}],"coordinates":{"style":{"outline":{"r":0,"b":157,"g":100},"fill":{"r":255,"b":255,"g":255}},"dimensions":[{"scale":{"padding":{"left":"5%","right":"5%"}},"axis":[{"tickStyle":{"padding":5.0,"fill":{"r":0,"b":157,"g":100},"font":{"size":"8pt","weight":"normal","family":"sans-serif"}},"gridStyle":{"fill":{"r":0,"b":157,"g":100}},"lineStyle":{"fill":{"r":0,"b":157,"g":100},"stroke":{"width":0.6666667}},"titleStyle":{"padding":6.0,"fill":{"r":0,"b":157,"g":100},"font":{"size":"12pt","weight":"bold","family":"sans-serif"}},"title":["Mean of V1 LogMar"],"markStyle":{"fill":{"a":0,"r":0,"b":157,"g":100},"stroke":{"width":1.3333334}}}]},{"scale":{"padding":{"left":"5%","right":"5%"}},"axis":[{"tickStyle":{"padding":5.0,"fill":{"r":0,"b":157,"g":100},"font":{"size":"12pt","weight":"normal","family":"sans-serif"}},"lineStyle":{"fill":{"r":0,"b":157,"g":100},"stroke":{"width":0.6666667}},"titleStyle":{"padding":6.0,"fill":{"r":0,"b":157,"g":100},"font":{"size":"12pt","weight":"bold","family":"sans-serif"}},"title":["Albinism Type"],"markStyle":{"fill":{"a":0,"r":0,"b":157,"g":100},"stroke":{"width":1.3333334}}}]}]}}],"data":[{"id":"dSource","fields":[{"format":{"numericPattern":"###"},"id":"fVariable","label":"Albinism Type","categories":["OCA1A","OCA1B","OCA2"]},{"min":0.5999999999999999,"max":0.9500000000000002,"format":{"numericPattern":"###.00"},"id":"fVariable1","label":"V1 LogMar"}],"rows":[[0,0.9500000000000002],[1,0.5999999999999999],[2,0.7043478260869567]]}],"size":{"width":850.0,"height":500.0},"style":{"outline":{"a":0.0,"r":0,"b":0,"g":0},"fill":{"r":255,"b":255,"g":255}},"version":"6.0"}

Means Plots  
Means Plots - V2 LogMar - May 11, 2022

{"copyright":"(C) Copyright IBM Corp. 2011","grammar":[{"elements":[{"data":{"$ref":"dSource"},"style":{"outline":{"r":0,"b":0,"g":0},"fill":{"r":0,"b":157,"g":100},"stroke":{"width":3.3333333}},"position":[{"field":{"$ref":"fVariable1"}},{"field":{"$ref":"fVariable"}}],"type":"line"},{"data":{"$ref":"dSource"},"style":{"symbol":"circle","outline":{"r":0,"b":157,"g":100},"size":6.6666665,"fill":{"r":119,"b":119,"g":118}},"position":[{"field":{"$ref":"fVariable1"}},{"field":{"$ref":"fVariable"}}],"type":"point"}],"coordinates":{"style":{"outline":{"r":0,"b":157,"g":100},"fill":{"r":255,"b":255,"g":255}},"dimensions":[{"scale":{"padding":{"left":"5%","right":"5%"}},"axis":[{"tickStyle":{"padding":5.0,"fill":{"r":0,"b":157,"g":100},"font":{"size":"8pt","weight":"normal","family":"sans-serif"}},"gridStyle":{"fill":{"r":0,"b":157,"g":100}},"lineStyle":{"fill":{"r":0,"b":157,"g":100},"stroke":{"width":0.6666667}},"titleStyle":{"padding":6.0,"fill":{"r":0,"b":157,"g":100},"font":{"size":"12pt","weight":"bold","family":"sans-serif"}},"title":["Mean of V2 LogMar"],"markStyle":{"fill":{"a":0,"r":0,"b":157,"g":100},"stroke":{"width":1.3333334}}}]},{"scale":{"padding":{"left":"5%","right":"5%"}},"axis":[{"tickStyle":{"padding":5.0,"fill":{"r":0,"b":157,"g":100},"font":{"size":"12pt","weight":"normal","family":"sans-serif"}},"lineStyle":{"fill":{"r":0,"b":157,"g":100},"stroke":{"width":0.6666667}},"titleStyle":{"padding":6.0,"fill":{"r":0,"b":157,"g":100},"font":{"size":"12pt","weight":"bold","family":"sans-serif"}},"title":["Albinism Type"],"markStyle":{"fill":{"a":0,"r":0,"b":157,"g":100},"stroke":{"width":1.3333334}}}]}]}}],"data":[{"id":"dSource","fields":[{"format":{"numericPattern":"###"},"id":"fVariable","label":"Albinism Type","categories":["OCA1A","OCA1B","OCA2"]},{"min":0.5160714285714286,"max":0.8687500000000002,"format":{"numericPattern":"###.00"},"id":"fVariable1","label":"V2 LogMar"}],"rows":[[0,0.8687500000000002],[1,0.5160714285714286],[2,0.6250000000000001]]}],"size":{"width":850.0,"height":500.0},"style":{"outline":{"a":0.0,"r":0,"b":0,"g":0},"fill":{"r":255,"b":255,"g":255}},"version":"6.0"}

Means Plots  
Means Plots - V3 LogMar - May 11, 2022

{"copyright":"(C) Copyright IBM Corp. 2011","grammar":[{"elements":[{"data":{"$ref":"dSource"},"style":{"outline":{"r":0,"b":0,"g":0},"fill":{"r":0,"b":157,"g":100},"stroke":{"width":3.3333333}},"position":[{"field":{"$ref":"fVariable1"}},{"field":{"$ref":"fVariable"}}],"type":"line"},{"data":{"$ref":"dSource"},"style":{"symbol":"circle","outline":{"r":0,"b":157,"g":100},"size":6.6666665,"fill":{"r":119,"b":119,"g":118}},"position":[{"field":{"$ref":"fVariable1"}},{"field":{"$ref":"fVariable"}}],"type":"point"}],"coordinates":{"style":{"outline":{"r":0,"b":157,"g":100},"fill":{"r":255,"b":255,"g":255}},"dimensions":[{"scale":{"padding":{"left":"5%","right":"5%"}},"axis":[{"tickStyle":{"padding":5.0,"fill":{"r":0,"b":157,"g":100},"font":{"size":"8pt","weight":"normal","family":"sans-serif"}},"gridStyle":{"fill":{"r":0,"b":157,"g":100}},"lineStyle":{"fill":{"r":0,"b":157,"g":100},"stroke":{"width":0.6666667}},"titleStyle":{"padding":6.0,"fill":{"r":0,"b":157,"g":100},"font":{"size":"12pt","weight":"bold","family":"sans-serif"}},"title":["Mean of V3 LogMar"],"markStyle":{"fill":{"a":0,"r":0,"b":157,"g":100},"stroke":{"width":1.3333334}}}]},{"scale":{"padding":{"left":"5%","right":"5%"}},"axis":[{"tickStyle":{"padding":5.0,"fill":{"r":0,"b":157,"g":100},"font":{"size":"12pt","weight":"normal","family":"sans-serif"}},"lineStyle":{"fill":{"r":0,"b":157,"g":100},"stroke":{"width":0.6666667}},"titleStyle":{"padding":6.0,"fill":{"r":0,"b":157,"g":100},"font":{"size":"12pt","weight":"bold","family":"sans-serif"}},"title":["Albinism Type"],"markStyle":{"fill":{"a":0,"r":0,"b":157,"g":100},"stroke":{"width":1.3333334}}}]}]}}],"data":[{"id":"dSource","fields":[{"format":{"numericPattern":"###"},"id":"fVariable","label":"Albinism Type","categories":["OCA1A","OCA1B","OCA2"]},{"min":0.4999999999999999,"max":0.7125,"format":{"numericPattern":"###.00"},"id":"fVariable1","label":"V3 LogMar"}],"rows":[[0,0.7125],[1,0.4999999999999999],[2,0.5333333333333333]]}],"size":{"width":850.0,"height":500.0},"style":{"outline":{"a":0.0,"r":0,"b":0,"g":0},"fill":{"r":255,"b":255,"g":255}},"version":"6.0"}

Means Plots  
Means Plots - V4 LogMar - May 11, 2022

{"copyright":"(C) Copyright IBM Corp. 2011","grammar":[{"elements":[{"data":{"$ref":"dSource"},"style":{"outline":{"r":0,"b":0,"g":0},"fill":{"r":0,"b":157,"g":100},"stroke":{"width":3.3333333}},"position":[{"field":{"$ref":"fVariable1"}},{"field":{"$ref":"fVariable"}}],"type":"line"},{"data":{"$ref":"dSource"},"style":{"symbol":"circle","outline":{"r":0,"b":157,"g":100},"size":6.6666665,"fill":{"r":119,"b":119,"g":118}},"position":[{"field":{"$ref":"fVariable1"}},{"field":{"$ref":"fVariable"}}],"type":"point"}],"coordinates":{"style":{"outline":{"r":0,"b":157,"g":100},"fill":{"r":255,"b":255,"g":255}},"dimensions":[{"scale":{"padding":{"left":"5%","right":"5%"}},"axis":[{"tickStyle":{"padding":5.0,"fill":{"r":0,"b":157,"g":100},"font":{"size":"8pt","weight":"normal","family":"sans-serif"}},"gridStyle":{"fill":{"r":0,"b":157,"g":100}},"lineStyle":{"fill":{"r":0,"b":157,"g":100},"stroke":{"width":0.6666667}},"titleStyle":{"padding":6.0,"fill":{"r":0,"b":157,"g":100},"font":{"size":"12pt","weight":"bold","family":"sans-serif"}},"title":["Mean of V4 LogMar"],"markStyle":{"fill":{"a":0,"r":0,"b":157,"g":100},"stroke":{"width":1.3333334}}}]},{"scale":{"padding":{"left":"5%","right":"5%"}},"axis":[{"tickStyle":{"padding":5.0,"fill":{"r":0,"b":157,"g":100},"font":{"size":"12pt","weight":"normal","family":"sans-serif"}},"lineStyle":{"fill":{"r":0,"b":157,"g":100},"stroke":{"width":0.6666667}},"titleStyle":{"padding":6.0,"fill":{"r":0,"b":157,"g":100},"font":{"size":"12pt","weight":"bold","family":"sans-serif"}},"title":["Albinism Type"],"markStyle":{"fill":{"a":0,"r":0,"b":157,"g":100},"stroke":{"width":1.3333334}}}]}]}}],"data":[{"id":"dSource","fields":[{"format":{"numericPattern":"###"},"id":"fVariable","label":"Albinism Type","categories":["OCA1A","OCA1B","OCA2"]},{"min":0.5277777777777777,"max":0.69,"format":{"numericPattern":"###.00"},"id":"fVariable1","label":"V4 LogMar"}],"rows":[[0,0.69],[1,0.6],[2,0.5277777777777777]]}],"size":{"width":850.0,"height":500.0},"style":{"outline":{"a":0.0,"r":0,"b":0,"g":0},"fill":{"r":255,"b":255,"g":255}},"version":"6.0"}

Log  
Log - Log - May 11, 2022

GLM V1LogMar V2LogMar V3LogMar V4LogMar BY AlbinismType  
  /WSFACTOR=Visit 4 Polynomial  
  /MEASURE=Time  
  /METHOD=SSTYPE(3)  
  /CRITERIA=ALPHA(.05)  
  /WSDESIGN=Visit  
  /DESIGN=AlbinismType.

General Linear Model  
General Linear Model - Within-Subjects Factors - May 11, 2022

Within-Subjects FactorsWithin-Subjects Factors, table, Measure, Time, 1 layers, 1 levels of column headers and 1 levels of row headers, table with 2 columns and 7 rows

|  |  |  |  |  |
| --- | --- | --- | --- | --- |
| |  |  |  | | --- | --- | --- | | Measure: | Time | Time | | |
|  |  |
| --- | --- |
| Visit | Dependent Variable |
| 1 | V1LogMar |
| 2 | V2LogMar |
| 3 | V3LogMar |
| 4 | V4LogMar |
|  |  |

General Linear Model  
General Linear Model - Between-Subjects Factors - May 11, 2022

Between-Subjects FactorsBetween-Subjects Factors, table, 1 levels of column headers and 2 levels of row headers, table with 4 columns and 5 rows

|  |  |  |  |
| --- | --- | --- | --- |
|  | | Value Label | N |
| Albinism Type | 1 | OCA1A | 4 |
|  | 2 | OCA1B | 4 |
|  | 3 | OCA2 | 6 |
|  |  |  |  |

General Linear Model  
General Linear Model - Multivariate Tests - May 11, 2022

Multivariate TestsaMultivariate Tests, table, 1 levels of column headers and 2 levels of row headers, table with 7 columns and 13 rows

|  |  |  |  |  |  |  |
| --- | --- | --- | --- | --- | --- | --- |
| Effect | | Value | F | Hypothesis df | Error df | Sig. |
| Visit | Pillai's Trace | .639 | 5.306b | 3.000 | 9.000 | .022 |
|  | Wilks' Lambda | .361 | 5.306b | 3.000 | 9.000 | .022 |
|  | Hotelling's Trace | 1.769 | 5.306b | 3.000 | 9.000 | .022 |
|  | Roy's Largest Root | 1.769 | 5.306b | 3.000 | 9.000 | .022 |
| Visit \* AlbinismType | Pillai's Trace | .325 | .646 | 6.000 | 20.000 | .693 |
|  | Wilks' Lambda | .688 | .617b | 6.000 | 18.000 | .714 |
|  | Hotelling's Trace | .435 | .581 | 6.000 | 16.000 | .741 |
|  | Roy's Largest Root | .389 | 1.295c | 3.000 | 10.000 | .329 |
|  |  |  |  |  |  |  |
| --- | --- | --- | --- | --- | --- | --- |
| a. Design: Intercept + AlbinismType   Within Subjects Design: Visit | | | | | | |
| b. Exact statistic | | | | | | |
| c. The statistic is an upper bound on F that yields a lower bound on the significance level. | | | | | | |
|  |  |  |  |  |  |  |

General Linear Model  
General Linear Model - Mauchly's Test of Sphericity - May 11, 2022

Mauchly's Test of SphericityaMauchly's Test of Sphericity, table, Measure, Time, 1 layers, 2 levels of column headers and 1 levels of row headers, table with 8 columns and 8 rows

|  |  |  |  |  |  |  |  |
| --- | --- | --- | --- | --- | --- | --- | --- |
| |  |  |  | | --- | --- | --- | | Measure: | Time | Time | | | | | | | | |
|  |  |  |  |  |  |  |  |
| --- | --- | --- | --- | --- | --- | --- | --- |
| Within Subjects Effect | Mauchly's W | Approx. Chi-Square | df | Sig. | Epsilonb | | |
|  |  |  |  |  | Greenhouse-Geisser | Huynh-Feldt | Lower-bound |
| Visit | .448 | 7.813 | 5 | .169 | .740 | 1.000 | .333 |
|  |  |  |  |  |  |  |  |
| --- | --- | --- | --- | --- | --- | --- | --- |
| Tests the null hypothesis that the error covariance matrix of the orthonormalized transformed dependent variables is proportional to an identity matrix. | | | | | | | |
| a. Design: Intercept + AlbinismType   Within Subjects Design: Visit | | | | | | | |
| b. May be used to adjust the degrees of freedom for the averaged tests of significance. Corrected tests are displayed in the Tests of Within-Subjects Effects table. | | | | | | | |
|  |  |  |  |  |  |  |  |

General Linear Model  
General Linear Model - Tests of Within-Subjects Effects - May 11, 2022

Tests of Within-Subjects EffectsTests of Within-Subjects Effects, table, Measure, Time, 1 layers, 1 levels of column headers and 2 levels of row headers, table with 7 columns and 15 rows

|  |  |  |  |  |  |  |
| --- | --- | --- | --- | --- | --- | --- |
| |  |  |  | | --- | --- | --- | | Measure: | Time | Time | | | | | | | |
|  |  |  |  |  |  |  |
| --- | --- | --- | --- | --- | --- | --- |
| Source | | Type III Sum of Squares | df | Mean Square | F | Sig. |
| Visit | Sphericity Assumed | .129 | 3 | .043 | 10.438 | .000 |
|  | Greenhouse-Geisser | .129 | 2.220 | .058 | 10.438 | .000 |
|  | Huynh-Feldt | .129 | 3.000 | .043 | 10.438 | .000 |
|  | Lower-bound | .129 | 1.000 | .129 | 10.438 | .008 |
| Visit \* AlbinismType | Sphericity Assumed | .008 | 6 | .001 | .337 | .913 |
|  | Greenhouse-Geisser | .008 | 4.439 | .002 | .337 | .868 |
|  | Huynh-Feldt | .008 | 6.000 | .001 | .337 | .913 |
|  | Lower-bound | .008 | 2.000 | .004 | .337 | .721 |
| Error(Visit) | Sphericity Assumed | .136 | 33 | .004 |  |  |
|  | Greenhouse-Geisser | .136 | 24.415 | .006 |  |  |
|  | Huynh-Feldt | .136 | 33.000 | .004 |  |  |
|  | Lower-bound | .136 | 11.000 | .012 |  |  |
|  |  |  |  |  |  |  |

General Linear Model  
General Linear Model - Tests of Within-Subjects Contrasts - May 11, 2022

Tests of Within-Subjects ContrastsTests of Within-Subjects Contrasts, table, Measure, Time, 1 layers, 1 levels of column headers and 2 levels of row headers, table with 7 columns and 12 rows

|  |  |  |  |  |  |  |
| --- | --- | --- | --- | --- | --- | --- |
| |  |  |  | | --- | --- | --- | | Measure: | Time | Time | | | | | | | |
|  |  |  |  |  |  |  |
| --- | --- | --- | --- | --- | --- | --- |
| Source | Visit | Type III Sum of Squares | df | Mean Square | F | Sig. |
| Visit | Linear | .123 | 1 | .123 | 17.892 | .001 |
|  | Quadratic | 5.859E-5 | 1 | 5.859E-5 | .030 | .865 |
|  | Cubic | .007 | 1 | .007 | 1.830 | .203 |
| Visit \* AlbinismType | Linear | .003 | 2 | .001 | .212 | .812 |
|  | Quadratic | .005 | 2 | .002 | 1.199 | .338 |
|  | Cubic | .001 | 2 | .000 | .107 | .900 |
| Error(Visit) | Linear | .075 | 11 | .007 |  |  |
|  | Quadratic | .021 | 11 | .002 |  |  |
|  | Cubic | .039 | 11 | .004 |  |  |
|  |  |  |  |  |  |  |

General Linear Model  
General Linear Model - Tests of Between-Subjects Effects - May 11, 2022

Tests of Between-Subjects EffectsTests of Between-Subjects Effects, table, Measure, Time, Transformed Variable, Average, 1 layers, 1 levels of column headers and 1 levels of row headers, table with 6 columns and 7 rows

|  |  |  |  |  |  |  |  |  |  |  |  |
| --- | --- | --- | --- | --- | --- | --- | --- | --- | --- | --- | --- |
| |  |  |  | | --- | --- | --- | | Measure: | Time | Time | | Transformed Variable: | Average | Average | | | | | | |
|  |  |  |  |  |  |
| --- | --- | --- | --- | --- | --- |
| Source | Type III Sum of Squares | df | Mean Square | F | Sig. |
| Intercept | 26.329 | 1 | 26.329 | 234.796 | .000 |
| AlbinismType | .179 | 2 | .090 | .798 | .475 |
| Error | 1.233 | 11 | .112 |  |  |
|  |  |  |  |  |  |

Log  
Log - Log - May 11, 2022

GLM V1LogMar V2LogMar V3LogMar V4LogMar BY nystagmus Eyemusclesurgery Srabismus  
  /WSFACTOR=Visit 4 Polynomial  
  /MEASURE=Time  
  /METHOD=SSTYPE(3)  
  /CRITERIA=ALPHA(.05)  
  /WSDESIGN=Visit  
  /DESIGN=nystagmus Eyemusclesurgery Srabismus nystagmus\*Eyemusclesurgery nystagmus\*Srabismus  
    Eyemusclesurgery\*Srabismus nystagmus\*Eyemusclesurgery\*Srabismus.

General Linear Model  
General Linear Model - Within-Subjects Factors - May 11, 2022

Within-Subjects FactorsWithin-Subjects Factors, table, Measure, Time, 1 layers, 1 levels of column headers and 1 levels of row headers, table with 2 columns and 7 rows

|  |  |  |  |  |
| --- | --- | --- | --- | --- |
| |  |  |  | | --- | --- | --- | | Measure: | Time | Time | | |
|  |  |
| --- | --- |
| Visit | Dependent Variable |
| 1 | V1LogMar |
| 2 | V2LogMar |
| 3 | V3LogMar |
| 4 | V4LogMar |
|  |  |

General Linear Model  
General Linear Model - Between-Subjects Factors - May 11, 2022

Between-Subjects FactorsBetween-Subjects Factors, table, 1 levels of column headers and 2 levels of row headers, table with 4 columns and 8 rows

|  |  |  |  |
| --- | --- | --- | --- |
|  | | Value Label | N |
| nystagmus | 1 | Yes | 14 |
| Eye muscle surgery | 0 | No | 5 |
|  | 1 | Yes | 9 |
| Srabismus | 0 | None | 4 |
|  | 1 | Iso | 6 |
|  | 2 | Exo | 4 |
|  |  |  |  |

General Linear Model  
General Linear Model - Multivariate Tests - May 11, 2022

Multivariate TestsaMultivariate Tests, table, 1 levels of column headers and 2 levels of row headers, table with 7 columns and 37 rows

|  |  |  |  |  |  |  |
| --- | --- | --- | --- | --- | --- | --- |
| Effect | | Value | F | Hypothesis df | Error df | Sig. |
| Visit | Pillai's Trace | .630 | 3.411b | 3.000 | 6.000 | .094 |
|  | Wilks' Lambda | .370 | 3.411b | 3.000 | 6.000 | .094 |
|  | Hotelling's Trace | 1.705 | 3.411b | 3.000 | 6.000 | .094 |
|  | Roy's Largest Root | 1.705 | 3.411b | 3.000 | 6.000 | .094 |
| Visit \* nystagmus | Pillai's Trace | .000 | .b | .000 | .000 | . |
|  | Wilks' Lambda | 1.000 | .b | .000 | 7.000 | . |
|  | Hotelling's Trace | .000 | .b | .000 | 2.000 | . |
|  | Roy's Largest Root | .000 | .000b | 3.000 | 5.000 | 1.000 |
| Visit \* Eyemusclesurgery | Pillai's Trace | .040 | .083b | 3.000 | 6.000 | .967 |
|  | Wilks' Lambda | .960 | .083b | 3.000 | 6.000 | .967 |
|  | Hotelling's Trace | .042 | .083b | 3.000 | 6.000 | .967 |
|  | Roy's Largest Root | .042 | .083b | 3.000 | 6.000 | .967 |
| Visit \* Srabismus | Pillai's Trace | .230 | .303 | 6.000 | 14.000 | .925 |
|  | Wilks' Lambda | .772 | .277b | 6.000 | 12.000 | .937 |
|  | Hotelling's Trace | .294 | .245 | 6.000 | 10.000 | .951 |
|  | Roy's Largest Root | .286 | .668c | 3.000 | 7.000 | .598 |
| Visit \* nystagmus \* Eyemusclesurgery | Pillai's Trace | .000 | .b | .000 | .000 | . |
|  | Wilks' Lambda | 1.000 | .b | .000 | 7.000 | . |
|  | Hotelling's Trace | .000 | .b | .000 | 2.000 | . |
|  | Roy's Largest Root | .000 | .000b | 3.000 | 5.000 | 1.000 |
| Visit \* nystagmus \* Srabismus | Pillai's Trace | .000 | .b | .000 | .000 | . |
|  | Wilks' Lambda | 1.000 | .b | .000 | 7.000 | . |
|  | Hotelling's Trace | .000 | .b | .000 | 2.000 | . |
|  | Roy's Largest Root | .000 | .000b | 3.000 | 5.000 | 1.000 |
| Visit \* Eyemusclesurgery \* Srabismus | Pillai's Trace | .331 | .462 | 6.000 | 14.000 | .825 |
|  | Wilks' Lambda | .681 | .423b | 6.000 | 12.000 | .850 |
|  | Hotelling's Trace | .449 | .375 | 6.000 | 10.000 | .879 |
|  | Roy's Largest Root | .405 | .945c | 3.000 | 7.000 | .469 |
| Visit \* nystagmus \* Eyemusclesurgery \* Srabismus | Pillai's Trace | .000 | .b | .000 | .000 | . |
|  | Wilks' Lambda | 1.000 | .b | .000 | 7.000 | . |
|  | Hotelling's Trace | .000 | .b | .000 | 2.000 | . |
|  | Roy's Largest Root | .000 | .000b | 3.000 | 5.000 | 1.000 |
|  |  |  |  |  |  |  |
| --- | --- | --- | --- | --- | --- | --- |
| a. Design: Intercept + nystagmus + Eyemusclesurgery + Srabismus + nystagmus \* Eyemusclesurgery + nystagmus \* Srabismus + Eyemusclesurgery \* Srabismus + nystagmus \* Eyemusclesurgery \* Srabismus   Within Subjects Design: Visit | | | | | | |
| b. Exact statistic | | | | | | |
| c. The statistic is an upper bound on F that yields a lower bound on the significance level. | | | | | | |
|  |  |  |  |  |  |  |

General Linear Model  
General Linear Model - Mauchly's Test of Sphericity - May 11, 2022

Mauchly's Test of SphericityaMauchly's Test of Sphericity, table, Measure, Time, 1 layers, 2 levels of column headers and 1 levels of row headers, table with 8 columns and 8 rows

|  |  |  |  |  |  |  |  |
| --- | --- | --- | --- | --- | --- | --- | --- |
| |  |  |  | | --- | --- | --- | | Measure: | Time | Time | | | | | | | | |
|  |  |  |  |  |  |  |  |
| --- | --- | --- | --- | --- | --- | --- | --- |
| Within Subjects Effect | Mauchly's W | Approx. Chi-Square | df | Sig. | Epsilonb | | |
|  |  |  |  |  | Greenhouse-Geisser | Huynh-Feldt | Lower-bound |
| Visit | .511 | 4.516 | 5 | .482 | .739 | 1.000 | .333 |
|  |  |  |  |  |  |  |  |
| --- | --- | --- | --- | --- | --- | --- | --- |
| Tests the null hypothesis that the error covariance matrix of the orthonormalized transformed dependent variables is proportional to an identity matrix. | | | | | | | |
| a. Design: Intercept + nystagmus + Eyemusclesurgery + Srabismus + nystagmus \* Eyemusclesurgery + nystagmus \* Srabismus + Eyemusclesurgery \* Srabismus + nystagmus \* Eyemusclesurgery \* Srabismus   Within Subjects Design: Visit | | | | | | | |
| b. May be used to adjust the degrees of freedom for the averaged tests of significance. Corrected tests are displayed in the Tests of Within-Subjects Effects table. | | | | | | | |
|  |  |  |  |  |  |  |  |

General Linear Model  
General Linear Model - Tests of Within-Subjects Effects - May 11, 2022

Tests of Within-Subjects EffectsTests of Within-Subjects Effects, table, Measure, Time, 1 layers, 1 levels of column headers and 2 levels of row headers, table with 7 columns and 39 rows

|  |  |  |  |  |  |  |
| --- | --- | --- | --- | --- | --- | --- |
| |  |  |  | | --- | --- | --- | | Measure: | Time | Time | | | | | | | |
|  |  |  |  |  |  |  |
| --- | --- | --- | --- | --- | --- | --- |
| Source | | Type III Sum of Squares | df | Mean Square | F | Sig. |
| Visit | Sphericity Assumed | .104 | 3 | .035 | 7.247 | .001 |
|  | Greenhouse-Geisser | .104 | 2.217 | .047 | 7.247 | .004 |
|  | Huynh-Feldt | .104 | 3.000 | .035 | 7.247 | .001 |
|  | Lower-bound | .104 | 1.000 | .104 | 7.247 | .027 |
| Visit \* nystagmus | Sphericity Assumed | .000 | 0 | . | . | . |
|  | Greenhouse-Geisser | .000 | .000 | . | . | . |
|  | Huynh-Feldt | .000 | .000 | . | . | . |
|  | Lower-bound | .000 | .000 | . | . | . |
| Visit \* Eyemusclesurgery | Sphericity Assumed | .001 | 3 | .000 | .086 | .967 |
|  | Greenhouse-Geisser | .001 | 2.217 | .001 | .086 | .933 |
|  | Huynh-Feldt | .001 | 3.000 | .000 | .086 | .967 |
|  | Lower-bound | .001 | 1.000 | .001 | .086 | .777 |
| Visit \* Srabismus | Sphericity Assumed | .014 | 6 | .002 | .478 | .818 |
|  | Greenhouse-Geisser | .014 | 4.434 | .003 | .478 | .769 |
|  | Huynh-Feldt | .014 | 6.000 | .002 | .478 | .818 |
|  | Lower-bound | .014 | 2.000 | .007 | .478 | .637 |
| Visit \* nystagmus \* Eyemusclesurgery | Sphericity Assumed | .000 | 0 | . | . | . |
|  | Greenhouse-Geisser | .000 | .000 | . | . | . |
|  | Huynh-Feldt | .000 | .000 | . | . | . |
|  | Lower-bound | .000 | .000 | . | . | . |
| Visit \* nystagmus \* Srabismus | Sphericity Assumed | .000 | 0 | . | . | . |
|  | Greenhouse-Geisser | .000 | .000 | . | . | . |
|  | Huynh-Feldt | .000 | .000 | . | . | . |
|  | Lower-bound | .000 | .000 | . | . | . |
| Visit \* Eyemusclesurgery \* Srabismus | Sphericity Assumed | .011 | 6 | .002 | .367 | .892 |
|  | Greenhouse-Geisser | .011 | 4.434 | .002 | .367 | .846 |
|  | Huynh-Feldt | .011 | 6.000 | .002 | .367 | .892 |
|  | Lower-bound | .011 | 2.000 | .005 | .367 | .704 |
| Visit \* nystagmus \* Eyemusclesurgery \* Srabismus | Sphericity Assumed | .000 | 0 | . | . | . |
|  | Greenhouse-Geisser | .000 | .000 | . | . | . |
|  | Huynh-Feldt | .000 | .000 | . | . | . |
|  | Lower-bound | .000 | .000 | . | . | . |
| Error(Visit) | Sphericity Assumed | .115 | 24 | .005 |  |  |
|  | Greenhouse-Geisser | .115 | 17.735 | .006 |  |  |
|  | Huynh-Feldt | .115 | 24.000 | .005 |  |  |
|  | Lower-bound | .115 | 8.000 | .014 |  |  |
|  |  |  |  |  |  |  |

General Linear Model  
General Linear Model - Tests of Within-Subjects Contrasts - May 11, 2022

Tests of Within-Subjects ContrastsTests of Within-Subjects Contrasts, table, Measure, Time, 1 layers, 1 levels of column headers and 2 levels of row headers, table with 7 columns and 30 rows

|  |  |  |  |  |  |  |
| --- | --- | --- | --- | --- | --- | --- |
| |  |  |  | | --- | --- | --- | | Measure: | Time | Time | | | | | | | |
|  |  |  |  |  |  |  |
| --- | --- | --- | --- | --- | --- | --- |
| Source | Visit | Type III Sum of Squares | df | Mean Square | F | Sig. |
| Visit | Linear | .100 | 1 | .100 | 12.382 | .008 |
|  | Quadratic | .003 | 1 | .003 | 1.404 | .270 |
|  | Cubic | .001 | 1 | .001 | .238 | .639 |
| Visit \* nystagmus | Linear | .000 | 0 | . | . | . |
|  | Quadratic | .000 | 0 | . | . | . |
|  | Cubic | .000 | 0 | . | . | . |
| Visit \* Eyemusclesurgery | Linear | 2.299E-6 | 1 | 2.299E-6 | .000 | .987 |
|  | Quadratic | 1.149E-5 | 1 | 1.149E-5 | .005 | .945 |
|  | Cubic | .001 | 1 | .001 | .300 | .599 |
| Visit \* Srabismus | Linear | .010 | 2 | .005 | .639 | .553 |
|  | Quadratic | 2.939E-5 | 2 | 1.469E-5 | .007 | .993 |
|  | Cubic | .003 | 2 | .002 | .416 | .673 |
| Visit \* nystagmus \* Eyemusclesurgery | Linear | .000 | 0 | . | . | . |
|  | Quadratic | .000 | 0 | . | . | . |
|  | Cubic | .000 | 0 | . | . | . |
| Visit \* nystagmus \* Srabismus | Linear | .000 | 0 | . | . | . |
|  | Quadratic | .000 | 0 | . | . | . |
|  | Cubic | .000 | 0 | . | . | . |
| Visit \* Eyemusclesurgery \* Srabismus | Linear | .000 | 2 | .000 | .029 | .972 |
|  | Quadratic | .007 | 2 | .003 | 1.539 | .272 |
|  | Cubic | .003 | 2 | .002 | .397 | .685 |
| Visit \* nystagmus \* Eyemusclesurgery \* Srabismus | Linear | .000 | 0 | . | . | . |
|  | Quadratic | .000 | 0 | . | . | . |
|  | Cubic | .000 | 0 | . | . | . |
| Error(Visit) | Linear | .065 | 8 | .008 |  |  |
|  | Quadratic | .018 | 8 | .002 |  |  |
|  | Cubic | .032 | 8 | .004 |  |  |
|  |  |  |  |  |  |  |

General Linear Model  
General Linear Model - Tests of Between-Subjects Effects - May 11, 2022

Tests of Between-Subjects EffectsTests of Between-Subjects Effects, table, Measure, Time, Transformed Variable, Average, 1 layers, 1 levels of column headers and 1 levels of row headers, table with 6 columns and 13 rows

|  |  |  |  |  |  |  |  |  |  |  |  |
| --- | --- | --- | --- | --- | --- | --- | --- | --- | --- | --- | --- |
| |  |  |  | | --- | --- | --- | | Measure: | Time | Time | | Transformed Variable: | Average | Average | | | | | | |
|  |  |  |  |  |  |
| --- | --- | --- | --- | --- | --- |
| Source | Type III Sum of Squares | df | Mean Square | F | Sig. |
| Intercept | 15.162 | 1 | 15.162 | 153.308 | .000 |
| nystagmus | .000 | 0 | . | . | . |
| Eyemusclesurgery | .010 | 1 | .010 | .098 | .763 |
| Srabismus | .159 | 2 | .080 | .805 | .480 |
| nystagmus \* Eyemusclesurgery | .000 | 0 | . | . | . |
| nystagmus \* Srabismus | .000 | 0 | . | . | . |
| Eyemusclesurgery \* Srabismus | .486 | 2 | .243 | 2.456 | .147 |
| nystagmus \* Eyemusclesurgery \* Srabismus | .000 | 0 | . | . | . |
| Error | .791 | 8 | .099 |  |  |
|  |  |  |  |  |  |

Log  
Log - Log - May 11, 2022

GLM V1LogMar V2LogMar V3LogMar V4LogMar BY RE\_Myopia LE\_Myopia  
  /WSFACTOR=Visit 4 Polynomial  
  /MEASURE=Time  
  /METHOD=SSTYPE(3)  
  /CRITERIA=ALPHA(.05)  
  /WSDESIGN=Visit  
  /DESIGN=RE\_Myopia LE\_Myopia RE\_Myopia\*LE\_Myopia.

General Linear Model  
General Linear Model - Within-Subjects Factors - May 11, 2022

Within-Subjects FactorsWithin-Subjects Factors, table, Measure, Time, 1 layers, 1 levels of column headers and 1 levels of row headers, table with 2 columns and 7 rows

|  |  |  |  |  |
| --- | --- | --- | --- | --- |
| |  |  |  | | --- | --- | --- | | Measure: | Time | Time | | |
|  |  |
| --- | --- |
| Visit | Dependent Variable |
| 1 | V1LogMar |
| 2 | V2LogMar |
| 3 | V3LogMar |
| 4 | V4LogMar |
|  |  |

General Linear Model  
General Linear Model - Between-Subjects Factors - May 11, 2022

Between-Subjects FactorsBetween-Subjects Factors, table, 1 levels of column headers and 2 levels of row headers, table with 4 columns and 6 rows

|  |  |  |  |
| --- | --- | --- | --- |
|  | | Value Label | N |
| RE\_Myopia | 1 | Hyperm | 11 |
|  | 2 | Myopia | 3 |
| LE\_Myopia | 1 | Hyperm | 11 |
|  | 2 | Myopia | 3 |
|  |  |  |  |

General Linear Model  
General Linear Model - Multivariate Tests - May 11, 2022

Multivariate TestsaMultivariate Tests, table, 1 levels of column headers and 2 levels of row headers, table with 7 columns and 20 rows

|  |  |  |  |  |  |  |
| --- | --- | --- | --- | --- | --- | --- |
| Effect | | Value | F | Hypothesis df | Error df | Sig. |
| Visit | Pillai's Trace | .494 | 3.254b | 3.000 | 10.000 | .068 |
|  | Wilks' Lambda | .506 | 3.254b | 3.000 | 10.000 | .068 |
|  | Hotelling's Trace | .976 | 3.254b | 3.000 | 10.000 | .068 |
|  | Roy's Largest Root | .976 | 3.254b | 3.000 | 10.000 | .068 |
| Visit \* RE\_Myopia | Pillai's Trace | .000 | .b | .000 | .000 | . |
|  | Wilks' Lambda | 1.000 | .b | .000 | 11.000 | . |
|  | Hotelling's Trace | .000 | .b | .000 | 2.000 | . |
|  | Roy's Largest Root | .000 | .000b | 3.000 | 9.000 | 1.000 |
| Visit \* LE\_Myopia | Pillai's Trace | .000 | .b | .000 | .000 | . |
|  | Wilks' Lambda | 1.000 | .b | .000 | 11.000 | . |
|  | Hotelling's Trace | .000 | .b | .000 | 2.000 | . |
|  | Roy's Largest Root | .000 | .000b | 3.000 | 9.000 | 1.000 |
| Visit \* RE\_Myopia \* LE\_Myopia | Pillai's Trace | .000 | .b | .000 | .000 | . |
|  | Wilks' Lambda | 1.000 | .b | .000 | 11.000 | . |
|  | Hotelling's Trace | .000 | .b | .000 | 2.000 | . |
|  | Roy's Largest Root | .000 | .000b | 3.000 | 9.000 | 1.000 |
|  |  |  |  |  |  |  |
| --- | --- | --- | --- | --- | --- | --- |
| a. Design: Intercept + RE\_Myopia + LE\_Myopia + RE\_Myopia \* LE\_Myopia   Within Subjects Design: Visit | | | | | | |
| b. Exact statistic | | | | | | |
|  |  |  |  |  |  |  |

General Linear Model  
General Linear Model - Mauchly's Test of Sphericity - May 11, 2022

Mauchly's Test of SphericityaMauchly's Test of Sphericity, table, Measure, Time, 1 layers, 2 levels of column headers and 1 levels of row headers, table with 8 columns and 8 rows

|  |  |  |  |  |  |  |  |
| --- | --- | --- | --- | --- | --- | --- | --- |
| |  |  |  | | --- | --- | --- | | Measure: | Time | Time | | | | | | | | |
|  |  |  |  |  |  |  |  |
| --- | --- | --- | --- | --- | --- | --- | --- |
| Within Subjects Effect | Mauchly's W | Approx. Chi-Square | df | Sig. | Epsilonb | | |
|  |  |  |  |  | Greenhouse-Geisser | Huynh-Feldt | Lower-bound |
| Visit | .469 | 8.129 | 5 | .151 | .752 | 1.000 | .333 |
|  |  |  |  |  |  |  |  |
| --- | --- | --- | --- | --- | --- | --- | --- |
| Tests the null hypothesis that the error covariance matrix of the orthonormalized transformed dependent variables is proportional to an identity matrix. | | | | | | | |
| a. Design: Intercept + RE\_Myopia + LE\_Myopia + RE\_Myopia \* LE\_Myopia   Within Subjects Design: Visit | | | | | | | |
| b. May be used to adjust the degrees of freedom for the averaged tests of significance. Corrected tests are displayed in the Tests of Within-Subjects Effects table. | | | | | | | |
|  |  |  |  |  |  |  |  |

General Linear Model  
General Linear Model - Tests of Within-Subjects Effects - May 11, 2022

Tests of Within-Subjects EffectsTests of Within-Subjects Effects, table, Measure, Time, 1 layers, 1 levels of column headers and 2 levels of row headers, table with 7 columns and 23 rows

|  |  |  |  |  |  |  |
| --- | --- | --- | --- | --- | --- | --- |
| |  |  |  | | --- | --- | --- | | Measure: | Time | Time | | | | | | | |
|  |  |  |  |  |  |  |
| --- | --- | --- | --- | --- | --- | --- |
| Source | | Type III Sum of Squares | df | Mean Square | F | Sig. |
| Visit | Sphericity Assumed | .063 | 3 | .021 | 6.358 | .001 |
|  | Greenhouse-Geisser | .063 | 2.257 | .028 | 6.358 | .004 |
|  | Huynh-Feldt | .063 | 3.000 | .021 | 6.358 | .001 |
|  | Lower-bound | .063 | 1.000 | .063 | 6.358 | .027 |
| Visit \* RE\_Myopia | Sphericity Assumed | .000 | 0 | . | . | . |
|  | Greenhouse-Geisser | .000 | .000 | . | . | . |
|  | Huynh-Feldt | .000 | .000 | . | . | . |
|  | Lower-bound | .000 | .000 | . | . | . |
| Visit \* LE\_Myopia | Sphericity Assumed | .000 | 0 | . | . | . |
|  | Greenhouse-Geisser | .000 | .000 | . | . | . |
|  | Huynh-Feldt | .000 | .000 | . | . | . |
|  | Lower-bound | .000 | .000 | . | . | . |
| Visit \* RE\_Myopia \* LE\_Myopia | Sphericity Assumed | .000 | 0 | . | . | . |
|  | Greenhouse-Geisser | .000 | .000 | . | . | . |
|  | Huynh-Feldt | .000 | .000 | . | . | . |
|  | Lower-bound | .000 | .000 | . | . | . |
| Error(Visit) | Sphericity Assumed | .119 | 36 | .003 |  |  |
|  | Greenhouse-Geisser | .119 | 27.087 | .004 |  |  |
|  | Huynh-Feldt | .119 | 36.000 | .003 |  |  |
|  | Lower-bound | .119 | 12.000 | .010 |  |  |
|  |  |  |  |  |  |  |

General Linear Model  
General Linear Model - Tests of Within-Subjects Contrasts - May 11, 2022

Tests of Within-Subjects ContrastsTests of Within-Subjects Contrasts, table, Measure, Time, 1 layers, 1 levels of column headers and 2 levels of row headers, table with 7 columns and 18 rows

|  |  |  |  |  |  |  |
| --- | --- | --- | --- | --- | --- | --- |
| |  |  |  | | --- | --- | --- | | Measure: | Time | Time | | | | | | | |
|  |  |  |  |  |  |  |
| --- | --- | --- | --- | --- | --- | --- |
| Source | Visit | Type III Sum of Squares | df | Mean Square | F | Sig. |
| Visit | Linear | .054 | 1 | .054 | 10.007 | .008 |
|  | Quadratic | .002 | 1 | .002 | 1.895 | .194 |
|  | Cubic | .007 | 1 | .007 | 2.069 | .176 |
| Visit \* RE\_Myopia | Linear | .000 | 0 | . | . | . |
|  | Quadratic | .000 | 0 | . | . | . |
|  | Cubic | .000 | 0 | . | . | . |
| Visit \* LE\_Myopia | Linear | .000 | 0 | . | . | . |
|  | Quadratic | .000 | 0 | . | . | . |
|  | Cubic | .000 | 0 | . | . | . |
| Visit \* RE\_Myopia \* LE\_Myopia | Linear | .000 | 0 | . | . | . |
|  | Quadratic | .000 | 0 | . | . | . |
|  | Cubic | .000 | 0 | . | . | . |
| Error(Visit) | Linear | .065 | 12 | .005 |  |  |
|  | Quadratic | .015 | 12 | .001 |  |  |
|  | Cubic | .039 | 12 | .003 |  |  |
|  |  |  |  |  |  |  |

General Linear Model  
General Linear Model - Tests of Between-Subjects Effects - May 11, 2022

Tests of Between-Subjects EffectsTests of Between-Subjects Effects, table, Measure, Time, Transformed Variable, Average, 1 layers, 1 levels of column headers and 1 levels of row headers, table with 6 columns and 9 rows

|  |  |  |  |  |  |  |  |  |  |  |  |
| --- | --- | --- | --- | --- | --- | --- | --- | --- | --- | --- | --- |
| |  |  |  | | --- | --- | --- | | Measure: | Time | Time | | Transformed Variable: | Average | Average | | | | | | |
|  |  |  |  |  |  |
| --- | --- | --- | --- | --- | --- |
| Source | Type III Sum of Squares | df | Mean Square | F | Sig. |
| Intercept | 17.591 | 1 | 17.591 | 149.698 | .000 |
| RE\_Myopia | .000 | 0 | . | . | . |
| LE\_Myopia | .000 | 0 | . | . | . |
| RE\_Myopia \* LE\_Myopia | .000 | 0 | . | . | . |
| Error | 1.410 | 12 | .118 |  |  |
|  |  |  |  |  |  |

Log  
Log - Log - May 11, 2022

SPLIT FILE OFF.  
FILTER OFF.  
USE ALL.  
EXECUTE.  
EXAMINE VARIABLES=V1LogMar V2LogMar V3LogMar V4LogMar  
  /PLOT NPPLOT  
  /STATISTICS NONE  
  /CINTERVAL 95  
  /MISSING LISTWISE  
  /NOTOTAL.

Explore  
Explore - Case Processing Summary - May 11, 2022

Case Processing SummaryCase Processing Summary, table, 3 levels of column headers and 1 levels of row headers, table with 7 columns and 8 rows

|  |  |  |  |  |  |  |
| --- | --- | --- | --- | --- | --- | --- |
|  | Cases | | | | | |
|  | Valid | | Missing | | Total | |
|  | N | Percent | N | Percent | N | Percent |
| V1 LogMar | 17 | 16.2% | 88 | 83.8% | 105 | 100.0% |
| V2 LogMar | 17 | 16.2% | 88 | 83.8% | 105 | 100.0% |
| V3 LogMar | 17 | 16.2% | 88 | 83.8% | 105 | 100.0% |
| V4 LogMar | 17 | 16.2% | 88 | 83.8% | 105 | 100.0% |
|  |  |  |  |  |  |  |

Explore  
Explore - Tests of Normality - May 11, 2022

Tests of NormalityTests of Normality, table, 2 levels of column headers and 1 levels of row headers, table with 7 columns and 8 rows

|  |  |  |  |  |  |  |
| --- | --- | --- | --- | --- | --- | --- |
|  | Kolmogorov-Smirnova | | | Shapiro-Wilk | | |
|  | Statistic | df | Sig. | Statistic | df | Sig. |
| V1 LogMar | .242 | 17 | .009 | .857 | 17 | .014 |
| V2 LogMar | .206 | 17 | .054 | .874 | 17 | .025 |
| V3 LogMar | .231 | 17 | .016 | .854 | 17 | .012 |
| V4 LogMar | .198 | 17 | .076 | .878 | 17 | .030 |
|  |  |  |  |  |  |  |
| --- | --- | --- | --- | --- | --- | --- |
| a. Lilliefors Significance Correction | | | | | | |
|  |  |  |  |  |  |  |

V1 LogMar  
V1 LogMar - Normal Q-Q Plot - May 11, 2022

V1 LogMar  
V1 LogMar - Detrended Normal Q-Q Plot - May 11, 2022

{"copyright":"(C) Copyright IBM Corp. 2011","grammar":[{"elements":[{"data":{"$ref":"dSource"},"style":{"symbol":"circle","outline":{"r":0,"b":157,"g":100},"size":6.6666665,"fill":{"r":119,"b":119,"g":118}},"position":[{"field":{"$ref":"fVariable1"}},{"field":{"$ref":"fVariable"}}],"type":"point"},{"data":{"$ref":"dSource"},"style":{"fill":{"r":0,"b":157,"g":100},"stroke":{"width":3.3333333}},"position":[{"value":"0"},{"field":{"$ref":"fVariable"}}],"type":"line"}],"coordinates":{"style":{"outline":{"r":0,"b":157,"g":100},"fill":{"r":255,"b":255,"g":255}},"dimensions":[{"scale":{"padding":{"left":"5%","right":"5%"}},"axis":[{"tickStyle":{"padding":5.0,"fill":{"r":0,"b":157,"g":100},"font":{"size":"8pt","weight":"normal","family":"sans-serif"}},"gridStyle":{"fill":{"r":0,"b":157,"g":100}},"lineStyle":{"fill":{"r":0,"b":157,"g":100},"stroke":{"width":0.6666667}},"titleStyle":{"padding":6.0,"fill":{"r":0,"b":157,"g":100},"font":{"size":"12pt","weight":"bold","family":"sans-serif"}},"title":["Dev from Normal"],"markStyle":{"fill":{"a":0,"r":0,"b":157,"g":100},"stroke":{"width":1.3333334}}}]},{"scale":{"padding":{"left":"5%","right":"5%"}},"axis":[{"tickStyle":{"padding":5.0,"fill":{"r":0,"b":157,"g":100},"font":{"size":"8pt","weight":"normal","family":"sans-serif"}},"gridStyle":{"fill":{"r":0,"b":157,"g":100}},"lineStyle":{"fill":{"r":0,"b":157,"g":100},"stroke":{"width":0.6666667}},"titleStyle":{"padding":6.0,"fill":{"r":0,"b":157,"g":100},"font":{"size":"12pt","weight":"bold","family":"sans-serif"}},"title":["Observed Value"],"markStyle":{"fill":{"a":0,"r":0,"b":157,"g":100},"stroke":{"width":1.3333334}}}]}]}}],"data":[{"id":"dSource","fields":[{"min":0.0,"max":1.0,"id":"fVariable","label":"X Variable\_1"},{"min":-0.6826729287829785,"max":0.3565657489635613,"id":"fVariable1","label":"Y Axis"},{"min":1.0,"max":101.0,"id":"fVariable2","label":"Case Number"}],"rows":[[0,-0.5822869566945978,3],[0,-0.5822869566945978,1],[0.1,-0.6826729287829785,15],[0.5,0.2851416109536289,101],[0.5,0.2851416109536289,95],[0.6,0.3565657489635613,41],[0.7,0.2410250385786605,96],[0.7,0.2410250385786605,89],[0.7,0.2410250385786605,75],[0.7,0.2410250385786605,58],[0.7,0.2410250385786605,51],[0.8,0.04772356837986103,100],[0.8,0.04772356837986103,46],[0.9,0.009764096225602037,43],[0.9,0.009764096225602037,39],[1,-0.1964093217903071,98],[1,-0.1964093217903071,61]]}],"size":{"width":850.0,"height":500.0},"style":{"outline":{"a":0.0,"r":0,"b":0,"g":0},"fill":{"r":255,"b":255,"g":255}},"titles":[{"backgroundStyle":{"outline":{"a":0.0,"r":0,"b":0,"g":0},"fill":{"a":0.0,"r":0,"b":0,"g":0}},"style":{"padding":3.0,"fill":{"r":0,"b":157,"g":100},"font":{"size":"12pt","weight":"bold","family":"sans-serif"}},"type":"title","content":["Detrended Normal Q-Q Plot of V1 LogMar"]}],"version":"6.0"}

V2 LogMar  
V2 LogMar - Normal Q-Q Plot - May 11, 2022

V2 LogMar  
V2 LogMar - Detrended Normal Q-Q Plot - May 11, 2022

{"copyright":"(C) Copyright IBM Corp. 2011","grammar":[{"elements":[{"data":{"$ref":"dSource"},"style":{"symbol":"circle","outline":{"r":0,"b":157,"g":100},"size":6.6666665,"fill":{"r":119,"b":119,"g":118}},"position":[{"field":{"$ref":"fVariable1"}},{"field":{"$ref":"fVariable"}}],"type":"point"},{"data":{"$ref":"dSource"},"style":{"fill":{"r":0,"b":157,"g":100},"stroke":{"width":3.3333333}},"position":[{"value":"0"},{"field":{"$ref":"fVariable"}}],"type":"line"}],"coordinates":{"style":{"outline":{"r":0,"b":157,"g":100},"fill":{"r":255,"b":255,"g":255}},"dimensions":[{"scale":{"padding":{"left":"5%","right":"5%"}},"axis":[{"tickStyle":{"padding":5.0,"fill":{"r":0,"b":157,"g":100},"font":{"size":"8pt","weight":"normal","family":"sans-serif"}},"gridStyle":{"fill":{"r":0,"b":157,"g":100}},"lineStyle":{"fill":{"r":0,"b":157,"g":100},"stroke":{"width":0.6666667}},"titleStyle":{"padding":6.0,"fill":{"r":0,"b":157,"g":100},"font":{"size":"12pt","weight":"bold","family":"sans-serif"}},"title":["Dev from Normal"],"markStyle":{"fill":{"a":0,"r":0,"b":157,"g":100},"stroke":{"width":1.3333334}}}]},{"scale":{"padding":{"left":"5%","right":"5%"}},"axis":[{"tickStyle":{"padding":5.0,"fill":{"r":0,"b":157,"g":100},"font":{"size":"8pt","weight":"normal","family":"sans-serif"}},"gridStyle":{"fill":{"r":0,"b":157,"g":100}},"lineStyle":{"fill":{"r":0,"b":157,"g":100},"stroke":{"width":0.6666667}},"titleStyle":{"padding":6.0,"fill":{"r":0,"b":157,"g":100},"font":{"size":"12pt","weight":"bold","family":"sans-serif"}},"title":["Observed Value"],"markStyle":{"fill":{"a":0,"r":0,"b":157,"g":100},"stroke":{"width":1.3333334}}}]}]}}],"data":[{"id":"dSource","fields":[{"min":0.0,"max":1.0,"id":"fVariable","label":"X Variable\_1"},{"min":-0.6442243619490591,"max":0.2822161470625081,"id":"fVariable1","label":"Y Axis"},{"min":1.0,"max":101.0,"id":"fVariable2","label":"Case Number"}],"rows":[[0,-0.5509809865602737,3],[0,-0.5509809865602737,1],[0.1,-0.6442243619490591,15],[0.4,0.1200513025660832,101],[0.5,0.2671266122396265,41],[0.6,0.2822161470625081,96],[0.6,0.2822161470625081,95],[0.6,0.2822161470625081,51],[0.7,0.2526442652916964,89],[0.7,0.2526442652916964,75],[0.8,0.1361703121109479,100],[0.8,0.1361703121109479,98],[0.8,0.1361703121109479,58],[0.8,0.1361703121109479,43],[0.9,-0.1183373512463029,46],[0.9,-0.1183373512463029,39],[1,-0.3039020755824415,61]]}],"size":{"width":850.0,"height":500.0},"style":{"outline":{"a":0.0,"r":0,"b":0,"g":0},"fill":{"r":255,"b":255,"g":255}},"titles":[{"backgroundStyle":{"outline":{"a":0.0,"r":0,"b":0,"g":0},"fill":{"a":0.0,"r":0,"b":0,"g":0}},"style":{"padding":3.0,"fill":{"r":0,"b":157,"g":100},"font":{"size":"12pt","weight":"bold","family":"sans-serif"}},"type":"title","content":["Detrended Normal Q-Q Plot of V2 LogMar"]}],"version":"6.0"}

V3 LogMar  
V3 LogMar - Normal Q-Q Plot - May 11, 2022

V3 LogMar  
V3 LogMar - Detrended Normal Q-Q Plot - May 11, 2022

{"copyright":"(C) Copyright IBM Corp. 2011","grammar":[{"elements":[{"data":{"$ref":"dSource"},"style":{"symbol":"circle","outline":{"r":0,"b":157,"g":100},"size":6.6666665,"fill":{"r":119,"b":119,"g":118}},"position":[{"field":{"$ref":"fVariable1"}},{"field":{"$ref":"fVariable"}}],"type":"point"},{"data":{"$ref":"dSource"},"style":{"fill":{"r":0,"b":157,"g":100},"stroke":{"width":3.3333333}},"position":[{"value":"0"},{"field":{"$ref":"fVariable"}}],"type":"line"}],"coordinates":{"style":{"outline":{"r":0,"b":157,"g":100},"fill":{"r":255,"b":255,"g":255}},"dimensions":[{"scale":{"padding":{"left":"5%","right":"5%"}},"axis":[{"tickStyle":{"padding":5.0,"fill":{"r":0,"b":157,"g":100},"font":{"size":"8pt","weight":"normal","family":"sans-serif"}},"gridStyle":{"fill":{"r":0,"b":157,"g":100}},"lineStyle":{"fill":{"r":0,"b":157,"g":100},"stroke":{"width":0.6666667}},"titleStyle":{"padding":6.0,"fill":{"r":0,"b":157,"g":100},"font":{"size":"12pt","weight":"bold","family":"sans-serif"}},"title":["Dev from Normal"],"markStyle":{"fill":{"a":0,"r":0,"b":157,"g":100},"stroke":{"width":1.3333334}}}]},{"scale":{"padding":{"left":"5%","right":"5%"}},"axis":[{"tickStyle":{"padding":5.0,"fill":{"r":0,"b":157,"g":100},"font":{"size":"8pt","weight":"normal","family":"sans-serif"}},"gridStyle":{"fill":{"r":0,"b":157,"g":100}},"lineStyle":{"fill":{"r":0,"b":157,"g":100},"stroke":{"width":0.6666667}},"titleStyle":{"padding":6.0,"fill":{"r":0,"b":157,"g":100},"font":{"size":"12pt","weight":"bold","family":"sans-serif"}},"title":["Observed Value"],"markStyle":{"fill":{"a":0,"r":0,"b":157,"g":100},"stroke":{"width":1.3333334}}}]}]}}],"data":[{"id":"dSource","fields":[{"min":0.0,"max":0.9,"id":"fVariable","label":"X Variable\_1"},{"min":-0.5400958229634141,"max":0.2825211608670095,"id":"fVariable1","label":"Y Axis"},{"min":1.0,"max":101.0,"id":"fVariable2","label":"Case Number"}],"rows":[[0,-0.5400958229634141,15],[0,-0.5400958229634141,3],[0,-0.5400958229634141,1],[0.3,-0.009240291844717752,101],[0.4,0.06346682887147137,95],[0.4,0.06346682887147137,41],[0.6,0.2825211608670095,96],[0.6,0.2825211608670095,89],[0.6,0.2825211608670095,75],[0.6,0.2825211608670095,51],[0.7,0.2595488288792651,46],[0.8,0.105984037548606,100],[0.8,0.105984037548606,98],[0.8,0.105984037548606,61],[0.8,0.105984037548606,58],[0.8,0.105984037548606,43],[0.9,-0.3935963712948372,39]]}],"size":{"width":850.0,"height":500.0},"style":{"outline":{"a":0.0,"r":0,"b":0,"g":0},"fill":{"r":255,"b":255,"g":255}},"titles":[{"backgroundStyle":{"outline":{"a":0.0,"r":0,"b":0,"g":0},"fill":{"a":0.0,"r":0,"b":0,"g":0}},"style":{"padding":3.0,"fill":{"r":0,"b":157,"g":100},"font":{"size":"12pt","weight":"bold","family":"sans-serif"}},"type":"title","content":["Detrended Normal Q-Q Plot of V3 LogMar"]}],"version":"6.0"}

V4 LogMar  
V4 LogMar - Normal Q-Q Plot - May 11, 2022

V4 LogMar  
V4 LogMar - Detrended Normal Q-Q Plot - May 11, 2022

{"copyright":"(C) Copyright IBM Corp. 2011","grammar":[{"elements":[{"data":{"$ref":"dSource"},"style":{"symbol":"circle","outline":{"r":0,"b":157,"g":100},"size":6.6666665,"fill":{"r":119,"b":119,"g":118}},"position":[{"field":{"$ref":"fVariable1"}},{"field":{"$ref":"fVariable"}}],"type":"point"},{"data":{"$ref":"dSource"},"style":{"fill":{"r":0,"b":157,"g":100},"stroke":{"width":3.3333333}},"position":[{"value":"0"},{"field":{"$ref":"fVariable"}}],"type":"line"}],"coordinates":{"style":{"outline":{"r":0,"b":157,"g":100},"fill":{"r":255,"b":255,"g":255}},"dimensions":[{"scale":{"padding":{"left":"5%","right":"5%"}},"axis":[{"tickStyle":{"padding":5.0,"fill":{"r":0,"b":157,"g":100},"font":{"size":"8pt","weight":"normal","family":"sans-serif"}},"gridStyle":{"fill":{"r":0,"b":157,"g":100}},"lineStyle":{"fill":{"r":0,"b":157,"g":100},"stroke":{"width":0.6666667}},"titleStyle":{"padding":6.0,"fill":{"r":0,"b":157,"g":100},"font":{"size":"12pt","weight":"bold","family":"sans-serif"}},"title":["Dev from Normal"],"markStyle":{"fill":{"a":0,"r":0,"b":157,"g":100},"stroke":{"width":1.3333334}}}]},{"scale":{"padding":{"left":"5%","right":"5%"}},"axis":[{"tickStyle":{"padding":5.0,"fill":{"r":0,"b":157,"g":100},"font":{"size":"8pt","weight":"normal","family":"sans-serif"}},"gridStyle":{"fill":{"r":0,"b":157,"g":100}},"lineStyle":{"fill":{"r":0,"b":157,"g":100},"stroke":{"width":0.6666667}},"titleStyle":{"padding":6.0,"fill":{"r":0,"b":157,"g":100},"font":{"size":"12pt","weight":"bold","family":"sans-serif"}},"title":["Observed Value"],"markStyle":{"fill":{"a":0,"r":0,"b":157,"g":100},"stroke":{"width":1.3333334}}}]}]}}],"data":[{"id":"dSource","fields":[{"min":0.0,"max":0.9,"id":"fVariable","label":"X Variable\_1"},{"min":-0.529509956976564,"max":0.3916005411765027,"id":"fVariable1","label":"Y Axis"},{"min":1.0,"max":101.0,"id":"fVariable2","label":"Case Number"}],"rows":[[0,-0.529509956976564,15],[0,-0.529509956976564,3],[0,-0.529509956976564,1],[0.3,0.02885102247405968,101],[0.35,0.02264575562271542,41],[0.4,0.03296586615365882,95],[0.55,0.3916005411765027,96],[0.6,0.2784330031992587,89],[0.6,0.2784330031992587,75],[0.6,0.2784330031992587,51],[0.7,0.1858029220743301,98],[0.7,0.1858029220743301,61],[0.7,0.1858029220743301,58],[0.8,-0.0127941265613849,100],[0.8,-0.0127941265613849,46],[0.8,-0.0127941265613849,43],[0.9,-0.3004941603122049,39]]}],"size":{"width":850.0,"height":500.0},"style":{"outline":{"a":0.0,"r":0,"b":0,"g":0},"fill":{"r":255,"b":255,"g":255}},"titles":[{"backgroundStyle":{"outline":{"a":0.0,"r":0,"b":0,"g":0},"fill":{"a":0.0,"r":0,"b":0,"g":0}},"style":{"padding":3.0,"fill":{"r":0,"b":157,"g":100},"font":{"size":"12pt","weight":"bold","family":"sans-serif"}},"type":"title","content":["Detrended Normal Q-Q Plot of V4 LogMar"]}],"version":"6.0"}

IBM SPSS Web Report

X

ABOUT

|  |
| --- |
| Created Using: IBM SPSS Statistics 26 |
| Creation Date: May 11, 2022 |
| Document Version: OriginalSaved Copy |
| Saved Date:  May 11, 2022 |

Navigation Controls

|  |
| --- |
| Contents - Opens and closes the list of charts and tables in the Web Report |
| Next & Previous - Display the next or previous table or chart in the Web Report |
| Help - Opens Help |

Toolbar Buttons

|  |  |
| --- | --- |
|  | Undo - Undoes the last change in the document. |
|  | Edit - Open the Editor tool for tables and charts. Certain editing options are only available when you are connected to an Internet server. |
|  | Save - Creates a new copy of the Web Report with the saved changes. |
|  | Print - Prints the current object when in Object View and all objects in Page View. |
|  | Page View - Switches the Web Report to display all the tables and charts on a single page. |
|  | Object View - Switches the Web Report so that each table or chart is displayed one at a time. |

Connecting to a Server

:   The status of the Web Report's connection to an Internet server appears in the top right corner of the Web Report.
:   An Internet connection is not required to open a Web Report. With a saved copy of the Web Report you can view all of the charts and tables, and have some limited editing ability, when not connected to the Internet.
:   Connecting a Web Report to an Internet server will enable far greater editing capabilities for tables and for charts.

- If the author specified an Internet server when they created the Web Report, the Web Report will attempt to connect to the server automatically when it is opened.
- If the Web Report does not connect to a server, click on the server Status Message to open tools to retry the connection, try a different server, or enter a new server address.
- For information about adding the enhanced controls to your Internet Server, go to https://developer.ibm.com/predictiveanalytics.
- If you specify a new server connection, the preferred format is http://xxx.xxx.xxx.xxx:xxxx.

Editing Tables

|  |  |
| --- | --- |
| Some of this functionality is only available when connected to an Internet server. | |
|  | Create a chart - Create a chart from the selected cells in the table. |
|  | Pivot and Sort - Transpose, sort, and pivot the table. |
|  | Background color - The background color of the selected cells. |
|  | Text Color and Style - Font color, style, and size. |
|  | Number Format - Font color, style, and size. |

Editing Charts

|  |  |
| --- | --- |
| All of this functionality is only available when connected to an Internet server. | |
|  | Chart Size - Change the height and width of the chart |
|  | Background color - The background color of the selected object. |
|  | Border and Line Style - The color and thickness of the line or border. |
|  | Text Color and Style - Font color, style, and size. |
|  | Number Format - Font color, style, and size. |
|  | Axis Properties - Change the scale and display axis titles and ticks. |

Save

X
New Name  
   
  
What to Save   

Save the entire document  
Only save the current object

Server Connection

X
  
Saved Server Connections  
    http://192.168.36.112:9080/webreport/   
  
  
  
Status  
Cannot connect to the specified server.

Add a chart

Pivot and Sort

Chart Size   
  

|  |  |  |
| --- | --- | --- |
|  |  |  |
|  |  |  |
| Lock aspect ratio | | |

Background   

|  |  |  |  |  |  |
| --- | --- | --- | --- | --- | --- |
|  | |  | |  | |
|  |  |  |  |  |  |
|  |  |  |  |  |  |
|  |  |  |  |  |  |

Line and Borders   

|  |  |  |  |  |  |
| --- | --- | --- | --- | --- | --- |
|  | |  | |  | |
|  |  |  |  |  |  |
|  |  |  |  |  |  |
|  |  |  |  |  |  |

  

|  |  |  |
| --- | --- | --- |
|  |  |  |

Text Format   

|  |  |  |  |  |  |
| --- | --- | --- | --- | --- | --- |
|  | |  | |  | |
|  |  |  |  |  |  |
|  |  |  |  |  |  |
|  |  |  |  |  |  |

  

|  |  |  |
| --- | --- | --- |
|  |  |  |

  

|  |  |  |  |
| --- | --- | --- | --- |
|  |  |  | Font Family  Agency FB Aharoni Algerian Arial Arial Black Arial Narrow Arial Rounded MT Bold Bahnschrift Baskerville Old Face Bauhaus 93 Bell MT Berlin Sans FB Berlin Sans FB Demi Bernard MT Condensed Blackadder ITC Bodoni MT Bodoni MT Black Bodoni MT Condensed Bodoni MT Poster Compressed Book Antiqua Bookman Old Style Bookshelf Symbol 7 Bradley Hand ITC Britannic Bold Broadway Brush Script MT Calibri Calibri Light Californian FB Calisto MT Cambria Cambria Math Candara Candara Light Castellar Centaur Century Century Gothic Century Schoolbook Chiller Colonna MT Comic Sans MS Consolas Constantia Cooper Black Copperplate Gothic Bold Copperplate Gothic Light Corbel Corbel Light Courier New Curlz MT David Dialog DialogInput Dubai Dubai Light Dubai Medium Ebrima Edwardian Script ITC Elephant Engravers MT Eras Bold ITC Eras Demi ITC Eras Light ITC Eras Medium ITC Felix Titling Footlight MT Light Forte Franklin Gothic Book Franklin Gothic Demi Franklin Gothic Demi Cond Franklin Gothic Heavy Franklin Gothic Medium Franklin Gothic Medium Cond FrankRuehl Freestyle Script French Script MT Gabriola Gadugi Garamond Georgia Gigi Gill Sans MT Gill Sans MT Condensed Gill Sans MT Ext Condensed Bold Gill Sans Ultra Bold Gill Sans Ultra Bold Condensed Gisha Gloucester MT Extra Condensed Goudy Old Style Goudy Stout Guttman Aharoni Guttman Drogolin Guttman Frank Guttman Frnew Guttman Haim Guttman Haim-Condensed Guttman Hatzvi Guttman Kav Guttman Kav-Light Guttman Logo1 Guttman Mantova Guttman Mantova-Decor Guttman Miryam Guttman Myamfix Guttman Rashi Guttman Stam Guttman Stam1 Guttman Vilna Guttman Yad Guttman Yad-Brush Guttman Yad-Light Guttman-Aharoni Guttman-Aram Guttman-CourMir Hadassah Friedlaender Haettenschweiler Harlow Solid Italic Harrington HelvNeue Roman for IBM High Tower Text HoloLens MDL2 Assets Impact Imprint MT Shadow Informal Roman Ink Free Javanese Text Jokerman Juice ITC Kristen ITC Kunstler Script Leelawadee Leelawadee UI Leelawadee UI Semilight Levenim MT Lucida Bright Lucida Calligraphy Lucida Console Lucida Fax Lucida Handwriting Lucida Sans Lucida Sans Typewriter Lucida Sans Unicode Magneto Maiandra GD Malgun Gothic Malgun Gothic Semilight Marlett Matura MT Script Capitals Microsoft Himalaya Microsoft JhengHei Microsoft JhengHei Light Microsoft JhengHei UI Microsoft JhengHei UI Light Microsoft New Tai Lue Microsoft PhagsPa Microsoft Sans Serif Microsoft Tai Le Microsoft Uighur Microsoft YaHei Microsoft YaHei Light Microsoft YaHei UI Microsoft YaHei UI Light Microsoft Yi Baiti MingLiU-ExtB MingLiU\_HKSCS-ExtB Miriam Miriam Fixed Mistral Modern No. 20 Mongolian Baiti Monospaced Monotype Corsiva MS Gothic MS Outlook MS PGothic MS Reference Sans Serif MS Reference Specialty MS UI Gothic MT Extra MV Boli Myanmar Text Narkisim Niagara Engraved Niagara Solid Nirmala UI Nirmala UI Semilight NSimSun OCR A Extended Old English Text MT Onyx Palace Script MT Palatino Linotype Papyrus Parchment Perpetua Perpetua Titling MT Playbill PMingLiU-ExtB Poor Richard Pristina Rage Italic Ravie Rockwell Rockwell Condensed Rockwell Extra Bold Rod SansSerif Script MT Bold Segoe MDL2 Assets Segoe Print Segoe Script Segoe UI Segoe UI Black Segoe UI Emoji Segoe UI Historic Segoe UI Light Segoe UI Semibold Segoe UI Semilight Segoe UI Symbol Serif Showcard Gothic SimSun SimSun-ExtB Sitka Banner Sitka Display Sitka Heading Sitka Small Sitka Subheading Sitka Text Snap ITC Stencil Sylfaen Symbol Tahoma TeamViewer15 Tempus Sans ITC Times New Roman Trebuchet MS Tw Cen MT Tw Cen MT Condensed Tw Cen MT Condensed Extra Bold Verdana Viner Hand ITC Vivaldi Vladimir Script Webdings Wide Latin Wingdings Wingdings 2 Wingdings 3 Yu Gothic Yu Gothic Light Yu Gothic Medium Yu Gothic UI Yu Gothic UI Light Yu Gothic UI Semibold Yu Gothic UI Semilight |

Number Format   
  

|  |  |  |
| --- | --- | --- |
| 0.00 |  |  |

Axis Options   
  

|  |  |  |
| --- | --- | --- |
|  |  |  |
|  |  |  |
| Display Axis Title | | | |
| Display Ticks | | | |
