# Supplementary material for: Visual acuity improvement in children with albinism beyond the first decade of life
Source: PLoS One. 2024 Jan 17;19(1):e0296744. doi: 10.1371/journal.pone.0296744 (PMC10793880; doi:10.1371/journal.pone.0296744)
Supplement: S3 Data — (HTML) [file pone.0296744.s004.html]

Descriptive Statistics\_new


|  |  |  |
| --- | --- | --- |
| IBM SPSS Web Report - Descriptive Statistics\_new.spv     ---   Contents  Previous  Next  Help |  | Not connected to the server     --- |

- Log

  - Log
- Frequencies

  - Warnings
  - Statistics
  - Frequency Table

    - Srabismus
    - nystagmus
    - Albinism
    - Eye muscle surgery
  - Pie Chart

    - Srabismus for AlbinismType = OCA1A
    - nystagmus for AlbinismType = OCA1A
    - Albinism for AlbinismType = OCA1A
    - Albinism Type for AlbinismType = OCA1A
    - Eye muscle surgery for AlbinismType = OCA1A
    - Srabismus for AlbinismType = OCA1B
    - nystagmus for AlbinismType = OCA1B
    - Albinism for AlbinismType = OCA1B
    - Albinism Type for AlbinismType = OCA1B
    - Eye muscle surgery for AlbinismType = OCA1B
    - Srabismus for AlbinismType = OCA2
    - nystagmus for AlbinismType = OCA2
    - Albinism for AlbinismType = OCA2
    - Albinism Type for AlbinismType = OCA2
    - Eye muscle surgery for AlbinismType = OCA2
- Log

  - Log
- Frequencies

  - Statistics
  - Frequency Table

    - RE\_Myopia
    - RE\_ref error
    - RE\_cyl
    - LE\_Myopia
    - LE\_ref error
    - LE\_cyl
  - Pie Chart

    - RE\_Myopia for AlbinismType = OCA1A
    - RE\_ref error for AlbinismType = OCA1A
    - RE\_cyl for AlbinismType = OCA1A
    - LE\_Myopia for AlbinismType = OCA1A
    - LE\_ref error for AlbinismType = OCA1A
    - LE\_cyl for AlbinismType = OCA1A
    - RE\_Myopia for AlbinismType = OCA1B
    - RE\_ref error for AlbinismType = OCA1B
    - RE\_cyl for AlbinismType = OCA1B
    - LE\_Myopia for AlbinismType = OCA1B
    - LE\_ref error for AlbinismType = OCA1B
    - LE\_cyl for AlbinismType = OCA1B
    - RE\_Myopia for AlbinismType = OCA2
    - RE\_ref error for AlbinismType = OCA2
    - RE\_cyl for AlbinismType = OCA2
    - LE\_Myopia for AlbinismType = OCA2
    - LE\_ref error for AlbinismType = OCA2
    - LE\_cyl for AlbinismType = OCA2
- Log

  - Log
- Frequencies

  - Statistics
- Log

  - Log

- Delete

Log  
Log - Log - May 11, 2022

FREQUENCIES VARIABLES=Srabismus nystagmus Albinism AlbinismType Eyemusclesurgery  
  /PIECHART PERCENT  
  /ORDER=ANALYSIS.

Frequencies  
Frequencies - Warnings - May 11, 2022

WarningsWarnings, table, 0 levels of column headers and 0 levels of row headers, table with 1 columns and 2 rows

|  |
| --- |
| Frequency tables are not produced for the following variables because they are split variables: Albinism Type. |
|  |

Frequencies  
Frequencies - Statistics - May 11, 2022

StatisticsStatistics, table, 1 levels of column headers and 3 levels of row headers, table with 8 columns and 8 rows

|  |  |  |  |  |  |  |  |
| --- | --- | --- | --- | --- | --- | --- | --- |
| Albinism Type | | | Srabismus | nystagmus | Albinism | Albinism Type | Eye muscle surgery |
| OCA1A | N | Valid | 17 | 17 | 17 | 17 | 17 |
|  |  | Missing | 0 | 0 | 0 | 0 | 0 |
| OCA1B | N | Valid | 27 | 28 | 28 | 28 | 28 |
|  |  | Missing | 1 | 0 | 0 | 0 | 0 |
| OCA2 | N | Valid | 26 | 26 | 26 | 26 | 26 |
|  |  | Missing | 0 | 0 | 0 | 0 | 0 |
|  |  |  |  |  |  |  |  |

Frequency Table  
Frequency Table - Srabismus - May 11, 2022

SrabismusSrabismus, table, 1 levels of column headers and 3 levels of row headers, table with 7 columns and 16 rows

|  |  |  |  |  |  |  |
| --- | --- | --- | --- | --- | --- | --- |
| Albinism Type | | | Frequency | Percent | Valid Percent | Cumulative Percent |
| OCA1A | Valid | None | 6 | 35.3 | 35.3 | 35.3 |
|  |  | Iso | 8 | 47.1 | 47.1 | 82.4 |
|  |  | Exo | 3 | 17.6 | 17.6 | 100.0 |
|  |  | Total | 17 | 100.0 | 100.0 |  |
| OCA1B | Valid | None | 8 | 28.6 | 29.6 | 29.6 |
|  |  | Iso | 9 | 32.1 | 33.3 | 63.0 |
|  |  | Exo | 10 | 35.7 | 37.0 | 100.0 |
|  |  | Total | 27 | 96.4 | 100.0 |  |
|  | Missing | System | 1 | 3.6 |  |  |
|  | Total | | 28 | 100.0 |  |  |
| OCA2 | Valid | None | 8 | 30.8 | 30.8 | 30.8 |
|  |  | Iso | 7 | 26.9 | 26.9 | 57.7 |
|  |  | Exo | 11 | 42.3 | 42.3 | 100.0 |
|  |  | Total | 26 | 100.0 | 100.0 |  |
|  |  |  |  |  |  |  |

Frequency Table  
Frequency Table - nystagmus - May 11, 2022

nystagmusnystagmus, table, 1 levels of column headers and 3 levels of row headers, table with 7 columns and 9 rows

|  |  |  |  |  |  |  |
| --- | --- | --- | --- | --- | --- | --- |
| Albinism Type | | | Frequency | Percent | Valid Percent | Cumulative Percent |
| OCA1A | Valid | Yes | 17 | 100.0 | 100.0 | 100.0 |
| OCA1B | Valid | No | 3 | 10.7 | 10.7 | 10.7 |
|  |  | Yes | 25 | 89.3 | 89.3 | 100.0 |
|  |  | Total | 28 | 100.0 | 100.0 |  |
| OCA2 | Valid | No | 2 | 7.7 | 7.7 | 7.7 |
|  |  | Yes | 24 | 92.3 | 92.3 | 100.0 |
|  |  | Total | 26 | 100.0 | 100.0 |  |
|  |  |  |  |  |  |  |

Frequency Table  
Frequency Table - Albinism - May 11, 2022

AlbinismAlbinism, table, 1 levels of column headers and 3 levels of row headers, table with 7 columns and 5 rows

|  |  |  |  |  |  |  |
| --- | --- | --- | --- | --- | --- | --- |
| Albinism Type | | | Frequency | Percent | Valid Percent | Cumulative Percent |
| OCA1A | Valid | Yes | 17 | 100.0 | 100.0 | 100.0 |
| OCA1B | Valid | Yes | 28 | 100.0 | 100.0 | 100.0 |
| OCA2 | Valid | Yes | 26 | 100.0 | 100.0 | 100.0 |
|  |  |  |  |  |  |  |

Frequency Table  
Frequency Table - Eye muscle surgery - May 11, 2022

Eye muscle surgeryEye muscle surgery, table, 1 levels of column headers and 3 levels of row headers, table with 7 columns and 11 rows

|  |  |  |  |  |  |  |
| --- | --- | --- | --- | --- | --- | --- |
| Albinism Type | | | Frequency | Percent | Valid Percent | Cumulative Percent |
| OCA1A | Valid | No | 5 | 29.4 | 29.4 | 29.4 |
|  |  | Yes | 12 | 70.6 | 70.6 | 100.0 |
|  |  | Total | 17 | 100.0 | 100.0 |  |
| OCA1B | Valid | No | 17 | 60.7 | 60.7 | 60.7 |
|  |  | Yes | 11 | 39.3 | 39.3 | 100.0 |
|  |  | Total | 28 | 100.0 | 100.0 |  |
| OCA2 | Valid | No | 14 | 53.8 | 53.8 | 53.8 |
|  |  | Yes | 12 | 46.2 | 46.2 | 100.0 |
|  |  | Total | 26 | 100.0 | 100.0 |  |
|  |  |  |  |  |  |  |

Pie Chart  
Pie Chart - Srabismus for AlbinismType = OCA1A - May 11, 2022

{"copyright":"(C) Copyright IBM Corp. 2011","grammar":[{"elements":[{"color":[{"field":{"$ref":"fVariable"},"palette":["#00B2EF","#D9182D","#FFCF10","#7F1C7D","#007670","#DD731C","#17AF4B","#EE3D96","#838329","#82D1F5","#F04E37","#FFE14F","#AB1A86","#00A6A0","#F19027","#8CC63F","#F389AF","#A5A215","#00649D","#A91024","#FDB813","#3B2056","#006058","#B8471B","#00BA52","#BA006E","#594F13"],"id":"colorAestheticID"}],"data":{"$ref":"dSource"},"style":{"outline":{"r":0,"b":0,"g":0},"stroke":{"cap":"round","width":0.0,"join":"round"}},"position":[{"field":{"$ref":"fVariable1"}}],"type":"interval"}],"coordinates":{"transforms":[{"type":"stack"},{"polarParameters":{"startAngle":90.0},"type":"polar"}],"style":{"outline":{"a":0.0,"r":0,"b":0,"g":0},"fill":{"r":255,"b":255,"g":255}},"dimensions":[{"axis":[]}]}}],"legends":[{"labelStyle":{"fill":{"r":0,"b":157,"g":100},"font":{"size":"10pt","weight":"normal","family":"sans-serif"}},"itemStyle":{"padding":3,"size":11},"location":{"width":"24%"},"aesthetics":[{"aesthetic":{"$ref":"colorAestheticID"}}],"boundsStyle":{"padding":5,"outline":{"a":0.0,"r":0,"b":0,"g":0},"fill":{"a":0.0,"r":0,"b":0,"g":0}}}],"data":[{"id":"dSource","fields":[{"format":{"numericPattern":"###"},"id":"fVariable","label":"V4","categories":["None","Iso","Exo"]},{"min":17.64705882352941,"max":47.05882352941176,"id":"fVariable1","label":"Y Axis"}],"rows":[[0,35.29411764705883],[1,47.05882352941176],[2,17.64705882352941]]}],"size":{"width":850.0,"height":500.0},"style":{"outline":{"a":0.0,"r":0,"b":0,"g":0},"fill":{"r":255,"b":255,"g":255}},"titles":[{"backgroundStyle":{"outline":{"a":0.0,"r":0,"b":0,"g":0},"fill":{"a":0.0,"r":0,"b":0,"g":0}},"style":{"padding":3.0,"fill":{"r":0,"b":157,"g":100},"font":{"size":"12pt","weight":"bold","family":"sans-serif"}},"type":"title","content":["Srabismus"]},{"backgroundStyle":{"outline":{"a":0.0,"r":0,"b":0,"g":0},"fill":{"a":0.0,"r":0,"b":0,"g":0}},"style":{"padding":3.0,"fill":{"r":0,"b":157,"g":100},"font":{"size":"10pt","weight":"bold","family":"sans-serif"}},"type":"title","content":["Albinism Type: OCA1A"]}],"version":"6.0"}

Pie Chart  
Pie Chart - nystagmus for AlbinismType = OCA1A - May 11, 2022

{"copyright":"(C) Copyright IBM Corp. 2011","grammar":[{"elements":[{"color":[{"field":{"$ref":"fVariable"},"palette":["#00B2EF","#D9182D","#FFCF10","#7F1C7D","#007670","#DD731C","#17AF4B","#EE3D96","#838329","#82D1F5","#F04E37","#FFE14F","#AB1A86","#00A6A0","#F19027","#8CC63F","#F389AF","#A5A215","#00649D","#A91024","#FDB813","#3B2056","#006058","#B8471B","#00BA52","#BA006E","#594F13"],"id":"colorAestheticID"}],"data":{"$ref":"dSource"},"style":{"outline":{"r":0,"b":0,"g":0},"stroke":{"cap":"round","width":0.0,"join":"round"}},"position":[{"field":{"$ref":"fVariable1"}}],"type":"interval"}],"coordinates":{"transforms":[{"type":"stack"},{"polarParameters":{"startAngle":90.0},"type":"polar"}],"style":{"outline":{"a":0.0,"r":0,"b":0,"g":0},"fill":{"r":255,"b":255,"g":255}},"dimensions":[{"axis":[]}]}}],"legends":[{"labelStyle":{"fill":{"r":0,"b":157,"g":100},"font":{"size":"10pt","weight":"normal","family":"sans-serif"}},"itemStyle":{"padding":3,"size":11},"location":{"width":"24%"},"aesthetics":[{"aesthetic":{"$ref":"colorAestheticID"}}],"boundsStyle":{"padding":5,"outline":{"a":0.0,"r":0,"b":0,"g":0},"fill":{"a":0.0,"r":0,"b":0,"g":0}}}],"data":[{"id":"dSource","fields":[{"format":{"numericPattern":"###"},"id":"fVariable","label":"V4","categories":["Yes"]},{"min":100.0,"max":100.0,"id":"fVariable1","label":"Y Axis"}],"rows":[[0,100]]}],"size":{"width":850.0,"height":500.0},"style":{"outline":{"a":0.0,"r":0,"b":0,"g":0},"fill":{"r":255,"b":255,"g":255}},"titles":[{"backgroundStyle":{"outline":{"a":0.0,"r":0,"b":0,"g":0},"fill":{"a":0.0,"r":0,"b":0,"g":0}},"style":{"padding":3.0,"fill":{"r":0,"b":157,"g":100},"font":{"size":"12pt","weight":"bold","family":"sans-serif"}},"type":"title","content":["nystagmus"]},{"backgroundStyle":{"outline":{"a":0.0,"r":0,"b":0,"g":0},"fill":{"a":0.0,"r":0,"b":0,"g":0}},"style":{"padding":3.0,"fill":{"r":0,"b":157,"g":100},"font":{"size":"10pt","weight":"bold","family":"sans-serif"}},"type":"title","content":["Albinism Type: OCA1A"]}],"version":"6.0"}

Pie Chart  
Pie Chart - Albinism for AlbinismType = OCA1A - May 11, 2022

{"copyright":"(C) Copyright IBM Corp. 2011","grammar":[{"elements":[{"color":[{"field":{"$ref":"fVariable"},"palette":["#00B2EF","#D9182D","#FFCF10","#7F1C7D","#007670","#DD731C","#17AF4B","#EE3D96","#838329","#82D1F5","#F04E37","#FFE14F","#AB1A86","#00A6A0","#F19027","#8CC63F","#F389AF","#A5A215","#00649D","#A91024","#FDB813","#3B2056","#006058","#B8471B","#00BA52","#BA006E","#594F13"],"id":"colorAestheticID"}],"data":{"$ref":"dSource"},"style":{"outline":{"r":0,"b":0,"g":0},"stroke":{"cap":"round","width":0.0,"join":"round"}},"position":[{"field":{"$ref":"fVariable1"}}],"type":"interval"}],"coordinates":{"transforms":[{"type":"stack"},{"polarParameters":{"startAngle":90.0},"type":"polar"}],"style":{"outline":{"a":0.0,"r":0,"b":0,"g":0},"fill":{"r":255,"b":255,"g":255}},"dimensions":[{"axis":[]}]}}],"legends":[{"labelStyle":{"fill":{"r":0,"b":157,"g":100},"font":{"size":"10pt","weight":"normal","family":"sans-serif"}},"itemStyle":{"padding":3,"size":11},"location":{"width":"24%"},"aesthetics":[{"aesthetic":{"$ref":"colorAestheticID"}}],"boundsStyle":{"padding":5,"outline":{"a":0.0,"r":0,"b":0,"g":0},"fill":{"a":0.0,"r":0,"b":0,"g":0}}}],"data":[{"id":"dSource","fields":[{"format":{"numericPattern":"###"},"id":"fVariable","label":"V4","categories":["Yes"]},{"min":100.0,"max":100.0,"id":"fVariable1","label":"Y Axis"}],"rows":[[0,100]]}],"size":{"width":850.0,"height":500.0},"style":{"outline":{"a":0.0,"r":0,"b":0,"g":0},"fill":{"r":255,"b":255,"g":255}},"titles":[{"backgroundStyle":{"outline":{"a":0.0,"r":0,"b":0,"g":0},"fill":{"a":0.0,"r":0,"b":0,"g":0}},"style":{"padding":3.0,"fill":{"r":0,"b":157,"g":100},"font":{"size":"12pt","weight":"bold","family":"sans-serif"}},"type":"title","content":["Albinism"]},{"backgroundStyle":{"outline":{"a":0.0,"r":0,"b":0,"g":0},"fill":{"a":0.0,"r":0,"b":0,"g":0}},"style":{"padding":3.0,"fill":{"r":0,"b":157,"g":100},"font":{"size":"10pt","weight":"bold","family":"sans-serif"}},"type":"title","content":["Albinism Type: OCA1A"]}],"version":"6.0"}

Pie Chart  
Pie Chart - Albinism Type for AlbinismType = OCA1A - May 11, 2022

{"copyright":"(C) Copyright IBM Corp. 2011","grammar":[{"elements":[{"color":[{"field":{"$ref":"fVariable"},"palette":["#00B2EF","#D9182D","#FFCF10","#7F1C7D","#007670","#DD731C","#17AF4B","#EE3D96","#838329","#82D1F5","#F04E37","#FFE14F","#AB1A86","#00A6A0","#F19027","#8CC63F","#F389AF","#A5A215","#00649D","#A91024","#FDB813","#3B2056","#006058","#B8471B","#00BA52","#BA006E","#594F13"],"id":"colorAestheticID"}],"data":{"$ref":"dSource"},"style":{"outline":{"r":0,"b":0,"g":0},"stroke":{"cap":"round","width":0.0,"join":"round"}},"position":[{"field":{"$ref":"fVariable1"}}],"type":"interval"}],"coordinates":{"transforms":[{"type":"stack"},{"polarParameters":{"startAngle":90.0},"type":"polar"}],"style":{"outline":{"a":0.0,"r":0,"b":0,"g":0},"fill":{"r":255,"b":255,"g":255}},"dimensions":[{"axis":[]}]}}],"legends":[{"labelStyle":{"fill":{"r":0,"b":157,"g":100},"font":{"size":"10pt","weight":"normal","family":"sans-serif"}},"itemStyle":{"padding":3,"size":11},"location":{"width":"24%"},"aesthetics":[{"aesthetic":{"$ref":"colorAestheticID"}}],"boundsStyle":{"padding":5,"outline":{"a":0.0,"r":0,"b":0,"g":0},"fill":{"a":0.0,"r":0,"b":0,"g":0}}}],"data":[{"id":"dSource","fields":[{"format":{"numericPattern":"###"},"id":"fVariable","label":"Albinism Type","categories":["OCA1A"]},{"min":100.0,"max":100.0,"id":"fVariable1","label":"Y Axis"}],"rows":[[0,100]]}],"size":{"width":850.0,"height":500.0},"style":{"outline":{"a":0.0,"r":0,"b":0,"g":0},"fill":{"r":255,"b":255,"g":255}},"titles":[{"backgroundStyle":{"outline":{"a":0.0,"r":0,"b":0,"g":0},"fill":{"a":0.0,"r":0,"b":0,"g":0}},"style":{"padding":3.0,"fill":{"r":0,"b":157,"g":100},"font":{"size":"12pt","weight":"bold","family":"sans-serif"}},"type":"title","content":["Albinism Type"]},{"backgroundStyle":{"outline":{"a":0.0,"r":0,"b":0,"g":0},"fill":{"a":0.0,"r":0,"b":0,"g":0}},"style":{"padding":3.0,"fill":{"r":0,"b":157,"g":100},"font":{"size":"10pt","weight":"bold","family":"sans-serif"}},"type":"title","content":["Albinism Type: OCA1A"]}],"version":"6.0"}

Pie Chart  
Pie Chart - Eye muscle surgery for AlbinismType = OCA1A - May 11, 2022

{"copyright":"(C) Copyright IBM Corp. 2011","grammar":[{"elements":[{"color":[{"field":{"$ref":"fVariable"},"palette":["#00B2EF","#D9182D","#FFCF10","#7F1C7D","#007670","#DD731C","#17AF4B","#EE3D96","#838329","#82D1F5","#F04E37","#FFE14F","#AB1A86","#00A6A0","#F19027","#8CC63F","#F389AF","#A5A215","#00649D","#A91024","#FDB813","#3B2056","#006058","#B8471B","#00BA52","#BA006E","#594F13"],"id":"colorAestheticID"}],"data":{"$ref":"dSource"},"style":{"outline":{"r":0,"b":0,"g":0},"stroke":{"cap":"round","width":0.0,"join":"round"}},"position":[{"field":{"$ref":"fVariable1"}}],"type":"interval"}],"coordinates":{"transforms":[{"type":"stack"},{"polarParameters":{"startAngle":90.0},"type":"polar"}],"style":{"outline":{"a":0.0,"r":0,"b":0,"g":0},"fill":{"r":255,"b":255,"g":255}},"dimensions":[{"axis":[]}]}}],"legends":[{"labelStyle":{"fill":{"r":0,"b":157,"g":100},"font":{"size":"10pt","weight":"normal","family":"sans-serif"}},"itemStyle":{"padding":3,"size":11},"location":{"width":"24%"},"aesthetics":[{"aesthetic":{"$ref":"colorAestheticID"}}],"boundsStyle":{"padding":5,"outline":{"a":0.0,"r":0,"b":0,"g":0},"fill":{"a":0.0,"r":0,"b":0,"g":0}}}],"data":[{"id":"dSource","fields":[{"format":{"numericPattern":"###"},"id":"fVariable","label":"Eye muscle surgery","categories":["No","Yes"]},{"min":29.41176470588236,"max":70.58823529411765,"id":"fVariable1","label":"Y Axis"}],"rows":[[0,29.41176470588236],[1,70.58823529411765]]}],"size":{"width":850.0,"height":500.0},"style":{"outline":{"a":0.0,"r":0,"b":0,"g":0},"fill":{"r":255,"b":255,"g":255}},"titles":[{"backgroundStyle":{"outline":{"a":0.0,"r":0,"b":0,"g":0},"fill":{"a":0.0,"r":0,"b":0,"g":0}},"style":{"padding":3.0,"fill":{"r":0,"b":157,"g":100},"font":{"size":"12pt","weight":"bold","family":"sans-serif"}},"type":"title","content":["Eye muscle surgery"]},{"backgroundStyle":{"outline":{"a":0.0,"r":0,"b":0,"g":0},"fill":{"a":0.0,"r":0,"b":0,"g":0}},"style":{"padding":3.0,"fill":{"r":0,"b":157,"g":100},"font":{"size":"10pt","weight":"bold","family":"sans-serif"}},"type":"title","content":["Albinism Type: OCA1A"]}],"version":"6.0"}

Pie Chart  
Pie Chart - Srabismus for AlbinismType = OCA1B - May 11, 2022

{"copyright":"(C) Copyright IBM Corp. 2011","grammar":[{"elements":[{"color":[{"field":{"$ref":"fVariable"},"palette":["#00B2EF","#D9182D","#FFCF10","#7F1C7D","#007670","#DD731C","#17AF4B","#EE3D96","#838329","#82D1F5","#F04E37","#FFE14F","#AB1A86","#00A6A0","#F19027","#8CC63F","#F389AF","#A5A215","#00649D","#A91024","#FDB813","#3B2056","#006058","#B8471B","#00BA52","#BA006E","#594F13"],"id":"colorAestheticID"}],"data":{"$ref":"dSource"},"style":{"outline":{"r":0,"b":0,"g":0},"stroke":{"cap":"round","width":0.0,"join":"round"}},"position":[{"field":{"$ref":"fVariable1"}}],"type":"interval"}],"coordinates":{"transforms":[{"type":"stack"},{"polarParameters":{"startAngle":90.0},"type":"polar"}],"style":{"outline":{"a":0.0,"r":0,"b":0,"g":0},"fill":{"r":255,"b":255,"g":255}},"dimensions":[{"axis":[]}]}}],"legends":[{"labelStyle":{"fill":{"r":0,"b":157,"g":100},"font":{"size":"10pt","weight":"normal","family":"sans-serif"}},"itemStyle":{"padding":3,"size":11},"location":{"width":"24%"},"aesthetics":[{"aesthetic":{"$ref":"colorAestheticID"}}],"boundsStyle":{"padding":5,"outline":{"a":0.0,"r":0,"b":0,"g":0},"fill":{"a":0.0,"r":0,"b":0,"g":0}}}],"data":[{"id":"dSource","fields":[{"format":{"numericPattern":"###"},"id":"fVariable","label":"V4","categories":["None","Iso","Exo"]},{"min":29.62962962962963,"max":37.03703703703704,"id":"fVariable1","label":"Y Axis"}],"rows":[[0,29.62962962962963],[1,33.33333333333333],[2,37.03703703703704]]}],"size":{"width":850.0,"height":500.0},"style":{"outline":{"a":0.0,"r":0,"b":0,"g":0},"fill":{"r":255,"b":255,"g":255}},"titles":[{"backgroundStyle":{"outline":{"a":0.0,"r":0,"b":0,"g":0},"fill":{"a":0.0,"r":0,"b":0,"g":0}},"style":{"padding":3.0,"fill":{"r":0,"b":157,"g":100},"font":{"size":"12pt","weight":"bold","family":"sans-serif"}},"type":"title","content":["Srabismus"]},{"backgroundStyle":{"outline":{"a":0.0,"r":0,"b":0,"g":0},"fill":{"a":0.0,"r":0,"b":0,"g":0}},"style":{"padding":3.0,"fill":{"r":0,"b":157,"g":100},"font":{"size":"10pt","weight":"bold","family":"sans-serif"}},"type":"title","content":["Albinism Type: OCA1B"]}],"version":"6.0"}

Pie Chart  
Pie Chart - nystagmus for AlbinismType = OCA1B - May 11, 2022

{"copyright":"(C) Copyright IBM Corp. 2011","grammar":[{"elements":[{"color":[{"field":{"$ref":"fVariable"},"palette":["#00B2EF","#D9182D","#FFCF10","#7F1C7D","#007670","#DD731C","#17AF4B","#EE3D96","#838329","#82D1F5","#F04E37","#FFE14F","#AB1A86","#00A6A0","#F19027","#8CC63F","#F389AF","#A5A215","#00649D","#A91024","#FDB813","#3B2056","#006058","#B8471B","#00BA52","#BA006E","#594F13"],"id":"colorAestheticID"}],"data":{"$ref":"dSource"},"style":{"outline":{"r":0,"b":0,"g":0},"stroke":{"cap":"round","width":0.0,"join":"round"}},"position":[{"field":{"$ref":"fVariable1"}}],"type":"interval"}],"coordinates":{"transforms":[{"type":"stack"},{"polarParameters":{"startAngle":90.0},"type":"polar"}],"style":{"outline":{"a":0.0,"r":0,"b":0,"g":0},"fill":{"r":255,"b":255,"g":255}},"dimensions":[{"axis":[]}]}}],"legends":[{"labelStyle":{"fill":{"r":0,"b":157,"g":100},"font":{"size":"10pt","weight":"normal","family":"sans-serif"}},"itemStyle":{"padding":3,"size":11},"location":{"width":"24%"},"aesthetics":[{"aesthetic":{"$ref":"colorAestheticID"}}],"boundsStyle":{"padding":5,"outline":{"a":0.0,"r":0,"b":0,"g":0},"fill":{"a":0.0,"r":0,"b":0,"g":0}}}],"data":[{"id":"dSource","fields":[{"format":{"numericPattern":"###"},"id":"fVariable","label":"V4","categories":["No","Yes"]},{"min":10.71428571428571,"max":89.28571428571429,"id":"fVariable1","label":"Y Axis"}],"rows":[[0,10.71428571428571],[1,89.28571428571429]]}],"size":{"width":850.0,"height":500.0},"style":{"outline":{"a":0.0,"r":0,"b":0,"g":0},"fill":{"r":255,"b":255,"g":255}},"titles":[{"backgroundStyle":{"outline":{"a":0.0,"r":0,"b":0,"g":0},"fill":{"a":0.0,"r":0,"b":0,"g":0}},"style":{"padding":3.0,"fill":{"r":0,"b":157,"g":100},"font":{"size":"12pt","weight":"bold","family":"sans-serif"}},"type":"title","content":["nystagmus"]},{"backgroundStyle":{"outline":{"a":0.0,"r":0,"b":0,"g":0},"fill":{"a":0.0,"r":0,"b":0,"g":0}},"style":{"padding":3.0,"fill":{"r":0,"b":157,"g":100},"font":{"size":"10pt","weight":"bold","family":"sans-serif"}},"type":"title","content":["Albinism Type: OCA1B"]}],"version":"6.0"}

Pie Chart  
Pie Chart - Albinism for AlbinismType = OCA1B - May 11, 2022

{"copyright":"(C) Copyright IBM Corp. 2011","grammar":[{"elements":[{"color":[{"field":{"$ref":"fVariable"},"palette":["#00B2EF","#D9182D","#FFCF10","#7F1C7D","#007670","#DD731C","#17AF4B","#EE3D96","#838329","#82D1F5","#F04E37","#FFE14F","#AB1A86","#00A6A0","#F19027","#8CC63F","#F389AF","#A5A215","#00649D","#A91024","#FDB813","#3B2056","#006058","#B8471B","#00BA52","#BA006E","#594F13"],"id":"colorAestheticID"}],"data":{"$ref":"dSource"},"style":{"outline":{"r":0,"b":0,"g":0},"stroke":{"cap":"round","width":0.0,"join":"round"}},"position":[{"field":{"$ref":"fVariable1"}}],"type":"interval"}],"coordinates":{"transforms":[{"type":"stack"},{"polarParameters":{"startAngle":90.0},"type":"polar"}],"style":{"outline":{"a":0.0,"r":0,"b":0,"g":0},"fill":{"r":255,"b":255,"g":255}},"dimensions":[{"axis":[]}]}}],"legends":[{"labelStyle":{"fill":{"r":0,"b":157,"g":100},"font":{"size":"10pt","weight":"normal","family":"sans-serif"}},"itemStyle":{"padding":3,"size":11},"location":{"width":"24%"},"aesthetics":[{"aesthetic":{"$ref":"colorAestheticID"}}],"boundsStyle":{"padding":5,"outline":{"a":0.0,"r":0,"b":0,"g":0},"fill":{"a":0.0,"r":0,"b":0,"g":0}}}],"data":[{"id":"dSource","fields":[{"format":{"numericPattern":"###"},"id":"fVariable","label":"V4","categories":["Yes"]},{"min":100.0,"max":100.0,"id":"fVariable1","label":"Y Axis"}],"rows":[[0,100]]}],"size":{"width":850.0,"height":500.0},"style":{"outline":{"a":0.0,"r":0,"b":0,"g":0},"fill":{"r":255,"b":255,"g":255}},"titles":[{"backgroundStyle":{"outline":{"a":0.0,"r":0,"b":0,"g":0},"fill":{"a":0.0,"r":0,"b":0,"g":0}},"style":{"padding":3.0,"fill":{"r":0,"b":157,"g":100},"font":{"size":"12pt","weight":"bold","family":"sans-serif"}},"type":"title","content":["Albinism"]},{"backgroundStyle":{"outline":{"a":0.0,"r":0,"b":0,"g":0},"fill":{"a":0.0,"r":0,"b":0,"g":0}},"style":{"padding":3.0,"fill":{"r":0,"b":157,"g":100},"font":{"size":"10pt","weight":"bold","family":"sans-serif"}},"type":"title","content":["Albinism Type: OCA1B"]}],"version":"6.0"}

Pie Chart  
Pie Chart - Albinism Type for AlbinismType = OCA1B - May 11, 2022

{"copyright":"(C) Copyright IBM Corp. 2011","grammar":[{"elements":[{"color":[{"field":{"$ref":"fVariable"},"palette":["#00B2EF","#D9182D","#FFCF10","#7F1C7D","#007670","#DD731C","#17AF4B","#EE3D96","#838329","#82D1F5","#F04E37","#FFE14F","#AB1A86","#00A6A0","#F19027","#8CC63F","#F389AF","#A5A215","#00649D","#A91024","#FDB813","#3B2056","#006058","#B8471B","#00BA52","#BA006E","#594F13"],"id":"colorAestheticID"}],"data":{"$ref":"dSource"},"style":{"outline":{"r":0,"b":0,"g":0},"stroke":{"cap":"round","width":0.0,"join":"round"}},"position":[{"field":{"$ref":"fVariable1"}}],"type":"interval"}],"coordinates":{"transforms":[{"type":"stack"},{"polarParameters":{"startAngle":90.0},"type":"polar"}],"style":{"outline":{"a":0.0,"r":0,"b":0,"g":0},"fill":{"r":255,"b":255,"g":255}},"dimensions":[{"axis":[]}]}}],"legends":[{"labelStyle":{"fill":{"r":0,"b":157,"g":100},"font":{"size":"10pt","weight":"normal","family":"sans-serif"}},"itemStyle":{"padding":3,"size":11},"location":{"width":"24%"},"aesthetics":[{"aesthetic":{"$ref":"colorAestheticID"}}],"boundsStyle":{"padding":5,"outline":{"a":0.0,"r":0,"b":0,"g":0},"fill":{"a":0.0,"r":0,"b":0,"g":0}}}],"data":[{"id":"dSource","fields":[{"format":{"numericPattern":"###"},"id":"fVariable","label":"Albinism Type","categories":["OCA1B"]},{"min":100.0,"max":100.0,"id":"fVariable1","label":"Y Axis"}],"rows":[[0,100]]}],"size":{"width":850.0,"height":500.0},"style":{"outline":{"a":0.0,"r":0,"b":0,"g":0},"fill":{"r":255,"b":255,"g":255}},"titles":[{"backgroundStyle":{"outline":{"a":0.0,"r":0,"b":0,"g":0},"fill":{"a":0.0,"r":0,"b":0,"g":0}},"style":{"padding":3.0,"fill":{"r":0,"b":157,"g":100},"font":{"size":"12pt","weight":"bold","family":"sans-serif"}},"type":"title","content":["Albinism Type"]},{"backgroundStyle":{"outline":{"a":0.0,"r":0,"b":0,"g":0},"fill":{"a":0.0,"r":0,"b":0,"g":0}},"style":{"padding":3.0,"fill":{"r":0,"b":157,"g":100},"font":{"size":"10pt","weight":"bold","family":"sans-serif"}},"type":"title","content":["Albinism Type: OCA1B"]}],"version":"6.0"}

Pie Chart  
Pie Chart - Eye muscle surgery for AlbinismType = OCA1B - May 11, 2022

{"copyright":"(C) Copyright IBM Corp. 2011","grammar":[{"elements":[{"color":[{"field":{"$ref":"fVariable"},"palette":["#00B2EF","#D9182D","#FFCF10","#7F1C7D","#007670","#DD731C","#17AF4B","#EE3D96","#838329","#82D1F5","#F04E37","#FFE14F","#AB1A86","#00A6A0","#F19027","#8CC63F","#F389AF","#A5A215","#00649D","#A91024","#FDB813","#3B2056","#006058","#B8471B","#00BA52","#BA006E","#594F13"],"id":"colorAestheticID"}],"data":{"$ref":"dSource"},"style":{"outline":{"r":0,"b":0,"g":0},"stroke":{"cap":"round","width":0.0,"join":"round"}},"position":[{"field":{"$ref":"fVariable1"}}],"type":"interval"}],"coordinates":{"transforms":[{"type":"stack"},{"polarParameters":{"startAngle":90.0},"type":"polar"}],"style":{"outline":{"a":0.0,"r":0,"b":0,"g":0},"fill":{"r":255,"b":255,"g":255}},"dimensions":[{"axis":[]}]}}],"legends":[{"labelStyle":{"fill":{"r":0,"b":157,"g":100},"font":{"size":"10pt","weight":"normal","family":"sans-serif"}},"itemStyle":{"padding":3,"size":11},"location":{"width":"24%"},"aesthetics":[{"aesthetic":{"$ref":"colorAestheticID"}}],"boundsStyle":{"padding":5,"outline":{"a":0.0,"r":0,"b":0,"g":0},"fill":{"a":0.0,"r":0,"b":0,"g":0}}}],"data":[{"id":"dSource","fields":[{"format":{"numericPattern":"###"},"id":"fVariable","label":"Eye muscle surgery","categories":["No","Yes"]},{"min":39.28571428571429,"max":60.71428571428571,"id":"fVariable1","label":"Y Axis"}],"rows":[[0,60.71428571428571],[1,39.28571428571429]]}],"size":{"width":850.0,"height":500.0},"style":{"outline":{"a":0.0,"r":0,"b":0,"g":0},"fill":{"r":255,"b":255,"g":255}},"titles":[{"backgroundStyle":{"outline":{"a":0.0,"r":0,"b":0,"g":0},"fill":{"a":0.0,"r":0,"b":0,"g":0}},"style":{"padding":3.0,"fill":{"r":0,"b":157,"g":100},"font":{"size":"12pt","weight":"bold","family":"sans-serif"}},"type":"title","content":["Eye muscle surgery"]},{"backgroundStyle":{"outline":{"a":0.0,"r":0,"b":0,"g":0},"fill":{"a":0.0,"r":0,"b":0,"g":0}},"style":{"padding":3.0,"fill":{"r":0,"b":157,"g":100},"font":{"size":"10pt","weight":"bold","family":"sans-serif"}},"type":"title","content":["Albinism Type: OCA1B"]}],"version":"6.0"}

Pie Chart  
Pie Chart - Srabismus for AlbinismType = OCA2 - May 11, 2022

{"copyright":"(C) Copyright IBM Corp. 2011","grammar":[{"elements":[{"color":[{"field":{"$ref":"fVariable"},"palette":["#00B2EF","#D9182D","#FFCF10","#7F1C7D","#007670","#DD731C","#17AF4B","#EE3D96","#838329","#82D1F5","#F04E37","#FFE14F","#AB1A86","#00A6A0","#F19027","#8CC63F","#F389AF","#A5A215","#00649D","#A91024","#FDB813","#3B2056","#006058","#B8471B","#00BA52","#BA006E","#594F13"],"id":"colorAestheticID"}],"data":{"$ref":"dSource"},"style":{"outline":{"r":0,"b":0,"g":0},"stroke":{"cap":"round","width":0.0,"join":"round"}},"position":[{"field":{"$ref":"fVariable1"}}],"type":"interval"}],"coordinates":{"transforms":[{"type":"stack"},{"polarParameters":{"startAngle":90.0},"type":"polar"}],"style":{"outline":{"a":0.0,"r":0,"b":0,"g":0},"fill":{"r":255,"b":255,"g":255}},"dimensions":[{"axis":[]}]}}],"legends":[{"labelStyle":{"fill":{"r":0,"b":157,"g":100},"font":{"size":"10pt","weight":"normal","family":"sans-serif"}},"itemStyle":{"padding":3,"size":11},"location":{"width":"24%"},"aesthetics":[{"aesthetic":{"$ref":"colorAestheticID"}}],"boundsStyle":{"padding":5,"outline":{"a":0.0,"r":0,"b":0,"g":0},"fill":{"a":0.0,"r":0,"b":0,"g":0}}}],"data":[{"id":"dSource","fields":[{"format":{"numericPattern":"###"},"id":"fVariable","label":"V4","categories":["None","Iso","Exo"]},{"min":26.92307692307692,"max":42.30769230769231,"id":"fVariable1","label":"Y Axis"}],"rows":[[0,30.76923076923077],[1,26.92307692307692],[2,42.30769230769231]]}],"size":{"width":850.0,"height":500.0},"style":{"outline":{"a":0.0,"r":0,"b":0,"g":0},"fill":{"r":255,"b":255,"g":255}},"titles":[{"backgroundStyle":{"outline":{"a":0.0,"r":0,"b":0,"g":0},"fill":{"a":0.0,"r":0,"b":0,"g":0}},"style":{"padding":3.0,"fill":{"r":0,"b":157,"g":100},"font":{"size":"12pt","weight":"bold","family":"sans-serif"}},"type":"title","content":["Srabismus"]},{"backgroundStyle":{"outline":{"a":0.0,"r":0,"b":0,"g":0},"fill":{"a":0.0,"r":0,"b":0,"g":0}},"style":{"padding":3.0,"fill":{"r":0,"b":157,"g":100},"font":{"size":"10pt","weight":"bold","family":"sans-serif"}},"type":"title","content":["Albinism Type: OCA2"]}],"version":"6.0"}

Pie Chart  
Pie Chart - nystagmus for AlbinismType = OCA2 - May 11, 2022

{"copyright":"(C) Copyright IBM Corp. 2011","grammar":[{"elements":[{"color":[{"field":{"$ref":"fVariable"},"palette":["#00B2EF","#D9182D","#FFCF10","#7F1C7D","#007670","#DD731C","#17AF4B","#EE3D96","#838329","#82D1F5","#F04E37","#FFE14F","#AB1A86","#00A6A0","#F19027","#8CC63F","#F389AF","#A5A215","#00649D","#A91024","#FDB813","#3B2056","#006058","#B8471B","#00BA52","#BA006E","#594F13"],"id":"colorAestheticID"}],"data":{"$ref":"dSource"},"style":{"outline":{"r":0,"b":0,"g":0},"stroke":{"cap":"round","width":0.0,"join":"round"}},"position":[{"field":{"$ref":"fVariable1"}}],"type":"interval"}],"coordinates":{"transforms":[{"type":"stack"},{"polarParameters":{"startAngle":90.0},"type":"polar"}],"style":{"outline":{"a":0.0,"r":0,"b":0,"g":0},"fill":{"r":255,"b":255,"g":255}},"dimensions":[{"axis":[]}]}}],"legends":[{"labelStyle":{"fill":{"r":0,"b":157,"g":100},"font":{"size":"10pt","weight":"normal","family":"sans-serif"}},"itemStyle":{"padding":3,"size":11},"location":{"width":"24%"},"aesthetics":[{"aesthetic":{"$ref":"colorAestheticID"}}],"boundsStyle":{"padding":5,"outline":{"a":0.0,"r":0,"b":0,"g":0},"fill":{"a":0.0,"r":0,"b":0,"g":0}}}],"data":[{"id":"dSource","fields":[{"format":{"numericPattern":"###"},"id":"fVariable","label":"V4","categories":["No","Yes"]},{"min":7.692307692307693,"max":92.3076923076923,"id":"fVariable1","label":"Y Axis"}],"rows":[[0,7.692307692307693],[1,92.3076923076923]]}],"size":{"width":850.0,"height":500.0},"style":{"outline":{"a":0.0,"r":0,"b":0,"g":0},"fill":{"r":255,"b":255,"g":255}},"titles":[{"backgroundStyle":{"outline":{"a":0.0,"r":0,"b":0,"g":0},"fill":{"a":0.0,"r":0,"b":0,"g":0}},"style":{"padding":3.0,"fill":{"r":0,"b":157,"g":100},"font":{"size":"12pt","weight":"bold","family":"sans-serif"}},"type":"title","content":["nystagmus"]},{"backgroundStyle":{"outline":{"a":0.0,"r":0,"b":0,"g":0},"fill":{"a":0.0,"r":0,"b":0,"g":0}},"style":{"padding":3.0,"fill":{"r":0,"b":157,"g":100},"font":{"size":"10pt","weight":"bold","family":"sans-serif"}},"type":"title","content":["Albinism Type: OCA2"]}],"version":"6.0"}

Pie Chart  
Pie Chart - Albinism for AlbinismType = OCA2 - May 11, 2022

{"copyright":"(C) Copyright IBM Corp. 2011","grammar":[{"elements":[{"color":[{"field":{"$ref":"fVariable"},"palette":["#00B2EF","#D9182D","#FFCF10","#7F1C7D","#007670","#DD731C","#17AF4B","#EE3D96","#838329","#82D1F5","#F04E37","#FFE14F","#AB1A86","#00A6A0","#F19027","#8CC63F","#F389AF","#A5A215","#00649D","#A91024","#FDB813","#3B2056","#006058","#B8471B","#00BA52","#BA006E","#594F13"],"id":"colorAestheticID"}],"data":{"$ref":"dSource"},"style":{"outline":{"r":0,"b":0,"g":0},"stroke":{"cap":"round","width":0.0,"join":"round"}},"position":[{"field":{"$ref":"fVariable1"}}],"type":"interval"}],"coordinates":{"transforms":[{"type":"stack"},{"polarParameters":{"startAngle":90.0},"type":"polar"}],"style":{"outline":{"a":0.0,"r":0,"b":0,"g":0},"fill":{"r":255,"b":255,"g":255}},"dimensions":[{"axis":[]}]}}],"legends":[{"labelStyle":{"fill":{"r":0,"b":157,"g":100},"font":{"size":"10pt","weight":"normal","family":"sans-serif"}},"itemStyle":{"padding":3,"size":11},"location":{"width":"24%"},"aesthetics":[{"aesthetic":{"$ref":"colorAestheticID"}}],"boundsStyle":{"padding":5,"outline":{"a":0.0,"r":0,"b":0,"g":0},"fill":{"a":0.0,"r":0,"b":0,"g":0}}}],"data":[{"id":"dSource","fields":[{"format":{"numericPattern":"###"},"id":"fVariable","label":"V4","categories":["Yes"]},{"min":100.0,"max":100.0,"id":"fVariable1","label":"Y Axis"}],"rows":[[0,100]]}],"size":{"width":850.0,"height":500.0},"style":{"outline":{"a":0.0,"r":0,"b":0,"g":0},"fill":{"r":255,"b":255,"g":255}},"titles":[{"backgroundStyle":{"outline":{"a":0.0,"r":0,"b":0,"g":0},"fill":{"a":0.0,"r":0,"b":0,"g":0}},"style":{"padding":3.0,"fill":{"r":0,"b":157,"g":100},"font":{"size":"12pt","weight":"bold","family":"sans-serif"}},"type":"title","content":["Albinism"]},{"backgroundStyle":{"outline":{"a":0.0,"r":0,"b":0,"g":0},"fill":{"a":0.0,"r":0,"b":0,"g":0}},"style":{"padding":3.0,"fill":{"r":0,"b":157,"g":100},"font":{"size":"10pt","weight":"bold","family":"sans-serif"}},"type":"title","content":["Albinism Type: OCA2"]}],"version":"6.0"}

Pie Chart  
Pie Chart - Albinism Type for AlbinismType = OCA2 - May 11, 2022

{"copyright":"(C) Copyright IBM Corp. 2011","grammar":[{"elements":[{"color":[{"field":{"$ref":"fVariable"},"palette":["#00B2EF","#D9182D","#FFCF10","#7F1C7D","#007670","#DD731C","#17AF4B","#EE3D96","#838329","#82D1F5","#F04E37","#FFE14F","#AB1A86","#00A6A0","#F19027","#8CC63F","#F389AF","#A5A215","#00649D","#A91024","#FDB813","#3B2056","#006058","#B8471B","#00BA52","#BA006E","#594F13"],"id":"colorAestheticID"}],"data":{"$ref":"dSource"},"style":{"outline":{"r":0,"b":0,"g":0},"stroke":{"cap":"round","width":0.0,"join":"round"}},"position":[{"field":{"$ref":"fVariable1"}}],"type":"interval"}],"coordinates":{"transforms":[{"type":"stack"},{"polarParameters":{"startAngle":90.0},"type":"polar"}],"style":{"outline":{"a":0.0,"r":0,"b":0,"g":0},"fill":{"r":255,"b":255,"g":255}},"dimensions":[{"axis":[]}]}}],"legends":[{"labelStyle":{"fill":{"r":0,"b":157,"g":100},"font":{"size":"10pt","weight":"normal","family":"sans-serif"}},"itemStyle":{"padding":3,"size":11},"location":{"width":"24%"},"aesthetics":[{"aesthetic":{"$ref":"colorAestheticID"}}],"boundsStyle":{"padding":5,"outline":{"a":0.0,"r":0,"b":0,"g":0},"fill":{"a":0.0,"r":0,"b":0,"g":0}}}],"data":[{"id":"dSource","fields":[{"format":{"numericPattern":"###"},"id":"fVariable","label":"Albinism Type","categories":["OCA2"]},{"min":100.0,"max":100.0,"id":"fVariable1","label":"Y Axis"}],"rows":[[0,100]]}],"size":{"width":850.0,"height":500.0},"style":{"outline":{"a":0.0,"r":0,"b":0,"g":0},"fill":{"r":255,"b":255,"g":255}},"titles":[{"backgroundStyle":{"outline":{"a":0.0,"r":0,"b":0,"g":0},"fill":{"a":0.0,"r":0,"b":0,"g":0}},"style":{"padding":3.0,"fill":{"r":0,"b":157,"g":100},"font":{"size":"12pt","weight":"bold","family":"sans-serif"}},"type":"title","content":["Albinism Type"]},{"backgroundStyle":{"outline":{"a":0.0,"r":0,"b":0,"g":0},"fill":{"a":0.0,"r":0,"b":0,"g":0}},"style":{"padding":3.0,"fill":{"r":0,"b":157,"g":100},"font":{"size":"10pt","weight":"bold","family":"sans-serif"}},"type":"title","content":["Albinism Type: OCA2"]}],"version":"6.0"}

Pie Chart  
Pie Chart - Eye muscle surgery for AlbinismType = OCA2 - May 11, 2022

{"copyright":"(C) Copyright IBM Corp. 2011","grammar":[{"elements":[{"color":[{"field":{"$ref":"fVariable"},"palette":["#00B2EF","#D9182D","#FFCF10","#7F1C7D","#007670","#DD731C","#17AF4B","#EE3D96","#838329","#82D1F5","#F04E37","#FFE14F","#AB1A86","#00A6A0","#F19027","#8CC63F","#F389AF","#A5A215","#00649D","#A91024","#FDB813","#3B2056","#006058","#B8471B","#00BA52","#BA006E","#594F13"],"id":"colorAestheticID"}],"data":{"$ref":"dSource"},"style":{"outline":{"r":0,"b":0,"g":0},"stroke":{"cap":"round","width":0.0,"join":"round"}},"position":[{"field":{"$ref":"fVariable1"}}],"type":"interval"}],"coordinates":{"transforms":[{"type":"stack"},{"polarParameters":{"startAngle":90.0},"type":"polar"}],"style":{"outline":{"a":0.0,"r":0,"b":0,"g":0},"fill":{"r":255,"b":255,"g":255}},"dimensions":[{"axis":[]}]}}],"legends":[{"labelStyle":{"fill":{"r":0,"b":157,"g":100},"font":{"size":"10pt","weight":"normal","family":"sans-serif"}},"itemStyle":{"padding":3,"size":11},"location":{"width":"24%"},"aesthetics":[{"aesthetic":{"$ref":"colorAestheticID"}}],"boundsStyle":{"padding":5,"outline":{"a":0.0,"r":0,"b":0,"g":0},"fill":{"a":0.0,"r":0,"b":0,"g":0}}}],"data":[{"id":"dSource","fields":[{"format":{"numericPattern":"###"},"id":"fVariable","label":"Eye muscle surgery","categories":["No","Yes"]},{"min":46.15384615384615,"max":53.84615384615385,"id":"fVariable1","label":"Y Axis"}],"rows":[[0,53.84615384615385],[1,46.15384615384615]]}],"size":{"width":850.0,"height":500.0},"style":{"outline":{"a":0.0,"r":0,"b":0,"g":0},"fill":{"r":255,"b":255,"g":255}},"titles":[{"backgroundStyle":{"outline":{"a":0.0,"r":0,"b":0,"g":0},"fill":{"a":0.0,"r":0,"b":0,"g":0}},"style":{"padding":3.0,"fill":{"r":0,"b":157,"g":100},"font":{"size":"12pt","weight":"bold","family":"sans-serif"}},"type":"title","content":["Eye muscle surgery"]},{"backgroundStyle":{"outline":{"a":0.0,"r":0,"b":0,"g":0},"fill":{"a":0.0,"r":0,"b":0,"g":0}},"style":{"padding":3.0,"fill":{"r":0,"b":157,"g":100},"font":{"size":"10pt","weight":"bold","family":"sans-serif"}},"type":"title","content":["Albinism Type: OCA2"]}],"version":"6.0"}

Log  
Log - Log - May 11, 2022

FREQUENCIES VARIABLES=RE\_Myopia RE\_referror RE\_cyl LE\_Myopia LE\_referror LE\_cyl  
  /PIECHART PERCENT  
  /ORDER=ANALYSIS.

Frequencies  
Frequencies - Statistics - May 11, 2022

StatisticsStatistics, table, 1 levels of column headers and 3 levels of row headers, table with 9 columns and 8 rows

|  |  |  |  |  |  |  |  |  |
| --- | --- | --- | --- | --- | --- | --- | --- | --- |
| Albinism Type | | | RE\_Myopia | RE\_ref error | RE\_cyl | LE\_Myopia | LE\_ref error | LE\_cyl |
| OCA1A | N | Valid | 17 | 17 | 17 | 17 | 17 | 17 |
|  |  | Missing | 0 | 0 | 0 | 0 | 0 | 0 |
| OCA1B | N | Valid | 28 | 28 | 28 | 27 | 27 | 27 |
|  |  | Missing | 0 | 0 | 0 | 1 | 1 | 1 |
| OCA2 | N | Valid | 26 | 26 | 26 | 26 | 26 | 26 |
|  |  | Missing | 0 | 0 | 0 | 0 | 0 | 0 |
|  |  |  |  |  |  |  |  |  |

Frequency Table  
Frequency Table - RE\_Myopia - May 11, 2022

RE\_MyopiaRE\_Myopia, table, 1 levels of column headers and 3 levels of row headers, table with 7 columns and 11 rows

|  |  |  |  |  |  |  |
| --- | --- | --- | --- | --- | --- | --- |
| Albinism Type | | | Frequency | Percent | Valid Percent | Cumulative Percent |
| OCA1A | Valid | Hyperm | 13 | 76.5 | 76.5 | 76.5 |
|  |  | Myopia | 4 | 23.5 | 23.5 | 100.0 |
|  |  | Total | 17 | 100.0 | 100.0 |  |
| OCA1B | Valid | Hyperm | 23 | 82.1 | 82.1 | 82.1 |
|  |  | Myopia | 5 | 17.9 | 17.9 | 100.0 |
|  |  | Total | 28 | 100.0 | 100.0 |  |
| OCA2 | Valid | Hyperm | 19 | 73.1 | 73.1 | 73.1 |
|  |  | Myopia | 7 | 26.9 | 26.9 | 100.0 |
|  |  | Total | 26 | 100.0 | 100.0 |  |
|  |  |  |  |  |  |  |

Frequency Table  
Frequency Table - RE\_ref error - May 11, 2022

RE\_ref errorRE\_ref error, table, 1 levels of column headers and 3 levels of row headers, table with 7 columns and 11 rows

|  |  |  |  |  |  |  |
| --- | --- | --- | --- | --- | --- | --- |
| Albinism Type | | | Frequency | Percent | Valid Percent | Cumulative Percent |
| OCA1A | Valid | No | 2 | 11.8 | 11.8 | 11.8 |
|  |  | Yes | 15 | 88.2 | 88.2 | 100.0 |
|  |  | Total | 17 | 100.0 | 100.0 |  |
| OCA1B | Valid | No | 10 | 35.7 | 35.7 | 35.7 |
|  |  | Yes | 18 | 64.3 | 64.3 | 100.0 |
|  |  | Total | 28 | 100.0 | 100.0 |  |
| OCA2 | Valid | No | 7 | 26.9 | 26.9 | 26.9 |
|  |  | Yes | 19 | 73.1 | 73.1 | 100.0 |
|  |  | Total | 26 | 100.0 | 100.0 |  |
|  |  |  |  |  |  |  |

Frequency Table  
Frequency Table - RE\_cyl - May 11, 2022

RE\_cylRE\_cyl, table, 1 levels of column headers and 3 levels of row headers, table with 7 columns and 11 rows

|  |  |  |  |  |  |  |
| --- | --- | --- | --- | --- | --- | --- |
| Albinism Type | | | Frequency | Percent | Valid Percent | Cumulative Percent |
| OCA1A | Valid | No | 1 | 5.9 | 5.9 | 5.9 |
|  |  | Yes | 16 | 94.1 | 94.1 | 100.0 |
|  |  | Total | 17 | 100.0 | 100.0 |  |
| OCA1B | Valid | No | 7 | 25.0 | 25.0 | 25.0 |
|  |  | Yes | 21 | 75.0 | 75.0 | 100.0 |
|  |  | Total | 28 | 100.0 | 100.0 |  |
| OCA2 | Valid | No | 6 | 23.1 | 23.1 | 23.1 |
|  |  | Yes | 20 | 76.9 | 76.9 | 100.0 |
|  |  | Total | 26 | 100.0 | 100.0 |  |
|  |  |  |  |  |  |  |

Frequency Table  
Frequency Table - LE\_Myopia - May 11, 2022

LE\_MyopiaLE\_Myopia, table, 1 levels of column headers and 3 levels of row headers, table with 7 columns and 13 rows

|  |  |  |  |  |  |  |
| --- | --- | --- | --- | --- | --- | --- |
| Albinism Type | | | Frequency | Percent | Valid Percent | Cumulative Percent |
| OCA1A | Valid | Hyperm | 13 | 76.5 | 76.5 | 76.5 |
|  |  | Myopia | 4 | 23.5 | 23.5 | 100.0 |
|  |  | Total | 17 | 100.0 | 100.0 |  |
| OCA1B | Valid | Hyperm | 22 | 78.6 | 81.5 | 81.5 |
|  |  | Myopia | 5 | 17.9 | 18.5 | 100.0 |
|  |  | Total | 27 | 96.4 | 100.0 |  |
|  | Missing | System | 1 | 3.6 |  |  |
|  | Total | | 28 | 100.0 |  |  |
| OCA2 | Valid | Hyperm | 20 | 76.9 | 76.9 | 76.9 |
|  |  | Myopia | 6 | 23.1 | 23.1 | 100.0 |
|  |  | Total | 26 | 100.0 | 100.0 |  |
|  |  |  |  |  |  |  |

Frequency Table  
Frequency Table - LE\_ref error - May 11, 2022

LE\_ref errorLE\_ref error, table, 1 levels of column headers and 3 levels of row headers, table with 7 columns and 14 rows

|  |  |  |  |  |  |  |
| --- | --- | --- | --- | --- | --- | --- |
| Albinism Type | | | Frequency | Percent | Valid Percent | Cumulative Percent |
| OCA1A | Valid | No | 1 | 5.9 | 5.9 | 5.9 |
|  |  | Yes | 16 | 94.1 | 94.1 | 100.0 |
|  |  | Total | 17 | 100.0 | 100.0 |  |
| OCA1B | Valid | No | 10 | 35.7 | 37.0 | 37.0 |
|  |  | Yes | 17 | 60.7 | 63.0 | 100.0 |
|  |  | Total | 27 | 96.4 | 100.0 |  |
|  | Missing | System | 1 | 3.6 |  |  |
|  | Total | | 28 | 100.0 |  |  |
| OCA2 | Valid | No | 8 | 30.8 | 30.8 | 30.8 |
|  |  | Yes | 17 | 65.4 | 65.4 | 96.2 |
|  |  | 2 | 1 | 3.8 | 3.8 | 100.0 |
|  |  | Total | 26 | 100.0 | 100.0 |  |
|  |  |  |  |  |  |  |

Frequency Table  
Frequency Table - LE\_cyl - May 11, 2022

LE\_cylLE\_cyl, table, 1 levels of column headers and 3 levels of row headers, table with 7 columns and 13 rows

|  |  |  |  |  |  |  |
| --- | --- | --- | --- | --- | --- | --- |
| Albinism Type | | | Frequency | Percent | Valid Percent | Cumulative Percent |
| OCA1A | Valid | No | 2 | 11.8 | 11.8 | 11.8 |
|  |  | Yes | 15 | 88.2 | 88.2 | 100.0 |
|  |  | Total | 17 | 100.0 | 100.0 |  |
| OCA1B | Valid | No | 8 | 28.6 | 29.6 | 29.6 |
|  |  | Yes | 19 | 67.9 | 70.4 | 100.0 |
|  |  | Total | 27 | 96.4 | 100.0 |  |
|  | Missing | System | 1 | 3.6 |  |  |
|  | Total | | 28 | 100.0 |  |  |
| OCA2 | Valid | No | 5 | 19.2 | 19.2 | 19.2 |
|  |  | Yes | 21 | 80.8 | 80.8 | 100.0 |
|  |  | Total | 26 | 100.0 | 100.0 |  |
|  |  |  |  |  |  |  |

Pie Chart  
Pie Chart - RE\_Myopia for AlbinismType = OCA1A - May 11, 2022

{"copyright":"(C) Copyright IBM Corp. 2011","grammar":[{"elements":[{"color":[{"field":{"$ref":"fVariable"},"palette":["#00B2EF","#D9182D","#FFCF10","#7F1C7D","#007670","#DD731C","#17AF4B","#EE3D96","#838329","#82D1F5","#F04E37","#FFE14F","#AB1A86","#00A6A0","#F19027","#8CC63F","#F389AF","#A5A215","#00649D","#A91024","#FDB813","#3B2056","#006058","#B8471B","#00BA52","#BA006E","#594F13"],"id":"colorAestheticID"}],"data":{"$ref":"dSource"},"style":{"outline":{"r":0,"b":0,"g":0},"stroke":{"cap":"round","width":0.0,"join":"round"}},"position":[{"field":{"$ref":"fVariable1"}}],"type":"interval"}],"coordinates":{"transforms":[{"type":"stack"},{"polarParameters":{"startAngle":90.0},"type":"polar"}],"style":{"outline":{"a":0.0,"r":0,"b":0,"g":0},"fill":{"r":255,"b":255,"g":255}},"dimensions":[{"axis":[]}]}}],"legends":[{"labelStyle":{"fill":{"r":0,"b":157,"g":100},"font":{"size":"10pt","weight":"normal","family":"sans-serif"}},"itemStyle":{"padding":3,"size":11},"location":{"width":"24%"},"aesthetics":[{"aesthetic":{"$ref":"colorAestheticID"}}],"boundsStyle":{"padding":5,"outline":{"a":0.0,"r":0,"b":0,"g":0},"fill":{"a":0.0,"r":0,"b":0,"g":0}}}],"data":[{"id":"dSource","fields":[{"format":{"numericPattern":"###"},"id":"fVariable","label":"V4","categories":["Hyperm","Myopia"]},{"min":23.52941176470588,"max":76.47058823529412,"id":"fVariable1","label":"Y Axis"}],"rows":[[0,76.47058823529412],[1,23.52941176470588]]}],"size":{"width":850.0,"height":500.0},"style":{"outline":{"a":0.0,"r":0,"b":0,"g":0},"fill":{"r":255,"b":255,"g":255}},"titles":[{"backgroundStyle":{"outline":{"a":0.0,"r":0,"b":0,"g":0},"fill":{"a":0.0,"r":0,"b":0,"g":0}},"style":{"padding":3.0,"fill":{"r":0,"b":157,"g":100},"font":{"size":"12pt","weight":"bold","family":"sans-serif"}},"type":"title","content":["RE\_Myopia"]},{"backgroundStyle":{"outline":{"a":0.0,"r":0,"b":0,"g":0},"fill":{"a":0.0,"r":0,"b":0,"g":0}},"style":{"padding":3.0,"fill":{"r":0,"b":157,"g":100},"font":{"size":"10pt","weight":"bold","family":"sans-serif"}},"type":"title","content":["Albinism Type: OCA1A"]}],"version":"6.0"}

Pie Chart  
Pie Chart - RE\_ref error for AlbinismType = OCA1A - May 11, 2022

{"copyright":"(C) Copyright IBM Corp. 2011","grammar":[{"elements":[{"color":[{"field":{"$ref":"fVariable"},"palette":["#00B2EF","#D9182D","#FFCF10","#7F1C7D","#007670","#DD731C","#17AF4B","#EE3D96","#838329","#82D1F5","#F04E37","#FFE14F","#AB1A86","#00A6A0","#F19027","#8CC63F","#F389AF","#A5A215","#00649D","#A91024","#FDB813","#3B2056","#006058","#B8471B","#00BA52","#BA006E","#594F13"],"id":"colorAestheticID"}],"data":{"$ref":"dSource"},"style":{"outline":{"r":0,"b":0,"g":0},"stroke":{"cap":"round","width":0.0,"join":"round"}},"position":[{"field":{"$ref":"fVariable1"}}],"type":"interval"}],"coordinates":{"transforms":[{"type":"stack"},{"polarParameters":{"startAngle":90.0},"type":"polar"}],"style":{"outline":{"a":0.0,"r":0,"b":0,"g":0},"fill":{"r":255,"b":255,"g":255}},"dimensions":[{"axis":[]}]}}],"legends":[{"labelStyle":{"fill":{"r":0,"b":157,"g":100},"font":{"size":"10pt","weight":"normal","family":"sans-serif"}},"itemStyle":{"padding":3,"size":11},"location":{"width":"24%"},"aesthetics":[{"aesthetic":{"$ref":"colorAestheticID"}}],"boundsStyle":{"padding":5,"outline":{"a":0.0,"r":0,"b":0,"g":0},"fill":{"a":0.0,"r":0,"b":0,"g":0}}}],"data":[{"id":"dSource","fields":[{"format":{"numericPattern":"###"},"id":"fVariable","label":"RE\_ref error","categories":["No","Yes"]},{"min":11.76470588235294,"max":88.23529411764706,"id":"fVariable1","label":"Y Axis"}],"rows":[[0,11.76470588235294],[1,88.23529411764706]]}],"size":{"width":850.0,"height":500.0},"style":{"outline":{"a":0.0,"r":0,"b":0,"g":0},"fill":{"r":255,"b":255,"g":255}},"titles":[{"backgroundStyle":{"outline":{"a":0.0,"r":0,"b":0,"g":0},"fill":{"a":0.0,"r":0,"b":0,"g":0}},"style":{"padding":3.0,"fill":{"r":0,"b":157,"g":100},"font":{"size":"12pt","weight":"bold","family":"sans-serif"}},"type":"title","content":["RE\_ref error"]},{"backgroundStyle":{"outline":{"a":0.0,"r":0,"b":0,"g":0},"fill":{"a":0.0,"r":0,"b":0,"g":0}},"style":{"padding":3.0,"fill":{"r":0,"b":157,"g":100},"font":{"size":"10pt","weight":"bold","family":"sans-serif"}},"type":"title","content":["Albinism Type: OCA1A"]}],"version":"6.0"}

Pie Chart  
Pie Chart - RE\_cyl for AlbinismType = OCA1A - May 11, 2022

{"copyright":"(C) Copyright IBM Corp. 2011","grammar":[{"elements":[{"color":[{"field":{"$ref":"fVariable"},"palette":["#00B2EF","#D9182D","#FFCF10","#7F1C7D","#007670","#DD731C","#17AF4B","#EE3D96","#838329","#82D1F5","#F04E37","#FFE14F","#AB1A86","#00A6A0","#F19027","#8CC63F","#F389AF","#A5A215","#00649D","#A91024","#FDB813","#3B2056","#006058","#B8471B","#00BA52","#BA006E","#594F13"],"id":"colorAestheticID"}],"data":{"$ref":"dSource"},"style":{"outline":{"r":0,"b":0,"g":0},"stroke":{"cap":"round","width":0.0,"join":"round"}},"position":[{"field":{"$ref":"fVariable1"}}],"type":"interval"}],"coordinates":{"transforms":[{"type":"stack"},{"polarParameters":{"startAngle":90.0},"type":"polar"}],"style":{"outline":{"a":0.0,"r":0,"b":0,"g":0},"fill":{"r":255,"b":255,"g":255}},"dimensions":[{"axis":[]}]}}],"legends":[{"labelStyle":{"fill":{"r":0,"b":157,"g":100},"font":{"size":"10pt","weight":"normal","family":"sans-serif"}},"itemStyle":{"padding":3,"size":11},"location":{"width":"24%"},"aesthetics":[{"aesthetic":{"$ref":"colorAestheticID"}}],"boundsStyle":{"padding":5,"outline":{"a":0.0,"r":0,"b":0,"g":0},"fill":{"a":0.0,"r":0,"b":0,"g":0}}}],"data":[{"id":"dSource","fields":[{"format":{"numericPattern":"###"},"id":"fVariable","label":"V4","categories":["No","Yes"]},{"min":5.88235294117647,"max":94.11764705882352,"id":"fVariable1","label":"Y Axis"}],"rows":[[0,5.88235294117647],[1,94.11764705882352]]}],"size":{"width":850.0,"height":500.0},"style":{"outline":{"a":0.0,"r":0,"b":0,"g":0},"fill":{"r":255,"b":255,"g":255}},"titles":[{"backgroundStyle":{"outline":{"a":0.0,"r":0,"b":0,"g":0},"fill":{"a":0.0,"r":0,"b":0,"g":0}},"style":{"padding":3.0,"fill":{"r":0,"b":157,"g":100},"font":{"size":"12pt","weight":"bold","family":"sans-serif"}},"type":"title","content":["RE\_cyl"]},{"backgroundStyle":{"outline":{"a":0.0,"r":0,"b":0,"g":0},"fill":{"a":0.0,"r":0,"b":0,"g":0}},"style":{"padding":3.0,"fill":{"r":0,"b":157,"g":100},"font":{"size":"10pt","weight":"bold","family":"sans-serif"}},"type":"title","content":["Albinism Type: OCA1A"]}],"version":"6.0"}

Pie Chart  
Pie Chart - LE\_Myopia for AlbinismType = OCA1A - May 11, 2022

{"copyright":"(C) Copyright IBM Corp. 2011","grammar":[{"elements":[{"color":[{"field":{"$ref":"fVariable"},"palette":["#00B2EF","#D9182D","#FFCF10","#7F1C7D","#007670","#DD731C","#17AF4B","#EE3D96","#838329","#82D1F5","#F04E37","#FFE14F","#AB1A86","#00A6A0","#F19027","#8CC63F","#F389AF","#A5A215","#00649D","#A91024","#FDB813","#3B2056","#006058","#B8471B","#00BA52","#BA006E","#594F13"],"id":"colorAestheticID"}],"data":{"$ref":"dSource"},"style":{"outline":{"r":0,"b":0,"g":0},"stroke":{"cap":"round","width":0.0,"join":"round"}},"position":[{"field":{"$ref":"fVariable1"}}],"type":"interval"}],"coordinates":{"transforms":[{"type":"stack"},{"polarParameters":{"startAngle":90.0},"type":"polar"}],"style":{"outline":{"a":0.0,"r":0,"b":0,"g":0},"fill":{"r":255,"b":255,"g":255}},"dimensions":[{"axis":[]}]}}],"legends":[{"labelStyle":{"fill":{"r":0,"b":157,"g":100},"font":{"size":"10pt","weight":"normal","family":"sans-serif"}},"itemStyle":{"padding":3,"size":11},"location":{"width":"24%"},"aesthetics":[{"aesthetic":{"$ref":"colorAestheticID"}}],"boundsStyle":{"padding":5,"outline":{"a":0.0,"r":0,"b":0,"g":0},"fill":{"a":0.0,"r":0,"b":0,"g":0}}}],"data":[{"id":"dSource","fields":[{"format":{"numericPattern":"###"},"id":"fVariable","label":"V4","categories":["Hyperm","Myopia"]},{"min":23.52941176470588,"max":76.47058823529412,"id":"fVariable1","label":"Y Axis"}],"rows":[[0,76.47058823529412],[1,23.52941176470588]]}],"size":{"width":850.0,"height":500.0},"style":{"outline":{"a":0.0,"r":0,"b":0,"g":0},"fill":{"r":255,"b":255,"g":255}},"titles":[{"backgroundStyle":{"outline":{"a":0.0,"r":0,"b":0,"g":0},"fill":{"a":0.0,"r":0,"b":0,"g":0}},"style":{"padding":3.0,"fill":{"r":0,"b":157,"g":100},"font":{"size":"12pt","weight":"bold","family":"sans-serif"}},"type":"title","content":["LE\_Myopia"]},{"backgroundStyle":{"outline":{"a":0.0,"r":0,"b":0,"g":0},"fill":{"a":0.0,"r":0,"b":0,"g":0}},"style":{"padding":3.0,"fill":{"r":0,"b":157,"g":100},"font":{"size":"10pt","weight":"bold","family":"sans-serif"}},"type":"title","content":["Albinism Type: OCA1A"]}],"version":"6.0"}

Pie Chart  
Pie Chart - LE\_ref error for AlbinismType = OCA1A - May 11, 2022

{"copyright":"(C) Copyright IBM Corp. 2011","grammar":[{"elements":[{"color":[{"field":{"$ref":"fVariable"},"palette":["#00B2EF","#D9182D","#FFCF10","#7F1C7D","#007670","#DD731C","#17AF4B","#EE3D96","#838329","#82D1F5","#F04E37","#FFE14F","#AB1A86","#00A6A0","#F19027","#8CC63F","#F389AF","#A5A215","#00649D","#A91024","#FDB813","#3B2056","#006058","#B8471B","#00BA52","#BA006E","#594F13"],"id":"colorAestheticID"}],"data":{"$ref":"dSource"},"style":{"outline":{"r":0,"b":0,"g":0},"stroke":{"cap":"round","width":0.0,"join":"round"}},"position":[{"field":{"$ref":"fVariable1"}}],"type":"interval"}],"coordinates":{"transforms":[{"type":"stack"},{"polarParameters":{"startAngle":90.0},"type":"polar"}],"style":{"outline":{"a":0.0,"r":0,"b":0,"g":0},"fill":{"r":255,"b":255,"g":255}},"dimensions":[{"axis":[]}]}}],"legends":[{"labelStyle":{"fill":{"r":0,"b":157,"g":100},"font":{"size":"10pt","weight":"normal","family":"sans-serif"}},"itemStyle":{"padding":3,"size":11},"location":{"width":"24%"},"aesthetics":[{"aesthetic":{"$ref":"colorAestheticID"}}],"boundsStyle":{"padding":5,"outline":{"a":0.0,"r":0,"b":0,"g":0},"fill":{"a":0.0,"r":0,"b":0,"g":0}}}],"data":[{"id":"dSource","fields":[{"format":{"numericPattern":"###"},"id":"fVariable","label":"LE\_ref error","categories":["No","Yes"]},{"min":5.88235294117647,"max":94.11764705882352,"id":"fVariable1","label":"Y Axis"}],"rows":[[0,5.88235294117647],[1,94.11764705882352]]}],"size":{"width":850.0,"height":500.0},"style":{"outline":{"a":0.0,"r":0,"b":0,"g":0},"fill":{"r":255,"b":255,"g":255}},"titles":[{"backgroundStyle":{"outline":{"a":0.0,"r":0,"b":0,"g":0},"fill":{"a":0.0,"r":0,"b":0,"g":0}},"style":{"padding":3.0,"fill":{"r":0,"b":157,"g":100},"font":{"size":"12pt","weight":"bold","family":"sans-serif"}},"type":"title","content":["LE\_ref error"]},{"backgroundStyle":{"outline":{"a":0.0,"r":0,"b":0,"g":0},"fill":{"a":0.0,"r":0,"b":0,"g":0}},"style":{"padding":3.0,"fill":{"r":0,"b":157,"g":100},"font":{"size":"10pt","weight":"bold","family":"sans-serif"}},"type":"title","content":["Albinism Type: OCA1A"]}],"version":"6.0"}

Pie Chart  
Pie Chart - LE\_cyl for AlbinismType = OCA1A - May 11, 2022

{"copyright":"(C) Copyright IBM Corp. 2011","grammar":[{"elements":[{"color":[{"field":{"$ref":"fVariable"},"palette":["#00B2EF","#D9182D","#FFCF10","#7F1C7D","#007670","#DD731C","#17AF4B","#EE3D96","#838329","#82D1F5","#F04E37","#FFE14F","#AB1A86","#00A6A0","#F19027","#8CC63F","#F389AF","#A5A215","#00649D","#A91024","#FDB813","#3B2056","#006058","#B8471B","#00BA52","#BA006E","#594F13"],"id":"colorAestheticID"}],"data":{"$ref":"dSource"},"style":{"outline":{"r":0,"b":0,"g":0},"stroke":{"cap":"round","width":0.0,"join":"round"}},"position":[{"field":{"$ref":"fVariable1"}}],"type":"interval"}],"coordinates":{"transforms":[{"type":"stack"},{"polarParameters":{"startAngle":90.0},"type":"polar"}],"style":{"outline":{"a":0.0,"r":0,"b":0,"g":0},"fill":{"r":255,"b":255,"g":255}},"dimensions":[{"axis":[]}]}}],"legends":[{"labelStyle":{"fill":{"r":0,"b":157,"g":100},"font":{"size":"10pt","weight":"normal","family":"sans-serif"}},"itemStyle":{"padding":3,"size":11},"location":{"width":"24%"},"aesthetics":[{"aesthetic":{"$ref":"colorAestheticID"}}],"boundsStyle":{"padding":5,"outline":{"a":0.0,"r":0,"b":0,"g":0},"fill":{"a":0.0,"r":0,"b":0,"g":0}}}],"data":[{"id":"dSource","fields":[{"format":{"numericPattern":"###"},"id":"fVariable","label":"V4","categories":["No","Yes"]},{"min":11.76470588235294,"max":88.23529411764706,"id":"fVariable1","label":"Y Axis"}],"rows":[[0,11.76470588235294],[1,88.23529411764706]]}],"size":{"width":850.0,"height":500.0},"style":{"outline":{"a":0.0,"r":0,"b":0,"g":0},"fill":{"r":255,"b":255,"g":255}},"titles":[{"backgroundStyle":{"outline":{"a":0.0,"r":0,"b":0,"g":0},"fill":{"a":0.0,"r":0,"b":0,"g":0}},"style":{"padding":3.0,"fill":{"r":0,"b":157,"g":100},"font":{"size":"12pt","weight":"bold","family":"sans-serif"}},"type":"title","content":["LE\_cyl"]},{"backgroundStyle":{"outline":{"a":0.0,"r":0,"b":0,"g":0},"fill":{"a":0.0,"r":0,"b":0,"g":0}},"style":{"padding":3.0,"fill":{"r":0,"b":157,"g":100},"font":{"size":"10pt","weight":"bold","family":"sans-serif"}},"type":"title","content":["Albinism Type: OCA1A"]}],"version":"6.0"}

Pie Chart  
Pie Chart - RE\_Myopia for AlbinismType = OCA1B - May 11, 2022

{"copyright":"(C) Copyright IBM Corp. 2011","grammar":[{"elements":[{"color":[{"field":{"$ref":"fVariable"},"palette":["#00B2EF","#D9182D","#FFCF10","#7F1C7D","#007670","#DD731C","#17AF4B","#EE3D96","#838329","#82D1F5","#F04E37","#FFE14F","#AB1A86","#00A6A0","#F19027","#8CC63F","#F389AF","#A5A215","#00649D","#A91024","#FDB813","#3B2056","#006058","#B8471B","#00BA52","#BA006E","#594F13"],"id":"colorAestheticID"}],"data":{"$ref":"dSource"},"style":{"outline":{"r":0,"b":0,"g":0},"stroke":{"cap":"round","width":0.0,"join":"round"}},"position":[{"field":{"$ref":"fVariable1"}}],"type":"interval"}],"coordinates":{"transforms":[{"type":"stack"},{"polarParameters":{"startAngle":90.0},"type":"polar"}],"style":{"outline":{"a":0.0,"r":0,"b":0,"g":0},"fill":{"r":255,"b":255,"g":255}},"dimensions":[{"axis":[]}]}}],"legends":[{"labelStyle":{"fill":{"r":0,"b":157,"g":100},"font":{"size":"10pt","weight":"normal","family":"sans-serif"}},"itemStyle":{"padding":3,"size":11},"location":{"width":"24%"},"aesthetics":[{"aesthetic":{"$ref":"colorAestheticID"}}],"boundsStyle":{"padding":5,"outline":{"a":0.0,"r":0,"b":0,"g":0},"fill":{"a":0.0,"r":0,"b":0,"g":0}}}],"data":[{"id":"dSource","fields":[{"format":{"numericPattern":"###"},"id":"fVariable","label":"V4","categories":["Hyperm","Myopia"]},{"min":17.85714285714286,"max":82.14285714285714,"id":"fVariable1","label":"Y Axis"}],"rows":[[0,82.14285714285714],[1,17.85714285714286]]}],"size":{"width":850.0,"height":500.0},"style":{"outline":{"a":0.0,"r":0,"b":0,"g":0},"fill":{"r":255,"b":255,"g":255}},"titles":[{"backgroundStyle":{"outline":{"a":0.0,"r":0,"b":0,"g":0},"fill":{"a":0.0,"r":0,"b":0,"g":0}},"style":{"padding":3.0,"fill":{"r":0,"b":157,"g":100},"font":{"size":"12pt","weight":"bold","family":"sans-serif"}},"type":"title","content":["RE\_Myopia"]},{"backgroundStyle":{"outline":{"a":0.0,"r":0,"b":0,"g":0},"fill":{"a":0.0,"r":0,"b":0,"g":0}},"style":{"padding":3.0,"fill":{"r":0,"b":157,"g":100},"font":{"size":"10pt","weight":"bold","family":"sans-serif"}},"type":"title","content":["Albinism Type: OCA1B"]}],"version":"6.0"}

Pie Chart  
Pie Chart - RE\_ref error for AlbinismType = OCA1B - May 11, 2022

{"copyright":"(C) Copyright IBM Corp. 2011","grammar":[{"elements":[{"color":[{"field":{"$ref":"fVariable"},"palette":["#00B2EF","#D9182D","#FFCF10","#7F1C7D","#007670","#DD731C","#17AF4B","#EE3D96","#838329","#82D1F5","#F04E37","#FFE14F","#AB1A86","#00A6A0","#F19027","#8CC63F","#F389AF","#A5A215","#00649D","#A91024","#FDB813","#3B2056","#006058","#B8471B","#00BA52","#BA006E","#594F13"],"id":"colorAestheticID"}],"data":{"$ref":"dSource"},"style":{"outline":{"r":0,"b":0,"g":0},"stroke":{"cap":"round","width":0.0,"join":"round"}},"position":[{"field":{"$ref":"fVariable1"}}],"type":"interval"}],"coordinates":{"transforms":[{"type":"stack"},{"polarParameters":{"startAngle":90.0},"type":"polar"}],"style":{"outline":{"a":0.0,"r":0,"b":0,"g":0},"fill":{"r":255,"b":255,"g":255}},"dimensions":[{"axis":[]}]}}],"legends":[{"labelStyle":{"fill":{"r":0,"b":157,"g":100},"font":{"size":"10pt","weight":"normal","family":"sans-serif"}},"itemStyle":{"padding":3,"size":11},"location":{"width":"24%"},"aesthetics":[{"aesthetic":{"$ref":"colorAestheticID"}}],"boundsStyle":{"padding":5,"outline":{"a":0.0,"r":0,"b":0,"g":0},"fill":{"a":0.0,"r":0,"b":0,"g":0}}}],"data":[{"id":"dSource","fields":[{"format":{"numericPattern":"###"},"id":"fVariable","label":"RE\_ref error","categories":["No","Yes"]},{"min":35.71428571428572,"max":64.28571428571429,"id":"fVariable1","label":"Y Axis"}],"rows":[[0,35.71428571428572],[1,64.28571428571429]]}],"size":{"width":850.0,"height":500.0},"style":{"outline":{"a":0.0,"r":0,"b":0,"g":0},"fill":{"r":255,"b":255,"g":255}},"titles":[{"backgroundStyle":{"outline":{"a":0.0,"r":0,"b":0,"g":0},"fill":{"a":0.0,"r":0,"b":0,"g":0}},"style":{"padding":3.0,"fill":{"r":0,"b":157,"g":100},"font":{"size":"12pt","weight":"bold","family":"sans-serif"}},"type":"title","content":["RE\_ref error"]},{"backgroundStyle":{"outline":{"a":0.0,"r":0,"b":0,"g":0},"fill":{"a":0.0,"r":0,"b":0,"g":0}},"style":{"padding":3.0,"fill":{"r":0,"b":157,"g":100},"font":{"size":"10pt","weight":"bold","family":"sans-serif"}},"type":"title","content":["Albinism Type: OCA1B"]}],"version":"6.0"}

Pie Chart  
Pie Chart - RE\_cyl for AlbinismType = OCA1B - May 11, 2022

{"copyright":"(C) Copyright IBM Corp. 2011","grammar":[{"elements":[{"color":[{"field":{"$ref":"fVariable"},"palette":["#00B2EF","#D9182D","#FFCF10","#7F1C7D","#007670","#DD731C","#17AF4B","#EE3D96","#838329","#82D1F5","#F04E37","#FFE14F","#AB1A86","#00A6A0","#F19027","#8CC63F","#F389AF","#A5A215","#00649D","#A91024","#FDB813","#3B2056","#006058","#B8471B","#00BA52","#BA006E","#594F13"],"id":"colorAestheticID"}],"data":{"$ref":"dSource"},"style":{"outline":{"r":0,"b":0,"g":0},"stroke":{"cap":"round","width":0.0,"join":"round"}},"position":[{"field":{"$ref":"fVariable1"}}],"type":"interval"}],"coordinates":{"transforms":[{"type":"stack"},{"polarParameters":{"startAngle":90.0},"type":"polar"}],"style":{"outline":{"a":0.0,"r":0,"b":0,"g":0},"fill":{"r":255,"b":255,"g":255}},"dimensions":[{"axis":[]}]}}],"legends":[{"labelStyle":{"fill":{"r":0,"b":157,"g":100},"font":{"size":"10pt","weight":"normal","family":"sans-serif"}},"itemStyle":{"padding":3,"size":11},"location":{"width":"24%"},"aesthetics":[{"aesthetic":{"$ref":"colorAestheticID"}}],"boundsStyle":{"padding":5,"outline":{"a":0.0,"r":0,"b":0,"g":0},"fill":{"a":0.0,"r":0,"b":0,"g":0}}}],"data":[{"id":"dSource","fields":[{"format":{"numericPattern":"###"},"id":"fVariable","label":"V4","categories":["No","Yes"]},{"min":25.0,"max":75.0,"id":"fVariable1","label":"Y Axis"}],"rows":[[0,25],[1,75]]}],"size":{"width":850.0,"height":500.0},"style":{"outline":{"a":0.0,"r":0,"b":0,"g":0},"fill":{"r":255,"b":255,"g":255}},"titles":[{"backgroundStyle":{"outline":{"a":0.0,"r":0,"b":0,"g":0},"fill":{"a":0.0,"r":0,"b":0,"g":0}},"style":{"padding":3.0,"fill":{"r":0,"b":157,"g":100},"font":{"size":"12pt","weight":"bold","family":"sans-serif"}},"type":"title","content":["RE\_cyl"]},{"backgroundStyle":{"outline":{"a":0.0,"r":0,"b":0,"g":0},"fill":{"a":0.0,"r":0,"b":0,"g":0}},"style":{"padding":3.0,"fill":{"r":0,"b":157,"g":100},"font":{"size":"10pt","weight":"bold","family":"sans-serif"}},"type":"title","content":["Albinism Type: OCA1B"]}],"version":"6.0"}

Pie Chart  
Pie Chart - LE\_Myopia for AlbinismType = OCA1B - May 11, 2022

{"copyright":"(C) Copyright IBM Corp. 2011","grammar":[{"elements":[{"color":[{"field":{"$ref":"fVariable"},"palette":["#00B2EF","#D9182D","#FFCF10","#7F1C7D","#007670","#DD731C","#17AF4B","#EE3D96","#838329","#82D1F5","#F04E37","#FFE14F","#AB1A86","#00A6A0","#F19027","#8CC63F","#F389AF","#A5A215","#00649D","#A91024","#FDB813","#3B2056","#006058","#B8471B","#00BA52","#BA006E","#594F13"],"id":"colorAestheticID"}],"data":{"$ref":"dSource"},"style":{"outline":{"r":0,"b":0,"g":0},"stroke":{"cap":"round","width":0.0,"join":"round"}},"position":[{"field":{"$ref":"fVariable1"}}],"type":"interval"}],"coordinates":{"transforms":[{"type":"stack"},{"polarParameters":{"startAngle":90.0},"type":"polar"}],"style":{"outline":{"a":0.0,"r":0,"b":0,"g":0},"fill":{"r":255,"b":255,"g":255}},"dimensions":[{"axis":[]}]}}],"legends":[{"labelStyle":{"fill":{"r":0,"b":157,"g":100},"font":{"size":"10pt","weight":"normal","family":"sans-serif"}},"itemStyle":{"padding":3,"size":11},"location":{"width":"24%"},"aesthetics":[{"aesthetic":{"$ref":"colorAestheticID"}}],"boundsStyle":{"padding":5,"outline":{"a":0.0,"r":0,"b":0,"g":0},"fill":{"a":0.0,"r":0,"b":0,"g":0}}}],"data":[{"id":"dSource","fields":[{"format":{"numericPattern":"###"},"id":"fVariable","label":"V4","categories":["Hyperm","Myopia"]},{"min":18.51851851851852,"max":81.48148148148148,"id":"fVariable1","label":"Y Axis"}],"rows":[[0,81.48148148148148],[1,18.51851851851852]]}],"size":{"width":850.0,"height":500.0},"style":{"outline":{"a":0.0,"r":0,"b":0,"g":0},"fill":{"r":255,"b":255,"g":255}},"titles":[{"backgroundStyle":{"outline":{"a":0.0,"r":0,"b":0,"g":0},"fill":{"a":0.0,"r":0,"b":0,"g":0}},"style":{"padding":3.0,"fill":{"r":0,"b":157,"g":100},"font":{"size":"12pt","weight":"bold","family":"sans-serif"}},"type":"title","content":["LE\_Myopia"]},{"backgroundStyle":{"outline":{"a":0.0,"r":0,"b":0,"g":0},"fill":{"a":0.0,"r":0,"b":0,"g":0}},"style":{"padding":3.0,"fill":{"r":0,"b":157,"g":100},"font":{"size":"10pt","weight":"bold","family":"sans-serif"}},"type":"title","content":["Albinism Type: OCA1B"]}],"version":"6.0"}

Pie Chart  
Pie Chart - LE\_ref error for AlbinismType = OCA1B - May 11, 2022

{"copyright":"(C) Copyright IBM Corp. 2011","grammar":[{"elements":[{"color":[{"field":{"$ref":"fVariable"},"palette":["#00B2EF","#D9182D","#FFCF10","#7F1C7D","#007670","#DD731C","#17AF4B","#EE3D96","#838329","#82D1F5","#F04E37","#FFE14F","#AB1A86","#00A6A0","#F19027","#8CC63F","#F389AF","#A5A215","#00649D","#A91024","#FDB813","#3B2056","#006058","#B8471B","#00BA52","#BA006E","#594F13"],"id":"colorAestheticID"}],"data":{"$ref":"dSource"},"style":{"outline":{"r":0,"b":0,"g":0},"stroke":{"cap":"round","width":0.0,"join":"round"}},"position":[{"field":{"$ref":"fVariable1"}}],"type":"interval"}],"coordinates":{"transforms":[{"type":"stack"},{"polarParameters":{"startAngle":90.0},"type":"polar"}],"style":{"outline":{"a":0.0,"r":0,"b":0,"g":0},"fill":{"r":255,"b":255,"g":255}},"dimensions":[{"axis":[]}]}}],"legends":[{"labelStyle":{"fill":{"r":0,"b":157,"g":100},"font":{"size":"10pt","weight":"normal","family":"sans-serif"}},"itemStyle":{"padding":3,"size":11},"location":{"width":"24%"},"aesthetics":[{"aesthetic":{"$ref":"colorAestheticID"}}],"boundsStyle":{"padding":5,"outline":{"a":0.0,"r":0,"b":0,"g":0},"fill":{"a":0.0,"r":0,"b":0,"g":0}}}],"data":[{"id":"dSource","fields":[{"format":{"numericPattern":"###"},"id":"fVariable","label":"LE\_ref error","categories":["No","Yes"]},{"min":37.03703703703704,"max":62.96296296296296,"id":"fVariable1","label":"Y Axis"}],"rows":[[0,37.03703703703704],[1,62.96296296296296]]}],"size":{"width":850.0,"height":500.0},"style":{"outline":{"a":0.0,"r":0,"b":0,"g":0},"fill":{"r":255,"b":255,"g":255}},"titles":[{"backgroundStyle":{"outline":{"a":0.0,"r":0,"b":0,"g":0},"fill":{"a":0.0,"r":0,"b":0,"g":0}},"style":{"padding":3.0,"fill":{"r":0,"b":157,"g":100},"font":{"size":"12pt","weight":"bold","family":"sans-serif"}},"type":"title","content":["LE\_ref error"]},{"backgroundStyle":{"outline":{"a":0.0,"r":0,"b":0,"g":0},"fill":{"a":0.0,"r":0,"b":0,"g":0}},"style":{"padding":3.0,"fill":{"r":0,"b":157,"g":100},"font":{"size":"10pt","weight":"bold","family":"sans-serif"}},"type":"title","content":["Albinism Type: OCA1B"]}],"version":"6.0"}

Pie Chart  
Pie Chart - LE\_cyl for AlbinismType = OCA1B - May 11, 2022

{"copyright":"(C) Copyright IBM Corp. 2011","grammar":[{"elements":[{"color":[{"field":{"$ref":"fVariable"},"palette":["#00B2EF","#D9182D","#FFCF10","#7F1C7D","#007670","#DD731C","#17AF4B","#EE3D96","#838329","#82D1F5","#F04E37","#FFE14F","#AB1A86","#00A6A0","#F19027","#8CC63F","#F389AF","#A5A215","#00649D","#A91024","#FDB813","#3B2056","#006058","#B8471B","#00BA52","#BA006E","#594F13"],"id":"colorAestheticID"}],"data":{"$ref":"dSource"},"style":{"outline":{"r":0,"b":0,"g":0},"stroke":{"cap":"round","width":0.0,"join":"round"}},"position":[{"field":{"$ref":"fVariable1"}}],"type":"interval"}],"coordinates":{"transforms":[{"type":"stack"},{"polarParameters":{"startAngle":90.0},"type":"polar"}],"style":{"outline":{"a":0.0,"r":0,"b":0,"g":0},"fill":{"r":255,"b":255,"g":255}},"dimensions":[{"axis":[]}]}}],"legends":[{"labelStyle":{"fill":{"r":0,"b":157,"g":100},"font":{"size":"10pt","weight":"normal","family":"sans-serif"}},"itemStyle":{"padding":3,"size":11},"location":{"width":"24%"},"aesthetics":[{"aesthetic":{"$ref":"colorAestheticID"}}],"boundsStyle":{"padding":5,"outline":{"a":0.0,"r":0,"b":0,"g":0},"fill":{"a":0.0,"r":0,"b":0,"g":0}}}],"data":[{"id":"dSource","fields":[{"format":{"numericPattern":"###"},"id":"fVariable","label":"V4","categories":["No","Yes"]},{"min":29.62962962962963,"max":70.37037037037037,"id":"fVariable1","label":"Y Axis"}],"rows":[[0,29.62962962962963],[1,70.37037037037037]]}],"size":{"width":850.0,"height":500.0},"style":{"outline":{"a":0.0,"r":0,"b":0,"g":0},"fill":{"r":255,"b":255,"g":255}},"titles":[{"backgroundStyle":{"outline":{"a":0.0,"r":0,"b":0,"g":0},"fill":{"a":0.0,"r":0,"b":0,"g":0}},"style":{"padding":3.0,"fill":{"r":0,"b":157,"g":100},"font":{"size":"12pt","weight":"bold","family":"sans-serif"}},"type":"title","content":["LE\_cyl"]},{"backgroundStyle":{"outline":{"a":0.0,"r":0,"b":0,"g":0},"fill":{"a":0.0,"r":0,"b":0,"g":0}},"style":{"padding":3.0,"fill":{"r":0,"b":157,"g":100},"font":{"size":"10pt","weight":"bold","family":"sans-serif"}},"type":"title","content":["Albinism Type: OCA1B"]}],"version":"6.0"}

Pie Chart  
Pie Chart - RE\_Myopia for AlbinismType = OCA2 - May 11, 2022

{"copyright":"(C) Copyright IBM Corp. 2011","grammar":[{"elements":[{"color":[{"field":{"$ref":"fVariable"},"palette":["#00B2EF","#D9182D","#FFCF10","#7F1C7D","#007670","#DD731C","#17AF4B","#EE3D96","#838329","#82D1F5","#F04E37","#FFE14F","#AB1A86","#00A6A0","#F19027","#8CC63F","#F389AF","#A5A215","#00649D","#A91024","#FDB813","#3B2056","#006058","#B8471B","#00BA52","#BA006E","#594F13"],"id":"colorAestheticID"}],"data":{"$ref":"dSource"},"style":{"outline":{"r":0,"b":0,"g":0},"stroke":{"cap":"round","width":0.0,"join":"round"}},"position":[{"field":{"$ref":"fVariable1"}}],"type":"interval"}],"coordinates":{"transforms":[{"type":"stack"},{"polarParameters":{"startAngle":90.0},"type":"polar"}],"style":{"outline":{"a":0.0,"r":0,"b":0,"g":0},"fill":{"r":255,"b":255,"g":255}},"dimensions":[{"axis":[]}]}}],"legends":[{"labelStyle":{"fill":{"r":0,"b":157,"g":100},"font":{"size":"10pt","weight":"normal","family":"sans-serif"}},"itemStyle":{"padding":3,"size":11},"location":{"width":"24%"},"aesthetics":[{"aesthetic":{"$ref":"colorAestheticID"}}],"boundsStyle":{"padding":5,"outline":{"a":0.0,"r":0,"b":0,"g":0},"fill":{"a":0.0,"r":0,"b":0,"g":0}}}],"data":[{"id":"dSource","fields":[{"format":{"numericPattern":"###"},"id":"fVariable","label":"V4","categories":["Hyperm","Myopia"]},{"min":26.92307692307692,"max":73.07692307692307,"id":"fVariable1","label":"Y Axis"}],"rows":[[0,73.07692307692307],[1,26.92307692307692]]}],"size":{"width":850.0,"height":500.0},"style":{"outline":{"a":0.0,"r":0,"b":0,"g":0},"fill":{"r":255,"b":255,"g":255}},"titles":[{"backgroundStyle":{"outline":{"a":0.0,"r":0,"b":0,"g":0},"fill":{"a":0.0,"r":0,"b":0,"g":0}},"style":{"padding":3.0,"fill":{"r":0,"b":157,"g":100},"font":{"size":"12pt","weight":"bold","family":"sans-serif"}},"type":"title","content":["RE\_Myopia"]},{"backgroundStyle":{"outline":{"a":0.0,"r":0,"b":0,"g":0},"fill":{"a":0.0,"r":0,"b":0,"g":0}},"style":{"padding":3.0,"fill":{"r":0,"b":157,"g":100},"font":{"size":"10pt","weight":"bold","family":"sans-serif"}},"type":"title","content":["Albinism Type: OCA2"]}],"version":"6.0"}

Pie Chart  
Pie Chart - RE\_ref error for AlbinismType = OCA2 - May 11, 2022

{"copyright":"(C) Copyright IBM Corp. 2011","grammar":[{"elements":[{"color":[{"field":{"$ref":"fVariable"},"palette":["#00B2EF","#D9182D","#FFCF10","#7F1C7D","#007670","#DD731C","#17AF4B","#EE3D96","#838329","#82D1F5","#F04E37","#FFE14F","#AB1A86","#00A6A0","#F19027","#8CC63F","#F389AF","#A5A215","#00649D","#A91024","#FDB813","#3B2056","#006058","#B8471B","#00BA52","#BA006E","#594F13"],"id":"colorAestheticID"}],"data":{"$ref":"dSource"},"style":{"outline":{"r":0,"b":0,"g":0},"stroke":{"cap":"round","width":0.0,"join":"round"}},"position":[{"field":{"$ref":"fVariable1"}}],"type":"interval"}],"coordinates":{"transforms":[{"type":"stack"},{"polarParameters":{"startAngle":90.0},"type":"polar"}],"style":{"outline":{"a":0.0,"r":0,"b":0,"g":0},"fill":{"r":255,"b":255,"g":255}},"dimensions":[{"axis":[]}]}}],"legends":[{"labelStyle":{"fill":{"r":0,"b":157,"g":100},"font":{"size":"10pt","weight":"normal","family":"sans-serif"}},"itemStyle":{"padding":3,"size":11},"location":{"width":"24%"},"aesthetics":[{"aesthetic":{"$ref":"colorAestheticID"}}],"boundsStyle":{"padding":5,"outline":{"a":0.0,"r":0,"b":0,"g":0},"fill":{"a":0.0,"r":0,"b":0,"g":0}}}],"data":[{"id":"dSource","fields":[{"format":{"numericPattern":"###"},"id":"fVariable","label":"RE\_ref error","categories":["No","Yes"]},{"min":26.92307692307692,"max":73.07692307692307,"id":"fVariable1","label":"Y Axis"}],"rows":[[0,26.92307692307692],[1,73.07692307692307]]}],"size":{"width":850.0,"height":500.0},"style":{"outline":{"a":0.0,"r":0,"b":0,"g":0},"fill":{"r":255,"b":255,"g":255}},"titles":[{"backgroundStyle":{"outline":{"a":0.0,"r":0,"b":0,"g":0},"fill":{"a":0.0,"r":0,"b":0,"g":0}},"style":{"padding":3.0,"fill":{"r":0,"b":157,"g":100},"font":{"size":"12pt","weight":"bold","family":"sans-serif"}},"type":"title","content":["RE\_ref error"]},{"backgroundStyle":{"outline":{"a":0.0,"r":0,"b":0,"g":0},"fill":{"a":0.0,"r":0,"b":0,"g":0}},"style":{"padding":3.0,"fill":{"r":0,"b":157,"g":100},"font":{"size":"10pt","weight":"bold","family":"sans-serif"}},"type":"title","content":["Albinism Type: OCA2"]}],"version":"6.0"}

Pie Chart  
Pie Chart - RE\_cyl for AlbinismType = OCA2 - May 11, 2022

{"copyright":"(C) Copyright IBM Corp. 2011","grammar":[{"elements":[{"color":[{"field":{"$ref":"fVariable"},"palette":["#00B2EF","#D9182D","#FFCF10","#7F1C7D","#007670","#DD731C","#17AF4B","#EE3D96","#838329","#82D1F5","#F04E37","#FFE14F","#AB1A86","#00A6A0","#F19027","#8CC63F","#F389AF","#A5A215","#00649D","#A91024","#FDB813","#3B2056","#006058","#B8471B","#00BA52","#BA006E","#594F13"],"id":"colorAestheticID"}],"data":{"$ref":"dSource"},"style":{"outline":{"r":0,"b":0,"g":0},"stroke":{"cap":"round","width":0.0,"join":"round"}},"position":[{"field":{"$ref":"fVariable1"}}],"type":"interval"}],"coordinates":{"transforms":[{"type":"stack"},{"polarParameters":{"startAngle":90.0},"type":"polar"}],"style":{"outline":{"a":0.0,"r":0,"b":0,"g":0},"fill":{"r":255,"b":255,"g":255}},"dimensions":[{"axis":[]}]}}],"legends":[{"labelStyle":{"fill":{"r":0,"b":157,"g":100},"font":{"size":"10pt","weight":"normal","family":"sans-serif"}},"itemStyle":{"padding":3,"size":11},"location":{"width":"24%"},"aesthetics":[{"aesthetic":{"$ref":"colorAestheticID"}}],"boundsStyle":{"padding":5,"outline":{"a":0.0,"r":0,"b":0,"g":0},"fill":{"a":0.0,"r":0,"b":0,"g":0}}}],"data":[{"id":"dSource","fields":[{"format":{"numericPattern":"###"},"id":"fVariable","label":"V4","categories":["No","Yes"]},{"min":23.07692307692308,"max":76.92307692307693,"id":"fVariable1","label":"Y Axis"}],"rows":[[0,23.07692307692308],[1,76.92307692307693]]}],"size":{"width":850.0,"height":500.0},"style":{"outline":{"a":0.0,"r":0,"b":0,"g":0},"fill":{"r":255,"b":255,"g":255}},"titles":[{"backgroundStyle":{"outline":{"a":0.0,"r":0,"b":0,"g":0},"fill":{"a":0.0,"r":0,"b":0,"g":0}},"style":{"padding":3.0,"fill":{"r":0,"b":157,"g":100},"font":{"size":"12pt","weight":"bold","family":"sans-serif"}},"type":"title","content":["RE\_cyl"]},{"backgroundStyle":{"outline":{"a":0.0,"r":0,"b":0,"g":0},"fill":{"a":0.0,"r":0,"b":0,"g":0}},"style":{"padding":3.0,"fill":{"r":0,"b":157,"g":100},"font":{"size":"10pt","weight":"bold","family":"sans-serif"}},"type":"title","content":["Albinism Type: OCA2"]}],"version":"6.0"}

Pie Chart  
Pie Chart - LE\_Myopia for AlbinismType = OCA2 - May 11, 2022

{"copyright":"(C) Copyright IBM Corp. 2011","grammar":[{"elements":[{"color":[{"field":{"$ref":"fVariable"},"palette":["#00B2EF","#D9182D","#FFCF10","#7F1C7D","#007670","#DD731C","#17AF4B","#EE3D96","#838329","#82D1F5","#F04E37","#FFE14F","#AB1A86","#00A6A0","#F19027","#8CC63F","#F389AF","#A5A215","#00649D","#A91024","#FDB813","#3B2056","#006058","#B8471B","#00BA52","#BA006E","#594F13"],"id":"colorAestheticID"}],"data":{"$ref":"dSource"},"style":{"outline":{"r":0,"b":0,"g":0},"stroke":{"cap":"round","width":0.0,"join":"round"}},"position":[{"field":{"$ref":"fVariable1"}}],"type":"interval"}],"coordinates":{"transforms":[{"type":"stack"},{"polarParameters":{"startAngle":90.0},"type":"polar"}],"style":{"outline":{"a":0.0,"r":0,"b":0,"g":0},"fill":{"r":255,"b":255,"g":255}},"dimensions":[{"axis":[]}]}}],"legends":[{"labelStyle":{"fill":{"r":0,"b":157,"g":100},"font":{"size":"10pt","weight":"normal","family":"sans-serif"}},"itemStyle":{"padding":3,"size":11},"location":{"width":"24%"},"aesthetics":[{"aesthetic":{"$ref":"colorAestheticID"}}],"boundsStyle":{"padding":5,"outline":{"a":0.0,"r":0,"b":0,"g":0},"fill":{"a":0.0,"r":0,"b":0,"g":0}}}],"data":[{"id":"dSource","fields":[{"format":{"numericPattern":"###"},"id":"fVariable","label":"V4","categories":["Hyperm","Myopia"]},{"min":23.07692307692308,"max":76.92307692307693,"id":"fVariable1","label":"Y Axis"}],"rows":[[0,76.92307692307693],[1,23.07692307692308]]}],"size":{"width":850.0,"height":500.0},"style":{"outline":{"a":0.0,"r":0,"b":0,"g":0},"fill":{"r":255,"b":255,"g":255}},"titles":[{"backgroundStyle":{"outline":{"a":0.0,"r":0,"b":0,"g":0},"fill":{"a":0.0,"r":0,"b":0,"g":0}},"style":{"padding":3.0,"fill":{"r":0,"b":157,"g":100},"font":{"size":"12pt","weight":"bold","family":"sans-serif"}},"type":"title","content":["LE\_Myopia"]},{"backgroundStyle":{"outline":{"a":0.0,"r":0,"b":0,"g":0},"fill":{"a":0.0,"r":0,"b":0,"g":0}},"style":{"padding":3.0,"fill":{"r":0,"b":157,"g":100},"font":{"size":"10pt","weight":"bold","family":"sans-serif"}},"type":"title","content":["Albinism Type: OCA2"]}],"version":"6.0"}

Pie Chart  
Pie Chart - LE\_ref error for AlbinismType = OCA2 - May 11, 2022

{"copyright":"(C) Copyright IBM Corp. 2011","grammar":[{"elements":[{"color":[{"field":{"$ref":"fVariable"},"palette":["#00B2EF","#D9182D","#FFCF10","#7F1C7D","#007670","#DD731C","#17AF4B","#EE3D96","#838329","#82D1F5","#F04E37","#FFE14F","#AB1A86","#00A6A0","#F19027","#8CC63F","#F389AF","#A5A215","#00649D","#A91024","#FDB813","#3B2056","#006058","#B8471B","#00BA52","#BA006E","#594F13"],"id":"colorAestheticID"}],"data":{"$ref":"dSource"},"style":{"outline":{"r":0,"b":0,"g":0},"stroke":{"cap":"round","width":0.0,"join":"round"}},"position":[{"field":{"$ref":"fVariable1"}}],"type":"interval"}],"coordinates":{"transforms":[{"type":"stack"},{"polarParameters":{"startAngle":90.0},"type":"polar"}],"style":{"outline":{"a":0.0,"r":0,"b":0,"g":0},"fill":{"r":255,"b":255,"g":255}},"dimensions":[{"axis":[]}]}}],"legends":[{"labelStyle":{"fill":{"r":0,"b":157,"g":100},"font":{"size":"10pt","weight":"normal","family":"sans-serif"}},"itemStyle":{"padding":3,"size":11},"location":{"width":"24%"},"aesthetics":[{"aesthetic":{"$ref":"colorAestheticID"}}],"boundsStyle":{"padding":5,"outline":{"a":0.0,"r":0,"b":0,"g":0},"fill":{"a":0.0,"r":0,"b":0,"g":0}}}],"data":[{"id":"dSource","fields":[{"format":{"numericPattern":"###"},"id":"fVariable","label":"LE\_ref error","categories":["No","Yes","2"]},{"min":3.846153846153846,"max":65.38461538461539,"id":"fVariable1","label":"Y Axis"}],"rows":[[0,30.76923076923077],[1,65.38461538461539],[2,3.846153846153846]]}],"size":{"width":850.0,"height":500.0},"style":{"outline":{"a":0.0,"r":0,"b":0,"g":0},"fill":{"r":255,"b":255,"g":255}},"titles":[{"backgroundStyle":{"outline":{"a":0.0,"r":0,"b":0,"g":0},"fill":{"a":0.0,"r":0,"b":0,"g":0}},"style":{"padding":3.0,"fill":{"r":0,"b":157,"g":100},"font":{"size":"12pt","weight":"bold","family":"sans-serif"}},"type":"title","content":["LE\_ref error"]},{"backgroundStyle":{"outline":{"a":0.0,"r":0,"b":0,"g":0},"fill":{"a":0.0,"r":0,"b":0,"g":0}},"style":{"padding":3.0,"fill":{"r":0,"b":157,"g":100},"font":{"size":"10pt","weight":"bold","family":"sans-serif"}},"type":"title","content":["Albinism Type: OCA2"]}],"version":"6.0"}

Pie Chart  
Pie Chart - LE\_cyl for AlbinismType = OCA2 - May 11, 2022

{"copyright":"(C) Copyright IBM Corp. 2011","grammar":[{"elements":[{"color":[{"field":{"$ref":"fVariable"},"palette":["#00B2EF","#D9182D","#FFCF10","#7F1C7D","#007670","#DD731C","#17AF4B","#EE3D96","#838329","#82D1F5","#F04E37","#FFE14F","#AB1A86","#00A6A0","#F19027","#8CC63F","#F389AF","#A5A215","#00649D","#A91024","#FDB813","#3B2056","#006058","#B8471B","#00BA52","#BA006E","#594F13"],"id":"colorAestheticID"}],"data":{"$ref":"dSource"},"style":{"outline":{"r":0,"b":0,"g":0},"stroke":{"cap":"round","width":0.0,"join":"round"}},"position":[{"field":{"$ref":"fVariable1"}}],"type":"interval"}],"coordinates":{"transforms":[{"type":"stack"},{"polarParameters":{"startAngle":90.0},"type":"polar"}],"style":{"outline":{"a":0.0,"r":0,"b":0,"g":0},"fill":{"r":255,"b":255,"g":255}},"dimensions":[{"axis":[]}]}}],"legends":[{"labelStyle":{"fill":{"r":0,"b":157,"g":100},"font":{"size":"10pt","weight":"normal","family":"sans-serif"}},"itemStyle":{"padding":3,"size":11},"location":{"width":"24%"},"aesthetics":[{"aesthetic":{"$ref":"colorAestheticID"}}],"boundsStyle":{"padding":5,"outline":{"a":0.0,"r":0,"b":0,"g":0},"fill":{"a":0.0,"r":0,"b":0,"g":0}}}],"data":[{"id":"dSource","fields":[{"format":{"numericPattern":"###"},"id":"fVariable","label":"V4","categories":["No","Yes"]},{"min":19.23076923076923,"max":80.76923076923077,"id":"fVariable1","label":"Y Axis"}],"rows":[[0,19.23076923076923],[1,80.76923076923077]]}],"size":{"width":850.0,"height":500.0},"style":{"outline":{"a":0.0,"r":0,"b":0,"g":0},"fill":{"r":255,"b":255,"g":255}},"titles":[{"backgroundStyle":{"outline":{"a":0.0,"r":0,"b":0,"g":0},"fill":{"a":0.0,"r":0,"b":0,"g":0}},"style":{"padding":3.0,"fill":{"r":0,"b":157,"g":100},"font":{"size":"12pt","weight":"bold","family":"sans-serif"}},"type":"title","content":["LE\_cyl"]},{"backgroundStyle":{"outline":{"a":0.0,"r":0,"b":0,"g":0},"fill":{"a":0.0,"r":0,"b":0,"g":0}},"style":{"padding":3.0,"fill":{"r":0,"b":157,"g":100},"font":{"size":"10pt","weight":"bold","family":"sans-serif"}},"type":"title","content":["Albinism Type: OCA2"]}],"version":"6.0"}

Log  
Log - Log - May 11, 2022

FREQUENCIES VARIABLES=V1LogMar V2LogMar V3LogMar V4LogMar Flwup\_duration\_yrs Age\_lastFU  
  /FORMAT=NOTABLE  
  /NTILES=4  
  /STATISTICS=STDDEV VARIANCE RANGE MINIMUM MAXIMUM MEAN MEDIAN MODE  
  /ORDER=ANALYSIS.

Frequencies  
Frequencies - Statistics - May 11, 2022

StatisticsStatistics, table, 1 levels of column headers and 3 levels of row headers, table with 9 columns and 42 rows

|  |  |  |  |  |  |  |  |  |  |
| --- | --- | --- | --- | --- | --- | --- | --- | --- | --- |
| Albinism Type | | | V1 LogMar | V2 LogMar | V3 LogMar | V4 LogMar | years | Age\_lastFU |  |
| OCA1A | N | Valid | 16 | 16 | 8 | 5 | 17 | 16 |  |
|  |  | Missing | 1 | 1 | 9 | 12 | 0 | 1 |  |
|  | Mean | | .9500 | .8688 | .7125 | .6900 | 10.00 | 14.06 |  |
|  | Median | | .9000 | .9000 | .7000 | .8000 | 10.00 | 14.50 |  |
|  | Mode | | .90 | .90 | .70 | .80 | 10 | 11a |  |
|  | Std. Deviation | | .32863 | .20238 | .14577 | .21909 | 2.092 | 3.316 |  |
|  | Variance | | .108 | .041 | .021 | .048 | 4.375 | 10.996 |  |
|  | Range | | 1.40 | .80 | .50 | .55 | 8 | 13 |  |
|  | Minimum | | .60 | .50 | .40 | .35 | 5 | 9 |  |
|  | Maximum | | 2.00 | 1.30 | .90 | .90 | 13 | 22 |  |
|  | Percentiles | 25 | .8000 | .8000 | .7000 | .4750 | 9.50 | 11.00 |  |
|  |  | 50 | .9000 | .9000 | .7000 | .8000 | 10.00 | 14.50 |  |
|  |  | 75 | 1.0000 | .9750 | .8000 | .8500 | 11.50 | 16.00 |  |
| OCA1B | N | Valid | 26 | 28 | 13 | 5 | 27 | 28 |  |
|  |  | Missing | 2 | 0 | 15 | 23 | 1 | 0 |  |
|  | Mean | | .6000 | .5161 | .5000 | .6000 | 8.74 | 13.21 |  |
|  | Median | | .6000 | .5000 | .5000 | .6000 | 9.00 | 12.50 |  |
|  | Mode | | .60 | .30a | .30a | .60a | 9 | 11 |  |
|  | Std. Deviation | | .16248 | .19581 | .22361 | .12247 | 2.105 | 2.923 |  |
|  | Variance | | .026 | .038 | .050 | .015 | 4.430 | 8.545 |  |
|  | Range | | .70 | .80 | .70 | .30 | 9 | 10 |  |
|  | Minimum | | .30 | .20 | .10 | .40 | 3 | 9 |  |
|  | Maximum | | 1.00 | 1.00 | .80 | .70 | 12 | 19 |  |
|  | Percentiles | 25 | .5000 | .3250 | .3000 | .5000 | 8.00 | 11.00 |  |
|  |  | 50 | .6000 | .5000 | .5000 | .6000 | 9.00 | 12.50 |  |
|  |  | 75 | .7000 | .6000 | .7000 | .7000 | 10.00 | 16.00 |  |
| OCA2 | N | Valid | 23 | 24 | 12 | 9 | 26 | 26 |  |
|  |  | Missing | 3 | 2 | 14 | 17 | 0 | 0 |  |
|  | Mean | | .7043 | .6250 | .5333 | .5278 | 7.92 | 13.08 |  |
|  | Median | | .8000 | .7000 | .6000 | .5500 | 9.00 | 13.00 |  |
|  | Mode | | .80 | .70 | .60 | .80 | 9 | 9 |  |
|  | Std. Deviation | | .18210 | .19393 | .22293 | .23333 | 2.399 | 3.199 |  |
|  | Variance | | .033 | .038 | .050 | .054 | 5.754 | 10.234 |  |
|  | Range | | .70 | .80 | .70 | .70 | 10 | 10 |  |
|  | Minimum | | .30 | .30 | .10 | .10 | 3 | 9 |  |
|  | Maximum | | 1.00 | 1.10 | .80 | .80 | 13 | 19 |  |
|  | Percentiles | 25 | .5000 | .5000 | .3250 | .3500 | 6.00 | 10.00 |  |
|  |  | 50 | .8000 | .7000 | .6000 | .5500 | 9.00 | 13.00 |  |
|  |  | 75 | .8000 | .7750 | .7500 | .7500 | 9.00 | 16.00 |  |
|  |  |  |  |  |  |  |  |  |  |
| --- | --- | --- | --- | --- | --- | --- | --- | --- | --- |
| a. Multiple modes exist. The smallest value is shown | | | | | | | | |  |
|  |  |  |  |  |  |  |  |  |  |

Log  
Log - Log - May 11, 2022

GET  
  FILE='D:\liavofra\עבודה\לקוחות\Hadassah\Claudia\Albinism\WF2\_new.sav'.  
DATASET NAME DataSet1 WINDOW=FRONT.

IBM SPSS Web Report

X

ABOUT

|  |
| --- |
| Created Using: IBM SPSS Statistics 26 |
| Creation Date: May 11, 2022 |
| Document Version: OriginalSaved Copy |
| Saved Date:  May 11, 2022 |

Navigation Controls

|  |
| --- |
| Contents - Opens and closes the list of charts and tables in the Web Report |
| Next & Previous - Display the next or previous table or chart in the Web Report |
| Help - Opens Help |

Toolbar Buttons

|  |  |
| --- | --- |
|  | Undo - Undoes the last change in the document. |
|  | Edit - Open the Editor tool for tables and charts. Certain editing options are only available when you are connected to an Internet server. |
|  | Save - Creates a new copy of the Web Report with the saved changes. |
|  | Print - Prints the current object when in Object View and all objects in Page View. |
|  | Page View - Switches the Web Report to display all the tables and charts on a single page. |
|  | Object View - Switches the Web Report so that each table or chart is displayed one at a time. |

Connecting to a Server

:   The status of the Web Report's connection to an Internet server appears in the top right corner of the Web Report.
:   An Internet connection is not required to open a Web Report. With a saved copy of the Web Report you can view all of the charts and tables, and have some limited editing ability, when not connected to the Internet.
:   Connecting a Web Report to an Internet server will enable far greater editing capabilities for tables and for charts.

- If the author specified an Internet server when they created the Web Report, the Web Report will attempt to connect to the server automatically when it is opened.
- If the Web Report does not connect to a server, click on the server Status Message to open tools to retry the connection, try a different server, or enter a new server address.
- For information about adding the enhanced controls to your Internet Server, go to https://developer.ibm.com/predictiveanalytics.
- If you specify a new server connection, the preferred format is http://xxx.xxx.xxx.xxx:xxxx.

Editing Tables

|  |  |
| --- | --- |
| Some of this functionality is only available when connected to an Internet server. | |
|  | Create a chart - Create a chart from the selected cells in the table. |
|  | Pivot and Sort - Transpose, sort, and pivot the table. |
|  | Background color - The background color of the selected cells. |
|  | Text Color and Style - Font color, style, and size. |
|  | Number Format - Font color, style, and size. |

Editing Charts

|  |  |
| --- | --- |
| All of this functionality is only available when connected to an Internet server. | |
|  | Chart Size - Change the height and width of the chart |
|  | Background color - The background color of the selected object. |
|  | Border and Line Style - The color and thickness of the line or border. |
|  | Text Color and Style - Font color, style, and size. |
|  | Number Format - Font color, style, and size. |
|  | Axis Properties - Change the scale and display axis titles and ticks. |

Save

X
New Name  
   
  
What to Save   

Save the entire document  
Only save the current object

Server Connection

X
  
Saved Server Connections  
    http://192.168.36.112:9080/webreport/   
  
  
  
Status  
Cannot connect to the specified server.

Add a chart

Pivot and Sort

Chart Size   
  

|  |  |  |
| --- | --- | --- |
|  |  |  |
|  |  |  |
| Lock aspect ratio | | |

Background   

|  |  |  |  |  |  |
| --- | --- | --- | --- | --- | --- |
|  | |  | |  | |
|  |  |  |  |  |  |
|  |  |  |  |  |  |
|  |  |  |  |  |  |

Line and Borders   

|  |  |  |  |  |  |
| --- | --- | --- | --- | --- | --- |
|  | |  | |  | |
|  |  |  |  |  |  |
|  |  |  |  |  |  |
|  |  |  |  |  |  |

  

|  |  |  |
| --- | --- | --- |
|  |  |  |

Text Format   

|  |  |  |  |  |  |
| --- | --- | --- | --- | --- | --- |
|  | |  | |  | |
|  |  |  |  |  |  |
|  |  |  |  |  |  |
|  |  |  |  |  |  |

  

|  |  |  |
| --- | --- | --- |
|  |  |  |

  

|  |  |  |  |
| --- | --- | --- | --- |
|  |  |  | Font Family  Agency FB Aharoni Algerian Arial Arial Black Arial Narrow Arial Rounded MT Bold Bahnschrift Baskerville Old Face Bauhaus 93 Bell MT Berlin Sans FB Berlin Sans FB Demi Bernard MT Condensed Blackadder ITC Bodoni MT Bodoni MT Black Bodoni MT Condensed Bodoni MT Poster Compressed Book Antiqua Bookman Old Style Bookshelf Symbol 7 Bradley Hand ITC Britannic Bold Broadway Brush Script MT Calibri Calibri Light Californian FB Calisto MT Cambria Cambria Math Candara Candara Light Castellar Centaur Century Century Gothic Century Schoolbook Chiller Colonna MT Comic Sans MS Consolas Constantia Cooper Black Copperplate Gothic Bold Copperplate Gothic Light Corbel Corbel Light Courier New Curlz MT David Dialog DialogInput Dubai Dubai Light Dubai Medium Ebrima Edwardian Script ITC Elephant Engravers MT Eras Bold ITC Eras Demi ITC Eras Light ITC Eras Medium ITC Felix Titling Footlight MT Light Forte Franklin Gothic Book Franklin Gothic Demi Franklin Gothic Demi Cond Franklin Gothic Heavy Franklin Gothic Medium Franklin Gothic Medium Cond FrankRuehl Freestyle Script French Script MT Gabriola Gadugi Garamond Georgia Gigi Gill Sans MT Gill Sans MT Condensed Gill Sans MT Ext Condensed Bold Gill Sans Ultra Bold Gill Sans Ultra Bold Condensed Gisha Gloucester MT Extra Condensed Goudy Old Style Goudy Stout Guttman Aharoni Guttman Drogolin Guttman Frank Guttman Frnew Guttman Haim Guttman Haim-Condensed Guttman Hatzvi Guttman Kav Guttman Kav-Light Guttman Logo1 Guttman Mantova Guttman Mantova-Decor Guttman Miryam Guttman Myamfix Guttman Rashi Guttman Stam Guttman Stam1 Guttman Vilna Guttman Yad Guttman Yad-Brush Guttman Yad-Light Guttman-Aharoni Guttman-Aram Guttman-CourMir Hadassah Friedlaender Haettenschweiler Harlow Solid Italic Harrington HelvNeue Roman for IBM High Tower Text HoloLens MDL2 Assets Impact Imprint MT Shadow Informal Roman Ink Free Javanese Text Jokerman Juice ITC Kristen ITC Kunstler Script Leelawadee Leelawadee UI Leelawadee UI Semilight Levenim MT Lucida Bright Lucida Calligraphy Lucida Console Lucida Fax Lucida Handwriting Lucida Sans Lucida Sans Typewriter Lucida Sans Unicode Magneto Maiandra GD Malgun Gothic Malgun Gothic Semilight Marlett Matura MT Script Capitals Microsoft Himalaya Microsoft JhengHei Microsoft JhengHei Light Microsoft JhengHei UI Microsoft JhengHei UI Light Microsoft New Tai Lue Microsoft PhagsPa Microsoft Sans Serif Microsoft Tai Le Microsoft Uighur Microsoft YaHei Microsoft YaHei Light Microsoft YaHei UI Microsoft YaHei UI Light Microsoft Yi Baiti MingLiU-ExtB MingLiU\_HKSCS-ExtB Miriam Miriam Fixed Mistral Modern No. 20 Mongolian Baiti Monospaced Monotype Corsiva MS Gothic MS Outlook MS PGothic MS Reference Sans Serif MS Reference Specialty MS UI Gothic MT Extra MV Boli Myanmar Text Narkisim Niagara Engraved Niagara Solid Nirmala UI Nirmala UI Semilight NSimSun OCR A Extended Old English Text MT Onyx Palace Script MT Palatino Linotype Papyrus Parchment Perpetua Perpetua Titling MT Playbill PMingLiU-ExtB Poor Richard Pristina Rage Italic Ravie Rockwell Rockwell Condensed Rockwell Extra Bold Rod SansSerif Script MT Bold Segoe MDL2 Assets Segoe Print Segoe Script Segoe UI Segoe UI Black Segoe UI Emoji Segoe UI Historic Segoe UI Light Segoe UI Semibold Segoe UI Semilight Segoe UI Symbol Serif Showcard Gothic SimSun SimSun-ExtB Sitka Banner Sitka Display Sitka Heading Sitka Small Sitka Subheading Sitka Text Snap ITC Stencil Sylfaen Symbol Tahoma TeamViewer15 Tempus Sans ITC Times New Roman Trebuchet MS Tw Cen MT Tw Cen MT Condensed Tw Cen MT Condensed Extra Bold Verdana Viner Hand ITC Vivaldi Vladimir Script Webdings Wide Latin Wingdings Wingdings 2 Wingdings 3 Yu Gothic Yu Gothic Light Yu Gothic Medium Yu Gothic UI Yu Gothic UI Light Yu Gothic UI Semibold Yu Gothic UI Semilight |

Number Format   
  

|  |  |  |
| --- | --- | --- |
| 0.00 |  |  |

Axis Options   
  

|  |  |  |
| --- | --- | --- |
|  |  |  |
|  |  |  |
| Display Axis Title | | | |
| Display Ticks | | | |
